# Supplementary material for: Ring-closing C–O/C–O metathesis of ethers with primary aliphatic alcohols
Source: Nat Commun. 2023 Apr 5;14:1883. doi: 10.1038/s41467-023-37538-1 (PMC10076310; doi:10.1038/s41467-023-37538-1)
Supplement: Supplementary file 1 — Supplementary Information [file 41467_2023_37538_MOESM1_ESM.pdf]

Supplementary Information for

Ring-closing C–O/C–O Metathesis of Ethers with Primary Aliphatic Alcohols

Hongmei Liu,[1] Qing Huang,[1] Rong-zhen Liao,[1] Man Li,[1]* & Youwei Xie[1]*

[1]Hubei Key Laboratory of Bioinorganic Chemistry and Materia Medica; Key Laboratory of Material Chemistry for Energy Conversion and Storage, Ministry of Education; Hubei Key Laboratory of Materials Chemistry and Service Failure; School of Chemistry and Chemical .Engineering, Huazhong University of Science and Technology, 1037 Luoyu Road, Wuhan, 430074, China

*e-mail: manli_hx@hust.edu.cn, xieyw@hust.edu.cn

Table of Contents

1. Supplementary Note 1 2

2. Supplementary Note 2 3

3. Supplementary Methods 4

3.1 Syntheses of starting materials and spectroscopic data 4

3.2 Ring-Closing C–O/C–O Cross Metathesis of Primary aliphatic Alcohols with Ethers 17

4. Supplementary Discussion 27

4.1 Mechanistic experiments 27

4.2 DFT calculations 33

4.3 Calculated energies (in hartree) for all stationary points 42

5. Supplementary Note 3 45

6. Supplementary References 139

1. Supplementary Note 1

Re_2O_7 was purchased from Sigma Aldrich. HFIP used in the reactions was purchased from Leyan. Other chemicals used in this manuscript were purchased from Energy chemical company, Bide Pharmatech Ltd, Inno-Chem Ltd, Adamas Company, Alfa Aesar Company and Cambridge Isotope Laboratories, Inc. Other commercially available compounds were used as provided without further purification. Unless otherwise noted, all reactions were performed under air. Reactions were monitored by thin layer chromatography (TLC) on silica gel pre-coated plastic sheets (0.2 mm). Visualization was accomplished by irradiation with UV light at 254 nm and $KMnO_4$. Flash column chromatography was performed over silica gel (200-300 mesh). ^{1}H-, ^{13}C-, ^{19}F and ^{31}P -NMR spectra were recorded on Bruker AV400 or Bruker AscendTM 600MHz at room temperature. Chemical shifts were reported in ppm on the scale relative to $CDCl_3$ (δ = 7.26 for ^{1}H-NMR, δ = 77.00 for ^{13}C-NMR). Proton spectrum description analysis is as follows: chemical shift (ppm), multiplet analysis (s = singlet, d = doublet, t = triplet, q = quartet, m = multiplet), unidentified coupling the methods are all analyzed by multiple peak processing, and the carbon spectrum is described in ppm. Coupling constants (*J*) were reported in Hertz (Hz). High resolution mass spectra (HR-MS) were determined on Bruker SolariX 7.0T FT-MS (ESI source). Mass spectra (GC-MS) were determined on Agilent 7890A/5975C (EI source).

2. Supplementary Note 2 [a]

Supplementary Table 1. Optimization of the reaction condition

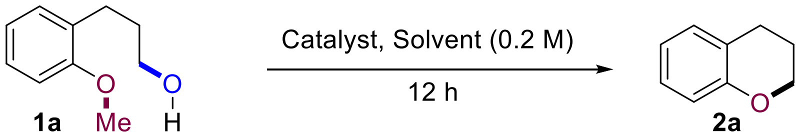

Entry	Catalyst	loading	Solvent	T (°C)	^{1}H-NMR yield
1	$FeCl_3$	5%	*n*-hexane	100	trace
2	$Fe(OTf)_3$	5%	*n*-hexane	100	3%
3	$Fe(OTf)_3$	2%	DCE	100	trace
4	$Fe(OTf)_3$	5%	DCE	100	10%
5	$Fe(OTf)_3$	2%	HFIP	100	67%
6	$Bi(OTf)_3$	5%	DCE	100	10%
7	$Bi(OTf)_3$	2%	HFIP	100	70%
8	$CuCl_2$	5%	DCE or HFIP	100	NR
9	$AlCl_3$	5%	DCE or HFIP	100	NR
10	$FeCl_3$	5%	DCE or HFIP	100	NR
11	$ZnCl_2$	5%	DCE	100	NR
12	V_2O_5	5%	DCE	100	NR
13	CF_3SO_3H	5%	DCE	100	14%
14	$TsOH \cdot H_2O$	5%	DCE or HFIP	100	trace
15	CF_3COOH	5%	DCE or HFIP	100	NR
16	HCl	5%	DCE or HFIP	100	NR
17	Re_2O_7	2%	DCE	100	30%
18	$HReO_4$	5%	DCE	100	24%
19	$MeReO_3$(MTO)	5%	DCE	100	NR
20	$(CO)_5ReBr$	5%	DCE	100	NR
21	$Re_2(CO)_{10}$	5%	DCE	100	NR
22	Re_2O_7	2%	*n*-hexane	100	NR
23	Re_2O_7	2%	toluene	100	trace
24	Re_2O_7	2%	1,4-dioxane	100	trace
25	Re_2O_7	2%	CF_3CH_2OH	100	54%
26	Re_2O_7	2%	$CF_2HCF_2CH_2OH$	100	60%
**27**	**Re_2O_7**	**2%**	**HFIP**	**100**	86% (85%[b])
28	Re_2O_7	1%	HFIP	100	70%
29	Re_2O_7	2%	HFIP	80	69%

[a] Reactions were performed with **1a** (1.0 equiv.), solvent (0.2 M), catalyst (1-5 mol%) at 100 °C for 12 hours. [b] Isolated yield.

3. Supplementary Methods

3.1 Syntheses of starting materials and spectroscopic data

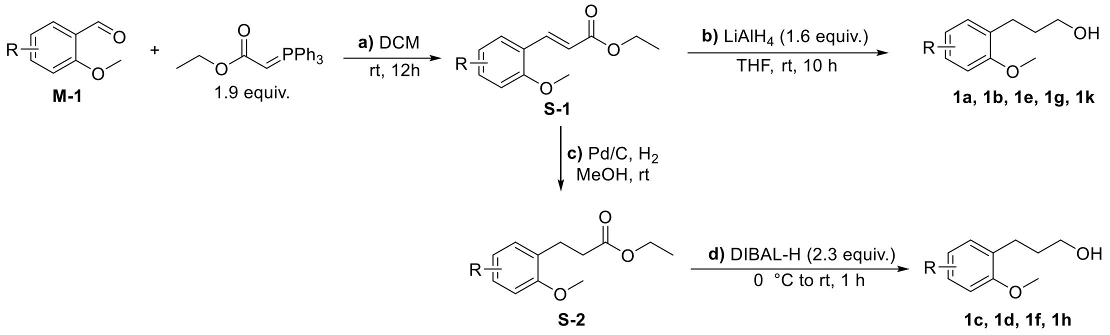

Supplementary Figure 1. General synthetic method A

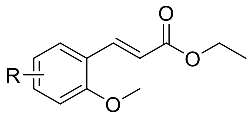

S-1

a) (Carbethoxmethylene)triphenylphosphorane (10 mmol, 1.9 equiv.) was added to a solution of aldehyde (**M-1**) in anhydrous DCM (10 mL) and the reaction mixture stirred at room temperature until TLC showed complete conversion of aldehyde. The reaction mixture was concentrated under reduced pressure and purified by column chromatography on silica gel (SiO_2, PE/EA = 50/1) to afford α, β-unsaturated ester (98% yield).

b) A solution of **S-1** (5.0 mmol, 1.0 equiv.) in dry THF (10 mL) was added to a suspension of $LiAlH_4$ (2.5 M in THF) (1.6 equiv.) at 0 °C under N_2 atmosphere. The resulting mixture was allowed to warm to room temperature and stirred for overnight until TLC showed complete conversion of **S-1**. The reaction mixture was quenched with HCl (3 ml, 1M) and then stirred at room temperature for 1 hour. The organic phase was separated and the aqueous phase was extracted with ethyl acetate (3×10 mL). The combined organic layers were dried with anhydrous Na_2SO_4, filtered, and concentrated under reduced pressure. Purification by column chromatography on silica gel (SiO_2, PE/EA = 5/1) to give **1a** (95% yield) as a pale-yellow oil.

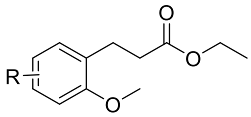

S-2

c) Pd/C (10% on carbon, wetted with ca. 55% water) was added into the reaction flask, filled with nitrogen and purged with hydrogen three times. Methanol and **S-1** were added to the reaction flask. The mixture was stirred at 45 °C for 24 hours and filtered through a pad of silica gel. The filtrate was concentrated to give a crude product **S-2**, which was used in the following step without further purification.

d) To an ice cooled solution of **S-2** (5.0 mmol, 1.0 equiv.) in dichloromethane (10 mL) a solution of DIBAL-H (1.5 M in dichloromethane, 2.30 equiv.) was added slowly over 15 min. The reaction mixture was stirred at 0 °C for 30 min, and then at room temperature for 2 hours. After that, the reaction mixture was cooled to 0 °C (ice bath) again and the excess DBAL-H was quenched by sequential addition of water (2.0 mL), NaOH solution (1.5 mL, 10% aqueous) and water (2.0 mL). The ice bath was removed and the suspension was stirred for at room temperature for 1 hour. Afterwards, the suspension was filtered, dried over anhydrous Na_2SO_4, filtered and concentrated under reduced pressure to give an oil. Purification by column chromatography on silica gel (SiO_2, PE/EA = 5/1) to give **1a** (72% yield) as a pale-yellow oil. The procedure d was according to the literature report[1].

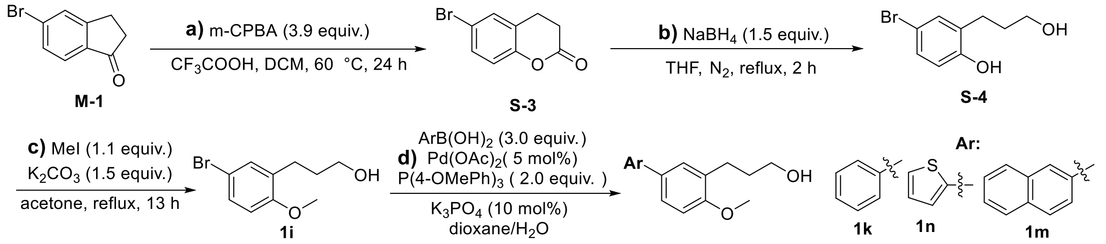

Supplementary Figure 2. General synthetic method B

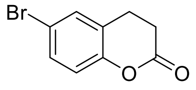

S-3

a) To a solution of **M-1** (8.46 g, 40.0 mmol, 1.0 equiv.) and m-chloroperoxybenzoic acid (26.92 g, 156 mmol, 3.9 equiv.) in CH_2Cl_2 (100 ml) was added TFA (2.3 ml) and the mixture was heated to 60 ºC and stirred at this temperature for 14 hours. The reaction mixture was cooled to room temperature and washed with aqueous NaOH (20.0 mL, 0.1 M), sat. aqueous $NaHCO_3$ (20 mL) and brine. The organic phase was dried over anhydrous $MgSO_4$ and concentrated under reduced pressure. The residue was purified by flash chromatography (SiO_2, PE/EA = 10/1) to yield 5.71 g (63 %) of the **S-3** as a yellow oil. The procedure a was according to the literature report[2].

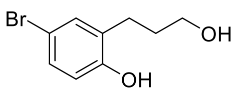

S-4

b) To a solution of $NaBH_4$ (1.17g, 30 mmol, 1.5 equiv.) in THF (40 mL) was added **S-3** (4.54g, 20 mmol, 1.0 equiv.) and the mixture was refluxed for 2 hours. The solution was quenched with saturated aqueous NH_4Cl (3.0 mL) and the reaction mixture was extracted with ethyl acetate (3 × 15 mL), dried over anhydrous $MgSO_4$, filtered and concentrated under reduced pressure. The resulting crude material was purified by flash column chromatography (SiO_2, PE/EA = 1/1) to give α, β-unsaturated ester **S-4** (3.00 g, 65% yield) as a white solid.

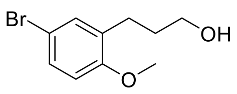

1i

c) To a 25 mL round bottom flask, diol **S-4** (395.2 mg, 1.61 mmol), anhydrous acetone (8 mL), K_2CO_3 (331.1 mg, 2.42 mmol, 1.5 equiv.), iodomethane (251.3 mg, 110 uL, 1.1 equiv.) were added. Reaction was refluxed for 10 hours. After cooling to rt, solvent was removed under reduced pressure, and the residue was dissolved in a EtOAc/H_2O (1:1) mixture. The product was extracted with EtOAc (3×5 mL), washed with H_2O (5 mL) and brine, then dried over anhydrous Na_2SO_4, filtered and concentrated under reduced pressure. The resulting crude material was purified by flash column chromatography (SiO_2, PE/EA = 5/ 1) to give **1i** (354.0 mg, 90% yield) as a pale-yellow oil. The procedure c was according to the literature report[3].

d) 1i (0.49 g, 2 mmol) was dissolved in 1,4-dioxane (1.5 mL) and H_2O (1.5 mL) and to the solution were added $Pd(OAc)_2$ (22.56 mg, 5 mol%), $P(4\text{-}MeOC_6H_4)_3$ (71.96 mg, 10 mol%), arylboronic acid (3.0 mmol, 1.5 equiv.), and K_3PO_4 (0.647g, 3 mmol). After stirring at 120 ºC for 12 hours, the reaction was quenched with H_2O (1.5 mL), and organic materials were extracted with EtOAc (3×7 mL). The combined extracts were washed with brine and dried over anhydrous Na_2SO_4. After removal of the solvent under reduced pressure, the residue was purified by silica gel column chromatography (SiO_2, PE/EA = 5/ 1) to give the corresponding product (45-60% yield). The procedure d was according to the literature report[4].

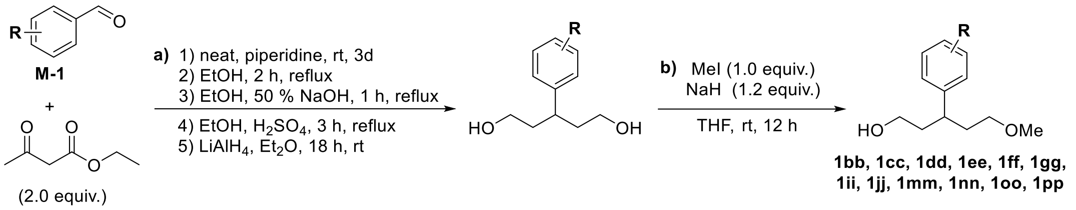

Supplementary Figure 3. General synthetic method C

a) A solution of the corresponding aldehyde (**M-1**) (20 mmol, 1.0 equiv.) in ethyl acetoacetate (40 mmol, 2.0 equiv.) was treated with piperidine (0.20 mL, 0.1 equiv.) and stirred at room temperature for 3 days. The resulting solid residue was dissolved in EtOH (20 mL, 1M) and refluxed for 2 hours. The solution was cooled to room temperature and the resulting crystalline product was filtered and washed with cold MTBE and dried in vacuo. This solid was dissolved in ethanol (20 mL, 0.5 mL) and treated with an 50% aqueous sodium hydroxide solution (100 equiv.), then refluxed for 1 hour. Afterwards, the organic solvent was removed under reduced pressure and the resulting aqueous phase was carefully acidified with conc. hydrochloric acid to pH = 1 in an ice bath (Careful, the reaction is highly exothermic!) and extracted with EtOAc (3×15 mL). The organic layers were combined, dried with anhydrous Na_2SO_4 and concentrated under reduced pressure. The resulting diacid was dissolved in ethanol (20 mL, 0.5 M) and 10 drops of concentrated sulfuric acid were added. The reaction mixture was refluxed for 3 hours. Afterwards, the organic solvent was removed under reduced pressure and the solid residue was dissolved in water (20 mL) and EtOAc (20 mL). The organic phase was separated and the aqueous phase was extracted with EtOAc (3×25 mL). The combined organic layers were dried with anhydrous Na_2SO_4 and concentrated under reduced pressure. The resulting diester was dissolved in dry THF (0.5 M) and cooled to 0 °C. Lithium aluminium hydride (2.0 equiv.) was carefully added and the reaction mixture was stirred at rt for 18 hours. After a FIESER workup, the obtained diols can be used without further purification for the next step. The procedure a was according to the literature report[5].

FIESER Workup – How to work up reactions containing x g of $LiAlH_4$

1. Dilute the reaction mixture with MTBE or diethyl ether and cool down to 0 °C
2. Add x g of H_2O carefully dropwise
3. Add x g of 15% aqueous NaOH carefully dropwise
4. Add 3x g of H_2O carefully dropwise
5. Allow the mixture to warm up to rt and stir for 20 min
6. Add Na_2SO_4 to the mixture and stir for 15 min
7. Filter the suspension over celite to obtain a clear solution of the crude product

b) Sodium hydride (6.2 mmol, 1.2 equiv.) was added at room temperature to a solution of alcohol (7.7 mmol 1.5 equiv.) in dry THF (10 mL). The medium was stirred at room temperature during 1 hour and CH_3I (5.1 mmol, 1.0 equiv.) was added slowly. After stirring at room temperature for 12 hours, water (2 mL) was added slowly to quench the reaction and the organic layer was extracted with ethyl acetate (3× 10 mL), dried with anhydrous Na_2SO_4 and concentrated under reduced pressure. The monoalkylated derivative was isolated as a colorless oil after purification by column chromatography (SiO_2, PE/EA = 3/ 1). The procedure b was according to the literature report[6].

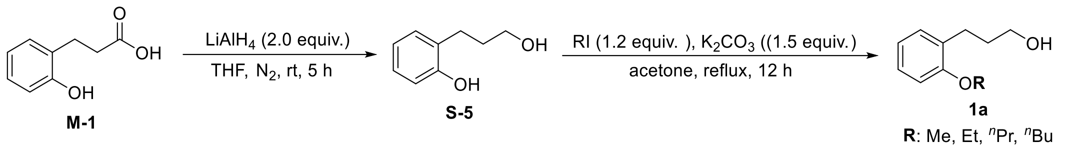

Supplementary Figure 4. Synthesis of **1a** with different R groups

S-5 was made from the **M-1** as the step b in Supplementary Figure 1, which is better than 90% in yield. The substrate **1a** with different R groups (Et, *n*-Pr, *n*-Bu) are made as the step c in Supplementary Figure 2, the yields are better than 85%.

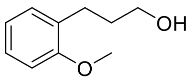

1a: 3-(2-methoxyphenyl)propan-1-ol[7].
Colorless oil. **^{1}H NMR** (400 MHz, $CDCl_3$) δ 7.24 – 7.09 (m, 2H), 6.94 – 6.83 (m, 2H), 3.84 (s, 3H), 3.60 (t, *J* = 6.3 Hz, 2H), 2.73 (t, *J* = 7.3 Hz, 2H), 1.89 – 1.82 (m, 2H).

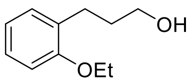

3-(2-propoxyphenyl)propan-1-ol.
Colorless oil. **^{1}H NMR** (400 MHz, $CDCl_3$) δ 7.16 (m, 2H), 6.93 – 6.82 (m, 2H), 4.06 (q, *J* = 7.0 Hz, 2H), 3.58 (t, *J* = 6.2 Hz, 2H), 2.75 (t, *J* = 7.2 Hz, 2H), 1.90 – 1.81 (m, 2H), 1.44 (t, *J* = 7.0 Hz, 3H). **^{13}C NMR** (151 MHz, $CDCl_3$) δ 156.65, 130.07, 129.99, 127.01, 120.54, 111.21, 63.52, 61.61, 32.88, 25.75, 14.77. **HRMS (ESI)** m/z: $M+H^+$ calculated for $C_{11}H_{17}O_2^+$: 181.1223, found 181.12215.

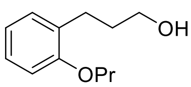

3-(2-propoxyphenyl)propan-1-ol.
Colorless oil. **^{1}H NMR** (400 MHz, $CDCl_3$) δ 7.19 (m, 2H), 6.97 – 6.82 (m, 2H), 3.97 (t, *J* = 6.5 Hz, 2H), 3.62 (t, *J* = 6.2 Hz, 2H), 2.77 (t, *J* = 7.2 Hz, 2H), 1.91 – 1.83 (m, 4H), 1.08 (t, *J* = 7.4 Hz, 3H). **^{13}C NMR** (101 MHz, $CDCl_3$) δ 156.80, 130.07, 130.03, 127.04, 120.47, 111.20, 69.52, 61.79, 32.91, 25.88, 22.63, 10.60. **HRMS (ESI)** m/z: $M+H^+$ calculated for $C_{12}H_{19}O_2^+$: 195.1380, found 195.1381.

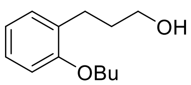

3-(2-butoxyphenyl) propan-1-ol.
Colorless oil. **^{1}H NMR** (400 MHz, $CDCl_3$) δ 7.22 – 7.09 (m, 2H), 6.93 – 6.80 (m, 2H), 3.99 (t, *J* = 6.5 Hz, 2H), 3.59 (t, *J* = 6.2 Hz, 2H), 2.74 (t, *J* = 7.2 Hz, 2H), 1.89 – 1.75 (m, 4H), 1.58 – 1.43 (m, 2H), 0.99 (t, *J* = 7.4 Hz, 3H). **^{13}C NMR** (101 MHz, $CDCl_3$) δ 156.85, 130.11, 130.02, 127.08, 120.51, 111.20, 67.74, 61.80, 32.97, 31.39, 25.85, 19.31, 13.82. **HRMS (ESI)** m/z: $M+H^+$ calculated for $C_{13}H_{21}O_2^+$: 209.1536, found 209.1538.

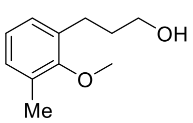

1b: 3-(2-methoxy-3-methylphenyl) propan-1-ol. 1b was synthesized according to the general synthetic method A.
Colorless oil. **^{1}H NMR** (400 MHz, $CDCl_3$) δ 7.10 – 6.89 (m, 3H), 3.75 (s, 3H), 3.57 (t, *J* = 6.1 Hz, 2H), 2.75 (t, *J* = 7.3 Hz, 2H), 2.30 (s, 3H), 1.90 – 1.78 (m, 2H). **^{13}C NMR** (151 MHz, $CDCl_3$) δ 156.68, 134.19, 130.82, 129.20, 127.89, 124.20, 61.48, 60.47, 33.50, 25.55, 16.15. **HRMS (ESI)** m/z: $M+H^+$ calculated for $C_{11}H_{17}O_2^+$: 181.1223, found 181.12217.

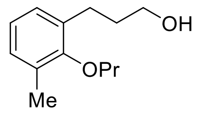

1b': 3-(3-methyl-2-propoxyphenyl) propan-1-ol. 1b' was synthesized according to the general synthetic method A.

Colorless oil. **^{1}H NMR** (400 MHz, $CDCl_3$) δ 7.04 – 6.95 (m, 3H), 3.75 (t, J = 6.7 Hz, 2H), 3.54 (t, J = 6.0 Hz, 2H), 2.74 (t, J = 7.2 Hz, 2H), 2.29 (s, 3H), 1.88 – 1.81 (m, 4H), 1.07 (t, J = 7.5 Hz, 3H). **^{13}C NMR** (101 MHz, $CDCl_3$) δ 155.68, 134.25, 130.93, 129.14, 127.88, 124.08, 74.76, 61.27, 33.50, 25.60, 23.51, 16.32, 10.51. **HRMS (ESI)** m/z: M+H$^+$ calculated for $C_{13}H_{21}O_2^+$: 209.1536, found 209.1537.

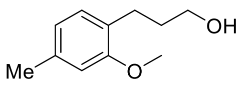

1c: 3-(2-methoxy-4-methylphenyl)propan-1-ol. 1c was synthesized according to the general synthetic method A.

Colorless oil. **^{1}H NMR** (400 MHz, $CDCl_3$) δ 7.02 (d, J = 7.5 Hz, 1H), 6.72 (d, J = 7.4 Hz, 1H), 6.68 (s, 1H), 3.82 (s, 3H), 3.59 (t, J = 6.3 Hz, 2H), 2.68 (t, J = 7.3 Hz, 2H), 2.34 (s, 3H), 1.86 – 1.78 (m, 2H). **^{13}C NMR** (101 MHz, $CDCl_3$) δ 157.17, 136.94, 129.80, 126.78, 121.18, 111.25, 61.84, 55.23, 32.94, 25.48, 21.35. **HRMS (ESI)** m/z: M+H$^+$ calculated for $C_{11}H_{17}O_2^+$: 181.1223, found 181.12217.

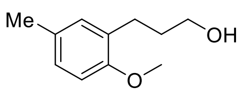

1d: 3-(2-methoxy-5-methylphenyl) propan-1-ol. 1d was synthesized according to the general synthetic method A.

Colorless oil. **^{1}H NMR** (400 MHz, $CDCl_3$) δ 7.04 – 6.90 (m, 2H), 6.76 (d, J = 8.1 Hz, 1H), 3.81 (s, 3H), 3.59 (t, J = 6.2 Hz, 2H), 2.69 (t, J = 7.3 Hz, 2H), 2.27 (s, 3H), 1.84 (p, J = 6.7 Hz, 2H). **^{13}C NMR** (151 MHz, $CDCl_3$) δ 155.27, 130.90, 129.88, 129.63, 127.36, 110.29, 61.82, 55.50, 33.00, 25.76, 20.41. **HRMS (ESI)** m/z: M+H$^+$ calculated for $C_{11}H_{17}O_2^+$: 181.1223, found 181.12215.

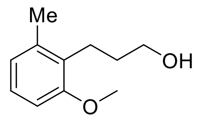

1e: 3-(2-methoxy-6-methylphenyl) propan-1-ol. 1e was synthesized according to the general synthetic method A.

Colorless oil. **^{1}H NMR** (400 MHz, $CDCl_3$) δ 7.09 (t, J = 7.9 Hz, 1H), 6.80 (d, J = 7.6 Hz, 1H), 6.74 (d, J = 8.2 Hz, 1H), 3.83 (s, 3H), 3.57 (t, J = 6.0 Hz, 2H), 2.77 (t, J = 7.2 Hz, 2H), 2.32 (s, 3H), 1.81 – 1.74 (m, 2H). **^{13}C NMR** (101 MHz, $CDCl_3$) δ 157.41, 137.62, 128.16, 126.36, 122.98, 107.99, 61.80, 55.56, 31.55, 21.62, 19.28. **HRMS (ESI)** m/z: M+H$^+$ calculated for $C_{11}H_{17}O_2^+$: 181.1223, found 181.12216.

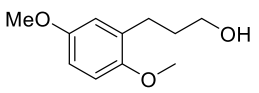

1f: 3-(2,5-dimethoxyphenyl) propan-1-ol[8]. 1f was synthesized according to the general synthetic method A.

Colorless oil. **^{1}H NMR** (400 MHz, $CDCl_3$) δ 6.81 – 6.67 (m, 3H), 3.80 (s, 3H), 3.76 (s, 3H), 3.59 (t, J = 6.2 Hz, 2H), 2.70 (t, J = 7.3 Hz, 2H), 1.84 (p, J = 6.5 Hz, 2H). **^{13}C NMR** (101 MHz, $CDCl_3$) δ 153.57, 151.60, 131.19, 116.32, 111.24, 111.01, 61.65, 55.93, 55.53, 32.80, 25.98. **HRMS (ESI)** m/z: M+H$^+$ calculated for $C_{11}H_{17}O_3^+$: 197.1172, found 197.1171.

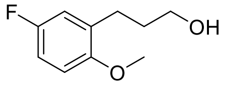

1g: 3-(5-fluoro-2-methoxyphenyl) propan-1-ol. 1g was synthesized according to the general synthetic method A.

Colorless oil. **^{1}H NMR** (400 MHz, $CDCl_3$) δ 6.88 – 6.84 (m, 2H), 6.78 – 6.75 (m, 1H), 3.81 (s, 3H), 3.60 (t, J = 6.2 Hz, 2H), 2.70 (t, J = 7.4 Hz, 2H), 1.88 – 1.81 (m, 2H). **^{13}C NMR** (151 MHz, $CDCl_3$) δ 157.00 (d, $^1J_{CF}$ = 238.58 Hz), 153.45 (d, $^4J_{CF}$ = 3.02 Hz), 131.81 (d, $^3J_{CF}$= 7.55 Hz), 116.61 (d, $^2J_{CF}$ = 22.65 Hz), 112.68 (d, $^2J_{CF}$= 24.16 Hz), 111.04 (d, ^3J = 7.55 Hz), 61.70, 55.89, 32.54,

25.89. **^{19}F NMR** (565 MHz, $CDCl_3$) δ -75.65. **HRMS (ESI)** m/z: $M+H^+$ calculated for $C_{10}H_{14}FO_2^+$: 185.0972, found 185.0971.

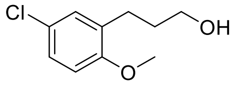

1h: 3-(5-chloro-2-methoxyphenyl) propan-1-ol. 1h was synthesized according to the general synthetic method A.

Colorless oil. **^{1}H NMR** (400 MHz, $CDCl_3$) δ 7.16 – 7.03 (m, 2H), 6.76 (d, J = 8.4 Hz, 1H), 3.81 (s, 3H), 3.60 (t, J = 6.3 Hz, 2H), 2.68 (t, J = 7.4 Hz, 2H), 1.79 – 1.86 (m, 2H). **^{13}C NMR** (151 MHz, $CDCl_3$) δ 155.99, 131.91, 129.77, 126.67, 125.32, 111.40, 61.77, 55.60, 32.52, 25.87. **HRMS (ESI)** m/z: $M+H^+$ calculated for $C_{10}H_{14}ClO_2^+$: 201.0677, found 201.0675.

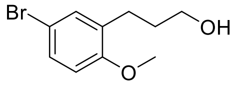

1i: 3-(5-bromo-2-methoxyphenyl) propan-1-ol. 1i was synthesized according to the general synthetic method B.

Colorless oil. **^{1}H NMR** (400 MHz, $CDCl_3$) δ 7.31 – 7.27 (m, 2H), 6.74 (d, J = 8.5 Hz, 1H), 3.83 (s, 3H), 3.63 (t, J = 6.3 Hz, 2H), 2.70 (t, J = 7.4 Hz, 2H), 1.89 – 1.81 (m, 2H). **^{13}C NMR** (151 MHz, $CDCl_3$) δ 156.50, 132.62, 132.39, 129.69, 112.78, 111.93, 61.79, 55.55, 32.54, 25.80. **HRMS (ESI)** m/z: $M+H^+$ calculated for $C_{10}H_{14}BrO_2^+$: 245.0172, found 245.0170.

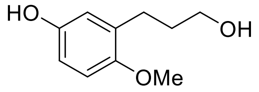

1j: 3-(3-Hydroxypropyl)-4-methoxyphenol. 1j was synthesized according to the general synthetic method A.

Brown solid. **^{1}H NMR** (600 MHz, $CDCl_3$) δ 6.73 (d, J = 8.3 Hz, 1H), 6.67 – 6.62 (m, 2H), 3.78 (s, 3H), 3.60 (t, J = 6.2 Hz, 2H), 2.67 (t, J = 7.2 Hz, 2H), 1.83 (p, J = 6.6 Hz, 2H). **^{13}C NMR** (151 MHz, $CDCl_3$) δ 156.50, 150.42, 132.28, 119.57, 118.76, 115.90, 61.79, 55.55, 32.54, 25.80. **HRMS (ESI)** m/z: $M+H^+$ calculated for $C_{10}H_{15}O_3^+$: 163.1016, found 163.1018.

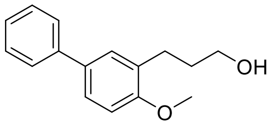

1k: 3-(4-methoxy-[1,1'-biphenyl]-3-yl) propan-1-ol. 1k was synthesized according to the general synthetic method B.

White solid. **^{1}H NMR** (400 MHz, $CDCl_3$) δ 7.58 (m, 2H), 7.47 – 7.39 (m, 4H), 7.33 (t, J = 8.0 Hz, 1H), 6.94 (d, J = 8.2 Hz, 1H), 3.89 (s, 3H), 3.66 (t, J = 6.3 Hz, 2H), 2.81 (t, J = 7.4 Hz, 2H), 1.92 (dd, J = 7.9, 6.5 Hz, 2H). **^{13}C NMR** (101 MHz, $CDCl_3$) δ 156.92, 140.78, 133.59, 130.24, 128.81, 128.59, 126.63, 126.52, 125.61, 110.51, 61.85, 55.39, 32.82, 26.10. **HRMS (ESI)** m/z: $M+H^+$ calculated for $C_{16}H_{19}O_2^+$: 243.1380, found 243.1378.

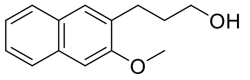

1l: 3-(3-methoxynaphthalen-2-yl) propan-1-ol. 1l was synthesized according to the general synthetic method A.

Colorless oil. **^{1}H NMR** (600 MHz, $CDCl_3$) δ 7.75 – 7.67 (m, 2H), 7.59 (s, 1H), 7.42 – 7.37 (m, 1H), 7.32 (t, J = 7.6 Hz, 1H), 7.11 (s, 1H), 3.95 (s, 3H), 3.67 (t, J = 6.3 Hz, 2H), 2.88 (t, J = 7.4 Hz, 2H), 1.95 (p, J = 6.7 Hz, 2H). **^{13}C NMR** (151 MHz, $CDCl_3$) δ 156.35, 133.34, 131.72, 128.83, 128.40, 126.97, 126.30, 125.57, 123.61, 104.88, 62.09, 55.29, 32.85, 26.54. **HRMS (ESI)** m/z: $M+H^+$ calculated for $C_{14}H_{17}O_2^+$: 217.1223, found 217.1221.

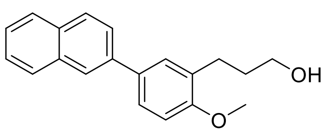

1m: 3-(2-methoxy-5-(naphthalen-2-yl)phenyl)propan-1-ol. 1m was synthesized according to the general synthetic method B.

White solid. **^{1}H NMR** (400 MHz, $CDCl_3$) δ 8.02 (s, 1H), 7.92 – 7.87 (m, 3H), 7.75 (dd, J = 8.5, 1.8 Hz, 1H), 7.57 (dd, J = 6.4, 2.5 Hz, 2H),

7.54 – 7.45 (m, 2H), 7.03 – 6.92 (m, 1H), 3.90 (s, 3H), 3.69 (t, *J* = 6.2 Hz, 2H), 2.85 (t, *J* = 7.4 Hz, 2H), 1.99 – 1.91 (m, 2H). **^{13}C NMR** (101 MHz, $CDCl_3$) δ 157.03, 138.11, 133.67, 133.42, 132.21, 130.39, 129.04, 128.22, 127.94, 127.52, 126.12, 125.93, 125.52, 125.36, 124.89, 110.61, 61.91, 55.43, 32.88, 26.17. **HRMS (ESI)** m/z: M+H$^+$ calculated for $C_{20}H_{21}O_2^+$: 293.1536, found 293.1535.

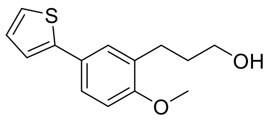

1n: 3-(2-methoxy-5-(thiophen-2-yl)phenyl)propan-1-ol. 1n was synthesized according to the general synthetic method B.

Colorless oil. **^{1}H NMR** (600 MHz, $CDCl_3$) δ 7.43 (dd, *J* = 8.4, 2.4 Hz, 1H), 7.40 (d, *J* = 2.4 Hz, 1H), 7.23 – 7.18 (m, 2H), 7.06 – 7.04 (m, 1H), 6.86 (d, *J* = 8.4 Hz, 1H), 3.86 (s, 3H), 3.64 (t, *J* = 6.2 Hz, 2H), 2.75 (t, *J* = 7.4 Hz, 2H), 1.92 – 1.85 (m, 2H). **^{13}C NMR** (101 MHz, $CDCl_3$) δ 157.08, 144.38, 130.47, 127.96, 127.87, 127.24, 124.84, 123.79, 122.05, 110.62, 61.95, 55.54, 32.87, 26.02. **HRMS (ESI)** m/z: M+H$^+$ calculated for $C_{14}H_{17}O_2S^+$: 249.0944, found 249.0942.

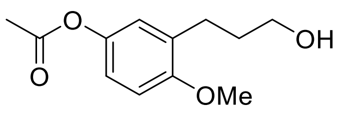

1o: 3-(3-hydroxypropyl)-4-methoxyphenyl acetate. 1o was synthesized according to the general synthetic method A.

Colorless oil. **^{1}H NMR** (600 MHz, $CDCl_3$) δ 6.92 – 6.86 (m, 2H), 6.82 (d, *J* = 8.7 Hz, 1H), 3.82 (s, 3H), 3.61 (t, *J* = 6.2 Hz, 2H), 2.70 (t, *J* = 7.4 Hz, 2H), 2.27 (s, 3H), 1.84 (t, *J* = 7.0 Hz, 2H). **^{13}C NMR** (101 MHz, $CDCl_3$) δ 169.94, 155.09, 144.05, 131.18, 123.02, 119.56, 110.71, 61.86, 55.75, 32.54, 25.92, 21.06. **HRMS (ESI)** m/z: M+H$^+$ calculated for $C_{12}H_{17}O_4^+$: 225.1121, found 225.1123.

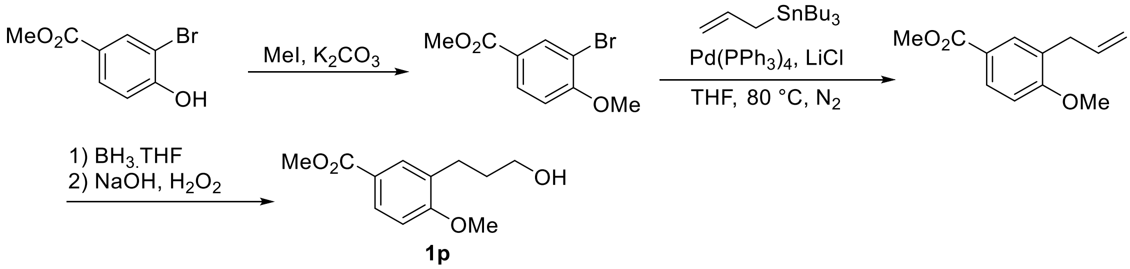

Allylation of the Aromatic Halides: To a solution of methyl 3-bromobenzoate (2.387 g, 9.78 mmol) and LiCl (3.060g, 70.42 mmol) in 20 mL of dry THF under nitrogen were added $Pd(PPh_3)_4$ (0.226 g, 0.20 mmol) and allyltributyltin (3.34 mL, 10.76 mmol). The mixture was degassed with N_2 for 5 min and heated at 80 °C for 36 h. After cooling, it was partitioned between EA and brine. The organic phase was washed with 10% aqueous NaOH solution. The ether layer was separated, washed with water and brine and dried ($MgSO_4$). The solvent was removed and the residue was purified by chromatography (5% EtOAc in hexane) to give 0.806 g (40%) of allylation product.

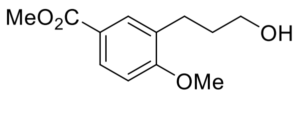

1p: Methyl 3-(3-hydroxypropyl)-4-methoxybenzoate.

Colorless oil. **^{1}H NMR** (600 MHz, $CDCl_3$) δ 7.90 (dd, *J* = 8.6, 2.2 Hz, 1H), 7.84 (d, *J* = 2.2 Hz, 1H), 6.86 (d, *J* = 8.5 Hz, 1H), 3.88 (s, 3H), 3.87 (s, 3H), 3.62 (t, *J* = 6.3 Hz, 2H), 2.73 (t, *J* = 7.5 Hz, 2H), 1.89 – 1.83 (m, 2H). **^{13}C NMR** (101 MHz, $CDCl_3$) δ 167.01, 161.16, 131.39, 130.03, 129.56, 122.29, 109.63, 61.99, 55.52, 51.80, 32.50, 25.97. **HRMS (ESI)** m/z: M+H$^+$ calculated for $C_{12}H_{17}O_4^+$: 225.1121, found 225.1120.

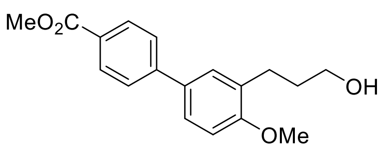

1q: Methyl 3'-(3-hydroxypropyl)-4'-methoxy-[1,1'-biphenyl]-4-carboxylate. 1q was synthesized according to the general synthetic method B.

White solid. **^{1}H NMR** (600 MHz, $CDCl_3$) δ 8.07 (d, *J* = 8.5 Hz, 2H), 7.62 (d, *J* = 8.5 Hz, 2H), 7.47 (dd, *J* = 8.4, 2.4 Hz, 1H), 7.43 (m, 1H), 6.95 (d, *J* = 8.5 Hz, 1H), 3.93 (s, 3H), 3.89 (s, 3H), 3.65 (t, *J* = 6.2 Hz, 2H), 2.79 (t, *J* = 7.4 Hz, 2H), 1.90 (dt, *J* = 13.1, 6.4 Hz, 2H). **^{13}C NMR** (101 MHz, $CDCl_3$) δ 167.08, 157.72, 145.29, 132.37, 130.61, 130.06, 129.02, 128.19, 126.48, 126.04, 110.67, 62.00, 55.57, 52.06, 32.92, 26.17. **HRMS (ESI)** m/z: M+H$^+$ calculated for $C_{18}H_{21}O_4^+$: 301.1434, found 301.1436.

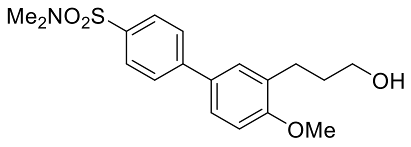

1r: 3'-(3-Hydroxypropyl)-4'-methoxy-N,N-dimethyl-[1,1'-biphenyl]-4-sulfonamide. 1r was synthesized according to the general synthetic method B.

Yellow solid**.** **^{1}H NMR** (600 MHz, $CDCl_3$) δ 7.80 (d, *J* = 8.3 Hz, 2H), 7.70 (d, *J* = 8.4 Hz, 2H), 7.46 (dd, *J* = 8.4, 2.4 Hz, 1H), 7.42 (d, *J* = 2.4 Hz, 1H), 6.96 (d, *J* = 8.4 Hz, 1H), 3.90 (s, 3H), 3.65 (t, *J* = 6.2 Hz, 2H), 2.80 (t, *J* = 7.4 Hz, 2H), 2.74 (s, 6H), 1.90 (dt, *J* = 13.0, 6.6 Hz, 2H). **^{13}C NMR** (101 MHz, $CDCl_3$) δ 157.97, 145.27, 133.23, 131.57, 130.82, 129.04, 128.22, 127.02, 126.10, 110.75, 61.99, 55.56, 37.97, 32.83, 26.22. **HRMS (ESI)** m/z: M+H$^+$ calculated for $C_{18}H_{24}O_4S^+$: 350.1421, found 350.1422.

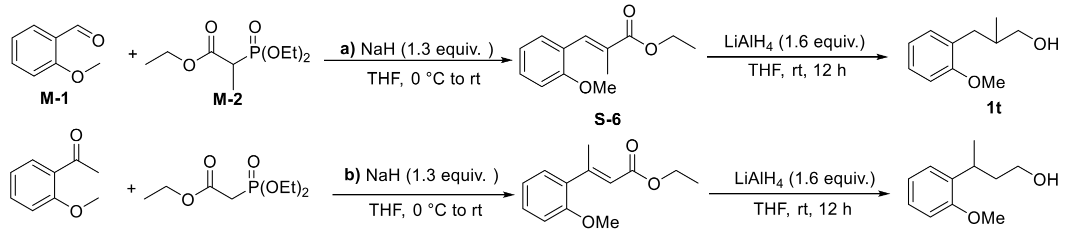

Supplementary Figure 5. Synthesis of **1t** and **1s**

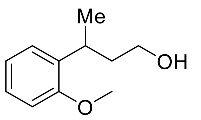

1s: 4-methylchromane.

To an ice-cold suspension of NaH (13.5 mmol, 1.35 equiv.) in dry THF (30 mL) was added triethyl phosphonoacetate (**M-2**) (12.5 mmol, 1.25 equiv.) under N_2 atmosphere. After stirring at room temperature for 30 min, **M-1** was added and the reaction was refluxed for 24 hours. The reaction mixture was cooled to 0 °C and quenched with saturated aqueous NH_4Cl (1.5 mL) and the organic materials were extracted with ethyl acetate (3×10 mL). The combined organic phase was washed with brine, dried over anhydrous $MgSO_4$, filtered and concentrated under reduced pressure. The resulting crude material was purified by flash column chromatography (SiO_2, PE/EA = 20/ 1) to give α, β-unsaturated ester **S-6** in quantitative yield as a colorless oil. The procedure a was according to the literature report[9]. And the next step is same as the step b of method A to get the product **1s** as a colorless oil. **^{1}H NMR** (600 MHz, $CDCl_3$) δ 7.24 – 7.13 (m, 2H), 6.96 (t, *J* = 7.5 Hz, 1H), 6.88 (d, *J* = 8.1 Hz, 1H), 3.84 (s, 3H), 3.59 – 3.51 (m, 1H), 3.45 – 3.36 (m, 2H), 1.93 – 1.89 (m, 1H), 1.72 – 1.65 (m, 1H), 1.27 (d, *J* = 7.0 Hz, 3H). **^{13}C NMR** (101 MHz, $CDCl_3$) δ 156.71, 134.48, 126.84, 126.77, 121.05, 110.47, 61.03, 55.48, 40.72, 27.62, 20.97. **HRMS (ESI)** m/z: M+H$^+$ calculated for $C_{11}H_{17}O_2^+$: 181.1223, found 181.12218.

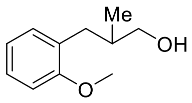

1t: 3-methylchromane.

Colorless oil. **^{1}H NMR** (400 MHz, $CDCl_3$) δ 7.20 (td, J = 7.8, 1.8 Hz, 1H), 7.12 (dd, J = 7.4, 1.8 Hz, 1H), 6.93 – 6.85 (m, 2H), 3.84 (s, 3H), 3.40 – 3.38 (m, 2H), 2.75 – 2.70 (m, 1H), 2.56 – 2.52 (m, 1H), 1.99 – 1.91 (m, 1H), 0.96 (d, J = 6.8 Hz, 3H). **^{13}C NMR** (101 MHz, $CDCl_3$) δ 157.37, 131.12, 128.66, 127.20, 120.56, 110.34, 66.84, 55.36, 36.62, 33.05, 16.88. **HRMS (ESI)** m/z: M+H$^+$ calculated for $C_{11}H_{17}O_2^+$: 181.1223, found 181.12216

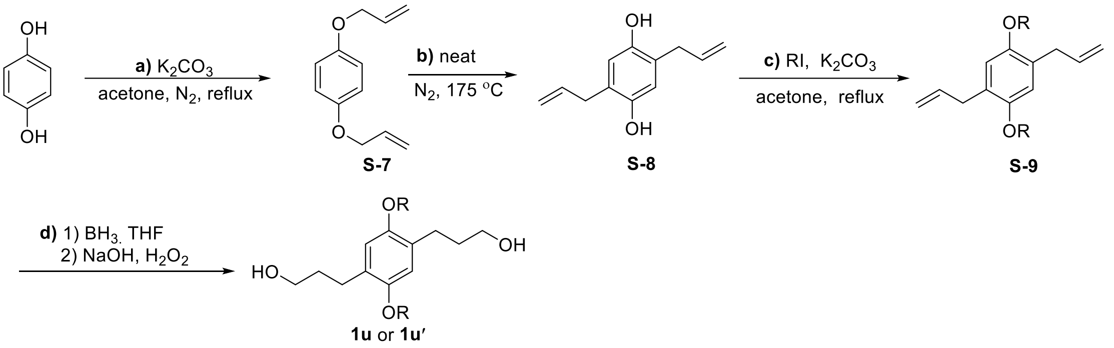

Supplementary Figure 6. Synthesis of substrates **1u** and **1u'**

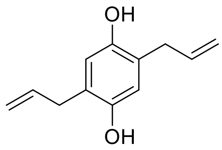

b) S-7 (5.003 g, 26.3 mmol) was added to a 10 mL round bottom flask and filled with nitrogen. After stirring at 175 °C for 12 hours, the reaction was cooled to room temperature and was purified by silica gel column chromatography to give the corresponding product **S-8** (1.254g, 25% yield) as a brown red solid.

d) The alkene **S-9** (0.391g, 1.79 mmol) and dry THF (5 mL) were added to a dry, nitrogen-flushed, 25-mL three-necked flask. The flask was cooled to 0 °C (ice bath), and hydroboration was initiated by dropwise addition of a 1.0 M solution of BH_3-THF (8.95 mmol, 9 mL). The mixture was stirred at room temperature until complete conversion of substrate monitored by TLC. The reaction mixture was cooled in an ice water bath and then added NaOH (1.8 mL, 3M) dropwise, followed by addition of 30% H_2O_2 (10.7 mmol, 1.1 mL). The reaction contents were allowed to warm to room temperature and then stirred for 3 hours. The reaction mixture was extracted with ethyl acetate (3×6 mL) and the combined organic extracts was washed with brine, dried over anhydrous Na_2SO_4, and concentrated under reduced pressure to afford **1u** (0.272 g, 60% yield) as a white solid. The procedure d was according to the literature report[10].

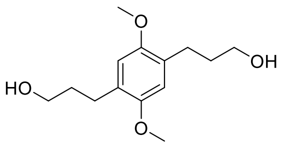

1u: 3,3'-(2,5-dimethoxy-1,4-phenylene) bis(propan-1-ol).

White solid. **^{1}H NMR** (400 MHz, $CDCl_3$) δ 6.70 (s, 2H), 3.79 (s, 6H), 3.59 (t, J = 6.0 Hz, 4H), 2.77 (t, J = 7.4 Hz, 4H), 1.81 – 1.74 (m, 4H). **^{13}C NMR** (101 MHz, $CDCl_3$) δ 152.00, 129.87, 108.16, 61.98, 55.85, 32.75, 21.99. **HRMS (ESI)** m/z: M+H$^+$ calculated for $C_{14}H_{23}O_4^+$: 255.1591, found 255.1589.

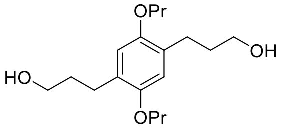

1u': 3,3'-(2,5-dipropoxy-1,4-phenylene) bis(propan-1-ol).

White solid. **^{1}H NMR** (400 MHz, $CDCl_3$) δ 6.67 (s, 2H), 3.88 (t, J = 6.5 Hz, 4H), 3.59 (t, J = 5.9 Hz, 4H), 2.79 (t, J = 7.3 Hz, 4H), 1.85 – 1.76 (m, 8H), 1.05 (t, J = 7.4 Hz, 6H). **^{13}C NMR** (151 MHz, $CDCl_3$) δ 151.40, 129.87, 109.38, 70.34, 61.90, 32.82, 22.80, 21.92, 10.69. **HRMS (ESI)** m/z: M+H$^+$ calculated for $C_{18}H_{31}O_4^+$: 311.2217, found 311.2218.

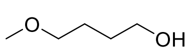

1v: 4-methoxybutan-1-ol[11]. 1v was synthesized according to the general synthetic method C (the step b).

Colorless oil. **^{1}H NMR** (400 MHz, $CDCl_3$) δ 3.63 (t, *J* = 5.7 Hz, 2H), 3.41 (t, *J* = 5.6 Hz, 2H), 3.34 (s, 3H), 1.66 (p, *J* = 6.1 Hz, 4H).

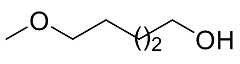

1w: 5-methoxypentan-1-ol[11]. 1w was synthesized according to the general synthetic method C (the step b).

Colorless oil. **^{1}H NMR** (600 MHz, $CDCl_3$) δ 3.67 – 3.58 (m, 2H), 3.40 – 3.35 (m, 2H), 3.31 (s, 3H), 1.65 – 1.52 (m, 4H), 1.43 – 1.38 (M, 2H).

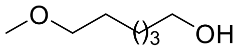

1x: 6-methoxyhexan-1-ol[12]. 1x was synthesized according to the general synthetic method C (the step b).

Colorless oil. **^{1}H NMR** (400 MHz, $CDCl_3$) δ 3.63 (t, *J* = 6.6 Hz, 2H), 3.36 (t, *J* = 6.6 Hz, 2H), 3.32 (s, 3H), 1.60 – 1.53 (m, 4H), 1.40 – 1.34 (m, 4H).

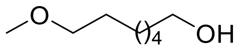

1y: 4-methoxybutan-1-ol[12]. 1y was synthesized according to the general synthetic method C (the step b).

Colorless oil. **^{1}H NMR** (400 MHz, $CDCl_3$) δ 3.61 (t, *J* = 6.6 Hz, 2H), 3.35 (t, *J* = 6.6 Hz, 2H), 3.31 (s, 3H), 1.77 (s, br, 1H), 1.59 – 1.50 (m, 4H), 1.37 – 1.27 (m, 6H).

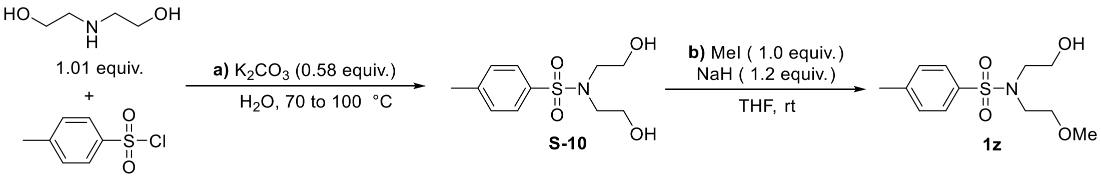

Supplementary Figure 7. Synthesis of **1z**

a) To a three-necked flask equipped with a thermometer and a reflux condenser were successively added H_2O (30 mL), K_2CO_3 (1.163 g, 11.6 mmol) and diethanolamine (2.123 g, 20.2 mmol). The mixture was heated to 70 °C and *p*-toluenesulfonyl chloride (3.813 g, 20 mmol) added portion wise over 10 minutes. The mixture was heated to 100 °C for 1 hour after which all the *p*-toluenesulfonyl chloride had been dissolved. The reaction mixture was then slowly cooled to room temperature and subsequently cooled to 0 °C (ice bath), after which a small amount of white solid appeared. The solid was removed by filtration and the filtrate was placed in the freezer overnight after which a white precipitate was obtained. The solid was collected by filtration, washed repeatedly with ice water, and dried under reduced pressure over P_2O_5 to give the product **S-10** (3.121 g, 60%) as a white solid. The procedure a was according to the literature report[13]. And then product **1z** was made from **S-10** by mono-methylation (Step b in Supplementary Figure 3).

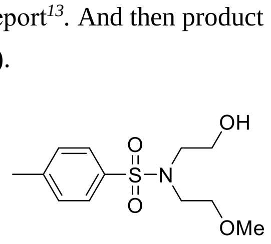

1z: N-(2-hydroxyethyl)-N-(2-methoxyethyl)-4-methylbenzenesulfonamide. Yellow oil. **^{1}H NMR** (400 MHz, $CDCl_3$) δ 7.68 (d, *J* = 8.3 Hz, 2H), 7.33 – 7.29 (m, 2H), 3.74 (t, *J* = 4.8 Hz, 2H), 3.65 (t, *J* = 5.0 Hz, 2H), 3.38 (s, 3H), 3.30 (t, *J* = 5.0 Hz, 2H), 3.24 – 3.22 (m, 2H), 2.42 (s, 3H). **^{13}C NMR** (101 MHz, $CDCl_3$) δ 143.46, 135.47, 129.63, 127.11, 72.36, 61.75, 58.71, 53.15, 49.81, 21.33. **HRMS (ESI)** m/z: M+H$^+$ calculated for $C_{12}H_{20}NO_4S^+$: 274.1108, found 274.1106.

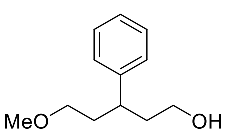

1bb: 5-methoxy-3-phenylpentan-1-ol. 1bb was synthesized according to the general synthetic method C.

Colorless oil. **^{1}H NMR** (400 MHz, CDCl3) δ 7.32 – 7.28 (m, 2H), 7.22 – 7.17 (m, 3H), 3.57 – 3.44 (m, 2H), 3.27 – 3.22 (m, 1H), 3.25 (s, 3H), 3.19 – 3.12 (m, 1H), 2.91 – 2.84 (m, 1H), 2.01 – 1.78 (m, 4H). **^{13}C NMR** (101 MHz, CDCl3) δ 144.42, 128.47, 127.55, 126.28, 70.57, 60.85, 58.48, 39.42, 38.85, 36.46. **HRMS (ESI)** m/z: M+H$^+$ calculated for $C_{12}H_{19}O_2^+$: 195.1380, found 195.1378.

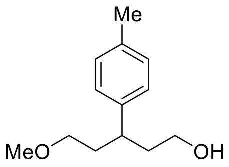

1cc: 5-methoxy-3-(p-tolyl)pentan-1-ol. 1cc was synthesized according to the general synthetic method C.

Colorless oil. **^{1}H NMR** (400 MHz, $CDCl_3$) δ 7.14 – 7.03 (m, 4H), 3.57 – 3.43 (m, 2H), 3.32 – 3.27 (m, 1H), 3.25 (s, 3H), 3.20 – 3.12 (m, 1H), 2.87 – 2.79 (m, 1H), 2.32 (s, 3H), 1.98 – 1.76 (m, 4H). **^{13}C NMR** (101 MHz, $CDCl_3$) δ 141.27, 135.63, 129.10, 127.36, 70.59, 60.74, 58.40, 39.44, 38.35, 36.42, 20.91. **HRMS (ESI)** m/z: M+H$^+$ calculated for $C_{13}H_{21}O_2^+$: 209.1536, found 209.1534.

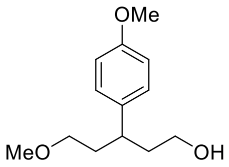

1dd: 5-methoxy-3-(4-methoxyphenyl)pentan-1-ol. 1dd was synthesized according to the general synthetic method C.

Colorless oil. **^{1}H NMR** (400 MHz, $CDCl_3$) δ 7.09 (d, J = 8.6 Hz, 2H), 6.84 (d, J = 8.7 Hz, 2H), 3.79 (s, 3H), 3.56 – 3.44 (m, 2H), 3.26 – 3.21 (m, 1H),3.25 (s, 4H), 3.18 – 3.13 (m, 1H), 2.81 (s, 1H), 1.95 – 1.74 (m, 4H). **^{13}C NMR** (101 MHz, $CDCl_3$) δ 157.99, 136.34, 128.41, 113.86, 70.60, 60.96, 58.49, 55.17, 39.58, 38.04, 36.65. **HRMS (ESI)** m/z: M+H$^+$ calculated for $C_{13}H_{21}O_3^+$: 225.1485, found 225.1484.

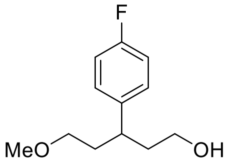

1ee: 3-(4-fluorophenyl)-5-methoxypentan-1-ol. 1ee was synthesized according to the general synthetic method C.

Colorless oil. **^{1}H NMR** (400 MHz, $CDCl_3$) δ 7.15 – 7.11 (m, 2H), 7.11 – 6.86 (m, 2H), 3.59 – 3.49 (m, 1H), 3.49 – 3.40 (m, 1H), 3.26 – 3.21 (m, 1H), 3.25 (s, 3H), 3.18 – 3.08 (m, 1H), 2.96 – 2.77 (m, 1H), 2.02 – 1.87 (m, 2H), 1.85 – 1.71 (m, 2H). **^{13}C NMR** (101 MHz, CDCl3) δ 161.33 (d, $^1J_{CF}$ = 245.43 Hz), 140.00 (d, $^4J_{CF}$ = 3.03 Hz), 128.86 (d, $^3J_{CF}$ = 8.08 Hz), 115.18 (d, $^2J_{CF}$ = 21.21 Hz), 70.36, 60.54, 58.46, 39.44, 37.95, 36.48. **^{19}F NMR** (565 MHz, $CDCl_3$) δ -116.94. **HRMS (ESI)** m/z: M+H$^+$ calculated for $C_{12}H_{18}FO_2^+$: 213.1285, found 213.1284.

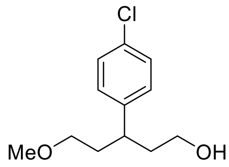

1ff: 3-(4-chlorophenyl)-5-methoxypentan-1-ol. 1ff was synthesized according to the general synthetic method C.

Colorless oil. **^{1}H NMR** (400 MHz, $CDCl_3$) δ 7.27 (d, J = 7.9 Hz, 2H), 7.12 (d, J = 8.2 Hz, 2H), 3.63 – 3.36 (m, 2H), 3.27 – 3.22 (m,1H), 3.25 (s, 3H), 3.18 – 3.08 (m, 1H), 2.95 – 2.82 (m, 1H), 2.02 – 1.87 (m, 2H), 1.84 – 1.70 (m, 2H). **^{13}C NMR** (101 MHz, $CDCl_3$) δ 142.92, 131.87, 128.94, 128.60, 70.32, 60.60, 58.52, 39.29, 38.17, 36.38. **HRMS (ESI)** m/z: M+H$^+$ calculated for $C_{12}H_{18}ClO_2^+$: 229.0990, found 229.0988.

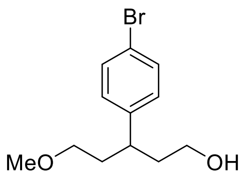

1gg: 3-(4-Bromophenyl)-5-methoxypentan-1-ol. 1gg was synthesized according to the general synthetic method C.

Colorless oil. **^{1}H NMR** (600 MHz, $CDCl_3$) δ 7.42 (d, J = 8.4 Hz, 2H), 7.06 (d, J = 8.4 Hz, 2H), 3.57 – 3.50 (m, 1H), 3.48 – 3.42 (m, 1H), 3.24 – 3.22 (m, 1H), 3.25 (s, 3H), 3.16 – 3.09 (m, 1H), 2.93 – 2.83 (m, 1H), 1.99 – 1.89 (m, 2H), 1.83 – 1.71 (m, 2H). **^{13}C NMR** (101 MHz, $CDCl_3$) δ 143.47, 131.58, 129.37, 119.94, 70.32, 60.70, 58.55, 39.25, 38.29, 36.40. **HRMS (ESI)** m/z: M+H$^+$ calculated for $C_{12}H_{18}BrO_2^+$: 273.0485, found 273.0486.

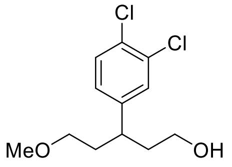

1hh: 3-(3,4-dichlorophenyl)-5-methoxypentan-1-ol. 1hh was synthesized according to the general synthetic method C.

Colorless oil. **^{1}H NMR** (400 MHz, $CDCl_3$) δ 7.36 (d, J = 8.2 Hz, 1H), 7.27 – 7.25 (m, 1H), 7.02 (dd, J = 8.3, 2.1 Hz, 1H), 3.59 – 3.50 (m, 1H), 3.48 – 3.40 (m, 1H), 3.30 – 3.19 (m, 1H), 3.28 (s, 3H), 3.17 – 3.08 (m, 1H), 2.96 – 2.85 (m, 1H), 2.00 – 1.86 (m, 2H), 1.83 – 1.71 (m, 2H). **^{13}C NMR** (101 MHz, $CDCl_3$) δ 144.91, 132.39, 130.38, 130.07, 129.53, 127.12, 70.10, 60.31, 58.53, 39.04, 37.97, 36.18. **HRMS (ESI)** m/z: M+H$^+$ calculated for $C_{12}H_{17}Cl_2O_2^+$: 263.0600, found 263.0958.

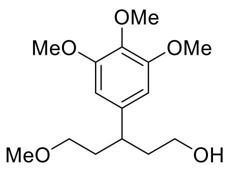

1ii: 5-methoxy-3-(3,4,5-trimethoxyphenyl)pentan-1-ol. 1ii was synthesized according to the general synthetic method C.

White solid. **^{1}H NMR** (400 MHz, $CDCl_3$) δ 6.37 (s, 2H), 3.82 (s, 6H), 3.81 (s, 3H), 3.59 – 3.44 (m, 2H), 3.48 – 3.24 (m, 1H), 3.26 (s, 3H), 3.21 – 3.08 (m, 1H), 2.81 – 2.76 (m, 1H), 1.98 – 1.75 (m, 4H). **^{13}C NMR** (101 MHz, $CDCl_3$) δ 153.14, 140.25, 136.29, 104.29, 70.51, 60.91, 60.77, 58.53, 56.02, 39.35, 39.27, 36.53. **HRMS (ESI)** m/z: M+H$^+$ calculated for $C_{15}H_{25}O_5^+$: 285.1697, found 285.1694.

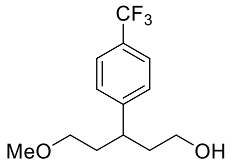

1jj: 5-methoxy-3-(4-(trifluoromethyl)phenyl)pentan-1-ol. 1jj was synthesized according to the general synthetic method C.

Colorless oil. **^{1}H NMR** (400 MHz, $CDCl_3$) δ 7.60 – 7.41 (m, 2H), 7.30 (d, J = 8.1 Hz, 2H), 3.57 – 3.50 (m, 1H), 3.48 – 3.40 (m, 1H), 3.28 – 3.22 (m, 1H), 3.25 (s, 3H), 3.15– 3.10 (m, 1H), 3.06 – 2.92 (m, 1H), 2.04 – 1.77 (m, 4H). ^{13}C NMR (101 MHz, $CDCl_3$) δ 148.75, 128.61 (q, $^2J_{CF}$ = 32.32 Hz), 127.96, 125.41 (q, $^3J_{CF}$ = 4.04 Hz), 124.23 (q, $^1J_{CF}$ = 272.70 Hz), 70.20, 60.39, 58.52, 39.10, 38.57, 38.54, 36.20. **^{19}F NMR** (565 MHz, $CDCl_3$) δ -62.34. **HRMS (ESI)** m/z: M+H$^+$ calculated for $C_{13}H_{18}F_3O_2^+$: 263.1253, found 263.1252.

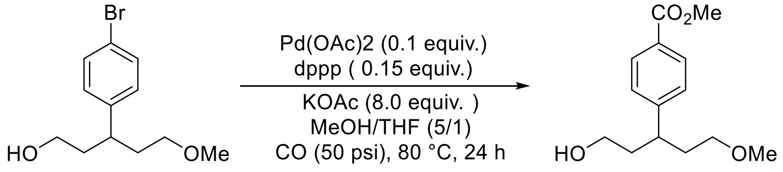

1gg **1kk**

To a solution of **1gg** (680 mg, 2.50 mmol, 1.00 eq) in MeOH (20.00 mL) and THF (10.00 mL) were added KOAc (1.963 g, 20 mmol, 8.00 eq), $Pd(OAc)_2$ (56.13 mg, 0.25 mmol, 0.10 eq),and 3-diphenylphosphanylpropyl(diphenyl)phosphane (154.66 mg,0.15 mmol, 0.15 eq). The suspension was degassed and purged with CO. The mixture was stirred under an atmosphere of CO (50 psi) at 80 °C for 16 h. The crude product was purified by silica gel column chromatography (Petroleum ethenEthyl acetate

20:1 to 3:1) to afford methyl 4-(2- hydroxyethyl) benzoate **1kk** (429.00 mg) as a yellow oil.

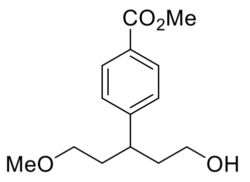

1kk: Methyl 4-(1-hydroxy-5-methoxypentan-3-yl) benzoate.

Colorless oil. ^{1}**H NMR** (600 MHz, $CDCl_3$) δ 7.97 (d, *J* = 8.3 Hz, 2H), 7.25 (d, *J* = 8.2 Hz, 2H), 3.90 (s, 3H), 3.55 – 3.51 (m, 1H), 3.47 – 3.42 (m, 1H), 3.25 – 3.21 (m, 1H), 3.23 (s, 3H), 3.13 – 3.09 (m, 1H), 3.02 – 2.93 (m, 1H), 2.01 – 1.93 (m, 2H), 1.87 – 1.77 (m, 2H). ^{13}**C NMR** (101 MHz, $CDCl_3$) δ 167.04, 150.12, 129.85, 128.36, 127.68, 70.30, 60.66, 58.55, 52.00, 39.16, 38.86, 36.30. **HRMS (ESI)** m/z: M+H$^+$ calculated for $C_{14}H_{21}O_4^+$: 253.1434, found 253.1435.

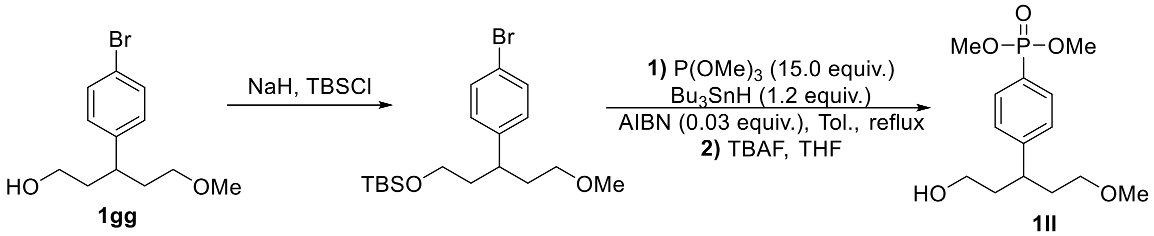

Heat a mixture of TBS-protected **1gg** (193.07 mg, 0.5 mmol) and trimethyl phosphit (930.6 mg, 7.5 mmol) in toluene (4 mL) under reflux for 6 hours. Add a solution of tri-n-butyltin hydride (174.64 mg, 0.6 mmol) and azo-bis(isobutyronitrile) (2.46 mg, 0.015 mmol) in toluene (3 mL) to the mixture. Heat the reaction mixture under reflux for 12 hours. Remove the solvent under reduced pressure. Then add THF (3 mL), TBAF (2 mL, 1 M/THF) to the residue and stirred at room temperature until the TBS-group completely removed. Purify the product **1ll** by column chromatography (silica gel, ethyl acetate).

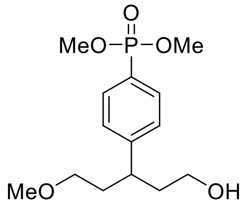

1ll: Dimethyl (4-(1-hydroxy-5-methoxypentan-3-yl) phenyl) phosphonate.

White solid. ^{1}**H NMR** (600 MHz, $CDCl_3$) δ 7.78 – 7.60 (m, 2H), 7.33 – 7.27 (m, 2H), 3.77 (s, 3H), 3.75 (s, 3H), 3.56 – 3.49 (m, 1H), 3.47 – 3.40 (m, 1H), 3.25 – 3.22 (m, 1H), 3.24 (s, 3H), 3.14 – 3.10 (m, 1H), 3.00 – 2.95 (m, 1H), 2.03 – 1.92 (m, 2H), 1.87 – 1.78 (m, 2H). ^{13}**C NMR** (101 MHz, $CDCl_3$) δ 132.12, 132.02, 128.02, 127.87, 70.26, 60.47, 58.51, 52.69, 39.09, 38.82, 36.21. ^{31}**P NMR** (243 MHz, $CDCl_3$) δ 22.03. **HRMS (ESI)** m/z: M+H$^+$ calculated for $C_{14}H_{24}O_5P^+$: 303.1356, found 303.1357.

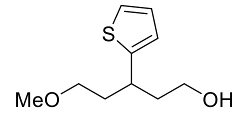

1mm: 5-methoxy-3-(thiophen-2-yl) pentan-1-ol. 1mm was synthesized according to the general synthetic method C.

Colorless oil. ^{1}**H NMR** (400 MHz, $CDCl_3$) δ 7.16 (d, *J* = 6.7 Hz, 1H), 6.99 – 6.86 (m, 1H), 6.88 – 6.72 (m, 1H), 3.67 – 3.51 (m, 2H), 3.36 – 3.21 (m, 3H), 3.28 (s, 3H), 2.04 – 1.90 (m, 2H), 1.89 – 1.78 (m, 2H). ^{13}**C NMR** (101 MHz, $CDCl_3$) δ 148.44, 126.52, 123.06, 70.27, 60.58, 58.54, 40.45, 37.53, 34.30. **HRMS (ESI)** m/z: M+H$^+$ calculated for $C_{10}H_{16}O_2S^+$: 201.0944, found 201.0941.

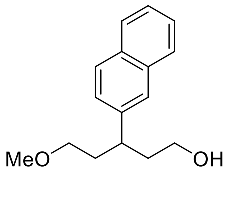

1nn: 5-methoxy-3-(naphthalen-2-yl) pentan-1-ol. 1nn was synthesized according to the general synthetic method C.

Colorless oil. ^{1}**H NMR** (400 MHz, $CDCl_3$) δ 7.82 – 7.78 (m, 3H), 7.62 (s, 1H), 7.53 – 7.37 (m, 2H), 7.34 (d, *J* = 8.6 Hz, 1H), 3.61 – 3.43 (m, 2H), 3.26 – 3.19

(m, 2H), 3.25 (s, 3H), 3.12 – 3.01 (m, 1H), 2.09 – 1.87 (m, 4H). **^{13}C NMR** (101 MHz, $CDCl_3$) δ 141.80, 133.52, 132.36, 128.29, 127.60, 127.52, 126.38, 125.98, 125.57, 125.34, 77.20, 70.60, 60.97, 58.55, 39.37, 39.04, 36.48. **HRMS (ESI)** m/z: M+H$^+$ calculated for $C_{16}H_{21}O_2^+$: 245.1536, found 245.1534.

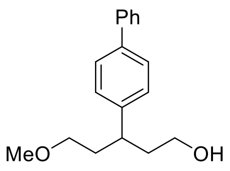

1oo: 3-([1,1'-biphenyl]-4-yl)-5-methoxypentan-1-ol. 1oo was synthesized according to the general synthetic method C.

Colorless oil. **^{1}H NMR** (400 MHz, $CDCl_3$) δ 7.62 – 7.57 (m, 2H), 7.57 – 7.50 (m, 2H), 7.45 – 7.42 (m, 2H), 7.35 – 7.32 (m, 1H), 7.27 – 7.25 (m, 2H), 3.64 – 3.47 (m, 2H), 3.32 – 3.27 (m, 1H), 3.28 (s, 3H), 3.24 – 3.18 (m, 1H), 2.97 – 2.90 (m, 1H), 2.04 – 1.82 (m, 4H). **^{13}C NMR** (101 MHz, $CDCl_3$) δ 143.55, 140.86, 139.17, 128.71, 127.99, 127.18, 127.07, 126.92, 70.59, 60.96, 58.54, 39.42, 38.55, 36.51. **HRMS (ESI)** m/z: M+H$^+$ calculated for $C_{18}H_{23}O_2^+$: 271.1693, found 271.1691.

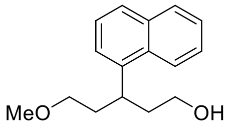

1pp: 5-methoxy-3-(naphthalen-1-yl) pentan-1-ol. 1pp was synthesized according to the general synthetic method C.

Colorless oil. **^{1}H NMR** (400 MHz, $CDCl_3$) δ 8.23 (d, *J* = 8.2 Hz, 1H), 7.90 – 7.80 (m, 1H), 7.73 (d, *J* = 8.0 Hz, 1H), 7.58 – 7.32 (m, 4H), 4.03 – 3.81 (m, 1H), 3.61 – 3.52 (m, 1H), 3.52 – 3.42 (m, 1H), 3.33 – 3.24 (m, 1H), 3.20 (s, 3H), 3.16 – 3.07 (m, 1H), 2.24 – 1.95 (m, 4H). **^{13}C NMR** (151 MHz, $CDCl_3$) δ 141.13, 133.83, 132.43, 128.80, 126.56, 125.79, 125.52, 125.38, 123.10, 70.58, 60.78, 58.46, 39.50, 36.74. **HRMS (ESI)** m/z: M+H$^+$ calculated for $C_{16}H_{21}O_2^+$: 245.1536, found 245.1534.

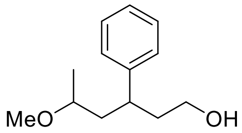

1qq: 5-Methoxy-3-phenylhexan-1-ol. 1qq was synthesized according to the general synthetic method C.

Colorless oil. **^{1}H NMR** (600 MHz, $CDCl_3$) δ7.31 – 7.29 (m, 2H), 7.21 – 7.18 (m, 3H),3.54 – 3.43 (m, 2H), 3.21 (s, 3H), 3.12 – 2.99 (m, 1H), 2.94 – 2.82 (m, 1H), 1.98 – 1.64 (m, 4H), 1.13 – 1.02 (m, 3H). **^{13}C NMR** (101 MHz, $CDCl_3$) δ 144.91, 128.50, 128.43, 127.71, 127.44, 126.17, 74.61, 60.78, 55.91, 43.19, 39.61, 38.70, 19.13. **HRMS (ESI)** m/z: M+H$^+$ calculated for $C_{13}H_{21}O_2^+$: 209.1536, found 209.1537.

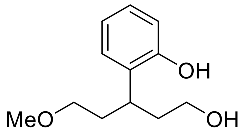

1rr: 2-(1-Hydroxy-5-methoxypentan-3-yl) phenol. 1rr was synthesized according to the general synthetic method C.

Colorless oil. **^{1}H NMR** (600 MHz, $CDCl_3$) δ 7.12 – 7.09 (m, 2H), 6.96 – 6.86 (m, 2H), 3.65 – 3.61 (m, 1H), 3.44 – 3.40 (m, 1H), 3.37 – 3.27 (m, 2H), 3.32 (s, 3H), 3.24 – 3.19 (m, 1H), 2.10 – 1.99 (m, 1H), 2H), 1.84 – 1.71 (m, 2H). **^{13}C NMR** (101 MHz, $CDCl_3$) δ 154.85, 129.51, 127.30, 126.67, 121.08, 116.79, 70.62, 60.58, 58.50, 38.57, 35.64, 29.48. **HRMS (ESI)** m/z: M+H$^+$ calculated for $C_{12}H_{19}O_3^+$: 211.1329, found 211.1330.

3.2 Ring-Closing C–O/C–O Cross Metathesis of Primary aliphatic Alcohols with Ethers

General procedure for the Ring-Closing C-O/C-O Cross Metathesis of Primary aliphatic Alcohols with Ethers catalyzed by Re_2O_7: To a 10 mL Schlenk tube was added Re_2O_7 (2-5 mol%), substrate **1** (1.0 equiv.) and HFIP (0.2 M). The reaction mixture was stirred at 100 °C for 12 hours, then the reaction was quenched by adding a proper amount of Et_3N, and the solvent was then removed under reduced pressure.

The crude mixture was purified by flash column chromatography to afford the target product.

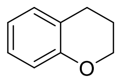

2a: Chromane.

2a was synthesized according to the general synthetic method with **1a** (24.12 mg, 0.145 mmol), Re_2O_7 (1.36 mg, 0.00281 mmol), and HFIP (0.7 mL). The reaction was stirred at 100 °C for 12 hours then quenched with 20 μL Et_3N, concentrated under reduced pressure and purified through flash chromatography (100% petroleum ether) to give the desired product **2a** as a colorless oil (16.52 mg, 85% yield). **^{1}H NMR** (400 MHz, $CDCl_3$) δ 7.16 – 6.97 (m, 2H), 6.90 – 6.69 (m, 2H), 4.24 – 4.13 (m, 2H), 2.79 (t, J = 6.5 Hz, 2H), 2.06 – 1.96 (m, 2H). **^{13}C NMR** (101 MHz, $CDCl_3$) δ 154.87, 129.79, 127.17, 122.20, 120.07, 116.67, 66.40, 24.86, 22.36. **HRMS (ESI)** m/z: M+H$^+$ calculated for $C_9H_{11}O^+$: 135.0804, found 135.0805.

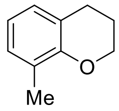

2b: 8-methylchromane.

2b was synthesized according to the general synthetic method with **1b** (20.59 mg, 0.114 mmol), Re_2O_7 (1.12 mg, 0.00231 mmol), and HFIP (0.8 mL). The reaction was stirred at 100 °C for 12 hours then quenched with 20 μL Et_3N, concentrated under reduced pressure and purified through flash chromatography (100% petroleum ether) to give the desired product **2b** as a colorless oil (10.26 mg, 60% yield). **^{1}H NMR** (400 MHz, $CDCl_3$) δ 7.02 – 6.93 (m, 1H), 6.94 – 6.84 (m, 1H), 6.75 (t, J = 7.4 Hz, 1H), 4.27 – 4.18 (m, 2H), 2.80 (t, J = 6.5 Hz, 2H), 2.19 (s, 3H), 2.04 – 1.96 (m, 2H). **^{13}C NMR** (101 MHz, $CDCl_3$) δ 153.04, 128.32, 127.34, 125.81, 121.56, 119.40, 66.48, 25.04, 22.46, 15.98. **HRMS (ESI)** m/z: M+H$^+$ calculated for $C_{10}H_{13}O^+$: 149.0961, found 149.09617.

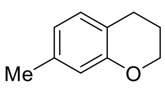

2c: 7-methylchromane.

2c was synthesized according to the general synthetic method with **1c** (40.44 mg, 0.224 mmol), Re_2O_7 (2.11 mg, 0.00435 mmol), and HFIP (1.1 mL). The reaction was stirred at 100 °C for 12 hours then quenched with 20 μL Et_3N, concentrated under reduced pressure and purified through flash chromatography (100% petroleum ether) to give the desired product **2c** as a colorless oil (26.85 mg, 81% yield). **^{1}H NMR** (400 MHz, $CDCl_3$) δ 6.92 (d, J = 7.6 Hz, 1H), 6.80 – 6.44 (m, 2H), 4.23 – 4.04 (m, 2H), 2.75 (t, J = 6.4 Hz, 2H), 2.27 (s, 3H), 2.05 – 1.91 (m, 2H). **^{13}C NMR** (101 MHz, $CDCl_3$) δ 154.64, 137.08, 129.53, 121.01, 119.07, 117.04, 66.39, 24.52, 22.50, 21.02. **HRMS (ESI)** m/z: M+H$^+$ calculated for $C_{10}H_{13}O^+$: 149.0961, found 149.09616.

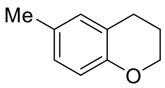

2d: 6-methylchromane.

2d was synthesized according to the general synthetic method with **1d** (41.80 mg, 0.224 mmol), Re_2O_7 (2.21 mg, 0.00456 mmol), and HFIP (1.1 mL). The reaction was stirred at 100 °C for 12 hours then quenched with 20 μL Et_3N, concentrated under reduced pressure and purified through flash chromatography (100% petroleum ether) to give the desired product **2d** as a colorless oil (25.73 mg, 75% yield). **^{1}H NMR** (400 MHz, $CDCl_3$) δ 6.91 – 4.86(m, 2H), 6.71 (d, J = 8.2 Hz, 1H), 4.18 – 4.16 (m, 2H), 2.76 (t, J = 6.5 Hz, 2H), 2.26 (s, 3H), 2.03 – 1.97 (m, 2H). **^{13}C NMR** (101 MHz, $CDCl_3$) δ 152.65, 130.14, 129.22, 127.77, 121.84, 116.39, 66.37, 24.83, 22.48, 20.43. **HRMS (ESI)** m/z: M+H$^+$ calculated for $C_{10}H_{13}O^+$: 149.0961, found 149.09617.

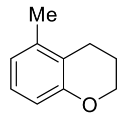

2e: 5-methylchromane.

2e was synthesized according to the general synthetic method with **1e** (34.79 mg, 0.193 mmol), Re_2O_7 (1.92 mg, 0.00396 mmol), and HFIP (1.0 mL). The reaction was stirred at 100 °C for 12 hours then quenched with 20 µL Et_3N, concentrated under reduced pressure and purified through flash chromatography (100% petroleum ether) to give the desired product **2e** as a colorless oil (23.07 mg, 81% yield). **^{1}H NMR** (400 MHz, $CDCl_3$) δ 7.02 (t, J = 7.8 Hz, 1H), 6.74 (d, J = 7.4 Hz, 1H), 6.69 (d, J = 8.2 Hz, 1H), 4.19 – 4.12 (m, 2H), 2.66 (t, J = 6.6 Hz, 2H), 2.22 (s, 3H), 2.09 – 2.01 (m, 2H). **^{13}C NMR** (101 MHz, $CDCl_3$) δ 155.00, 137.59, 126.52, 121.58, 121.01, 114.49, 77.20, 65.80, 22.46, 19.02. **HRMS (ESI)** m/z: M+H$^+$ calculated for $C_{10}H_{13}O^+$: 149.0961, found 149.09616.

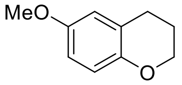

2f: 6-methoxychromane.

2f was synthesized according to the general synthetic method with **1f** (59.79 mg, 0.305 mmol), Re_2O_7 (2.85 mg, 0.00588 mmol), and HFIP (1.5 mL). The reaction was stirred at 100 °C for 12 hours then quenched with 20 µL Et_3N, concentrated under reduced pressure and purified through flash chromatography (100% petroleum ether) to give the desired product **2f** as a colorless oil (35.11 mg, 74% yield). **^{1}H NMR** (400 MHz, $CDCl_3$) δ 6.78 – 6.62 (m, 2H), 6.59 (s, 1H), 4.18 – 4.09 (m, 2H), 3.75 (s, 3H), 2.77 (t, J = 6.5 Hz, 2H), 2.03 – 1.94 (m, 2H). **^{13}C NMR** (101 MHz, $CDCl_3$) δ 153.13, 148.92, 122.69, 117.16, 114.28, 113.20, 66.28, 55.65, 25.11, 22.40. **HRMS (ESI)** m/z: M+H$^+$ calculated for $C_{10}H_{13}O^+$: 149.0961, found 149.09615.

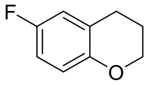

2g: 6-fluorochromane.

2g was synthesized according to the general synthetic method with **1g** (25.31 mg, 0.137 mmol), Re_2O_7 (1.29 mg, 0.00266 mmol), and HFIP (0.67 mL). The reaction was stirred at 100 °C for 12 hours then quenched with 20 µL Et_3N, concentrated under reduced pressure and purified through flash chromatography (100% petroleum ether) to give the desired product **2g** as a colorless oil (16.90 mg, 81% yield). **^{1}H NMR** (400 MHz, $CDCl_3$) δ 6.87 – 6.60 (m, 3H), 4.18 – 4.12 (m, 2H), 2.77 (t, J = 6.5 Hz, 2H), 2.02 – 1.95 (m, 2H). **^{13}C NMR** (101 MHz, $CDCl_3$) δ 156.61 (d, $^1J_{CF}$ = 238.36 Hz), 150.87 (d, $^4J_{CF}$ = 2.02 Hz), 123.22 (d, $^3J_{CF}$ = 7.07 Hz), 117.44 (d, $^3J_{CF}$ = 8.28 Hz), 115.51 (d, $^2J_{CF}$ = 22.22 Hz), 113.81 (d, $^2J_{CF}$ = 23.23 Hz), 66.36, 24.97, 22.05. **^{19}F NMR** (565 MHz, $CDCl_3$) δ -75.65. **HRMS (ESI)** m/z: M+H$^+$ calculated for $C_9H_{10}FO^+$: 153.0710, found 153.0712.

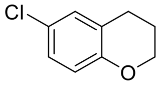

2h: 6-chlorochromane.

2h was synthesized according to the general synthetic method with **1h** (46.15 mg, 0.230 mmol), Re_2O_7 (2.31 mg, 0.00477 mmol), and HFIP (1.2 mL). The reaction was stirred at 100 °C for 12 hours then quenched with 20 µL Et_3N, concentrated under reduced pressure and purified through flash chromatography (100% petroleum ether) to give the desired product **2h** as a colorless oil (30.11 mg, 78% yield). **^{1}H NMR** (400 MHz, $CDCl_3$) δ 7.12 – 6.91 (m, 2H), 6.77 – 6.62 (m, 1H), 4.19 – 4.14 (m, 2H), 2.76 (t, J = 6.5 Hz, 2H), 2.02 – 1.95 (m, 2H). **^{13}C NMR** (101 MHz, $CDCl_3$) δ 153.47, 129.26, 127.10, 124.65, 123.70, 117.96, 66.45, 24.75, 21.94. **HRMS (ESI)** m/z: M+H$^+$ calculated for $C_9H_{10}ClO^+$: 169.0415, found 169.0417.

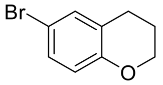

2i: 6-bromochromane.

2i was synthesized according to the general synthetic method with **1i** (28.04 mg, 0.108 mmol), Re_2O_7 (1.05 mg, 0.00217 mmol), and HFIP (0.54 mL). The reaction was stirred at 100 °C for 12 hours then quenched with 20 µL Et_3N, concentrated under reduced pressure and purified

through flash chromatography (100% petroleum ether) to give the desired product **2i** as a colorless oil (19.79 mg, 82% yield). **^{1}H NMR** (600 MHz, $CDCl_3$) δ 7.19 – 7.12 (m, 2H), 6.69 – 6.64 (m, 1H), 4.17 – 4.15 (m, 2H), 2.76 (t, J = 6.5 Hz, 2H), 2.00 – 1.96 (m, 2H). **^{13}C NMR** (101 MHz, $CDCl_3$) δ 154.00, 132.21, 130.01, 124.32, 118.47, 112.00, 66.45, 24.70, 21.91. **HRMS (ESI)** m/z: M+H$^+$ calculated for $C_9H_{10}BrO^+$: 212.9910, found 212.9911.

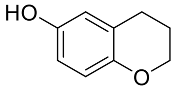

2j: Chroman-6-ol.

2j was synthesized according to the general synthetic method with **1j** (17.86 mg, 0.098 mmol), Re_2O_7 (0.95 mg, 0.00196mmol), and HFIP (0.50 mL). The reaction was stirred at 100 °C for 12 hours then quenched with 20 μL Et_3N, concentrated under reduced pressure and purified through flash chromatography (PE/EA = 10/ 1) to give the desired product **2j** as a colorless oil (7.66 mg, 51% yield). **^{1}H NMR** (600 MHz, $CDCl_3$) δ 6.66 (d, J = 8.7 Hz, 1H), 6.57 (dd, J = 8.7, 3.0 Hz, 1H), 6.53 (d, J = 3.1 Hz, 1H), 4.55 (s, br, 1H), 4.20 – 4.05 (m, 2H), 2.74 (t, J = 6.5 Hz, 2H), 2.03 – 1.94 (m, 2H). **^{13}C NMR** (101 MHz, $CDCl_3$) δ 148.95, 148.80, 123.03, 117.29, 115.80, 114.30, 66.32, 24.96, 22.37. **HRMS (ESI)** m/z: M+H$^+$ calculated for $C_9H_{11}O_2^+$: 151.0754, found 151.0756.

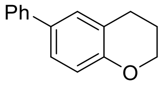

2k: 6-phenylchromane.

2k was synthesized according to the general synthetic method with **1k** (33.59 mg, 0.139 mmol), Re_2O_7 (1.32 mg, 0.00272 mmol), and HFIP (0.68 mL). The reaction was stirred at 100 °C for 12 hours then quenched with 20 μL Et_3N, concentrated under reduced pressure and purified through flash chromatography (100% petroleum ether) to give the desired product **2k**as a white solid (21.03mg, 73% yield). **^{1}H NMR** (600 MHz, $CDCl_3$) δ 7.57 – 7.51 (m, 2H), 7.42 – 7.39 (m, 2H), 7.36 – 7.27 (m, 3H), 6.87 (d, J = 8.4 Hz, 1H), 4.25 – 4.21 (m, 2H), 2.86 (t, J = 6.5 Hz, 2H), 2.07 – 2.02 (m, 2H). **^{13}C NMR** (101 MHz, $CDCl_3$) δ 154.55, 141.01, 133.26, 128.64, 128.45, 126.67, 126.50, 126.01, 122.37, 117.04, 66.58, 25.03, 22.40. **HRMS (ESI)** m/z: M+H$^+$ calculated for $C_{15}H_{15}O^+$: 211.1117, found 211.1119.

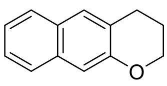

2l: 3,4-dihydro-2H-benzo-chromene.

2l was synthesized according to the general synthetic method with **1l** (32.87 mg, 0.152 mmol), Re_2O_7 (1.48 mg, 0.00305 mmol), and HFIP (0.76 mL). The reaction was stirred at 100 °C for 12 hours then quenched with 20 μL Et_3N, concentrated under reduced pressure and purified through flash chromatography (100% petroleum ether) to give the desired product **2l** as a white solid (22.02 mg, 78% yield). **^{1}H NMR** (400 MHz, $CDCl_3$) δ 7.70 (t, J = 7.0 Hz, 2H), 7.55 (s, 1H), 7.40– 7.36 (m, 1H), 7.33 – 7.28 (m, 1H), 7.22 (s, 1H), 4.34 – 4.28 (m, 2H), 3.05 (t, J = 5.9 Hz, 2H), 2.14 – 2.07 (m, 2H). **^{13}C NMR** (101 MHz, $CDCl_3$) δ 153.37, 133.49, 128.60, 128.14, 126.95, 126.25, 125.46, 124.33, 123.31, 111.01, 66.78, 25.65, 22.50. **HRMS (ESI)** m/z: M+H$^+$ calculated for $C_{13}H_{13}O^+$: 185.0961, found 185.0962.

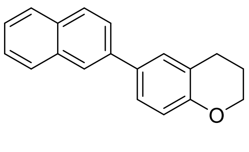

2m: 6-(naphthalen-2-yl)chromane.

2m was synthesized according to the general synthetic method with **1m** (25.76 mg, 0.088 mmol), Re_2O_7 (0.83 mg, 0.00171 mmol), and HFIP (0.43 mL). The reaction was stirred at 100 °C for 12 hours then quenched with 20 μL Et_3N, concentrated under reduced pressure and purified through flash chromatography (100% petroleum ether) to give the desired product **2m** as a white solid (13.98 mg, 63% yield). **^{1}H NMR** (400 MHz, $CDCl_3$) δ 7.97 (s, 1H), 7.93 – 7.80 (m, 3H), 7.71 (dd, J = 8.5, 1.9 Hz, 1H), 7.55 – 7.36 (m, 4H), 6.91 (d, J = 8.4

Hz, 1H), 4.28 – 4.20 (m, 2H), 2.90 (t, *J* = 6.5 Hz, 2H), 2.11 – 2.03 (m, 2H). **^{13}C NMR** (101 MHz, $CDCl_3$) δ 154.67, 138.33, 133.74, 133.08, 132.25, 128.70, 128.24, 128.00, 127.59, 126.27, 126.15, 125.53, 125.44, 124.88, 122.52, 117.17, 66.62, 25.07, 22.41. **HRMS (ESI)** m/z: M+H$^+$ calculated for $C_{19}H_{17}O^+$: 261.1274, found 261.1272.

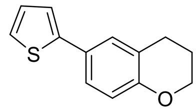

2n: 6-(thiophen-2-yl) chromane.

2n was synthesized according to the general synthetic method with **1n** (13.71 mg, 0.055 mmol), Re_2O_7 (0.99 mg, 0.00204 mmol), and HFIP (0.40 mL). The reaction was stirred at 120 °C for 24 hours then quenched with 20 μL Et_3N, concentrated under reduced pressure and purified through flash chromatography (100% petroleum ether) to give the desired product **2n** as a white solid (4.01 mg, 27% yield). **^{1}H NMR** (400 MHz, $CDCl_3$) δ 7.33 (dd, *J* = 8.4, 2.4 Hz, 1H), 7.30 – 7.27 (m, 1H), 7.24 – 7.13 (m, 2H), 7.05 – 7.03 (m, 1H), 6.80 (d, *J* = 8.5 Hz, 1H), 4.24 – 4.18 (m, 2H), 2.82 (t, *J* = 6.5 Hz, 2H), 2.06 – 2.00 (m, 2H). **^{13}C NMR** (151 MHz, $CDCl_3$) δ 154.64, 144.58, 127.84, 127.45, 126.71, 125.15, 123.61, 122.50, 121.87, 117.11, 66.60, 24.91, 22.31. **HRMS (ESI)** m/z: M+H$^+$ calculated for $C_{13}H_{13}OS^+$: 217.0682, found 217.0684.

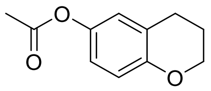

2o: Chroman-6-yl acetate.

2o was synthesized according to the general synthetic method with **1o** (23.11 mg, 0.100 mmol), Re_2O_7 (0.97 mg, 0.00200 mmol), and HFIP (0.5 mL). The reaction was stirred at 100 °C for 12 hours then quenched with 20 μL Et_3N, concentrated under reduced pressure and purified through flash chromatography (PE/EA = 10) to give the desired product **2o** as a colorless oil (5.77 mg, 30% yield). **^{1}H NMR** (600 MHz, $CDCl_3$) δ 6.76 (d, *J* = 7.2 Hz, 3H), 4.21 – 4.12 (m, 2H), 2.78 (t, *J* = 6.5 Hz, 2H), 2.26 (s, 3H), 2.01 – 1.96 (m, 2H). **^{13}C NMR** (101 MHz, $CDCl_3$) δ 170.06, 152.61, 143.51, 122.87, 122.25, 120.21, 117.30, 66.43, 24.92, 22.03, 21.05. **HRMS (ESI)** m/z: M+H$^+$ calculated for $C_{11}H_{13}O_3^+$: 193.0859, found 193.0860.

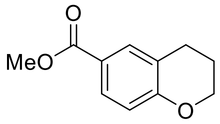

2p: methyl chromane-6-carboxylate.

2p was synthesized according to the general synthetic method with **1p** (51.47 mg, 0.226 mmol), Re_2O_7 (2.19 mg, 0.00452 mmol), and HFIP (1.13 mL). The reaction was stirred at 100 °C for 12 hours then quenched with 20 μL Et_3N, concentrated under reduced pressure and purified through flash chromatography (PE/EA = 20) to give the desired product **2p** as a white solid (10.86 mg, 25% yield). **^{1}H NMR** (600 MHz, $CDCl_3$) δ 7.49 (dd, *J* = 7.9, 1.7 Hz, 1H), 7.45 (d, *J* = 1.7 Hz, 1H), 7.08 (d, *J* = 7.9 Hz, 1H), 4.22 – 4.18 (m, 2H), 3.88 (s, 3H), 2.83 (t, *J* = 6.5 Hz, 2H), 2.04 – 2.00 (m, 2H). **^{13}C NMR** (101 MHz, $CDCl_3$) δ 154.80, 129.76, 129.29, 127.61, 121.08, 117.98, 66.49, 51.99, 25.09, 21.96. **HRMS (ESI)** m/z: M+H$^+$ calculated for $C_{11}H_{13}O_3^+$: 193.0859, found 193.0858.

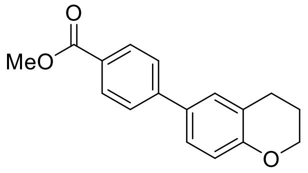

2q: Methyl 4-(chroman-6-yl) benzoate.

2q was synthesized according to the general synthetic method with **1q** (25.80 mg, 0.084 mmol), Re_2O_7 (0.81 mg, 0.00167 mmol), and HFIP (0.42 mL). The reaction was stirred at 100 °C for 12 hours then quenched with 20 μL Et_3N, concentrated under reduced pressure and purified through flash chromatography (PE/EA = 20) to give the desired product **2q** as a white solid (20.42 mg, 81% yield). **^{1}H NMR** (600 MHz, $CDCl_3$) δ 8.06 (d, *J* = 8.4 Hz, 2H), 7.60 (d, *J* = 8.4 Hz, 2H), 7.37 (dd,

J = 8.4, 2.3 Hz, 1H), 7.34 – 7.29 (m, 1H), 6.88 (d, *J* = 8.4 Hz, 1H), 4.24 – 4.22 (m, 2H), 3.93 (s, 3H), 2.86 (t, *J* = 6.5 Hz, 2H), 2.07 – 2.03 (m, 2H). **^{13}C NMR** (151 MHz, $CDCl_3$) δ 167.08, 155.26, 145.37, 131.80, 130.02, 128.62, 128.02, 126.35, 126.13, 122.60, 117.24, 66.63, 52.04, 24.99, 22.25. **HRMS (ESI)** m/z: M+H$^+$ calculated for $C_{17}H_{17}O_3^+$: 269.1172, found 269.1173.

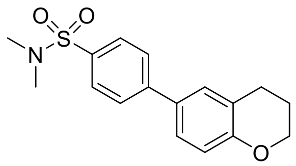

2r: 4-(Chroman-6-yl)-N,N-dimethylbenzenesulfonamide.

2r was synthesized according to the general synthetic method with **1r** (26.60, 0.076 mmol), Re_2O_7 (1.11 mg, 0.00229 mmol), and HFIP (0.38 mL). The reaction was stirred at 120 °C for 24 hours then quenched with 20 μL Et_3N, concentrated under reduced pressure and purified through flash chromatography (PE/EA = 5) to give the desired product **2r** as a white solid (10.85, 45% yield). **^{1}H NMR** (600 MHz, $CDCl_3$) δ 7.79 (d, *J* = 8.5 Hz, 2H), 7.68 (d, *J* = 8.4 Hz, 2H), 7.35 (dd, *J* = 8.4, 2.4 Hz, 1H), 7.31 (dd, *J* = 2.3, 1.1 Hz, 1H), 6.89 (d, *J* = 8.4 Hz, 1H), 4.27 – 4.21 (m, 2H), 2.87 (t, *J* = 6.5 Hz, 2H), 2.73 (s, 6H), 2.08 – 2.03 (m, 2H). **^{13}C NMR** (101 MHz, $CDCl_3$) δ 155.59, 145.40, 133.17, 131.11, 128.73, 128.22, 126.92, 126.20, 122.80, 117.43, 66.68, 37.98, 25.02, 22.24. **HRMS (ESI)** m/z: M+H$^+$ calculated for $C_{17}H_{20}NO_3S^+$: 318.1158, found 318.1159.

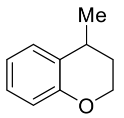

2s: 4-methylchromane.

2s was synthesized according to the general synthetic method with **1s** (35.47 mg, 0.197 mmol), Re_2O_7 (1.90 mg, 0.00392 mmol), and HFIP (1.00 mL). The reaction was stirred at 100 °C for 12 hours then quenched with 20 μL Et_3N, concentrated under reduced pressure and purified through flash chromatography (100% petroleum ether) to give the desired product **2s** as a colorless oil (23.90 mg, 82% yield). **^{1}H NMR** (600 MHz, $CDCl_3$) δ 7.15 (dd, *J* = 7.7, 1.6 Hz, 1H), 7.08 (t, *J* = 7.7 Hz, 1H), 6.86 (td, *J* = 7.4, 1.4 Hz, 1H), 6.79 (d, *J* = 8.1 Hz, 1H), 4.23 – 4.15 (m, 2H), 2.97 – 2.94 (m, 1H), 2.11 – 2.06 (m, 1H), 1.75 – 1.70 (m, 1H), 1.33 (dd, *J* = 7.0, 1.1 Hz, 3H). **^{13}C NMR** (151 MHz, $CDCl_3$) δ 154.31, 128.64, 127.60, 127.20, 120.19, 116.69, 63.84, 30.30, 28.48, 22.19. **HRMS (ESI)** m/z: M+H$^+$ calculated for $C_{10}H_{13}O^+$: 149.0961, found 149.0962.

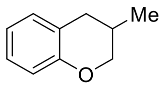

2t: 3-methylchromane.

2t was synthesized according to the general synthetic method with **1t** (20.51 mg, 0.114 mmol), Re_2O_7 (1.13 mg, 0.00233 mmol), and HFIP (0.60 mL). The reaction was stirred at 100 °C for 12 hours then quenched with 20 μL Et_3N, concentrated under reduced pressure and purified through flash chromatography (100% petroleum ether) to give the desired product **2t** as a colorless oil (8.43 mg, 50% yield). **^{1}H NMR** (600 MHz, $CDCl_3$) δ 7.08 (t, *J* = 7.8 Hz, 1H), 7.03 (d, *J* = 7.5 Hz, 1H), 6.89 – 6.71 (m, 2H), 4.19 – 4.17 (m, 1H), 3.71–3.67 (m, 1H), 2.85–2.81 (m, 1H), 2.47–2.43 (m, 1H), 2.20 – 2.11 (m, 1H), 1.05 (d, *J* = 6.8 Hz, 3H). **^{13}C NMR** (151 MHz, $CDCl_3$) δ 154.40, 129.78, 127.14, 121.83, 120.16, 116.39, 71.81, 33.28, 27.07, 17.01. **HRMS (ESI)** m/z: M+H$^+$ calculated for $C_{10}H_{13}O^+$: 149.0961, found 149.0963.

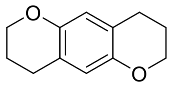

2u: 2,3,4,7,8,9-hexahydropyrano[2,3-g]chromene.

2u was synthesized according to the general synthetic method with **1u** (25.10 mg, 0.099 mmol), Re_2O_7 (1.39 mg, 0.00287 mmol), and HFIP (0.48 mL). The reaction was stirred at 120 °C for 24 hours then quenched with 20 μL Et_3N, concentrated under reduced pressure and purified through flash chromatography (100% petroleum ether) to give the desired product **2u** as a white

solid (7.00 mg, 38% yield). **^{1}H NMR** (400 MHz, $CDCl_3$) δ 6.61 (s, 2H), 4.11 – 4.06 (m, 4H), 2.57 (t, *J* = 6.6 Hz, 4H), 2.06 – 1.98 (m, 4H). **^{13}C NMR** (151 MHz, $CDCl_3$) δ 148.60, 120.84, 115.36, 65.60, 22.38, 22.04. **HRMS (ESI)** m/z: M+H$^+$ calculated for $C_{12}H_{15}O_2^+$: 191.1067, found 191.1068.

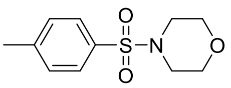

2z: 4-(p-tolyl) morpholine[13].

2z was synthesized according to the general synthetic method with **1z** (33.82 mg, 0.124 mmol), Re_2O_7 (2.58 mg, 0.00533 mmol), and HFIP (0.50 mL). The reaction was stirred at 120 °C for 24 hours then quenched with 20 μL Et_3N, concentrated under reduced pressure and purified through flash chromatography (100% petroleum ether) to give the desired product **2z** as a yellow solid (22.11 mg, 74% yield). **^{1}H NMR** (600 MHz, $CDCl_3$) δ 7.64 (d, *J* = 8.0 Hz, 2H), 7.34 (d, *J* = 7.9 Hz, 2H), 3.74 (t, *J* = 4.7 Hz, 4H), 3.01 – 2.95 (m, 4H), 2.44 (s, 3H).

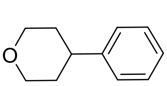

2bb: 4-phenyltetrahydro-2H-pyran[5].

2bb was synthesized according to the general synthetic method with **1bb** (28.07 mg, 0.144 mmol), Re_2O_7 (1.40 mg, 0.00289 mmol), and HFIP (0.72 mL). The reaction was stirred at 100 °C for 12 hours then quenched with 20 μL Et_3N, concentrated under reduced pressure and purified through flash chromatography (100% petroleum ether) to give the desired product **2bb** as a colorless oil (21.11 mg, 89% yield). **^{1}H NMR** (400 MHz, $CDCl_3$) δ7.31 – 7.35 (m, 2H), 7.24 – 7.20 (m, 3H), 4.14 – 4.04 (m, 2H), 3.54 (td, *J* = 11.5, 2.7 Hz, 2H), 2.85 – 2.68 (m, 1H), 1.90 – 1.75 (m, 4H).

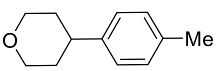

2cc: 4-(p-tolyl) tetrahydro-2H-pyran[5].

2cc was synthesized according to the general synthetic method with **1cc** (20.40 mg, 0.098 mmol), Re_2O_7 (1.04 mg, 0.00215 mmol), and HFIP (0.50 mL). The reaction was stirred at 100 °C for 12 hours then quenched with 20 μL Et_3N, concentrated under reduced pressure and purified through flash chromatography (100% petroleum ether) to give the desired product **2cc** as a colorless oil (12.97 mg, 75% yield). **^{1}H NMR** (400 MHz, $CDCl_3$) δ 7.14 (s, 4H), 4.10 – 4.06 (m, 2H), 3.53 (td, *J* = 11.5, 2.8 Hz, 2H), 2.78 – 2.67 (m, 1H), 2.34 (s, 3H), 1.87 – 1.74 (m, 4H).

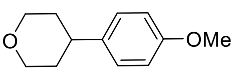

2dd: 4-(4-methoxyphenyl) tetrahydro-2H-pyran.

2dd was synthesized according to the general synthetic method with **1dd** (39.04 mg, 0.174 mmol), Re_2O_7 (1.61 mg, 0.00332 mmol), and HFIP (0.83 mL). The reaction was stirred at 100 °C for 12 hours then quenched with 20 μL Et_3N, concentrated under reduced pressure and purified through flash chromatography (100% petroleum ether) to give the desired product **2dd** as a colorless oil (29.10 mg, 87% yield). **^{1}H NMR** (400 MHz, $CDCl_3$) δ 7.16 – 7.13(m, 2H), 6.88 – 6.85 (m, 2H), 4.10 – 4.04 (m, 2H), 3.80 (s, 3H), 3.52 (td, *J* = 11.4, 3.2 Hz, 2H), 2.78 – 2.64 (m, 1H), 1.85 – 1.72 (m, 4H). **^{13}C NMR** (151 MHz, $CDCl_3$) δ 158.02, 138.09, 127.57, 113.86, 68.43, 55.24, 40.67, 34.18; **HRMS (ESI)** m/z: M+H$^+$ calculated for $C_{12}H_{17}O_2^+$: 193.1223, found 193.1225.

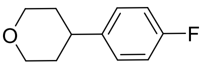

2ee: 4-(4-fluorophenyl) tetrahydro-2H-pyran[5].

2ee was synthesized according to the general synthetic method with **1ee** (21.20 mg, 0.100 mmol), Re_2O_7 (0.86 mg, 0.00178 mmol), and HFIP (0.40 mL). The reaction was stirred at 100 °C for 12 hours then quenched with 20 μL Et_3N, concentrated under reduced pressure and purified through flash chromatography (100% petroleum ether) to give the desired product **2ee** as a colorless oil (13.00 mg, 72% yield). **^{1}H NMR** (400 MHz, $CDCl_3$) δ 7.19 – 7.16 (m, 2H), 7.02 – 6.98 (m, 2H), 4.11 – 4.04 (m, 2H), 3.52 (td, *J* = 11.3, 3.5 Hz, 2H), 2.80 – 2.68 (m, 1H), 1.84 – 1.72 (m, 4H). **^{13}C NMR** (101 MHz,

$CDCl_3$) δ 161.37 (d, ${}^{1}J_{CF}$ = 245.43 Hz), 140.52 (d, ${}^{4}J_{CF}$ = 3.03 Hz), 128.04 (d, ${}^{3}J_{CF}$ = 8.08 Hz), 115.20 (d, ${}^{2}J_{CF}$ = 21.21 Hz), 68.31, 40.84, 34.08. **^{19}F NMR** (565 MHz, $CDCl_3$) δ -117.04; **HRMS (ESI)** m/z: $M+H^+$ calculated for $C_{11}H_{14}FO^+$: 181.1023, found 181.1024.

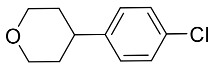

2ff: 4-(4-chlorophenyl) tetrahydro-2H-pyran[5].
2ff was synthesized according to the general synthetic method with **1ff** (25.72 mg, 0.112 mmol), Re_2O_7 (1.08 mg, 0.00223 mmol), and HFIP (0.56 mL). The reaction was stirred at 100 °C for 12 hours then quenched with 20 μL Et_3N, concentrated under reduced pressure and purified through flash chromatography (100% petroleum ether) to give the desired product **2ff** as a colorless oil (17.89 mg, 81% yield). **^{1}H NMR** (400 MHz, $CDCl_3$) δ 7.33 – 7.28 (m, 2H), 7.22 – 7.11 (m, 2H), 4.14 – 4.04 (m, 2H), 3.54 (td, J = 11.3, 3.4 Hz, 2H), 2.81 – 2.68 (m, 1H), 1.82 – 1.77 (m, 4H).

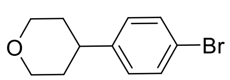

2gg: 4-(4-Bromophenyl) tetrahydro-2H-pyran.
2gg was synthesized according to the general synthetic method with **1gg** (49.10 mg, 0.176 mmol), Re_2O_7 (1.71 mg, 0.00353 mmol), and HFIP (0.88 mL). The reaction was stirred at 100 °C for 12 hours then quenched with 20 μL Et_3N, concentrated under reduced pressure and purified through flash chromatography (100% petroleum ether) to give the desired product **2hh** as a colorless oil (38.19 mg, 90% yield). **^{1}H NMR** (600 MHz, $CDCl_3$) δ 7.43 (d, J = 8.4 Hz, 2H), 7.10 (d, J = 8.4 Hz, 2H), 4.16 – 3.99 (m, 2H), 3.52 (td, J = 11.5, 2.7 Hz, 2H), 2.79 – 2.64 (m, 1H), 1.87 – 1.69 (m, 4H). **^{13}C NMR** (101 MHz, $CDCl_3$) δ 144.78, 131.55, 128.48, 119.94, 68.22, 41.04, 33.77. **HRMS (ESI)** m/z: $M+H^+$ calculated for $C_{11}H_{14}BrO^+$: 241.0223, found 241.0225.

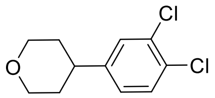

2hh: 4-(3,4-dichlorophenyl) tetrahydro-2H-pyran.
2hh was synthesized according to the general synthetic method with **1hh** (39.04 mg, 0.174 mmol), Re_2O_7 (1.61 mg, 0.00332 mmol), and HFIP (0.83 mL). The reaction was stirred at 100 °C for 12 hours then quenched with 20 μL Et_3N, concentrated under reduced pressure and purified through flash chromatography (100% petroleum ether) to give the desired product **2hh** as a colorless oil (19.98 mg, 91% yield). **^{1}H NMR** (400 MHz, $CDCl_3$) δ 7.37 (d, J = 8.3 Hz, 1H), 7.30 (s, 1H), 7.05 (dd, J = 8.3, 2.1 Hz, 1H), 4.10 – 4.05 (m, 2H), 3.55 – 3.46 (m, 2H), 2.76 – 2.68 (m, 1H), 1.79 – 1.72 (m, 4H). ^{13}C NMR (101 MHz, $CDCl_3$) δ 146.00, 132.41, 130.43, 130.13, 128.83, 126.17, 68.09, 40.80, 33.63. **HRMS (ESI)** m/z: $M+H^+$ calculated for $C_{11}H_{13}Cl_2O^+$: 231.0338, found 231.0339.

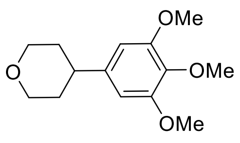

2ii: 4-(3,4,5-trimethoxyphenyl) tetrahydro-2H-pyran.
2ii was synthesized according to the general synthetic method with **1ii** (26.62 mg, 0.094 mmol), Re_2O_7 (0.86 mg, 0.00177 mmol), and HFIP (0.44 mL). The reaction was stirred at 100 °C for 12 hours then quenched with 20 μL Et_3N, concentrated under reduced pressure and purified through flash chromatography (PE/EA = 10) to give the desired product **2ii** as a white solid (17.05 mg, 72% yield). **^{1}H NMR** (400 MHz, $CDCl_3$) δ 6.44 (s, 2H), 4.11 – 4.05 (m, 2H), 3.86 (s, 6H), 3.83 (s, 3H), 3.52 (td, J = 11.4, 3.1 Hz, 2H), 2.76 – 2.63 (m, 1H), 1.86 – 1.75 (d, J = 10.0 Hz, 4H). **^{13}C NMR** (151 MHz, $CDCl_3$) δ 153.20, 141.74, 136.40, 103.65, 68.34, 60.81, 56.06, 41.98, 34.09. **HRMS (ESI)** m/z: $M+H^+$ calculated for $C_{14}H_{21}O_4^+$: 253.1434, found 253.1436.

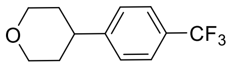

2jj: 4-(4-(trifluoromethyl)phenyl) tetrahydro-2H-pyran.

2jj was synthesized according to the general synthetic method with **1jj** (32.65 mg, 0.124 mmol), Re_2O_7 (2.93 mg, 0.00605 mmol), and HFIP (0.60 mL). The reaction was stirred at 120 °C for 24 hours then quenched with 20 µL Et_3N, concentrated under reduced pressure and purified through flash chromatography (100% petroleum ether) to give the desired product **2jj** as a colorless oil (24.51 mg, 86% yield). **^{1}H NMR** (400 MHz, $CDCl_3$) δ 7.57 (d, J = 8.1 Hz, 2H), 7.34 (d, J = 8.0 Hz, 2H), 4.11 – 4.08 (m, 2H), 3.54 (td, J = 11.5, 2.8 Hz, 2H), 2.91 – 2.74 (m, 1H), 1.90 – 1.74 (m, 4H). **^{13}C NMR** (101 MHz, $CDCl_3$) δ 149.74, 128.64 (q, $^2J_{CF}$ = 33.33 Hz), 127.09, 125.46 (q, $^3J_{CF}$ = 4.04 Hz), 124.43 (q, $^1J_{CF}$ = 272.7 Hz), 68.17, 41.47, 33.62. **^{19}F NMR** (565 MHz, $CDCl_3$) δ -62.37. **HRMS (ESI)** m/z: M+H$^+$ calculated for $C_{12}H_{14}F_3O^+$: 231.0991, found 231.0994.

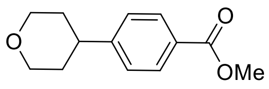

2kk: Methyl 4-(tetrahydro-2H-pyran-4-yl)benzoate.

2kk was synthesized according to the general synthetic method with **1kk** (35.02 mg, 0.135 mmol), Re_2O_7 (1.97 mg, 0.00407 mmol), and HFIP (0.68 mL). The reaction was stirred at 120 °C for 24 hours then quenched with 20 µL Et_3N, concentrated under reduced pressure and purified through flash chromatography (PE/EA = 20) to give the desired product **2kk** as a white solid (28.25 mg, 95% yield). **^{1}H NMR** (600 MHz, $CDCl_3$) δ 7.99 (d, J = 8.3 Hz, 2H), 7.29 (d, J = 8.3 Hz, 2H), 4.12 – 4.06 (m, 2H), 3.90 (s, 3H), 3.53 (td, J = 11.7, 2.3 Hz, 2H), 2.86 – 2.78 (m, 1H), 1.87 – 1.75 (m, 4H). **^{13}C NMR** (101 MHz, $CDCl_3$) δ 167.01, 151.06, 129.90, 128.29, 126.78, 68.21, 52.02, 41.65, 33.57. **HRMS (ESI)** m/z: M+H$^+$ calculated for $C_{13}H_{17}O_3^+$: 221.1172, found 221.1173.

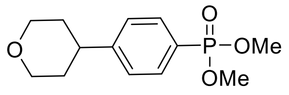

2ll: Dimethyl (4-(tetrahydro-2H-pyran-4-yl)phenyl)phosphonate.

2ll was synthesized according to the general synthetic method with **1ll** (29.31 mg, 0.097 mmol), Re_2O_7 (1.41 mg, 0.00291 mmol), and HFIP (0.50 mL). The reaction was stirred at 100 °C for 12 hours then quenched with 20 µL Et_3N, concentrated under reduced pressure and purified through flash chromatography (PE/EA = 1) to give the desired product **2ll** as a white solid (24.12 mg, 92% yield). **^{1}H NMR** (600 MHz, $CDCl_3$) δ 7.81 – 7.68 (m, 2H), 7.39 – 7.30 (m, 2H), 4.09 (dd, J = 11.8, 4.1 Hz, 2H), 3.77 (s, 3H), 3.75 (s, 3H), 3.53 (td, J = 11.7, 2.3 Hz, 2H), 2.86 – 2.76 (m, 1H), 1.87 – 1.73 (m, 4H). **^{13}C NMR** (101 MHz, $CDCl_3$) δ 185.07, 150.76, 132.79, 126.99, 68.15, 52.70, 41.64, 33.50. **^{31}P NMR** (243 MHz, $CDCl_3$) δ 22.01. **HRMS (ESI)** m/z: M+H$^+$ calculated for $C_{12}H_{20}O_4P^+$: 271.1094, found 271.1095.

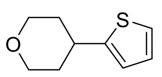

2mm: 4-(thiophen-2-yl) tetrahydro-2H-pyran.

2 mm was synthesized according to the general synthetic method with **1 mm** (29.55 mg, 0.147 mmol), Re_2O_7 (1.38 mg, 0.00285 mmol), and HFIP (0.70 mL). The reaction was stirred at 100 °C for 12 hours then quenched with 20 µL Et_3N, concentrated under reduced pressure and purified through flash chromatography (100% petroleum ether) to give the desired product **2 mm** as a colorless oil (18.55 mg, 75% yield). **^{1}H NMR** (600 MHz, $CDCl_3$) δ 7.16 – 7.15 (m, 1H), 6.96 – 6.94 m, 1H), 6.86 – 6.80 (m, 1H), 4.06 – 4.04 (m, 2H), 3.52 (t, J = 11.8 Hz, 2H), 3.11 – 3.02 (m, 1H), 1.97 – 1.92 (m, 2H), 1.86 – 1.79 (m, 2H). **^{13}C NMR** (151 MHz, $CDCl_3$) δ 150.05, 126.61, 122.69, 122.14, 67.92, 36.53, 34.95. **HRMS (ESI)** m/z: M+H$^+$ calculated for $C_9H_{12}OS^+$: 169.0682, found 169.0681.

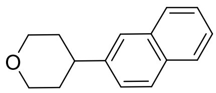

2nn: 4-(naphthalen-2-yl) tetrahydro-2H-pyran.

2nn was synthesized according to the general synthetic method with **1n** (22.07 mg, 0.090 mmol), Re_2O_7 (0.85 mg, 0.00175 mmol), and HFIP (0.44 mL). The reaction was stirred at 100 °C for 12 hours then quenched with 20 µL Et_3N, concentrated under reduced pressure and purified through flash chromatography (100% petroleum ether) to give the desired product **2nn** as a white solid (18.05 mg, 94% yield). **^{1}H NMR** (400 MHz, $CDCl_3$) δ 7.87 – 7.76 (m, 3H), 7.66 (s, 1H), 7.51 – 7.35 (m, 3H), 4.14 (dd, *J* = 11.0, 3.8 Hz, 2H), 3.59 (td, *J* = 11.7, 2.5 Hz, 2H), 2.99 – 2.87 (m, 1H), 2.01 – 1.84 (m, 4H). **^{13}C NMR** (101 MHz, $CDCl_3$) δ 143.27, 133.60, 132.26, 128.06, 127.61, 127.56, 125.98, 125.67, 125.34, 124.69, 68.41, 41.64, 33.87. **HRMS (ESI)** m/z: M+H$^+$ calculated for $C_{15}H_{17}O^+$: 213.1274, found 213.1275.

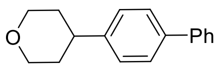

2oo: 4-([1,1'-biphenyl]-4-yl) tetrahydro-2H-pyran.

2oo was synthesized according to the general synthetic method with **1oo** (55.73 mg, 0.206 mmol), Re_2O_7 (1.98 mg, 0.00409 mmol), and HFIP (1.00 mL). The reaction was stirred at 100 °C for 12 hours then quenched with 20 µL Et_3N, concentrated under reduced pressure and purified through flash chromatography (100% petroleum ether) to give the desired product **2oo** as a white solid (39.88 mg, 81% yield). **^{1}H NMR** (400 MHz, $CDCl_3$) δ 7.66 – 7.54 (m, 4H), 7.46 (dd, *J* = 8.4, 6.9 Hz, 2H), 7.40 – 7.30 (m, 3H), 4.16 – 4.10 (m, 2H), 3.58 (td, *J* = 11.5, 2.8 Hz, 2H), 2.89 – 2.78 (m, 1H), 1.96 – 1.81 (m, 4H). **^{13}C NMR** (101 MHz, $CDCl_3$) δ 144.93, 140.93, 139.28, 128.71, 127.24, 127.13, 127.08, 126.99, 68.38, 41.22, 33.92; **HRMS (ESI)** m/z: M+H$^+$ calculated for $C_{17}H_{19}O^+$: 239.1430, found 239.1432.

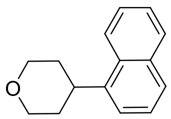

2pp: 4-(naphthalen-1-yl) tetrahydro-2H-pyran.

2pp was synthesized according to the general synthetic method with **1pp** (22.29 mg, 0.091 mmol), Re_2O_7 (0.84 mg, 0.00173 mmol), and HFIP (0.44 mL). The reaction was stirred at 100 °C for 12 hours then quenched with 20 µL Et_3N, concentrated under reduced pressure and purified through flash chromatography (100% petroleum ether) to give the desired product **2pp** as a white solid (14.96 mg, 77% yield). **^{1}H NMR** (400 MHz, $CDCl_3$) δ 8.12 (d, *J* = 8.3 Hz, 1H), 7.88 (d, *J* = 7.7 Hz, 1H), 7.74 (d, *J* = 8.0 Hz, 1H), 7.58 – 7.32 (m, 4H), 4.19 – 4.14 (m, 2H), 3.71 (td, *J* = 11.4, 2.8 Hz, 2H), 3.65 – 3.56 (m, 1H), 2.03 – 1.87 (m, 4H). **^{13}C NMR** (101 MHz, $CDCl_3$) δ 141.53, 134.00, 131.14, 129.14, 126.81, 125.90, 125.72, 125.44, 122.79, 122.57, 77.26, 68.74, 36.70, 33.80, 1.06. **HRMS (ESI)** m/z: M+H$^+$ calculated for $C_{15}H_{16}O^+$: 213.1274, found 213.1273.

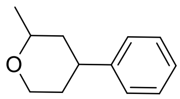

2qq: 2-Methyl-4-phenyltetrahydro-2H-pyran.

2qq was synthesized according to the general synthetic method with **1qq** (29.23 mg, 0.14 mmol), Re_2O_7 (1.37 mg, 0.00283 mmol), and HFIP (0.70 mL). The reaction was stirred at 80 °C for 12 hours then quenched with 20 µL Et_3N, concentrated under reduced pressure and purified through flash chromatography (100% petroleum ether) to give the desired product **2qq** as a colorless oil (15.05 mg, 61% yield). **^{1}H NMR** (600 MHz, $CDCl_3$) δ 7.34 – 7.30 (m, 2H), 7.23 – 7.20 (m, 3H), 4.16 – 4.08 (m, 1H), 3.63 – 3.53 (m, 2H), 2.83 – 2.75 (m, 1H), 1.84 – 1.82 (m, 1H), 1.78 – 1.73 (m, 2H), 1.49 – 1.41 (m, 1H), 1.24 (d, *J* = 6.2 Hz, 3H). **^{13}C NMR** (101 MHz, $CDCl_3$) δ 145.84, 128.49, 126.73, 126.26, 73.79, 68.15, 41.79, 41.19, 33.31, 22.06. **HRMS (ESI)** m/z: M+H$^+$ calculated for $C_{12}H_{17}O^+$: 177.1274, found 177.1277.

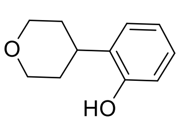

2rr: 2-(Tetrahydro-2H-pyran-4-yl) phenol.

2rr was synthesized according to the general synthetic method with **1rr** (13.52 mg, 0.065 mmol), Re_2O_7 (0.63 mg, 0.00130 mmol), and HFIP (0.33 mL). The reaction was stirred at 100 °C for 12 hours then quenched with 20 μL Et_3N, concentrated under reduced pressure and purified through flash chromatography (PE/EA = 10) to give the desired product **2rr** as a colorless oil (7.65 mg, 66% yield). **^{1}H NMR** (600 MHz, $CDCl_3$) δ 7.19 (dd, *J* = 7.6, 1.6 Hz, 1H), 7.09 (td, *J* = 7.7, 1.7 Hz, 1H), 6.93 (td, *J* = 7.5, 1.2 Hz, 1H), 6.74 (dd, *J* = 8.0, 1.2 Hz, 1H), 5.07 (s, br, 1H), 4.10 (dd, *J* = 11.6, 4.6 Hz, 2H), 3.59 (td, *J* = 11.7, 2.3 Hz, 2H), 3.19 – 3.12 (m, 1H), 1.88 – 1.77 (m, 4H). **^{13}C NMR** (101 MHz, $CDCl_3$) δ 152.90, 131.84, 127.05, 127.03, 121.02, 115.22, 68.59, 34.60, 32.46. **HRMS (ESI)** m/z: $M+H^+$ calculated for $C_{11}H_{15}O_2^+$: 179.1067, found 179.1068

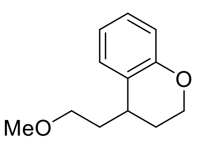

2rr': 4-(2-Methoxyethyl) chromane.

2rr' was synthesized according to the general synthetic method with **1rr** (13.52 mg, 0.065 mmol), Re_2O_7 (0.63 mg, 0.00130 mmol), and HFIP (0.33 mL). The reaction was stirred at 100 °C for 12 hours then quenched with 20 μL Et_3N, concentrated under reduced pressure and purified through flash chromatography (PE/EA = 10) to give the desired product **2rr'** as a colorless oil (1.75 mg, 14% yield). **^{1}H NMR** (600 MHz, $CDCl_3$) δ 7.13 (d, *J* = 9.0 Hz, 1H), 7.09 (t, *J* = 7.9 Hz, 1H), 6.86 (td, *J* = 7.4, 1.3 Hz, 1H), 6.80 (d, *J* = 8.1 Hz, 1H), 4.24 – 4.14 (m, 2H), 3.54 – 3.47 (m, 2H), 3.38 (s, 3H), 3.02 – 2.95 (m, 1H), 2.14 – 2.05 (m, 2H), 1.85 – 1.76 (m, 2H). **^{13}C NMR** (101 MHz, $CDCl_3$) δ 154.49, 129.21, 127.34, 125.96, 120.09, 116.80, 70.21, 63.30, 58.64, 36.12, 30.42, 27.01. **HRMS (ESI)** m/z: $M+H^+$ calculated for $C_{12}H_{17}O_2^+$: 193.1223, found 193.1225

4 Supplementary Discussion

4.1 Mechanistic experiments

4.1.1 Cyclization of alkylated substrates and dihydroxy substrates

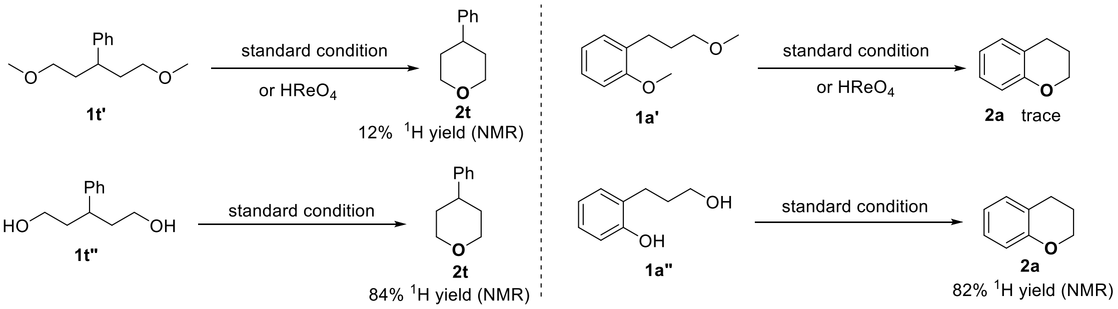

Supplementary Figure 8. cyclization of alkylated substrates

Methylated substrates **1a'**,**1t'** and dihydroxy substrates **1a''**, **1t''** reacted under standard conditions respectively (Supplementary Figure 8), which showed the importance of free OH in Re_2O_7 catalyzed reaction.

4.1.2 Selective transetherification between ether and alcohol.

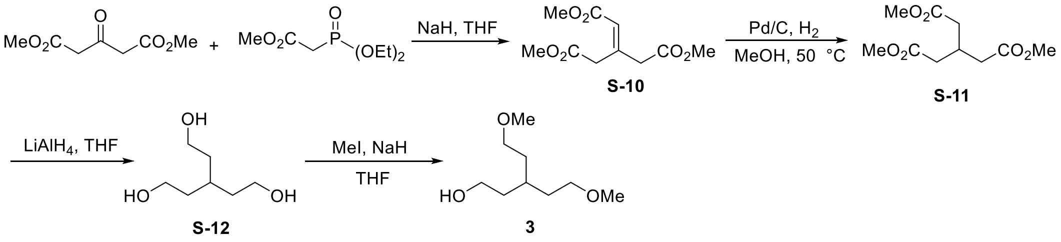

Supplementary Figure 9. Synthesis of **3**

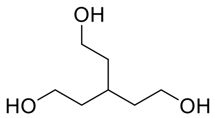

S-12: 3-(2-hydroxyethyl) pentane-1,5-diol

Lithium aluminium hydride (1.5 equiv.) is suspended in dry THF under nitrogen. A solution of **S-11** (1.0 equiv.) in dry THF is added dropwise and keeped the solution refluxing for 3 hours. Then the reaction mixture cooled with ice bath, quenched with a minimum amount of water (3-5 drops). This suspension was extracted with hot ethanol (5×5 mL). The solvent is evaporated under reduced pressure to yield the product **S-12** as a yellow oil. **S-12** was synthesized according to the literature report[14]. **^{1}H NMR** (400 MHz, Methanol-d$_4$) δ 3.63 (t, J = 6.9 Hz, 6H), 1.76-1.70 (m, 1H), 1.59-1.54 (q, J = 6.8 Hz 6H).

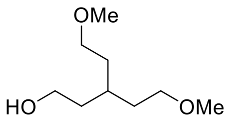

3: 5-methoxy-3-(2-methoxyethyl) pentan-1-ol. **^{1}H NMR** (400 MHz, $CDCl_3$) δ 3.66 (t, J = 6.5 Hz, 2H), 3.44-3.36 (m, 4H), 3.31 (s, 6H), 1.75-1.69 (m, 1H), 1.60 – 1.52 (m, 6H). **^{13}C NMR** (101 MHz, $CDCl_3$) δ 71.0, 60.7, 58.6, 36.9, 34.0, 29.3.

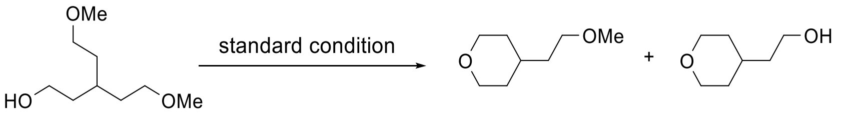

3 **4, 75%** **4', 5%**

Supplementary Figure 10. Selective transetherification between ether and alcohol.

Reaction with substrate **3** was performed under standard condition, the ratio of products was determined with by analyzing ^{1}H NMR of the reaction crude with mesitylene as an internal standard.

4.1.3 Effect of the alkyl group of 1a

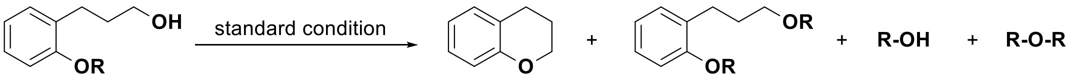

1a **2** **1a'**

		2	1a'	R-OH	R-O-R
**^{1}H NMR yield:**	**R = Me**	86%	14%	39%	NA
	**R = Et**	87%	trace	83%	6%
	**R = *n*-Pr**	98%	trace	64%	trace
	**R = *n*-Br**	96%	trace	54%	trace

Supplementary Figure 11: effect of the alkyl group of the reaction

1a with different alkyl groups (Me, Et, ^{n}Pr, ^{n}Bu) reacted under standard condition respectively and detected by NMR. By identifying different characteristic peaks of the products, we analyzed the ratio of different products (Supplementary Figure 11).

Efficiency improvement by tuning the alkyl groups of ethers

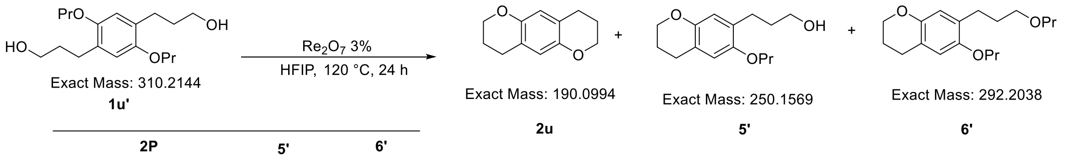

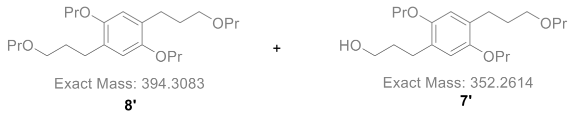

	2P	5'	6'
time	12.825	14.373	14.709
Ms	**190.12**	**250.14**	**292.18**
%	**56**	19	13

The GC-MS result

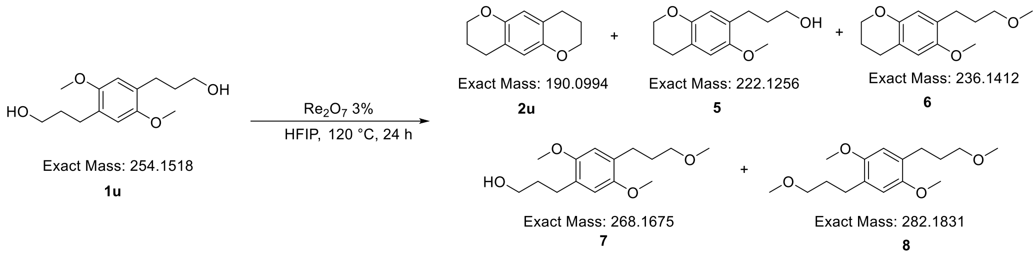

	2u	6	5	8	7
time	12.781	13.318	13.655	13.857	14.171
Ms	190.08	236.13	222.12	282.16	268.13
%	**27**	28	26	8	11

The GC-MS result

Supplementary Figure 12. Alkylation of substrate **1u** and **1u'**

Reactions with **1u** and **1u'** were conducted according to the general synthetic method (Re_2O_7 3%, HFIP, 120 °C, 24 hours) and then quenched with 20 μL Et_3N, measured by GC-MS and the results showed in the Supplementary Figure 12. The yield of **2u** increased significantly when the alkyl moiety changed from Me to ^{n}Pr and the alkylation by-products decreased.

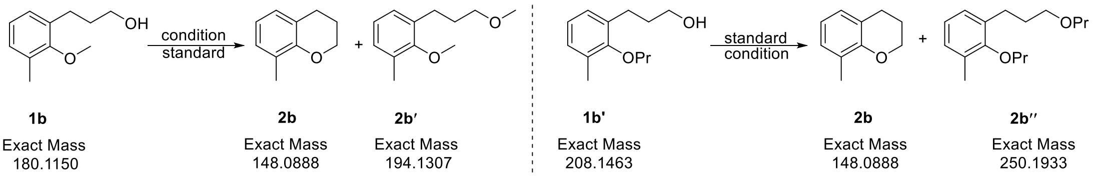

	2b	2b′
time	9.617	10.694
Ms	**148.11**	**194.09**
%	58	28

The GC-MS result

	2b	2b″
time	9.606	12.432
Ms	**148.06**	**250.17**
%	39	6

The GC-MS result

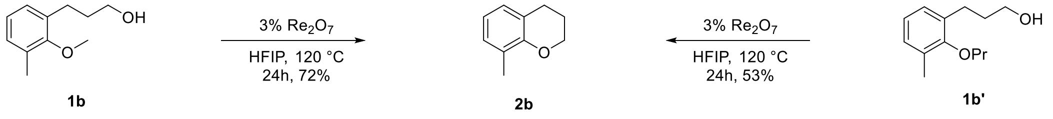

Supplementary Figure 13. Alkylation of substrate **1b** and **1b'**

1b and **1b'** are conducted according to the general synthetic method at 100 ℃ for 12 hours then quenched with 20 μL Et_3N, measured by GC-MS and the results showed in the Supplementary Figure 13. The increased steric hindrance of **1b** made the substrate alkylation easier but the formation of product

harder. The propyl-substrate **1b'** doesn't show positive effect similar to **1u'**, probably due to the increased steric hindrance as well.

4.1.4 ^{18}O labeling experiments

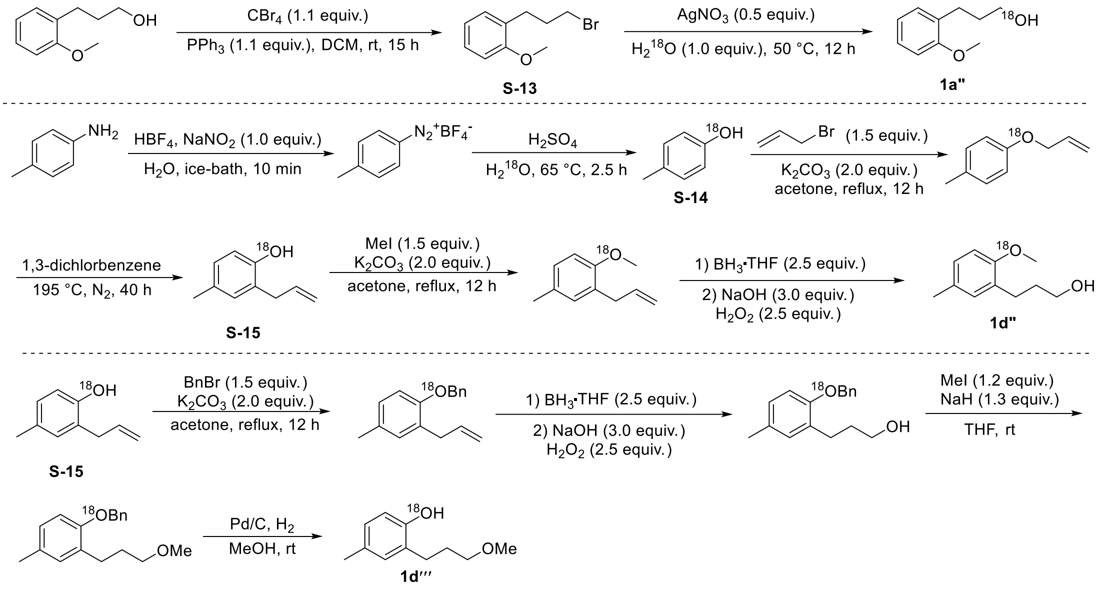

Supplementary Figure 14. Synthesis of ^{18}O-labeled substrates **1a''** and **1d''**

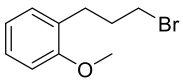

S-13: 1-(3-bromopropyl)-2-methoxybenzene

Phenylpropanol (1.660g, 10 mmol) and CBr_4(3.658g, 11 mmol) were dissolved in CH_2Cl_2 (20 mL) and cooled to 0 °C (ice bath), PPh_3 (2.886g, 11 mmol) was added in portions to the mixture, then the reaction was stirred at room temperature for 12 hours. Afterwards, the solvent was removed under reduced pressure and purification by flash column chromatography (SiO_2, PE/EA = 50/1) to give a yellow liquid **S-13** (94% yield). **S-13** was synthesized according to the literature report [15].

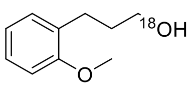

1a'': 3-(2-methoxyphenyl) propan-1-ol-^{18}O.

A mixture of phenethyl bromide (**S-13)** (2 mmol), $AgNO_3$ (1 mmol, 0.5 equiv.) and $H_2{}^{18}O$ (10 mmol, 1.0 equiv. 98 atom % ^{18}O) was stirred at 50 °C for 12 hours. The resulting mixture was filtered, the filtrate was concentrated under reduced pressure and purified by flash column chromatography (SiO_2, PE/EA = 5/1), affording ^{18}O-labeled phenethyl alcohol **1a''**. **1a''** was synthesized according to the literature report [16]. **^{1}H NMR** (600 MHz, $CDCl_3$) δ 7.19 (td, *J* = 7.7, 1.8 Hz, 1H), 7.15 (dd, *J* = 7.4, 1.7 Hz, 1H), 6.91 (t, *J* = 7.4 Hz, 1H), 6.86 (d, *J* = 8.2 Hz, 1H), 3.84 (s, 3H), 3.60 (t, *J* = 6.2 Hz, 2H), 2.73 (t, *J* = 7.3 Hz, 2H), 1.87 – 1.84 (m, 2H). **^{13}C NMR** (101 MHz, $CDCl_3$) δ 157.31, 130.03, 129.90, 127.11, 120.64, 110.24, 61.84, 55.27, 32.80, 25.86.

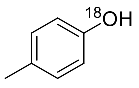

S-14: Synthesis ^{18}O-labeled phenol

Aniline (4.273 g, 40 mmol) was dissolved in water (10 mL) and 48% tetrafluoroboric acid (12.4 mL) was added. The solution was cooled to 0 °C (ice bath), and a solution of sodium nitrite (2.768 g, 40 mmol) in water (12 mL) was added dropwise. The suspension was stirred at 0 °C (ice bath) for 20 min, and the mixture was filtered, solid materials were purified by re-precipitation from

acetone/diethyl ether (5:1) solution. Benzenediazonium tetrafluoroborate was dried under reduced pressure and obtained in 75% yield (6.178g, 30 mmol).

Concentrated sulfuric acid (125 μL) was added to a stirred paste of benzenediazonium tetrafluoroborate (1.003g, 4.9 mmol) in 98% [^{18}O] water (0.7 mL, 98 atom % ^{18}O). The mixture was then heated to 65 °C until evolution of nitrogen ceased. The solution was extracted with ethyl acetate (5×10 mL). The organic layer was washed with HCl (10 mL, 1 M) and saturated brine, dried over anhydrous $MgSO_4$, and concentrated under reduced pressure. The residue was purified by sublimation to give the ^{18}O-labelled phenol **S-14** as colorless solid (323.8 mg, 60%). **S-14** was synthesized according to the literature report [17].

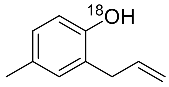

S-15: 2-allyl-4-methylphenol-^{18}O

A flame-dried round bottom flask was charged with the (allyloxy)benzene (8.0 mmol, 1.0 equiv.) in 1,3-dichlorbenzene (20 mL). And then the reaction was heated to reflux behind a blast shield for 24 hours. After cooling to the room temperature, Et_2O (20 mL) was added and the mixture was extracted with 20% aq. KOH solution (4×20 mL). The combined aqueous layer was acidified to pH=1 with 6M HCl, and the mixture was extracted with DCM (3 × 20 mL). The combined organic layers were washed with water (20 mL) and brine sequentially, dried over anhydrous Na_2SO_4, filtered and concentrated under reduced pressure. The crude product was purified by flash chromatography (SiO_2, PE/EA = 50/1) to afford the corresponding 2-allylphenol. **S-15** was synthesized according to the literature report [18].

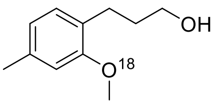

1d'': 3-(2-(methoxy)-4-methylphenyl) propan-1-ol.

Colorless oil. **^{1}H NMR** (400 MHz, $CDCl_3$) δ 7.00 – 6.95 (m, 2H), 6.78 – 6.75 (m, 1H), 3.81 (s, 3H), 3.59 (t, J = 6.2 Hz, 2H), 2.69 (t, J = 7.2 Hz, 2H), 2.27 (s, 3H), 1.84 (p, J = 6.7 Hz, 2H). **^{13}C NMR** (151 MHz, $CDCl_3$) δ 155.26, 130.92, 129.89, 129.63, 127.37, 110.28, 61.83, 55.49, 33.02, 25.75, 20.42.

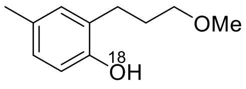

1d''': 2-(3-Methoxypropyl)-4-methylphenol-^{18}O.

Colorless oil. **^{1}H NMR** (600 MHz, $CDCl_3$) δ 6.93 (s, br, 1H), 6.92 – 6.88 (m, 2H), 6.77 (d, J = 8.1 Hz, 1H), 3.41 (s, 3H), 3.37 (t, J = 5.8 Hz, 2H), 2.69 (t, J = 6.7 Hz, 2H), 2.25 (s, 3H), 1.89 (m, 2H). **^{13}C NMR** (151 MHz, $CDCl_3$) δ 152.74, 130.99, 129.53, 128.11, 126.47, 116.15, 70.26, 58.45, 29.67, 25.40, 20.44.

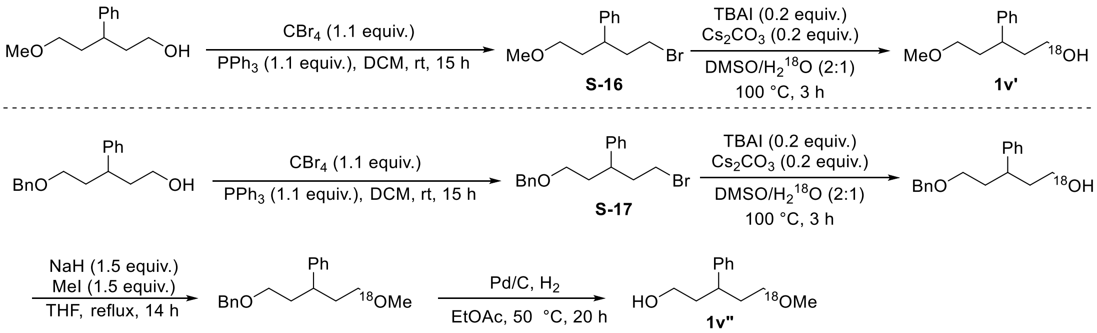

Supplementary Figure 15. Synthesis of ^{18}O-labeled substrates **1v'** and **1v''**

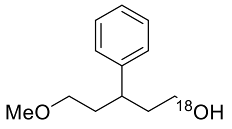

1v': 5-methoxy-3-phenylpentan-1-ol-^{18}O.

The mixture of **S-16** (393.32 mg, 1.53 mmol), extra dry DMSO (0.5 mL), $H_2{}^{18}O$ (0.25 mL, 98 atom % ^{18}O), Cs_2CO_3 (102.86 mg, 0.31 mmol), and TBAI (114.50 mg, 0.31 mmol) was sealed in a Schlenk tube (10 mL) under N_2 atmosphere and then stirred at 100 °C for 3 hours. Purification by flash column chromatography (SiO_2, PE/EA = 3/ 1). The ^{18}O-labeled **1v'** was obtained in 45% isolated yield as a pale-yellow oil. **^{1}H NMR** (400 MHz, $CDCl_3$) δ 7.32 – 7.28 (m, 2H), 7.23 – 7.15 (m, 3H), 3.57 – 3.44 (m, 2H), 3.25 (s, 3H), 3.27 – 3.22 (m, 1H), 3.19 – 3.13 (m, 1H), 2.90 – 2.85 (m, 1H), 2.00 – 1.79 (m, 4H). **^{13}C NMR** (101 MHz, $CDCl_3$) δ 144.38, 128.44, 127.53, 126.25, 70.54, 60.73, 58.45, 39.38, 38.76, 36.39.

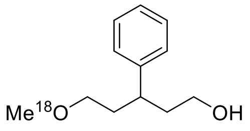

1v'': 5-(methoxy)-3-phenylpentan-1-ol. **^{1}H NMR** (600 MHz, $CDCl_3$) δ 7.31 – 7.29 (m, 2H), 7.24 – 7.15 (m, 3H), 3.56 – 3.45 (m, 2H), 3.26 – 3.23 (m, 1H), 3.25 (s, 3H), 3.19 – 3.13 (m, 1H), 2.89 – 2.86 (m, 1H), 2.00 – 1.95 (m, 2H), 1.87 – 1.79 (m, 2H). **^{13}C NMR** (101 MHz, $CDCl_3$) δ 144.41, 128.48, 127.56, 126.30, 70.55, 60.91, 58.50, 39.43, 38.88, 36.48.

Reactions with ^{18}O-labeled substrates (**1a''**, **1d'', 1d''', 1v'**, **1v''**) were performed under the standard condition (Re_2O_7/HFIP) (Supplementary Figure 16.), when the reaction was completed, 20 μL Et_3N was added to quench the reaction and 3 μL mesitylene was added as the internal standard. and GC-MS, respectively. The yield and ^{18}O incorporation are measured by NMR.

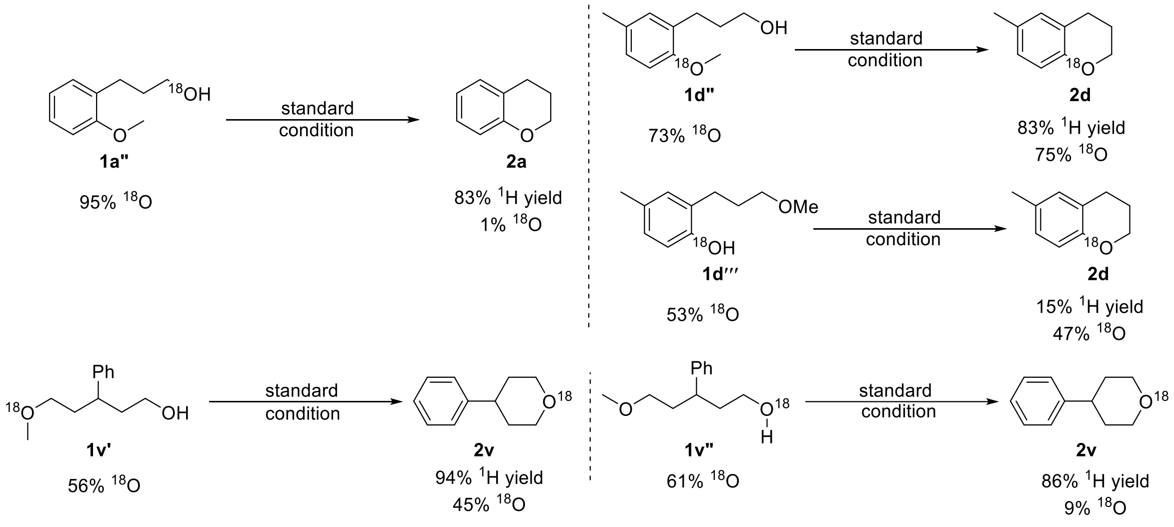

Supplementary Figure 16. The experiments of **1a''** and **1d''**

4.1.5 Radical Trap Experiments

To a 10 mL Schlenk tube was added Re_2O_7 (2.68 mg, 0.0055 mmol), substrate **1a** (46.21 mg, 0.277 mmol), TEMPO (44.67 mg, 0.277 mmol) and HFIP (1.4 ml). The reaction mixture was stirred at 100 °C for 12 hours, then the reaction was quenched by adding a proper amount of Et_3N, and the solvent was then removed under reduced pressure. There is no reaction measured by NMR.

To a 10 mL Schlenk tube was added Re_2O_7 (2.89 mg, 0.0060 mmol), substrate **1a** (50.29 mg, 0.30 mmol), duroquinone (49.14 mg, 0.30 mmol) and HFIP (1.5 ml). The reaction mixture was stirred at 100 °C for 12 hours, then the reaction was quenched by adding a proper amount of Et_3N, and the solvent was then removed under reduced pressure. The yield of **2a** is 79% measured by NMR.

To a 10 mL Schlenk tube was added Re_2O_7 (0.76 mg, 0.0016 mmol), substrate **1a** (13.24 mg, 0.078 mmol), 1,1-diphenylethylene (14.06 mg, 0.078 mmol) and HFIP (0.39 ml). The reaction mixture was stirred at 100 °C for 12 hours, then the reaction was quenched by adding a proper amount of Et_3N, and the solvent was then removed under reduced pressure. The yield of **2a** is 58% measured by NMR.

To a 10 mL Schlenk tube was added Re_2O_7 (0.84 mg, 0.0017 mmol), substrate **1a** (15.04 mg, 0.087 mmol), 2,6-di-tert-butyl-4-methylphenol (19.07 mg, 0.087 mmol) and HFIP (0.44 ml). The reaction mixture was stirred at 100 °C for 12 hours, then the reaction was quenched by adding a proper amount of Et_3N, and the solvent was then removed under reduced pressure. The yield of **2a** is 82% measured by NMR.

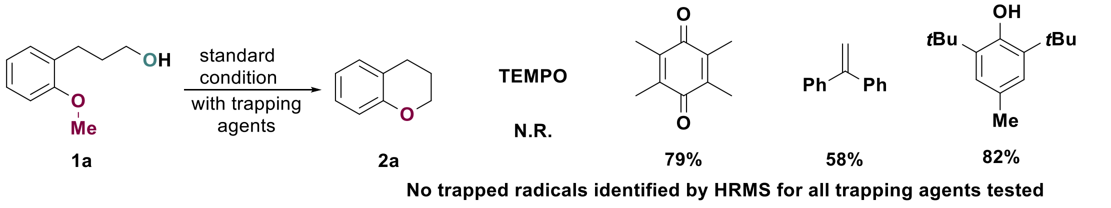

Supplementary Figure 17. Radical Trap Experiments

4.2 DFT calculations

All DFT calculations were conducted with Gaussian 16 software[19] with the B3LYP-D3[20,21] functional. Geometries were optimized in the gas phase in conjunction with a mixture of basis sets (SDD pseudopotential[22] for Re, def2-SVP basis sets for all other atoms). Frequency calculations of all the optimized structures were performed at the same theoretical level to confirm their nature, and also to obtain the Gibbs free energy corrections. Single-point energies were computed at the B3LYP-D3/def2-TZVPP level in the solvent, and the solvent effects were introduced using the SMD[23] model. Since HFIP is not available as a default solvent in Gaussian 16 software, the solvent effects of HFIP were evaluated by using the SMD model of 2-propanol together with reading the static dielectric constant of HFIP at 25°C (Eps = 16.7).

As shown in Supplementary Figure 18, pathway a assisted with one HFIP (black line) and without HFIP (red line) were theoretically investigated, and the calculated results suggested that one HFIP could only lower the overall energy by less than 1 kcal/mol (**TS3A**, total barrier of 29.8 kcal/mol vs **TS3B**, 29.1 kcal/mol). However, with the assistance of two HFIPs, the total energy barrier decreases to 24.3 kcal/mol

(TS3 in Fig. 5). Similar trends of the calculated results for pathways b and d were also obtained. As seen in Supplementary Figure 19, the energy barriers of **TS1'-A** and **TS1'-B** are almost the same but are more than 7 kcal/mol higher than **TS1'**, which involves two HFIPs. Additionally, pathway d assisted with two HFIPs (Supplementary Figure 20) is calculated to be at least 3.5 kcal/mol lower than with one HFIP (Supplementary Figure 21). Moreover, a concerted transition state for C-O/C-O σ-bond metathesis at **Int1** that directly leads to product **2a** and $MeOReO_3$ could not be located in the current theoretical calculation. Attempts to locate such structures always led to transition states along the step-wise mechanism (pathway a in Fig. 5). Taken together, pathway a involving the assistance of two HFIPs is the most favored one based on the calculated results, and HFIP as the solvent could possibly help to stabilize the critical transition states and ionic species, and thus promote the corresponding transformations.

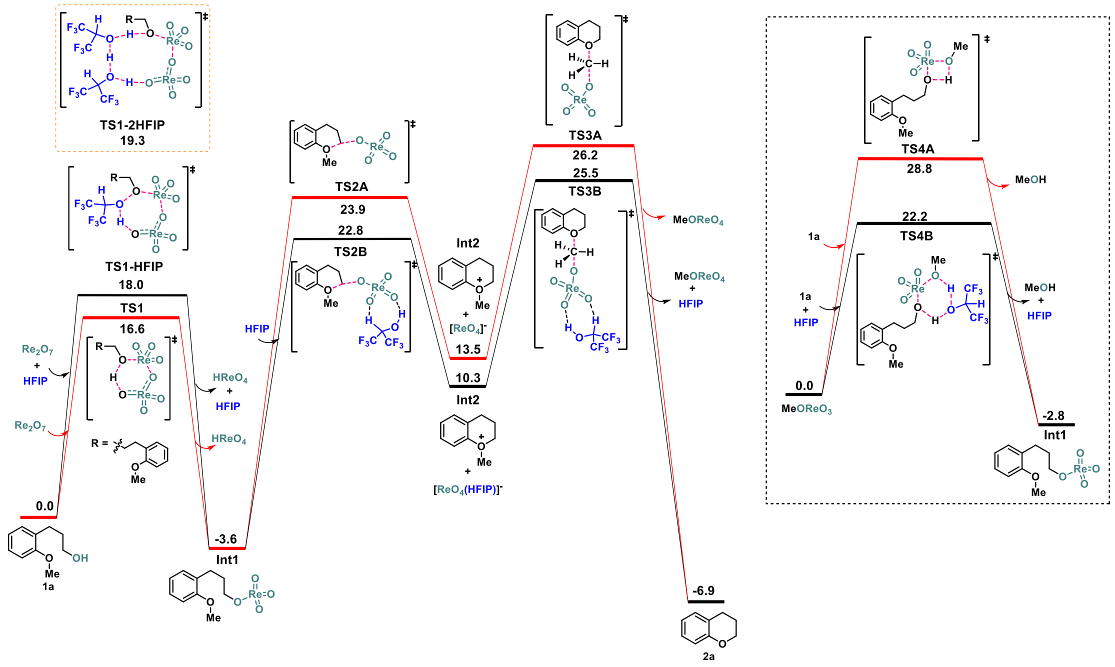

Supplementary Figure 18. Gibbs free energy profile (kcal/mol) of pathway a without HFIP (red line) and with one HFIP (black line) at the SMD-B3LYP-D3/def2-TZVPP//B3LYP-D3/def2-SVP-SDD(Re) level for the ring-closing C-O/C-O σ-bond metathesis of 1a catalyzed with Re_2O_7.

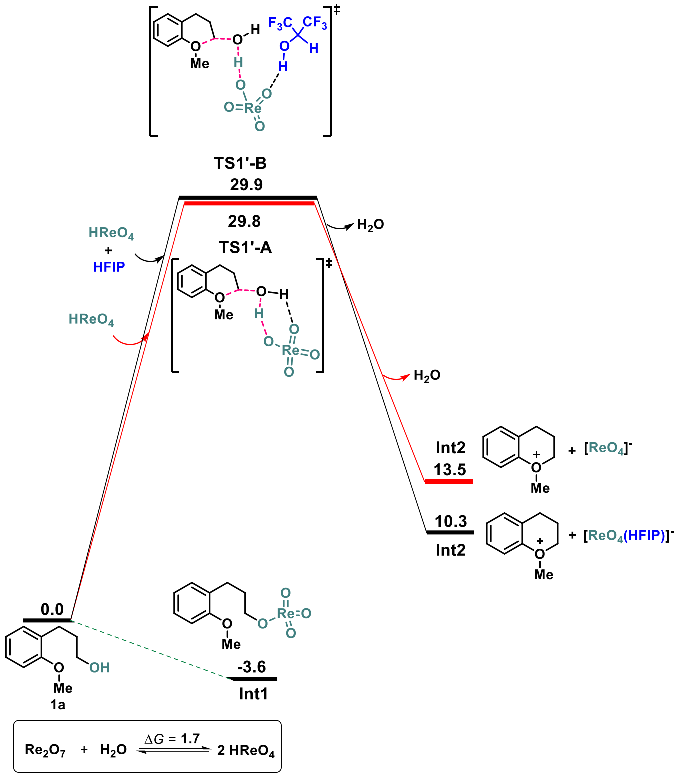

Supplementary Figure 19. Gibbs free energy profile (kcal/mol) of pathway b without HFIP (red line) and with one HFIP (black line) at the SMD-B3LYP-D3/def2-TZVPP//B3LYP-D3/def2-SVP-SDD(Re) level for the ring-closing C-O/C-O σ-bond metathesis of 1a catalyzed with Re_2O_7.

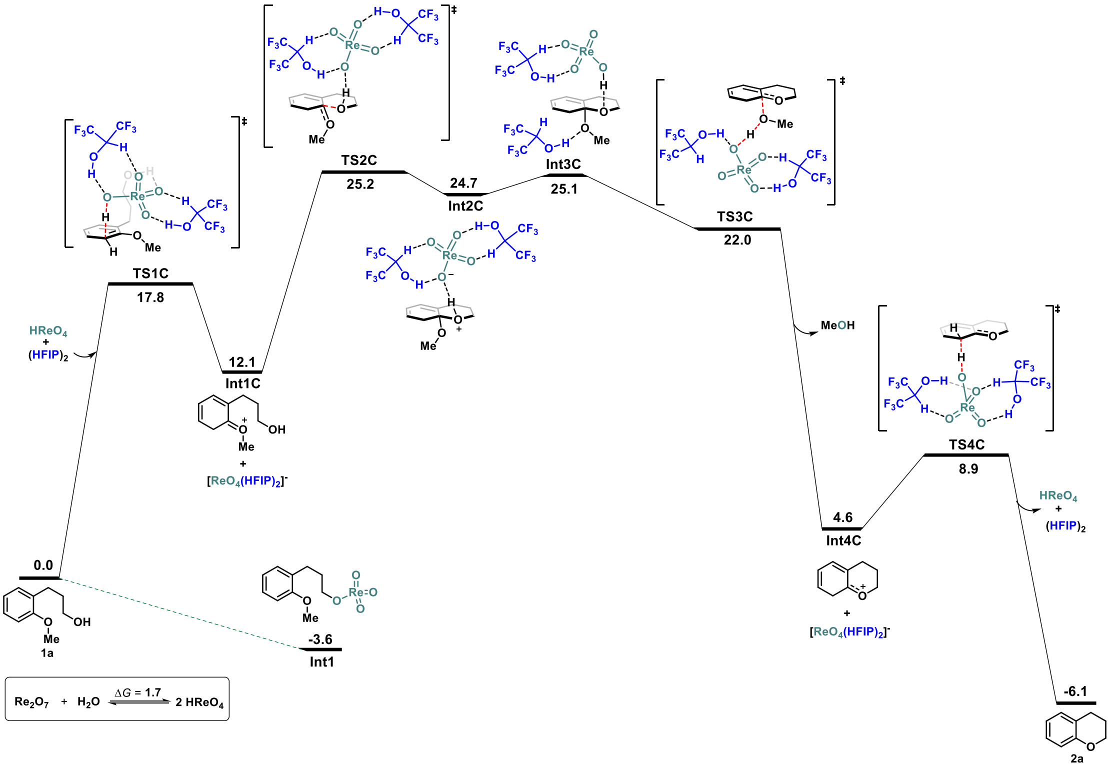

Supplementary Figure 20. Gibbs free energy profile (kcal/mol) of pathway d with two HFIPs at the SMD-B3LYP-D3/def2-TZVPP//B3LYP-D3/def2-SVP-SDD(Re) level for the ring-closing C-O/C-O σ-bond metathesis of 1a catalyzed with Re_2O_7.

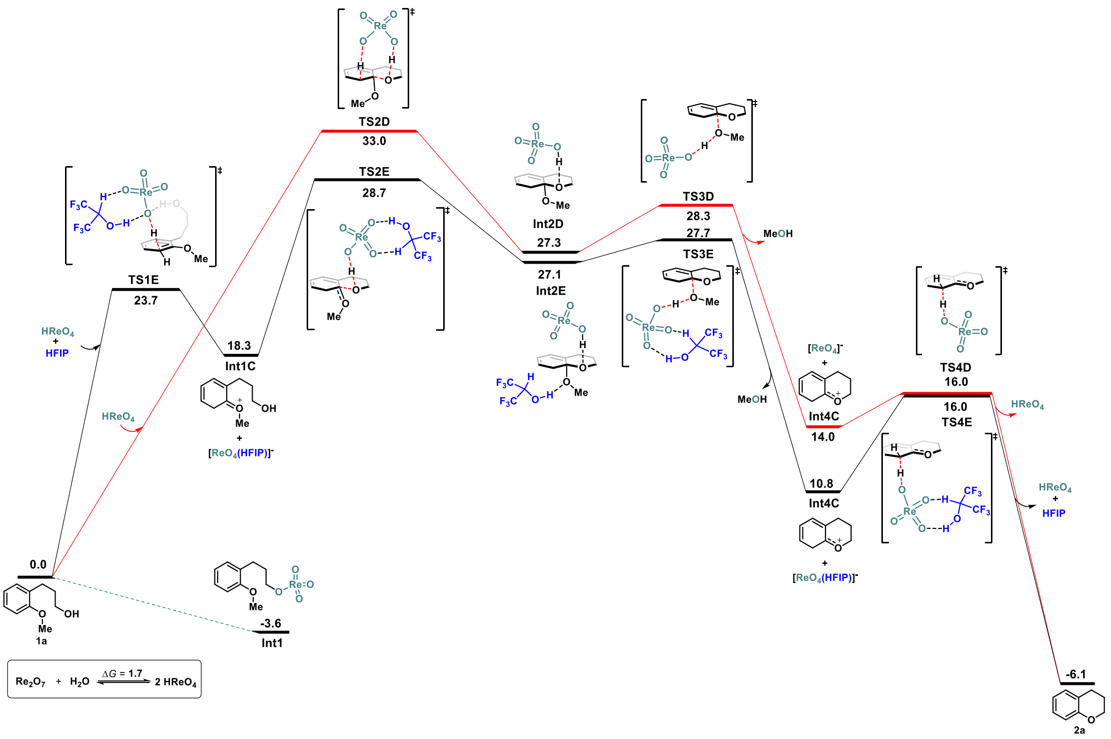

Supplementary Figure 21. Gibbs free energy profile (kcal/mol) of pathway d without HFIP (red line) and with one HFIP (black line) at the SMD-B3LYP-D3/def2-TZVPP//B3LYP-D3/def2-SVP-SDD(Re) level for the ring-closing C-O/C-O σ-bond metathesis of 1a catalyzed with Re_2O_7.

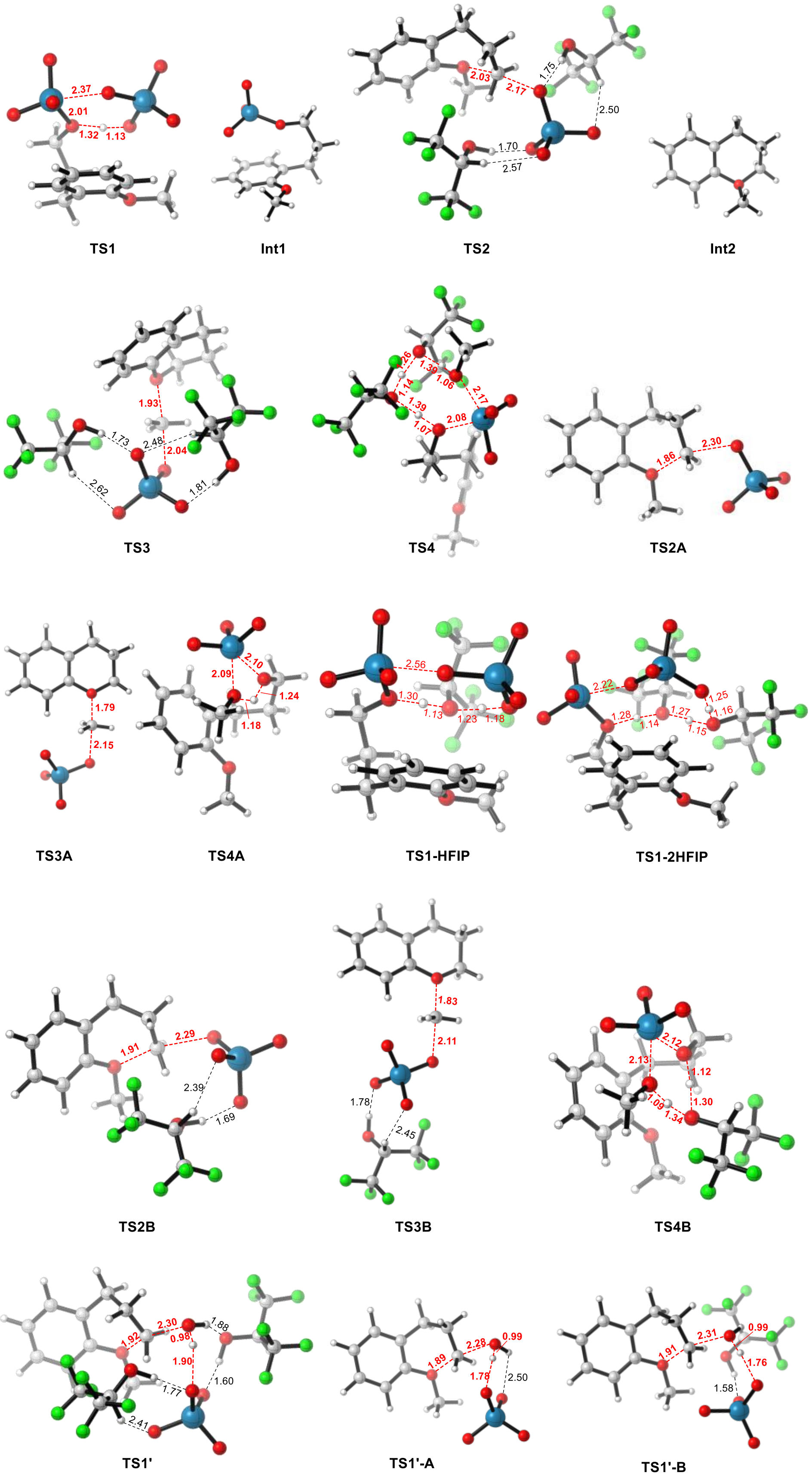

Supplementary Figure 22. Optimized geometries of the critical intermediates and transition states. All distances are given in Å.

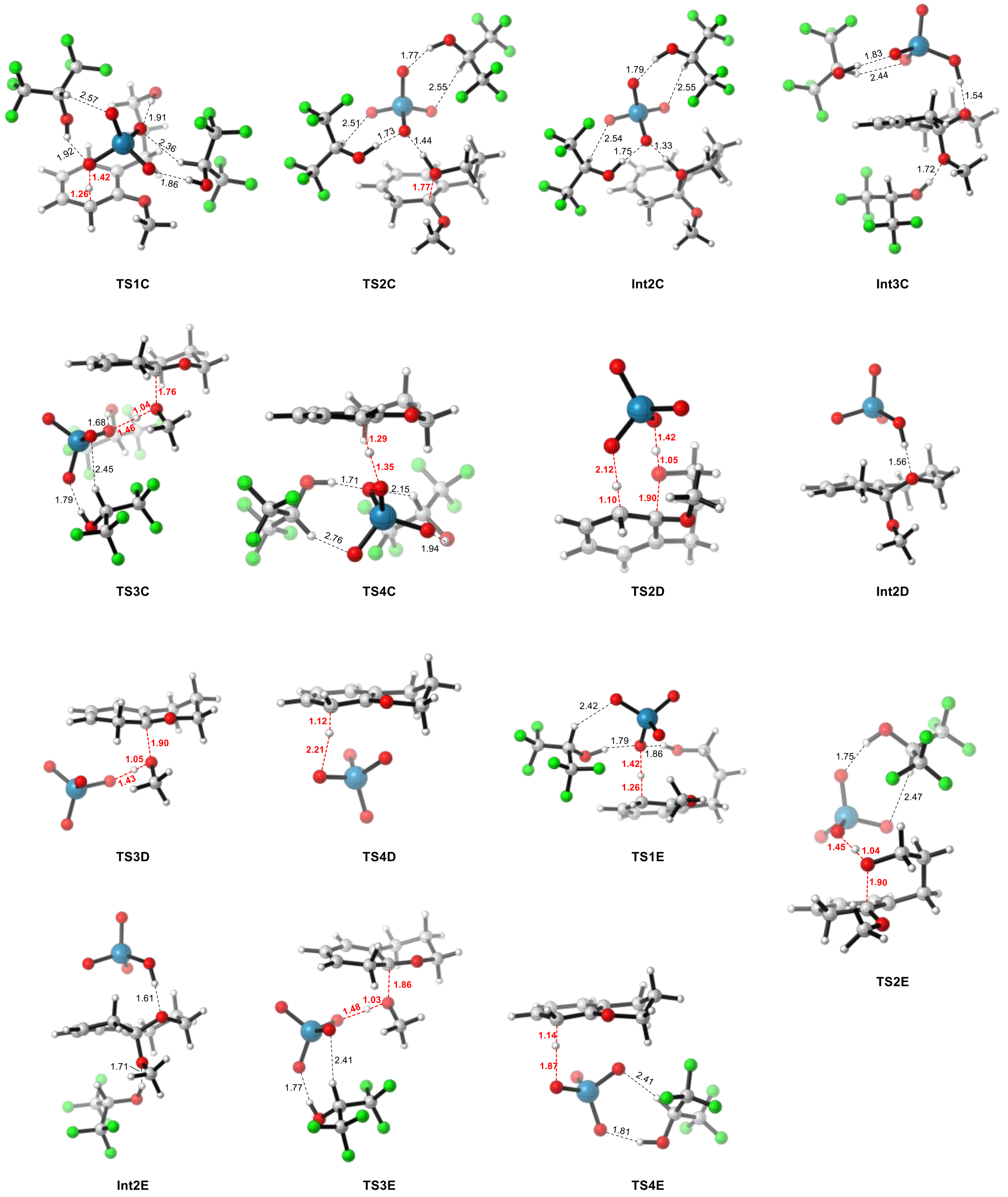

Supplementary Figure 23. Optimized geometries of the critical intermediates and transition states. All distances are given in Å.

Additionally, DFT calculations have been performed regarding the radical mechanism of **1a**. As depicted in Supplementary Figure 24, the direct homolytic dissociation of the OH bond in **1a** (eq. 1) requires a free energy penalty of 95.0 kcal/mol, highly impossible to take place under the given experimental condition. Moreover, different homolytic dissociation manners are accounted for the perrhenate ester **Int1**. As for the homolytic cleavage of bond a (C-O bond, eq. 2), the calculated free energy is 86.3 kcal/mol, while the dissociation of bond b (Re-O bond, eq. 3) is endergonic by 54.2 kcal/mol; both are lower than the O-H bond dissociation energy in eq. 1, but are still too high to overcome. In addition, the free energy for the C-O bond cleavage to form oxyl radical **A**, which automatically optimized to complex **B**, is also as high as 84.1 kcal/mol (eq. 4). Taken together, the involvement of radical intermediates is ruled out.

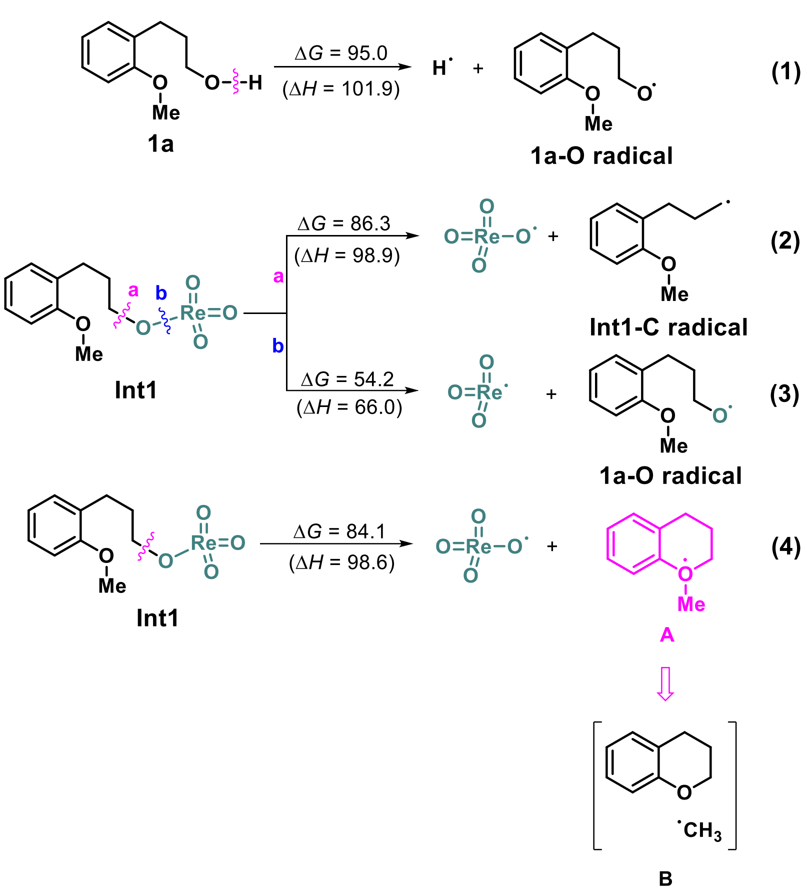

Supplementary Figure 24. Calculated results for the formation of radical intermediates at the SMD-B3LYP-D3/def2-TZVPP//B3LYP-D3/def2-SVP-SDD(Re) level.

The activation of Re_2O_7 by the solvent HFIP is also calculated (See **TS1F** and **TS1G** in Supplementary Figure 25) but was found to be less feasible than the esterification process with **1a** (via **TS1** in Fig. 5) to generate the perrhenate ester **Int1** (Fig. 5).

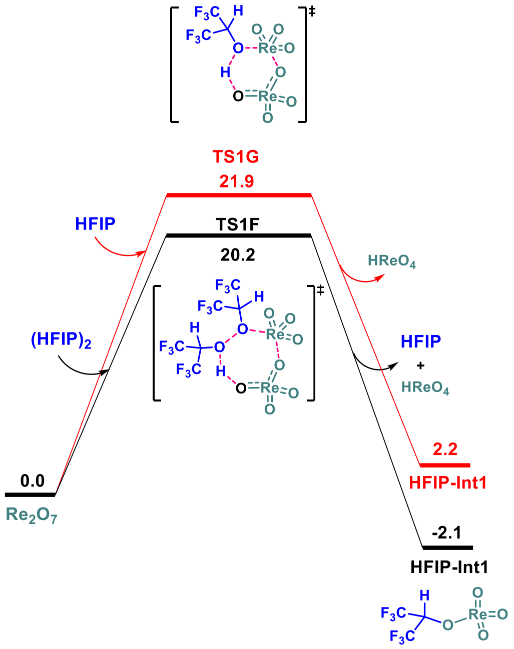

Supplementary Figure 25. Gibbs free energy profile (kcal/mol) at the SMD-B3LYP-D3/def2-TZVPP//B3LYP-D3/def2-SVP-SDD(Re) level for the activation of Re_2O_7 by the solvent HFIP.

As shown in Supplementary Figure 26, DFT calculations on the mechanism of the ring-closing C–O/C–O σ-bond metathesis of **1r** were carried out as well. Various reaction pathways (pathways a, b, and c) proposed in Fig. 3 in the main text were tested theoretically. Based on the calculated results, the most favorable reaction pathway for substrate **1r** is still pathway a, i.e., the perrhenate ester formation mechanism, while the Brønsted acid catalysis mechanism (pathway b) and the ether activation mechanism (pathway c) are found to be 2.1 kcal/mol and 5.8 kcal/mol less favored than pathway a, respectively.

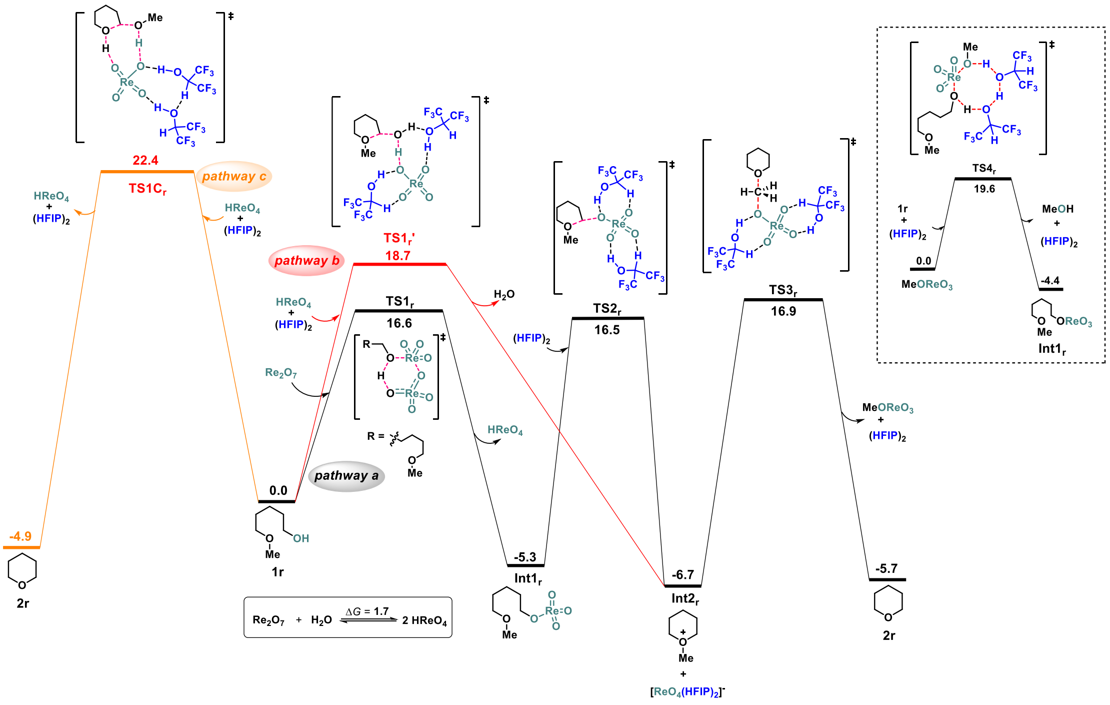

Supplementary Figure 26. Gibbs free energy profile (kcal/mol) at the SMD-B3LYP-D3/def2-TZVPP//B3LYP-D3/def2-SVP-SDD(Re) level for the ring-closing C–O/C–O σ-bond metathesis of 1r catalyzed with Re_2O_7.

4.3 Calculated energies (in hartree) for all stationary points

**Stationary point**	**Thermal correction to Gibbs free energy**	**Single-point energy (SMD)-B3LYP-D3/def2-TZVPP**
Re_2O_7	-0.018101	-683.8422348
$HReO_4$	-0.008233	-380.1576351
$(HFIP)_2$	0.074988	-1580.365915
$[ReO_4(HFIP)_2]^{\ominus}$	0.074153	-1960.110892
$[ReO_4(HFIP)]^{\ominus}$	0.028	-1169.915044
1a	0.186508	-540.185614
TS1	0.192447	-1224.022426
Int1	0.176267	-843.8756546
TS2	0.273916	-2424.223732
Int2	0.175383	-464.115152
TS3	0.272675	-2424.21977
2a	0.137369	-424.391373
$MeOReO_3$	0.017456	-419.4710898
TS4	0.32182	-2540.025166
MeOH	0.028269	-115.7861397
TS2A	0.175573	-843.8310378
TS3A	0.175679	-843.8275229
TS4A	0.223566	-959.6273271
TS1-HFIP	0.240008	-2014.214464
TS1-2HFIP	0.290695	-2804.402877
TS2B	0.226749	-1634.0306
TS3B	0.22166	-1634.02123

TS4B	0.276608	-1749.837651
TS1’	0.297077	-2500.712257
TS1’-A	0.199838	-920.3170477
TS1’-B	0.248651	-1710.512352
TS1C	0.296841	-2500.719531
Int1C	0.194543	-540.592658
TS2C	0.299079	-2500.710057
Int2C	0.297975	-2500.7098
Int3C	0.299857	-2500.710939
TS3C	0.296642	-2500.712763
Int4C	0.147603	-424.8028886
TS4C	0.244132	-2384.926305
TS2D	0.200205	-920.3122456
Int2D	0.203132	-920.3242027
TS3D	0.200572	-920.3200854
TS4D	0.152598	-804.536871
TS1E	0.244444	-1710.517982
TS2E	0.249664	-1710.515233
Int2E	0.251849	-1710.519922
TS3E	0.248999	-1710.516213
TS4E	0.199916	-1594.730986
1a-O radical	0.170391	-539.5084852
Int1-C radical	0.165359	-464.253613
ReO_4 radical	-0.020535	-379.4561475

ReO_3 radical	-0.021024	-304.2569565
B	0.161009	-464.2527459
TS1G	0.027938	-1474.000005
TS1F	0.080128	-2264.194768
HFIP-Int1	0.017022	-1093.857717
1r	0.161844	-387.6909943
2r	0.117498	-271.8996014
$TS1_r$	0.163577	-1071.523526
$Int1_r$	0.152192	-691.38432
$TS2_r$	0.252177	-2271.736018
$Int2_r$	0.155841	-311.6429483
$TS3_r$	0.251213	-2271.734349
$TS4_r$	0.296434	-2387.531724
$TS1_r'$	0.276336	-2348.227697
$TS1C_r$	0.276788	-2348.222189

5. Supplementary Note 3

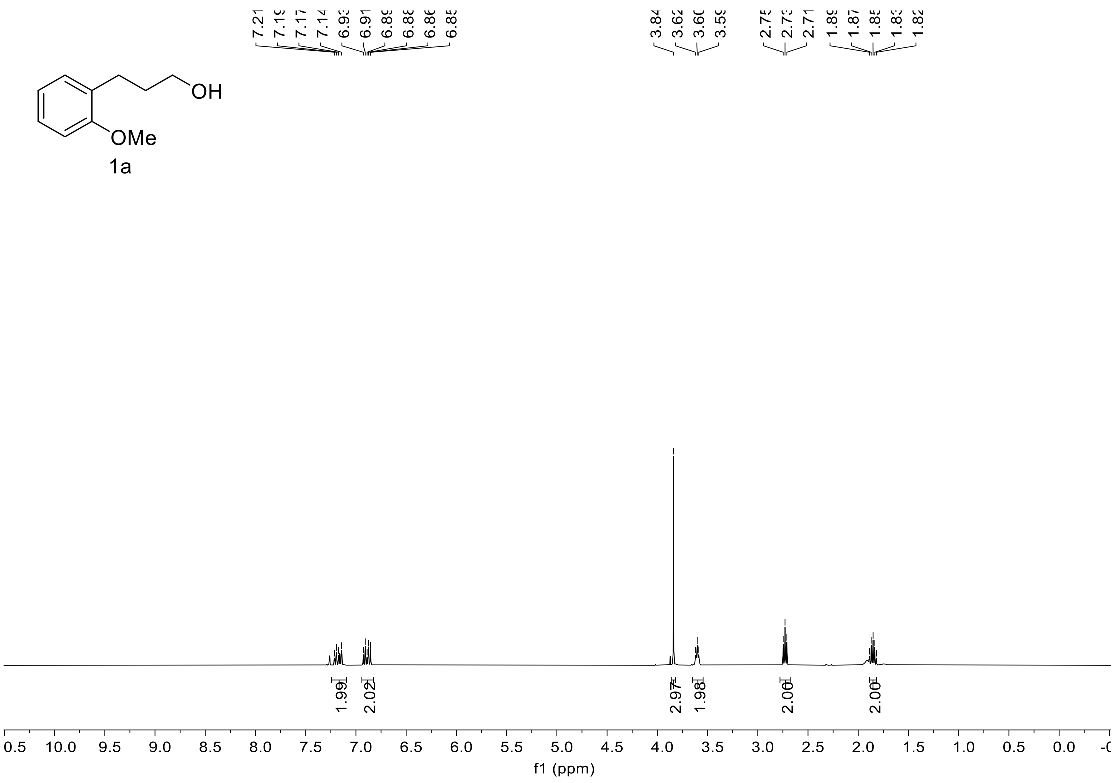

Supplementary Figure 27. ^{1}H NMR of compound **1a** (400 MHz, $CDCl_3$)

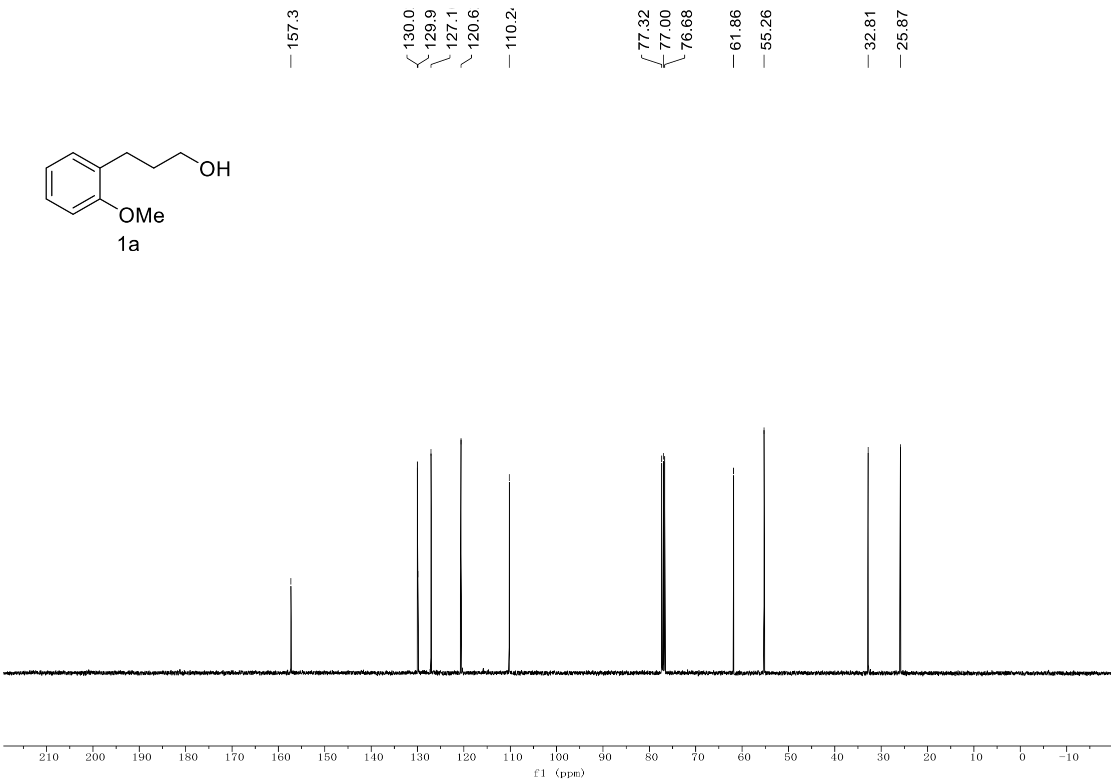

Supplementary Figure 28. ^{13}C NMR of compound **1a** (101 MHz, $CDCl_3$)

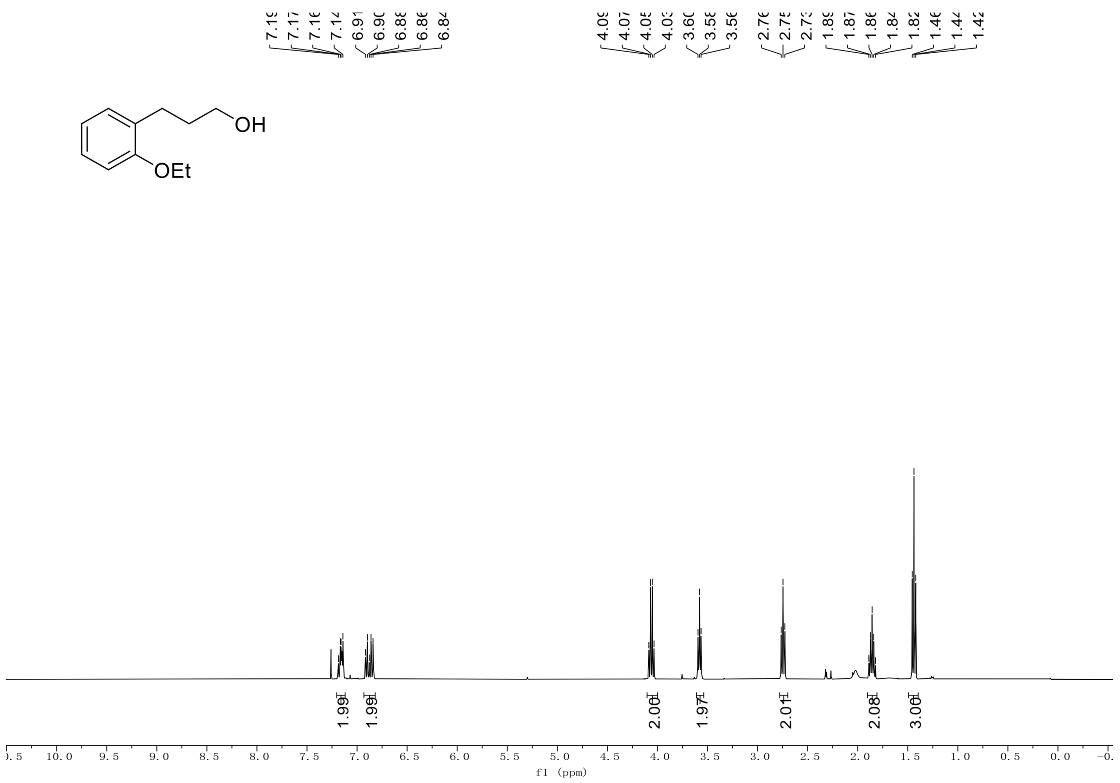

Supplementary Figure 29. ^{1}H NMR of compound **1a with Et** (400 MHz, $CDCl_3$)

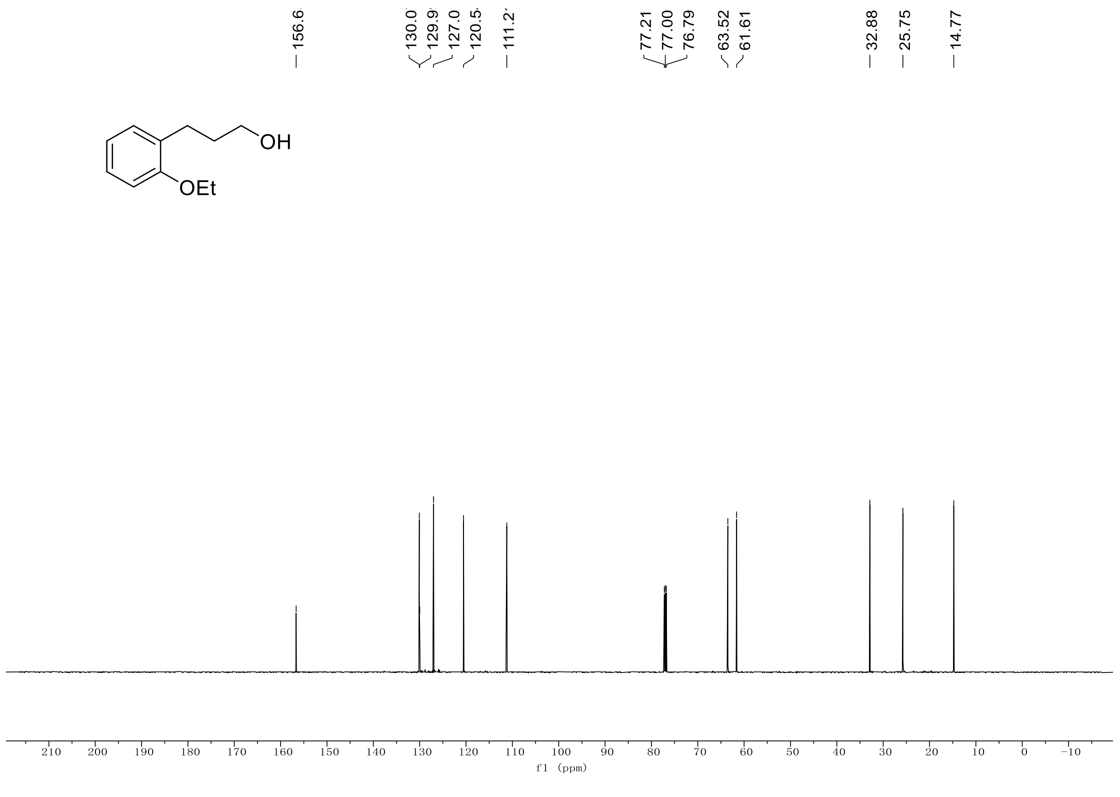

Supplementary Figure 30. ^{13}C NMR of compound **1a with Et** (151 MHz, $CDCl_3$)

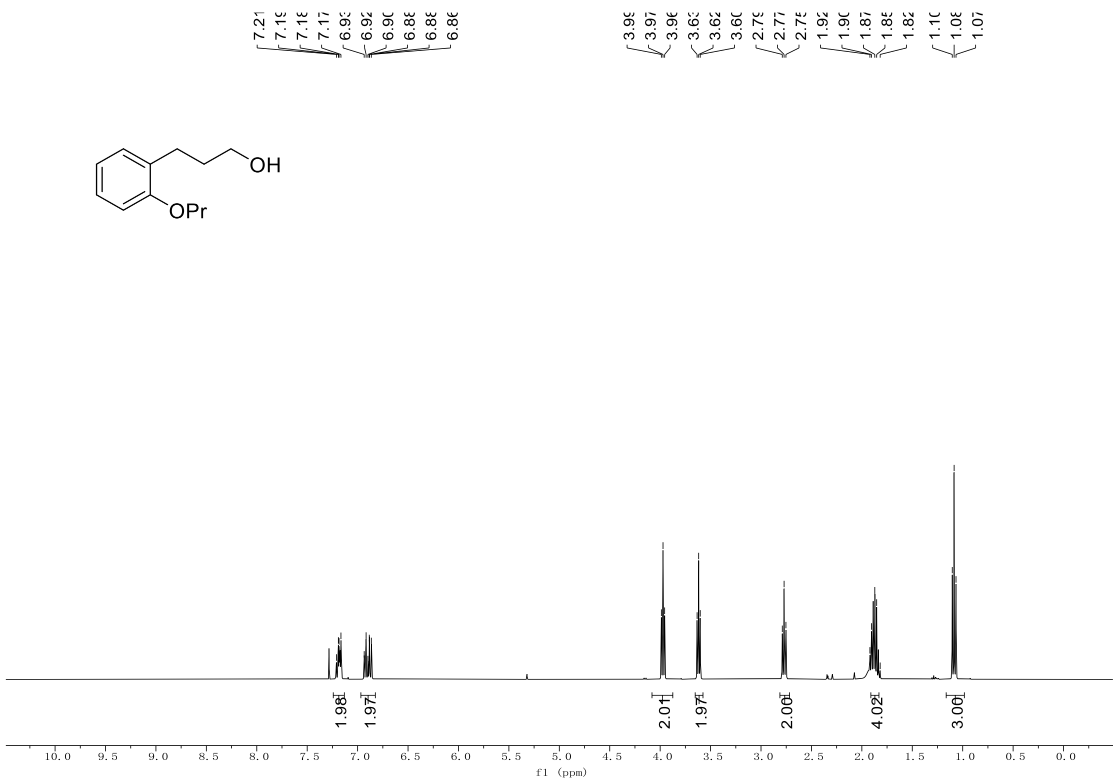

Supplementary Figure 31. ^{1}H NMR of compound **1a with Pr** (400 MHz, $CDCl_3$)

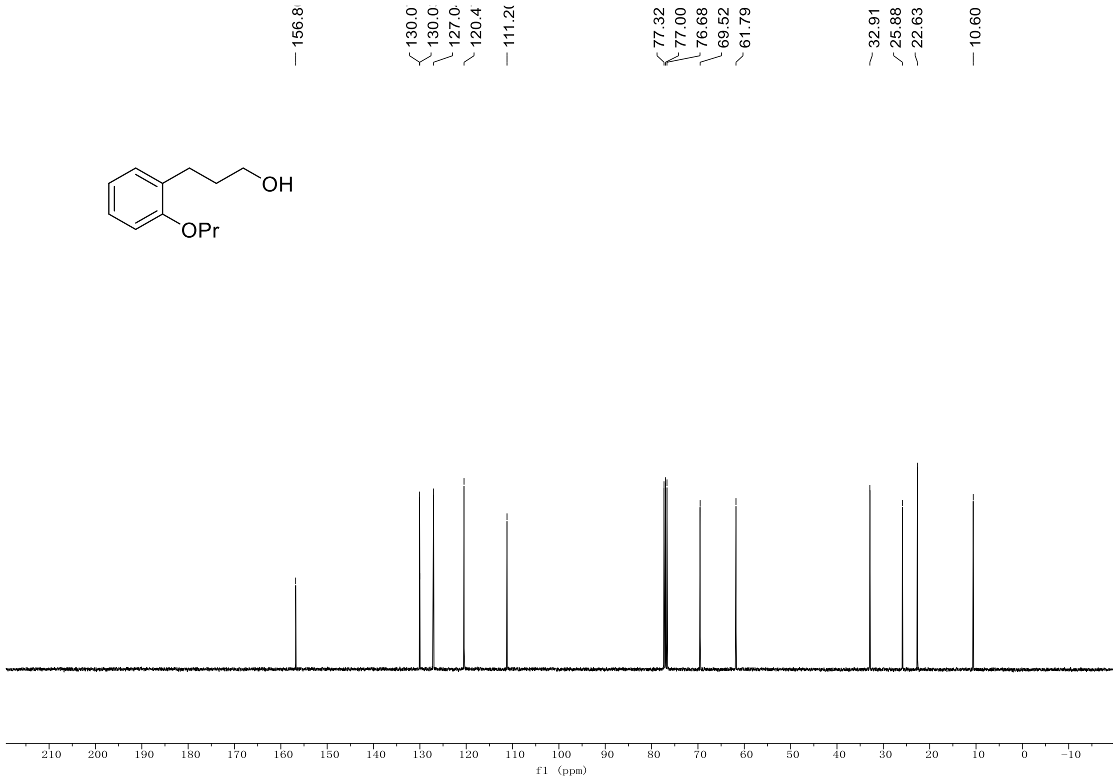

Supplementary Figure 32. ^{13}C NMR of compound **1a with Pr** (101 MHz, $CDCl_3$)

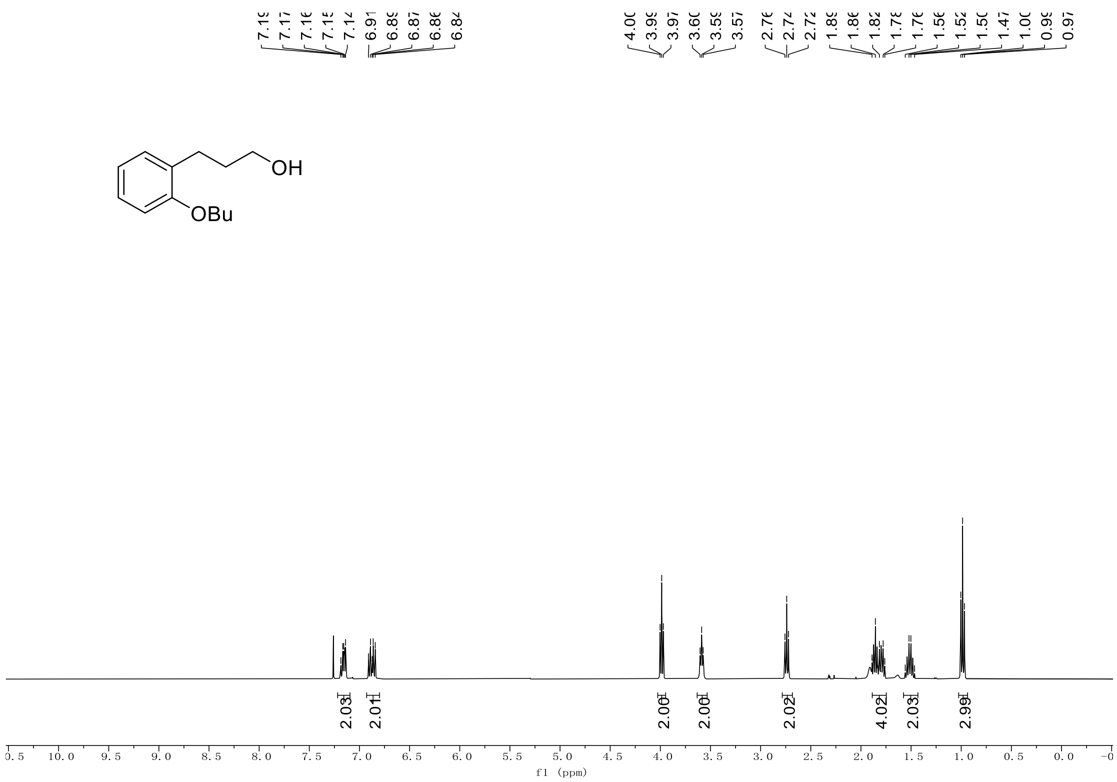

Supplementary Figure 33. ^{1}H NMR of compound **1a with Bu** (400 MHz, $CDCl_3$)

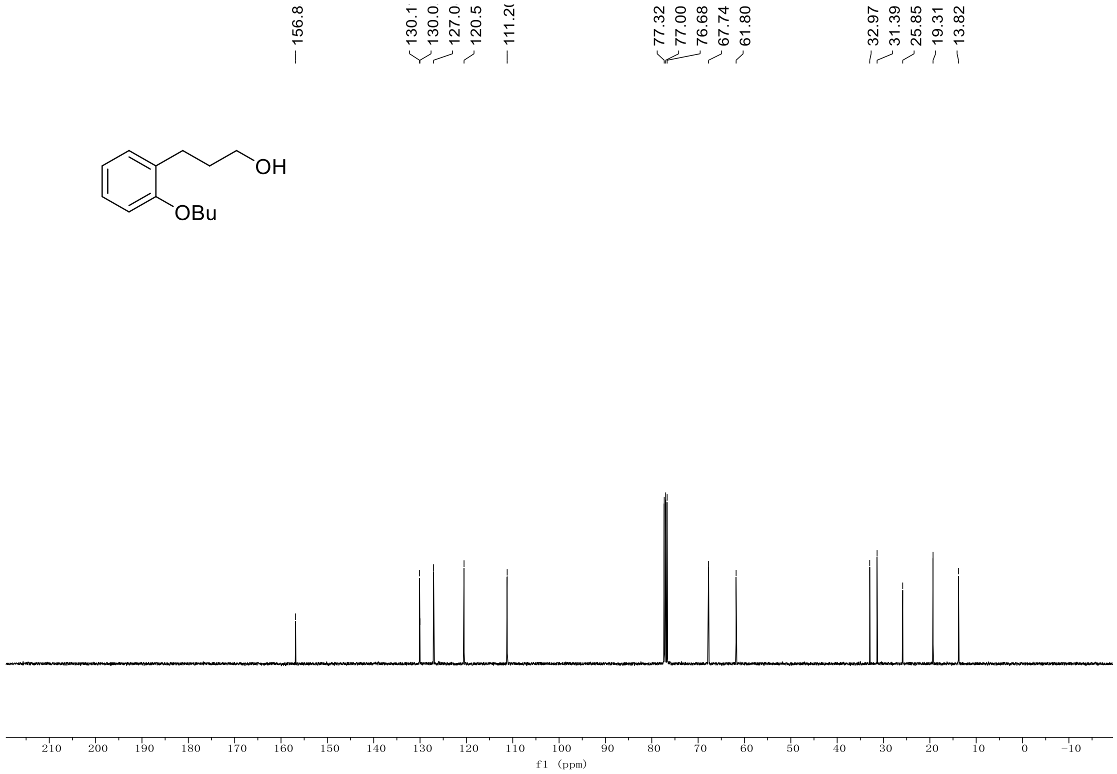

Supplementary Figure 34. ^{13}C NMR of compound **1a with Bu** (101 MHz, $CDCl_3$)

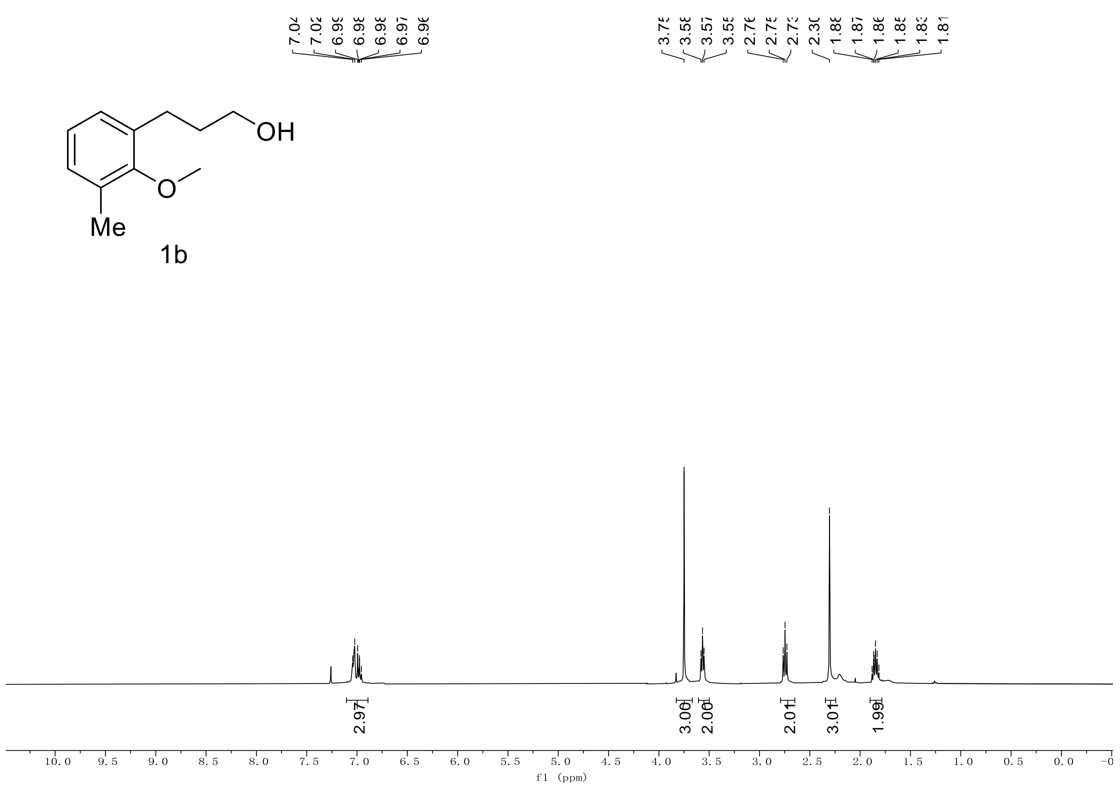

Supplementary Figure 35. ^{1}H NMR of compound **1b** (400 MHz, $CDCl_3$)

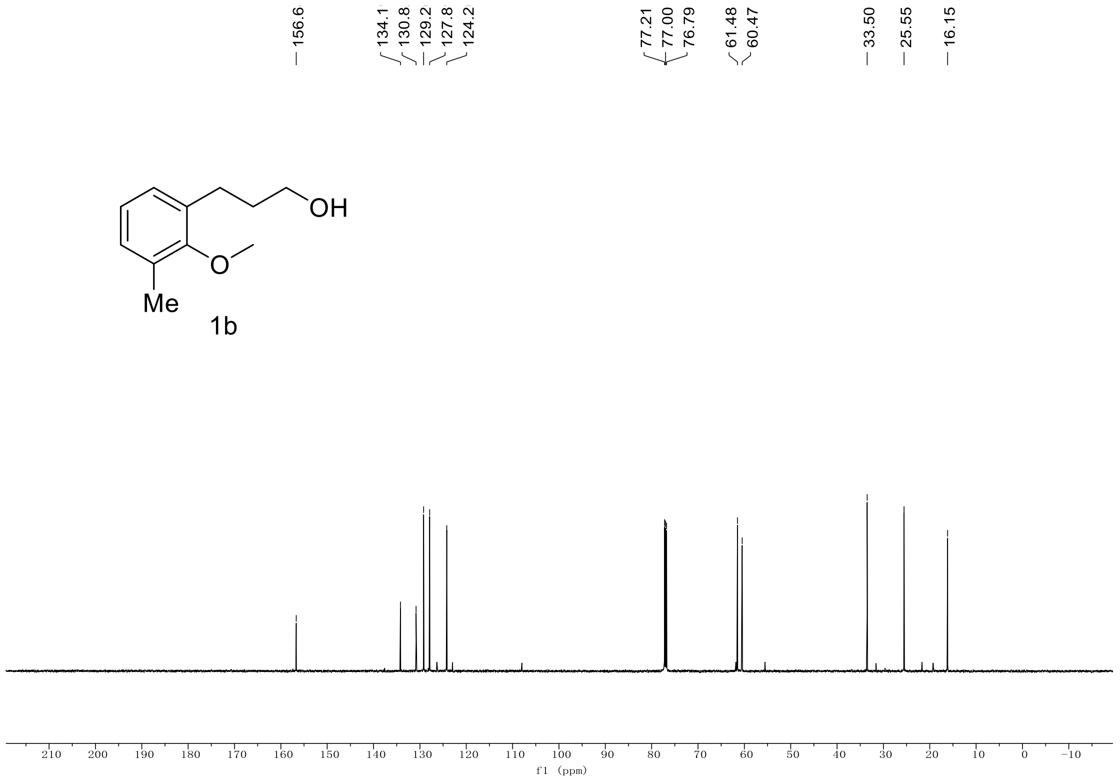

Supplementary Figure 36. ^{13}C NMR of compound **1b** (101 MHz, $CDCl_3$)

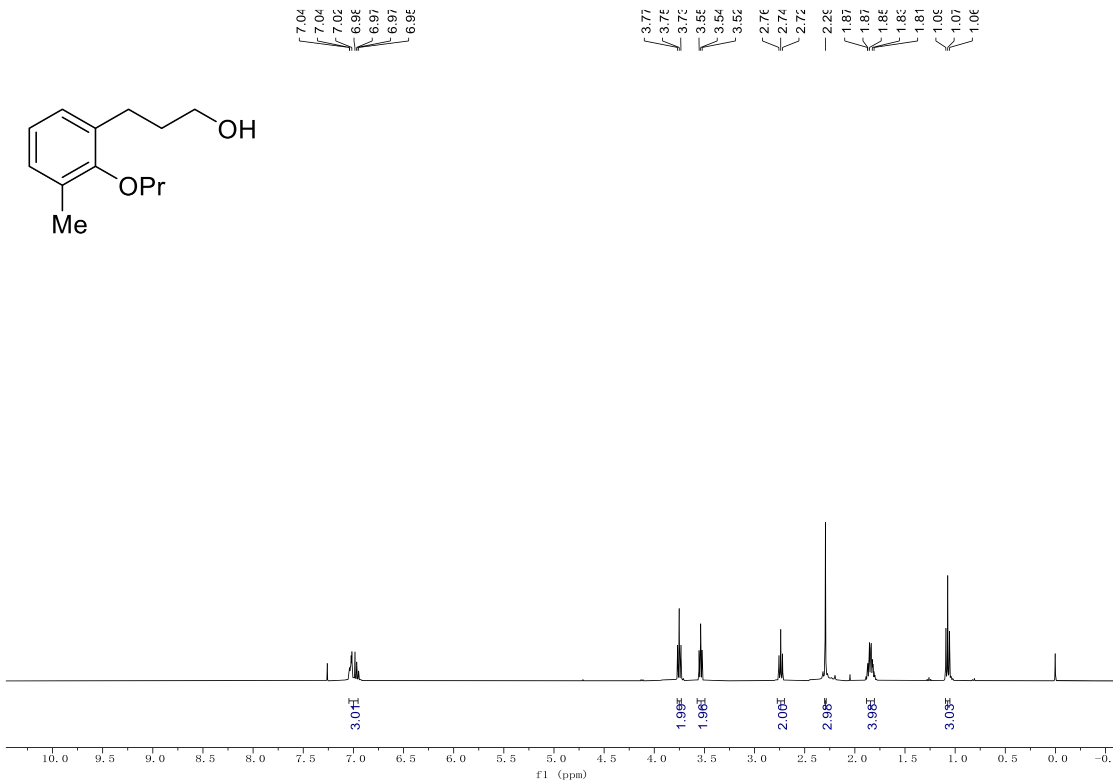

Supplementary Figure 37. ^{1}H NMR of compound **1b with Pr** (400 MHz, $CDCl_3$)

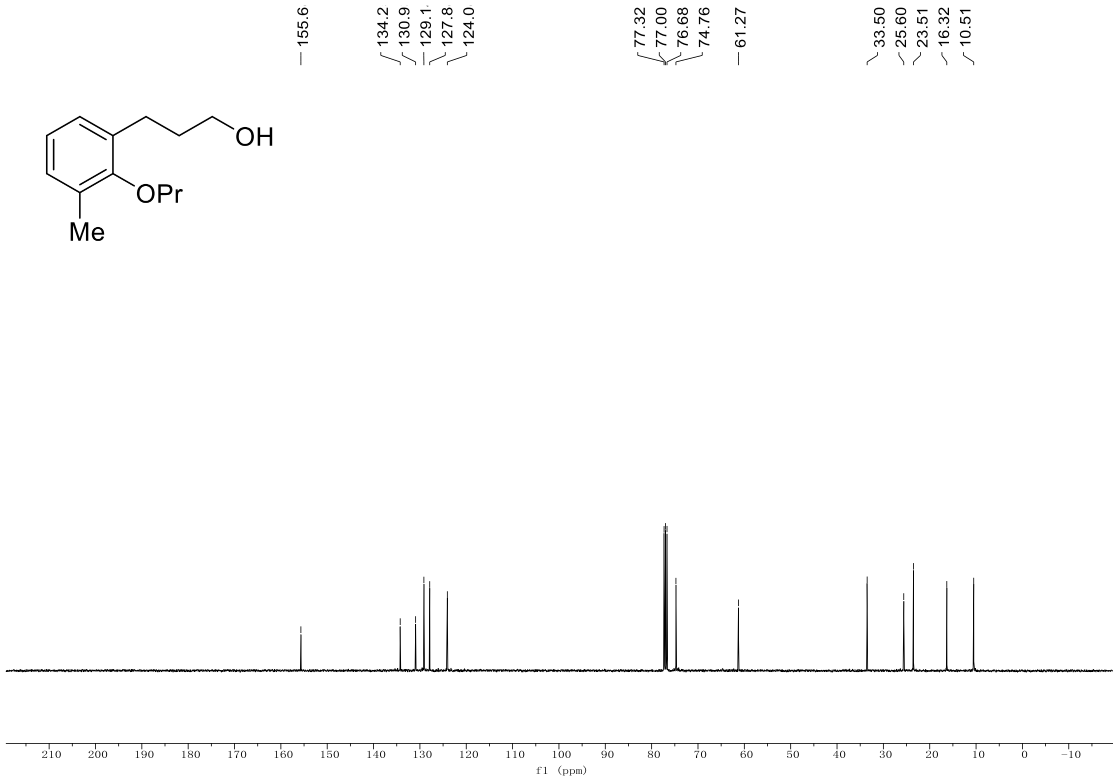

Supplementary Figure 38. ^{13}C NMR of compound **1b with Pr** (101 MHz, $CDCl_3$)

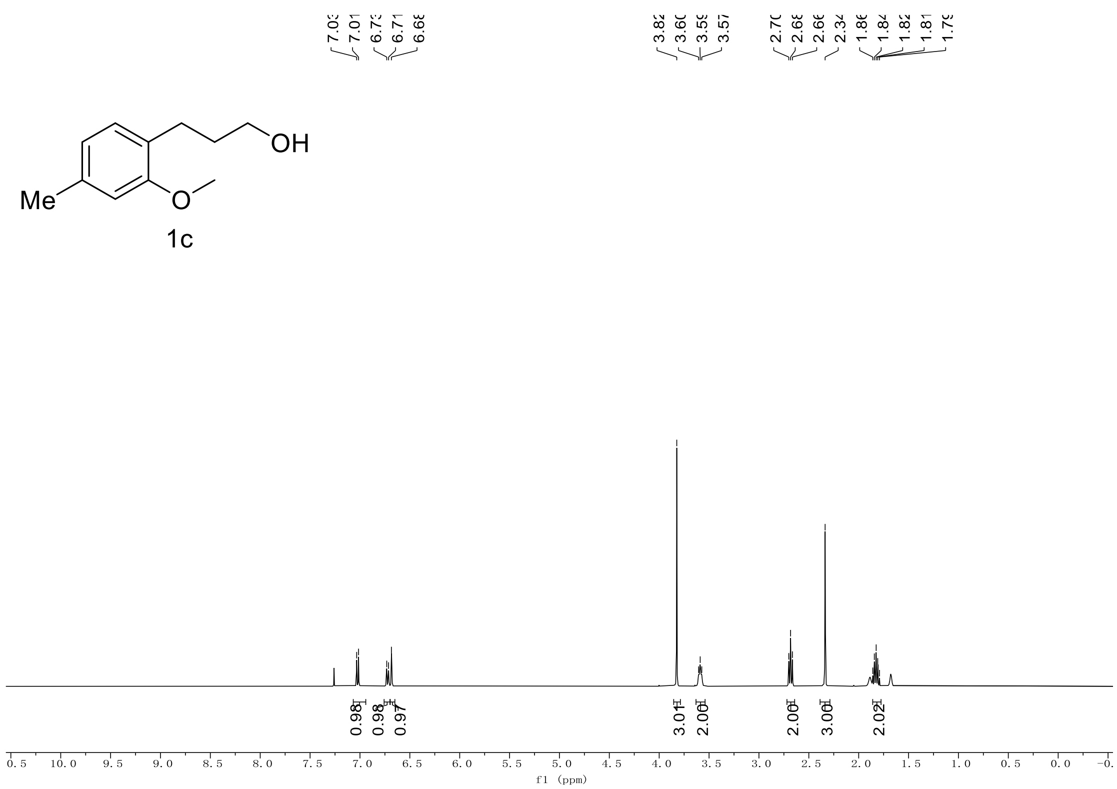

Supplementary Figure 39. ^{1}H NMR of compound **1c** (400 MHz, $CDCl_3$)

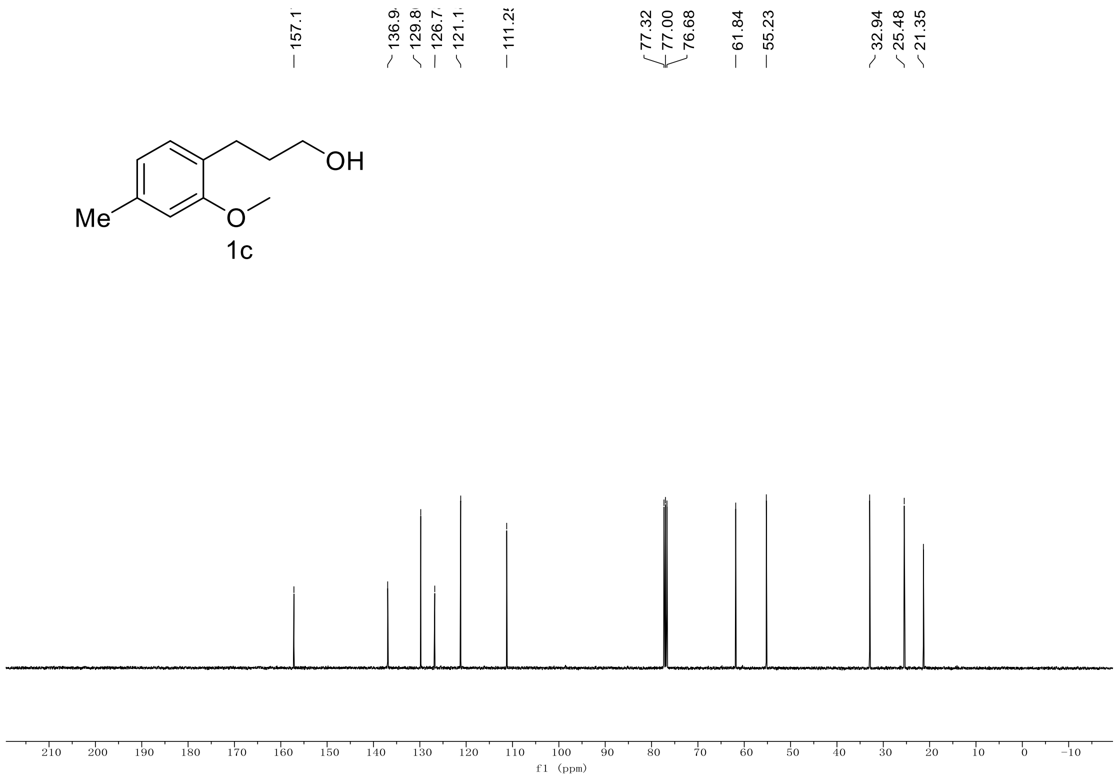

Supplementary Figure 40. ^{13}C NMR of compound **1c** (101 MHz, $CDCl_3$)

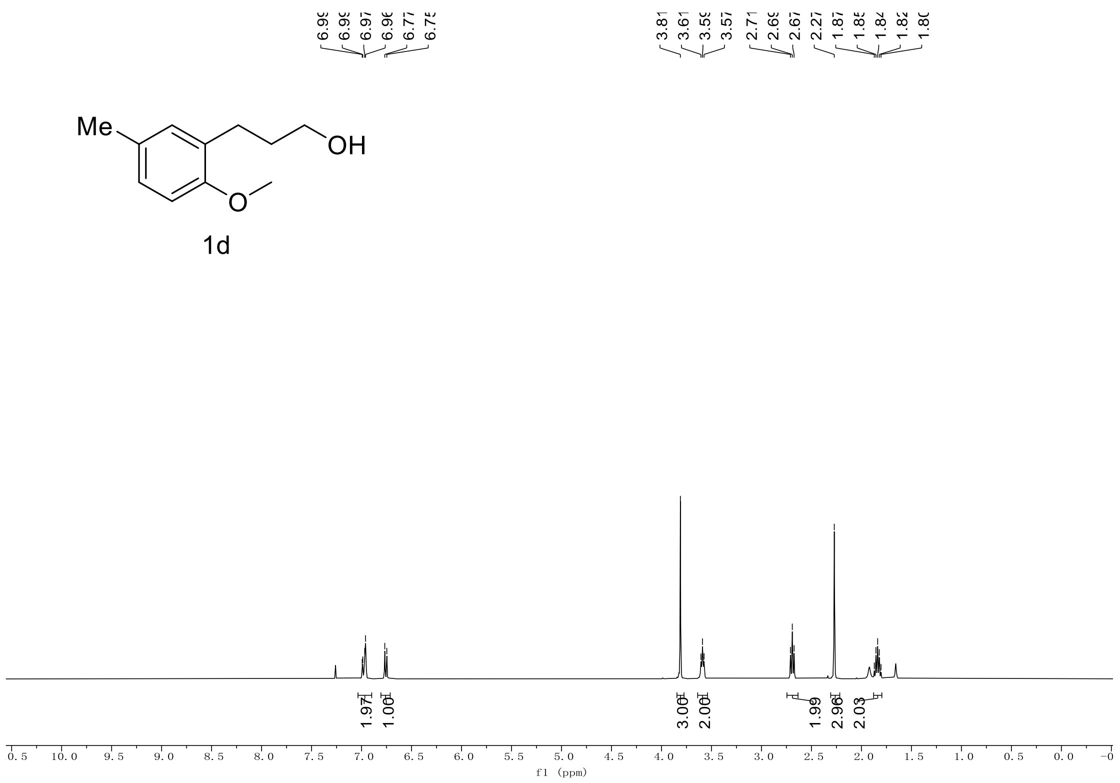

Supplementary Figure 41. ^{1}H NMR of compound **1d** (400 MHz, $CDCl_3$)

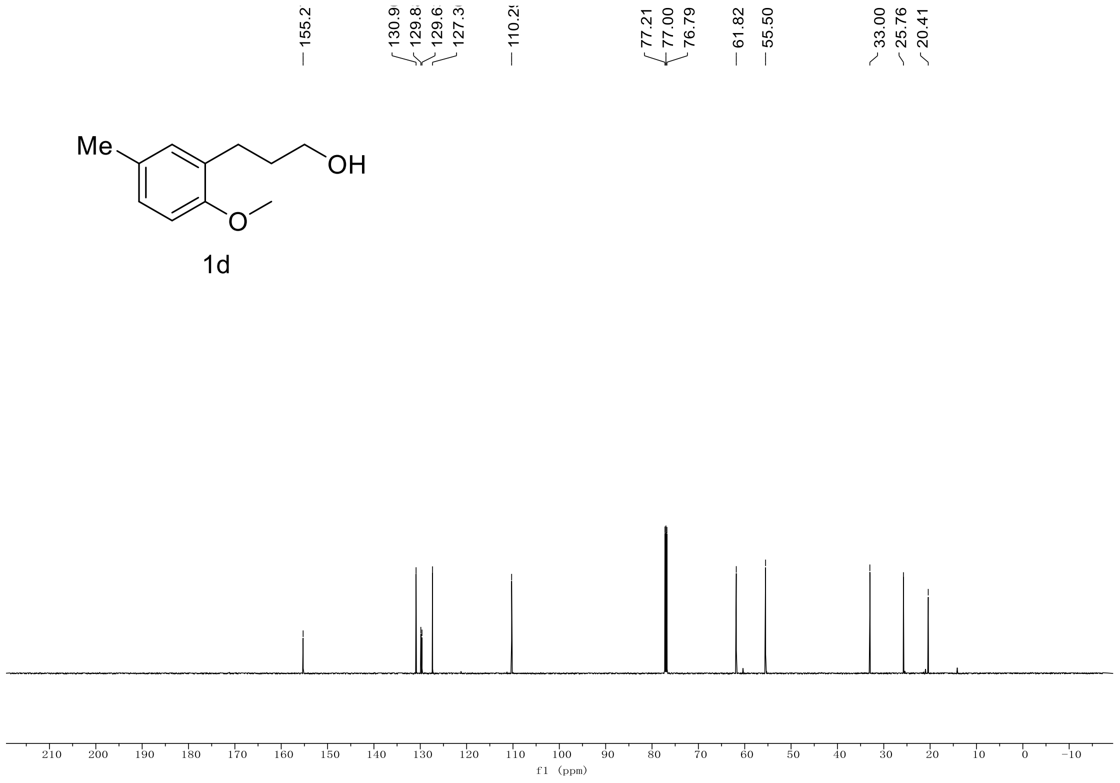

Supplementary Figure 42. ^{13}C NMR of compound **1d** (151 MHz, $CDCl_3$)

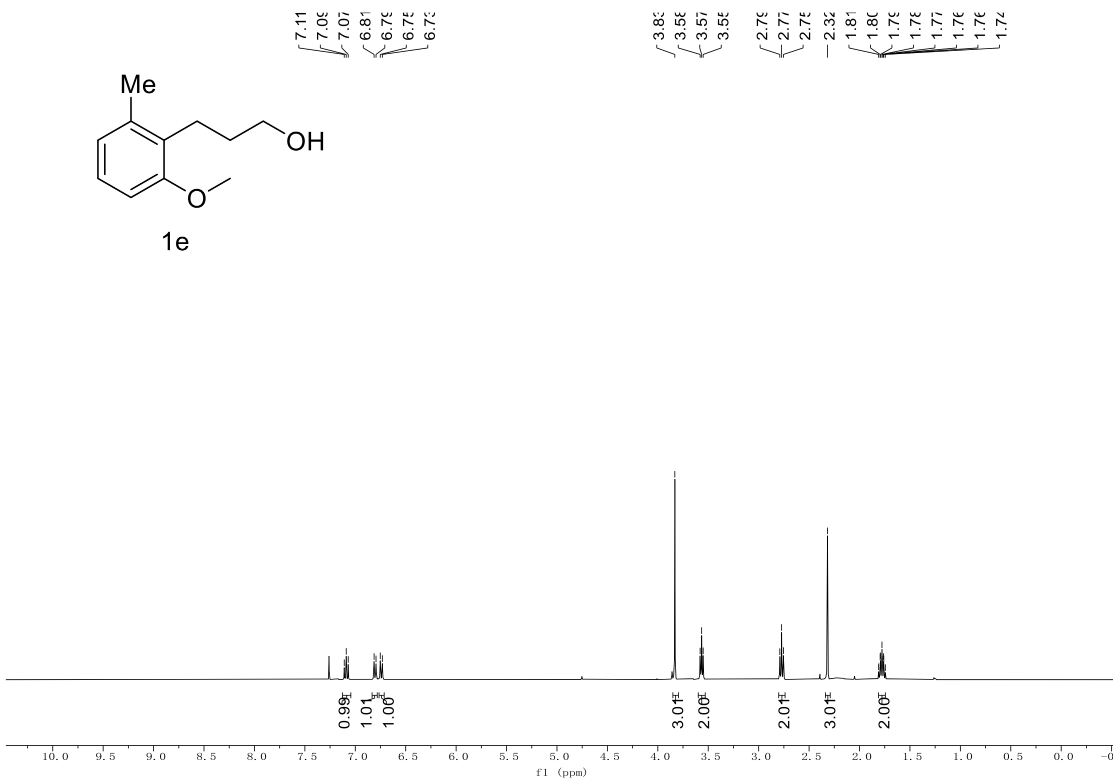

Supplementary Figure 43. ^{1}H NMR of compound **1e** (400 MHz, $CDCl_3$)

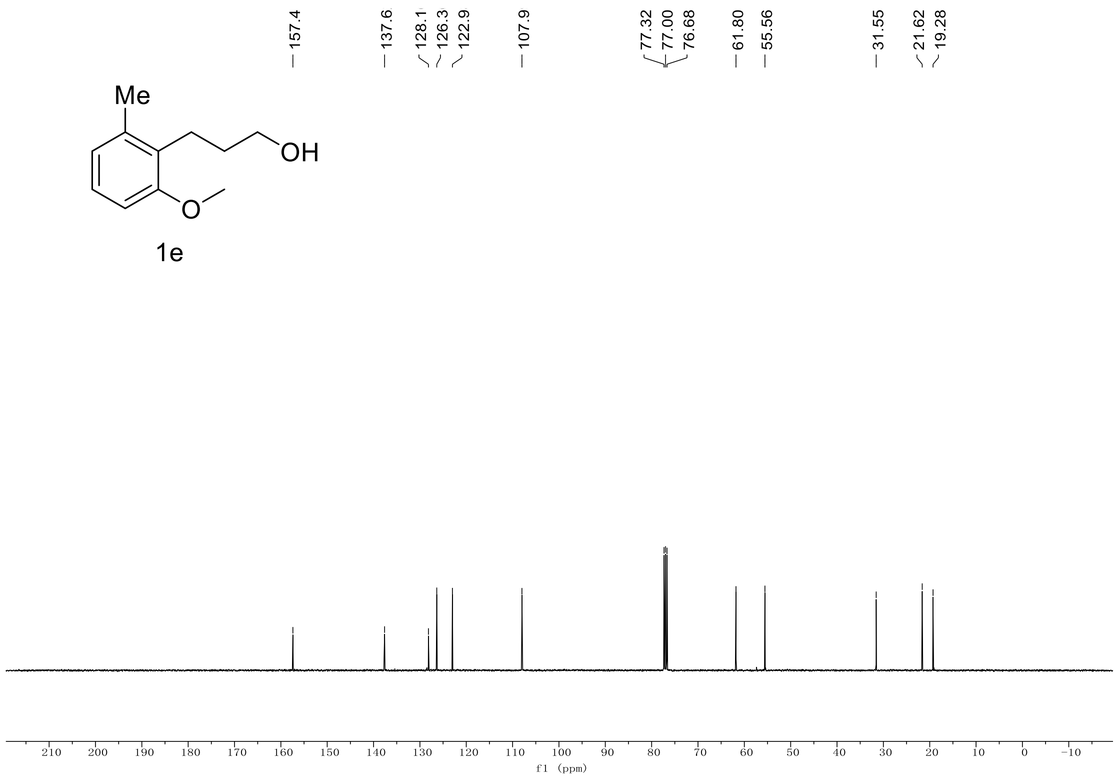

Supplementary Figure 44. ^{13}C NMR of compound **1e** (101 MHz, $CDCl_3$)

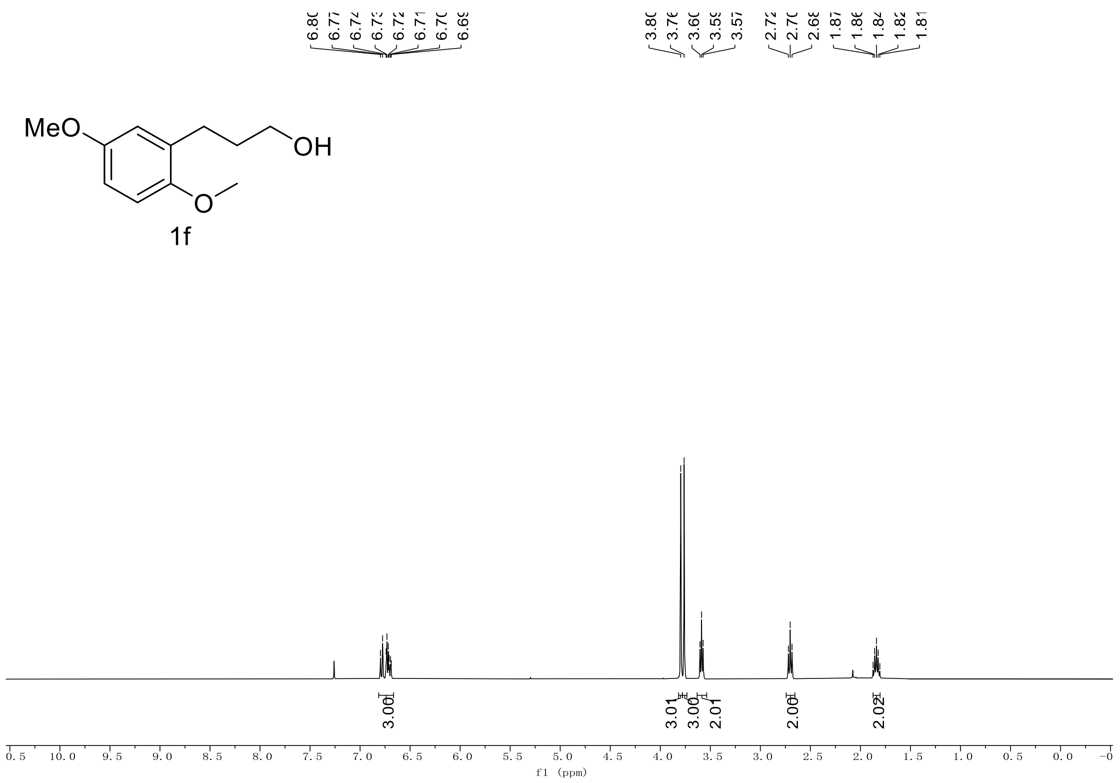

Supplementary Figure 45. ^{1}H NMR of compound **1f** (400 MHz, $CDCl_3$)

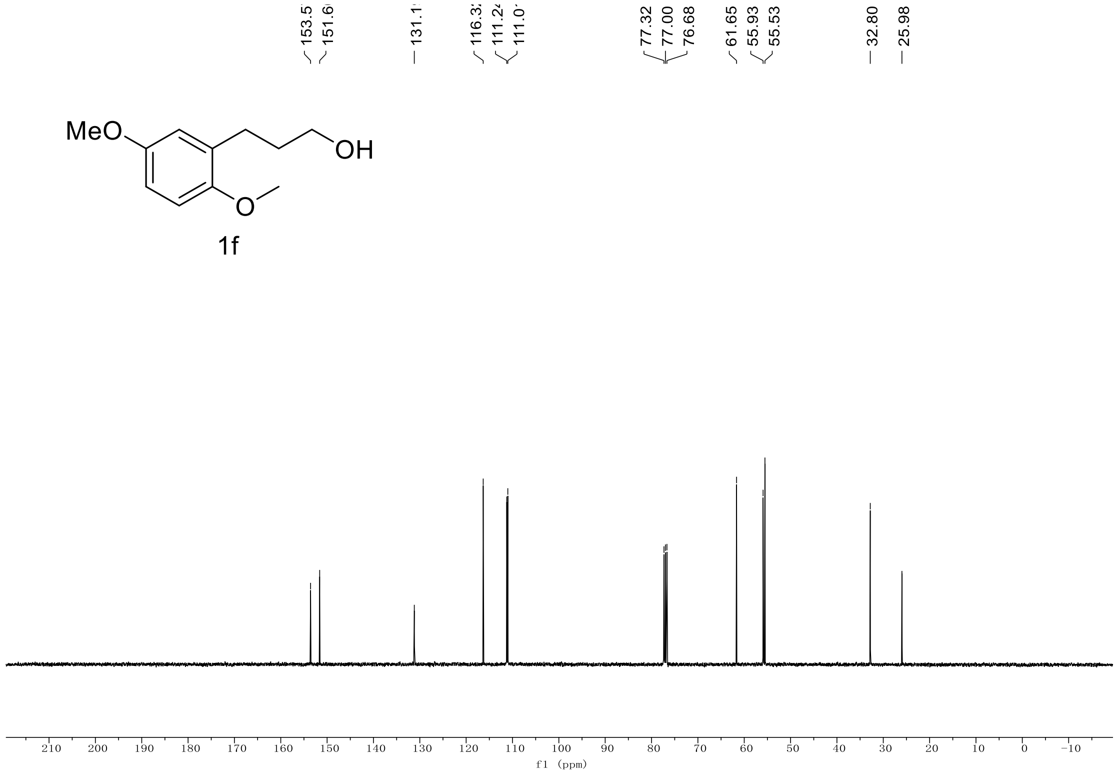

Supplementary Figure 46. ^{13}C NMR of compound **1f** (101 MHz, $CDCl_3$)

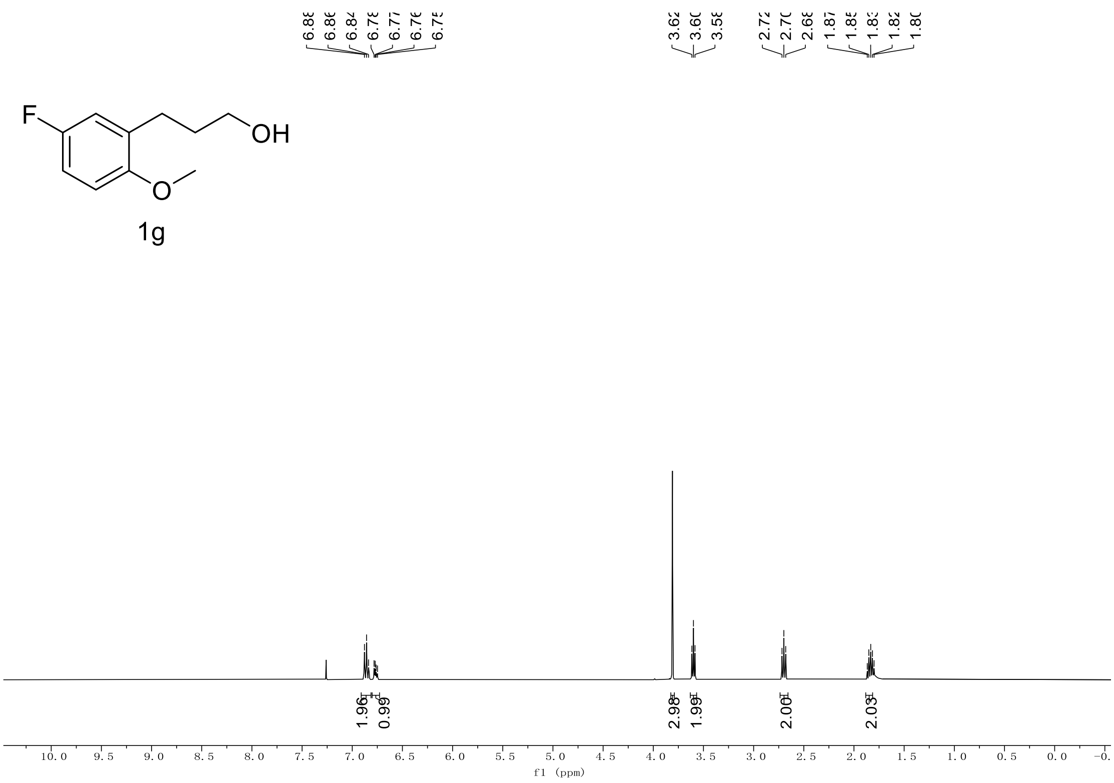

Supplementary Figure 47. ^{1}H NMR of compound **1g** (400 MHz, $CDCl_3$)

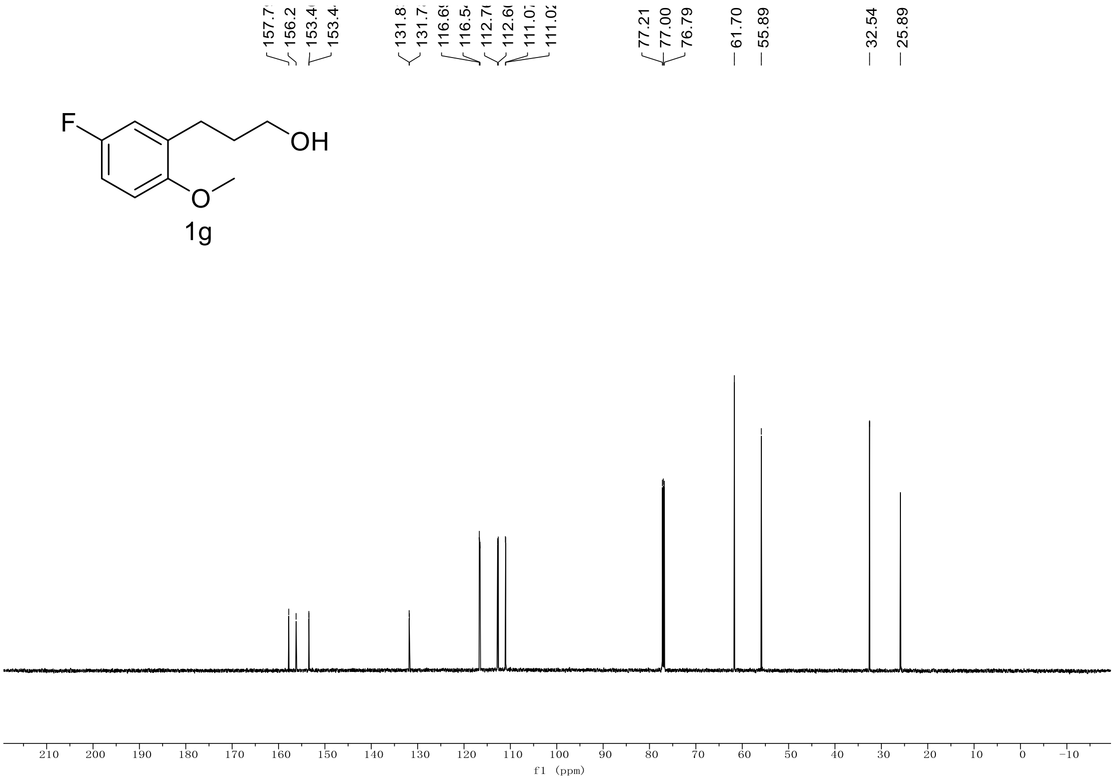

Supplementary Figure 48. ^{13}C NMR of compound **1g** (151 MHz, $CDCl_3$)

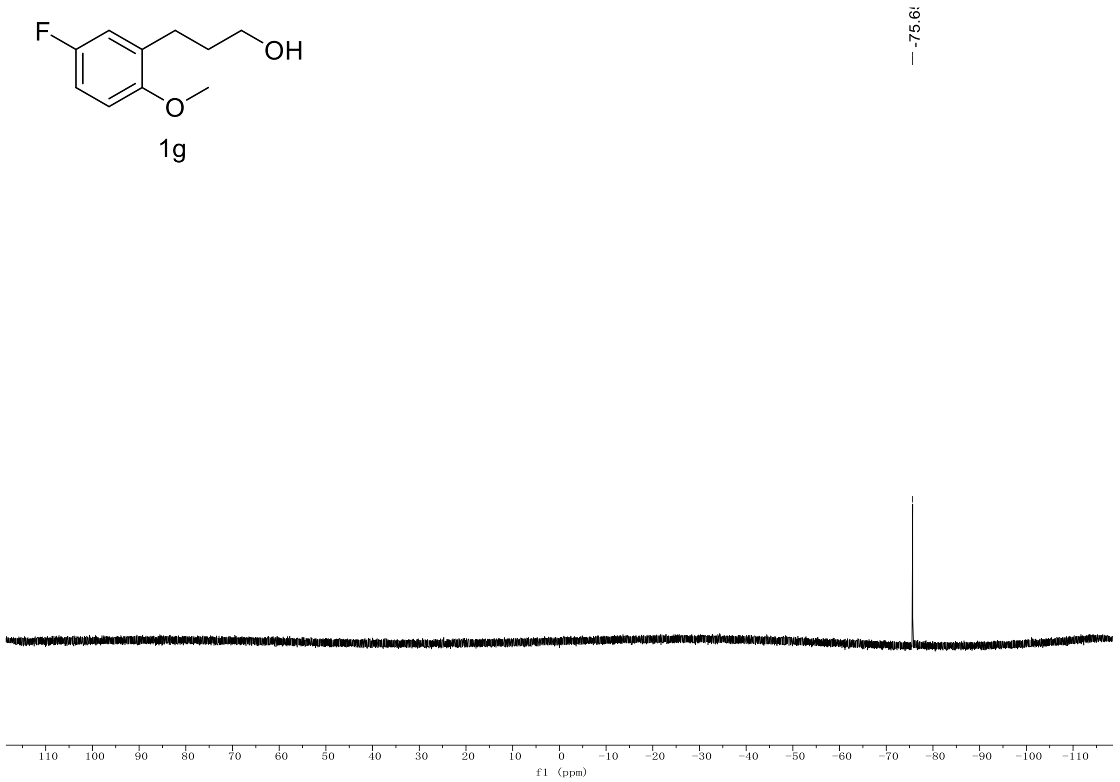

Supplementary Figure 49. ^{19}F NMR of compound **1g** (565 MHz, $CDCl_3$)

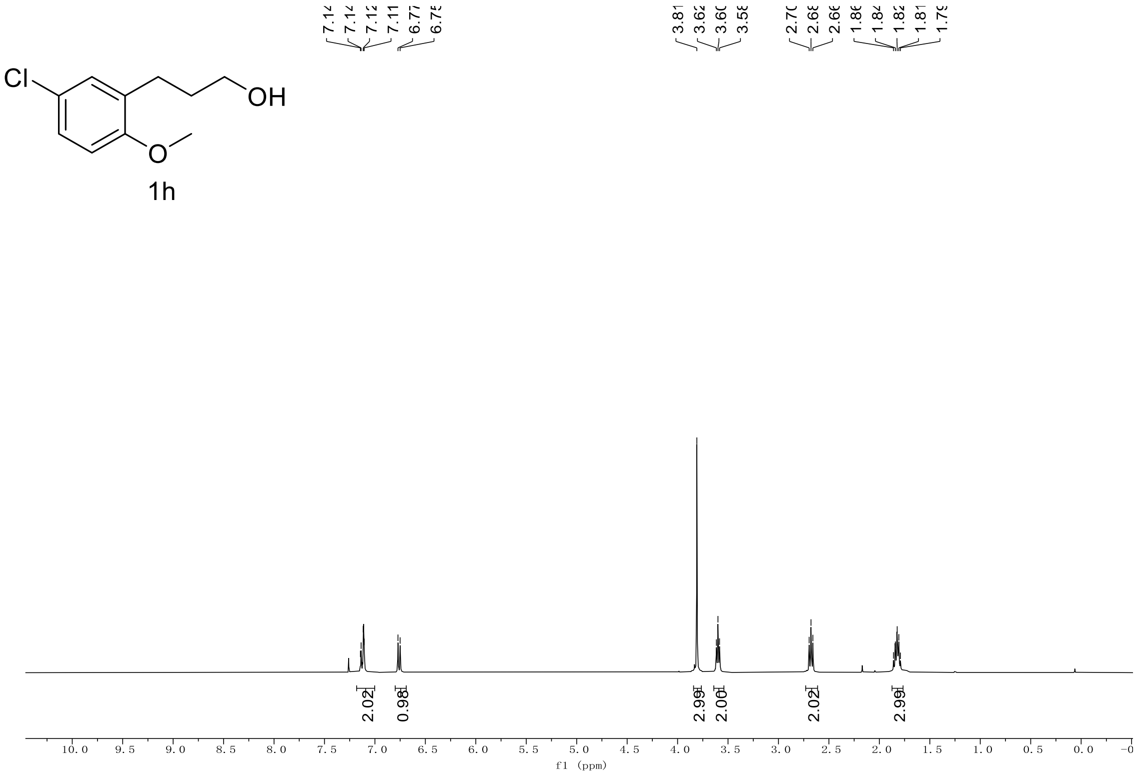

Supplementary Figure 50. ^{1}H NMR of compound **1h** (400 MHz, $CDCl_3$)

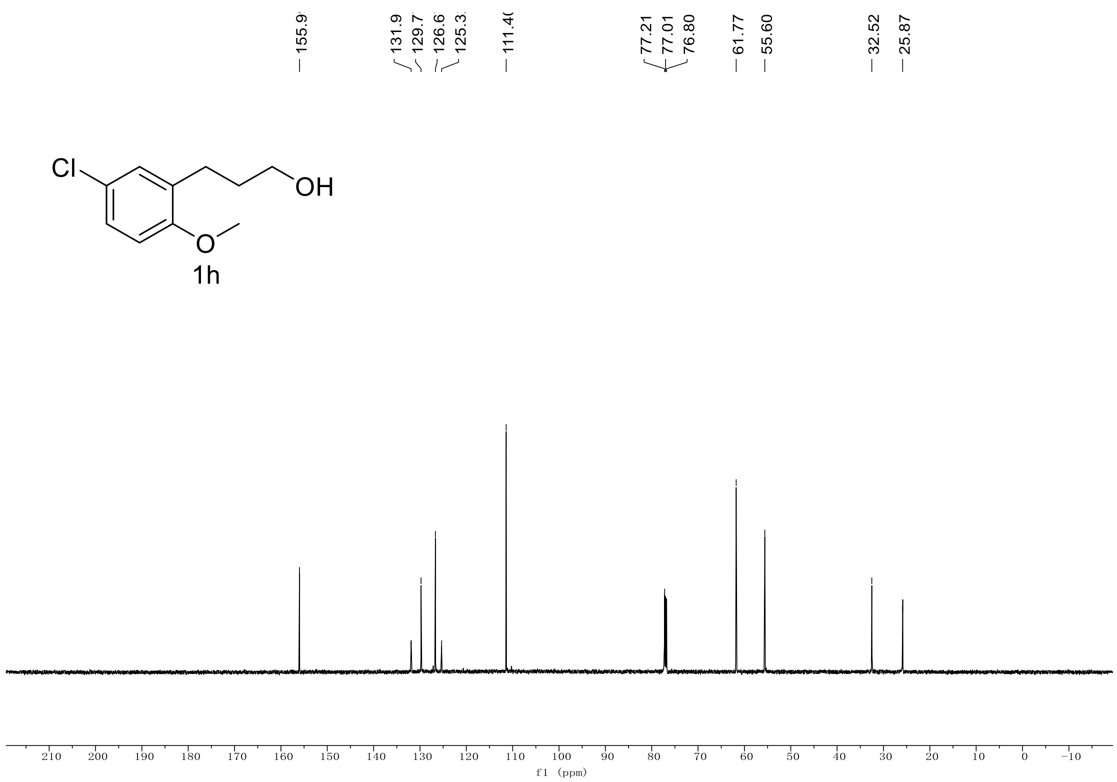

Supplementary Figure 51. ^{13}C NMR of compound **1h** (151 MHz, $CDCl_3$)

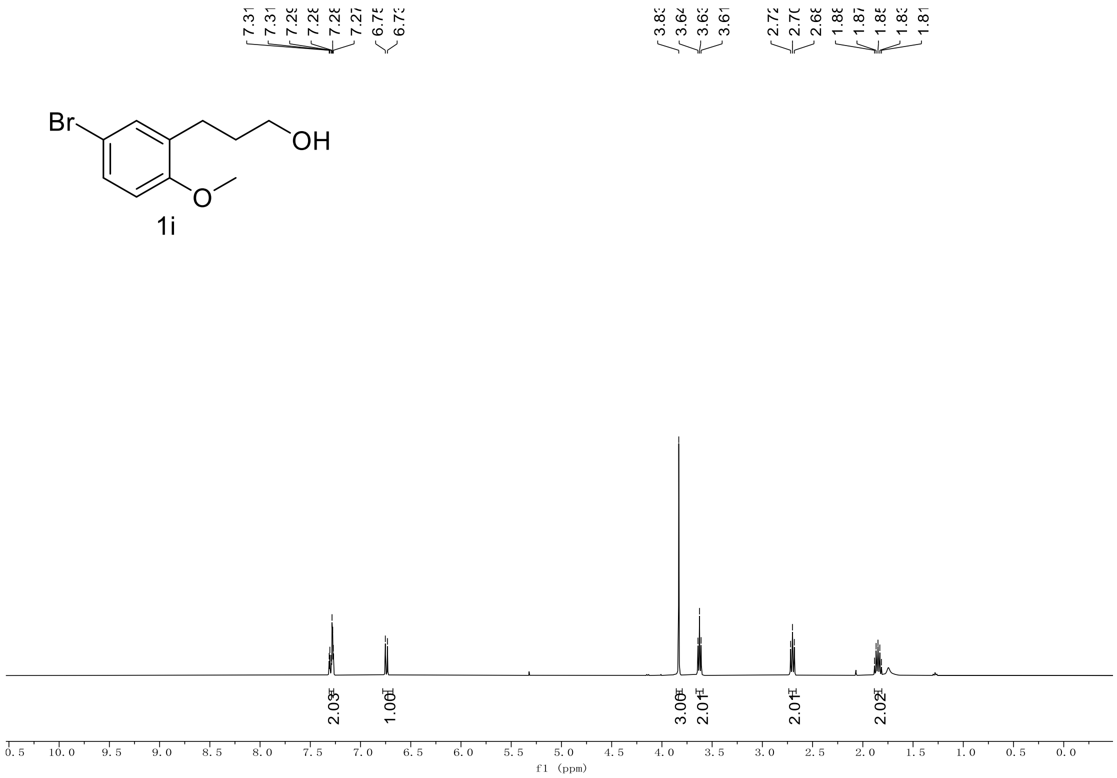

Supplementary Figure 52. ^{1}H NMR of compound **1i** (400 MHz, $CDCl_3$)

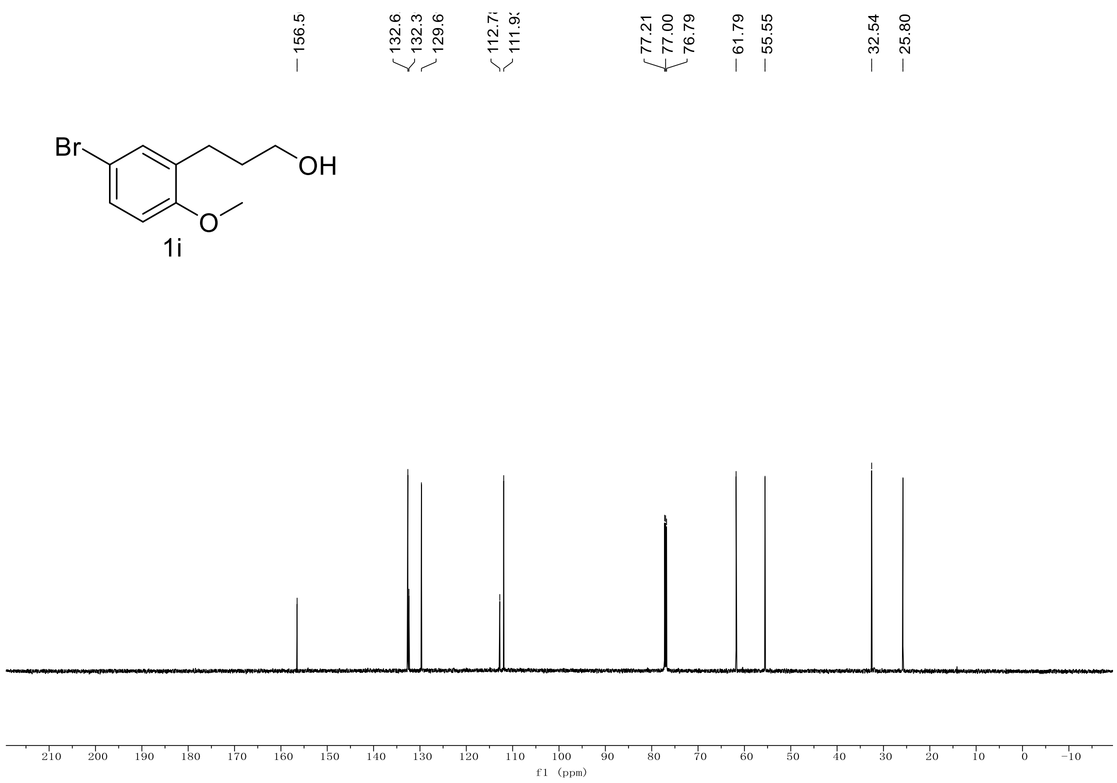

Supplementary Figure 53. ^{13}C NMR of compound **1i** (151 MHz, $CDCl_3$)

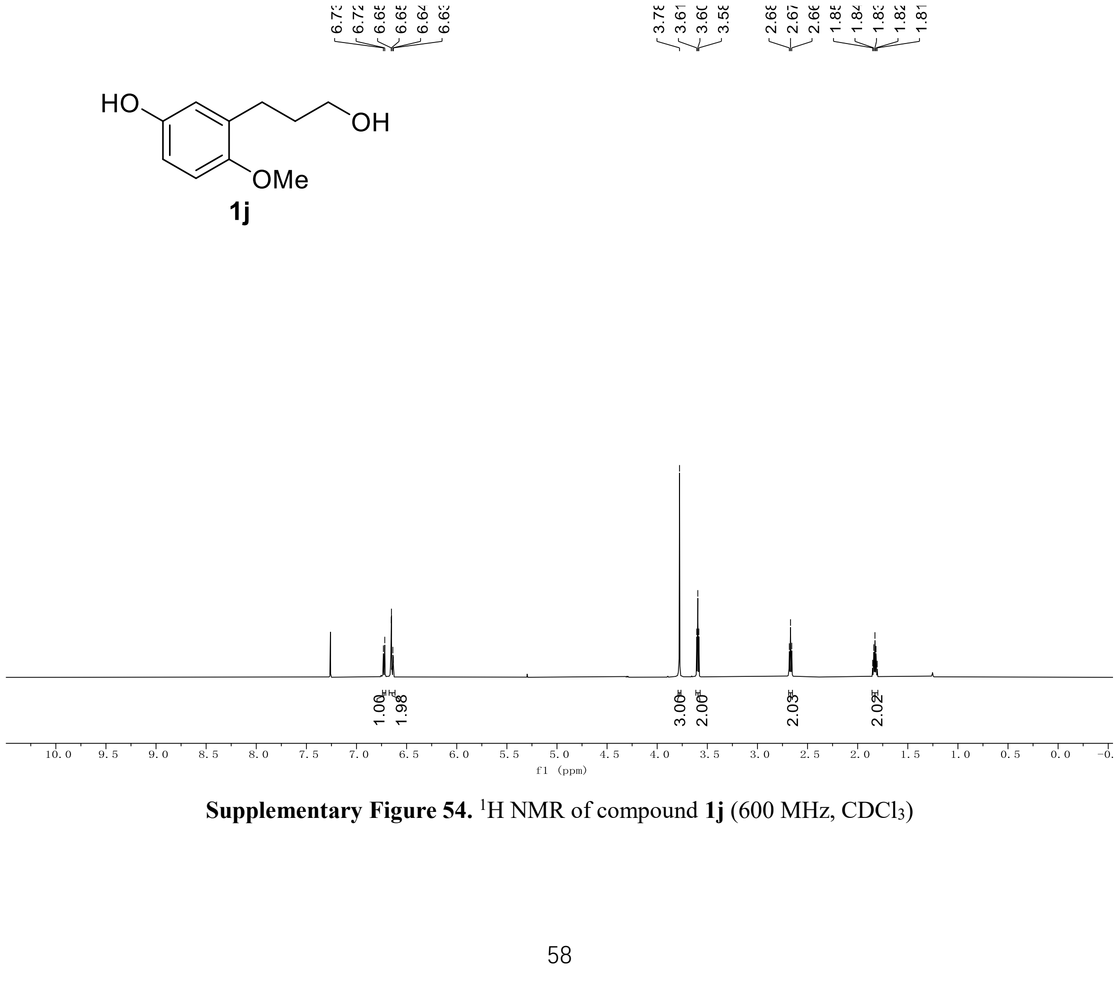

Supplementary Figure 54. ^{1}H NMR of compound **1j** (600 MHz, $CDCl_3$)

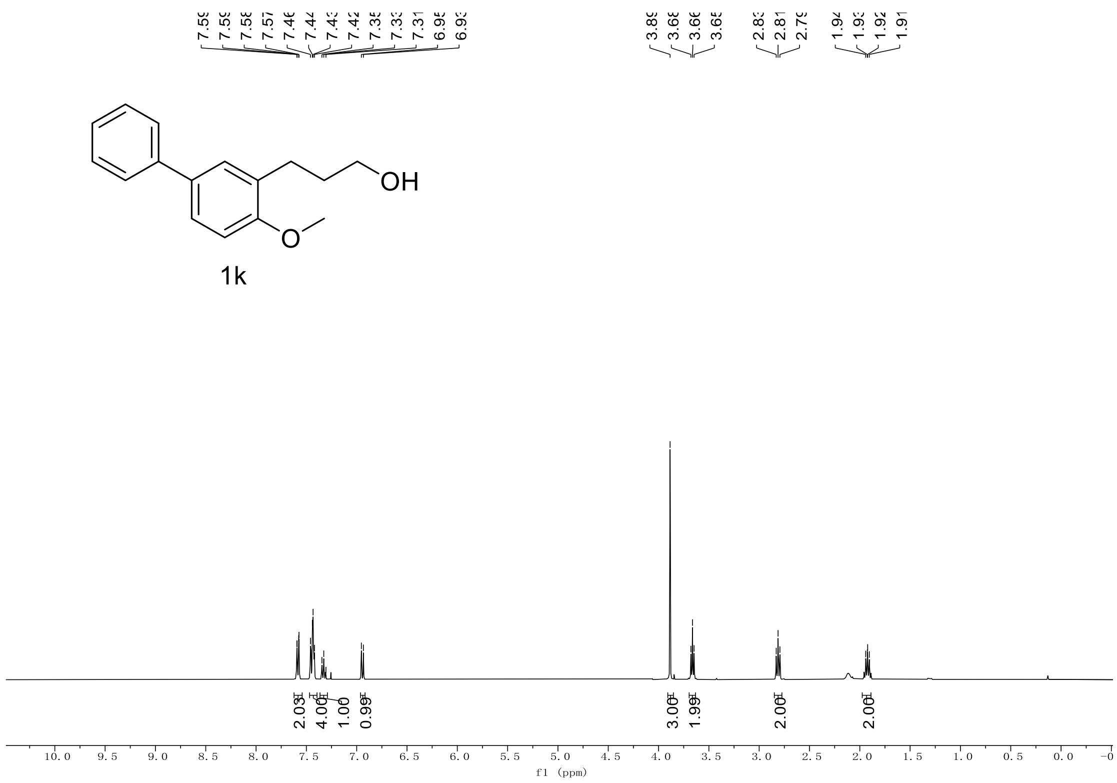

Supplementary Figure 55. ^{1}H NMR of compound **1k** (400 MHz, $CDCl_3$)

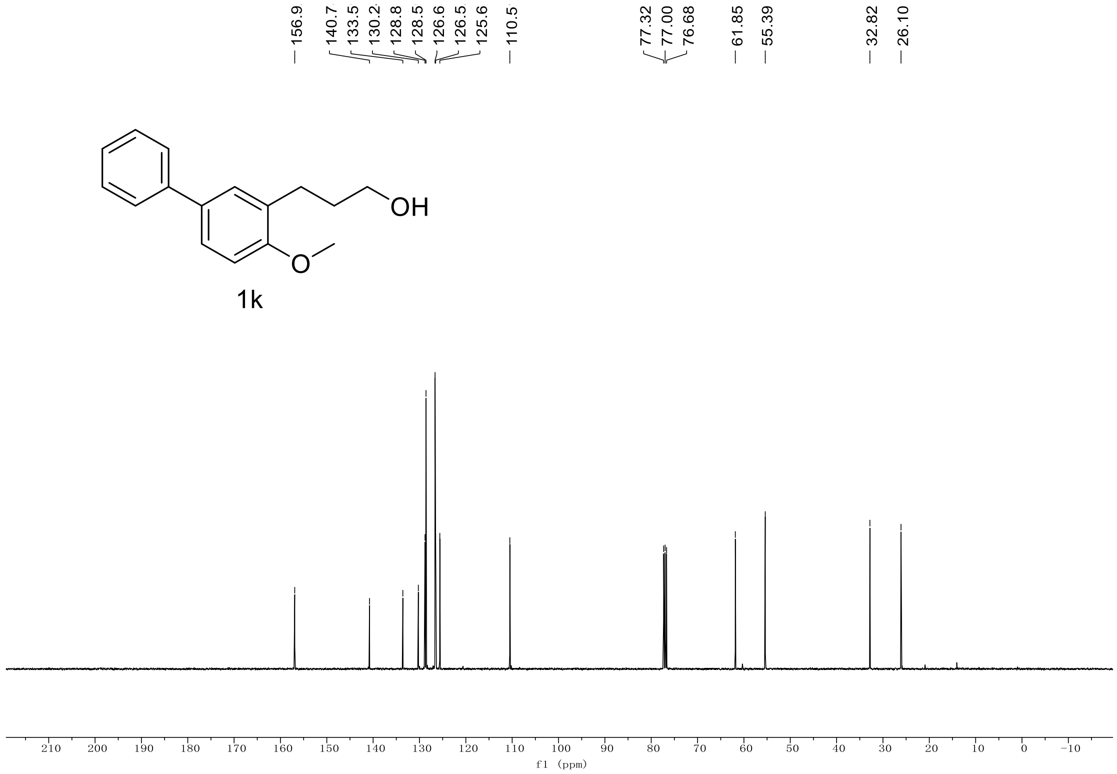

Supplementary Figure 56. ^{13}C NMR of compound **1k** (101 MHz, $CDCl_3$)

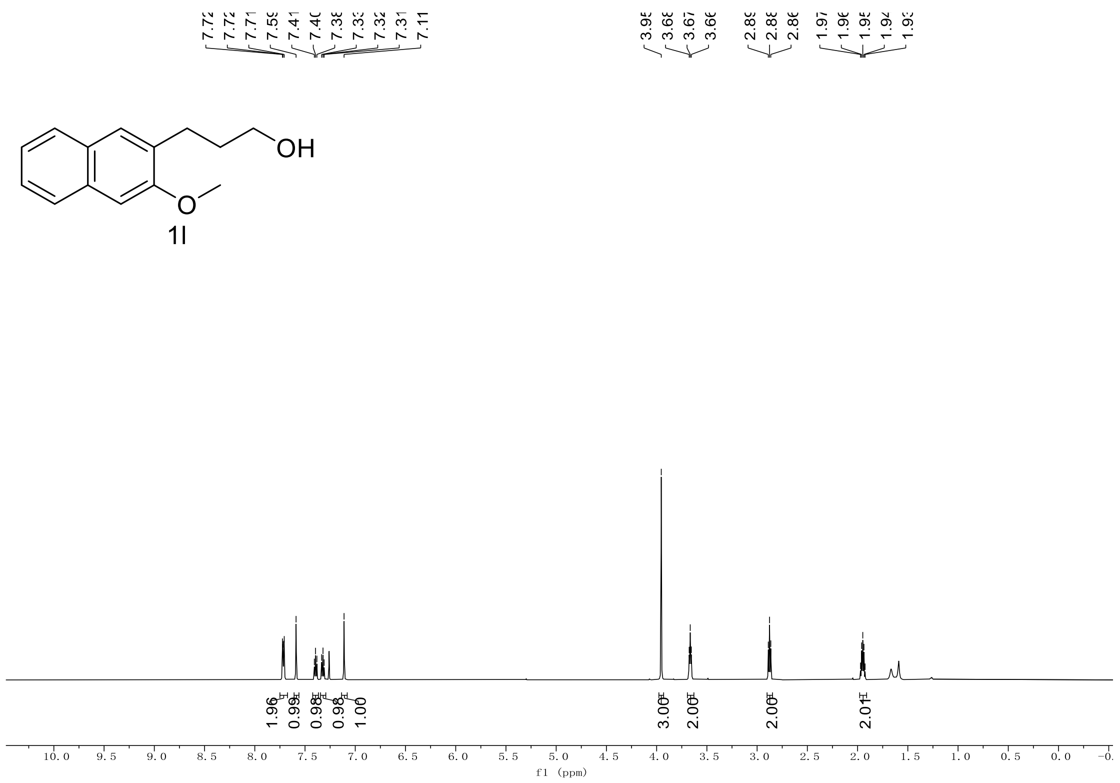

Supplementary Figure 57. ^{1}H NMR of compound **1l** (600 MHz, $CDCl_3$)

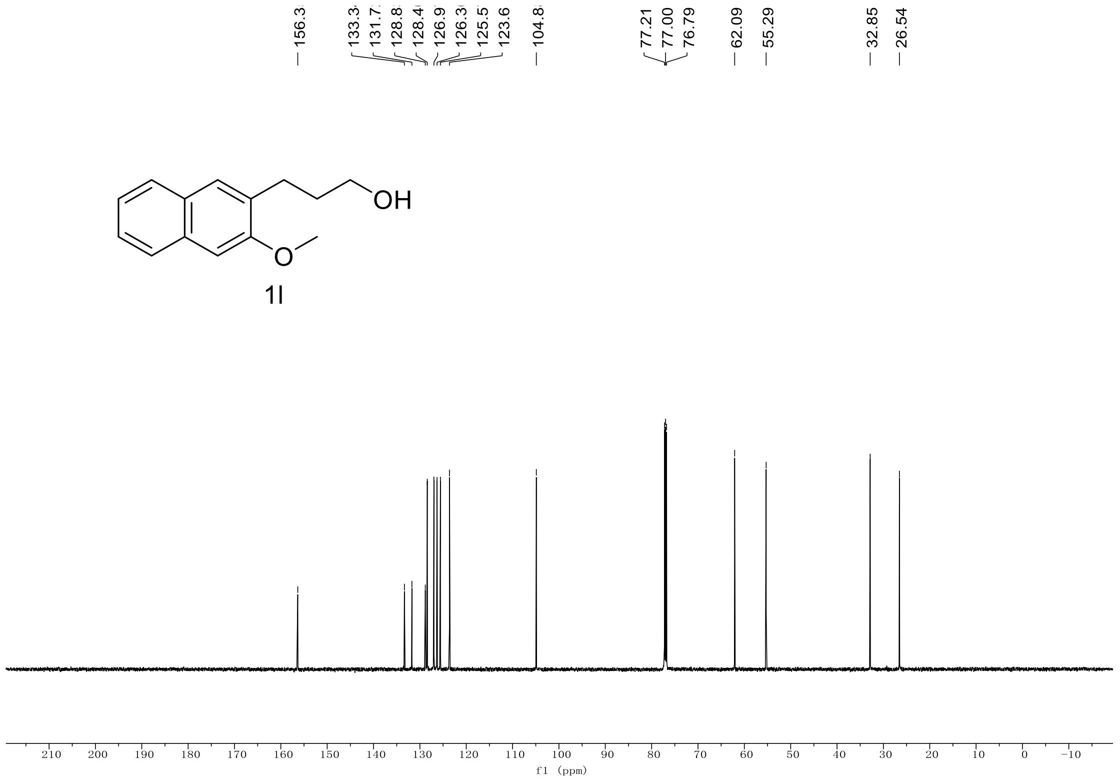

Supplementary Figure 58. ^{13}C NMR of compound **1l** (151 MHz, $CDCl_3$)

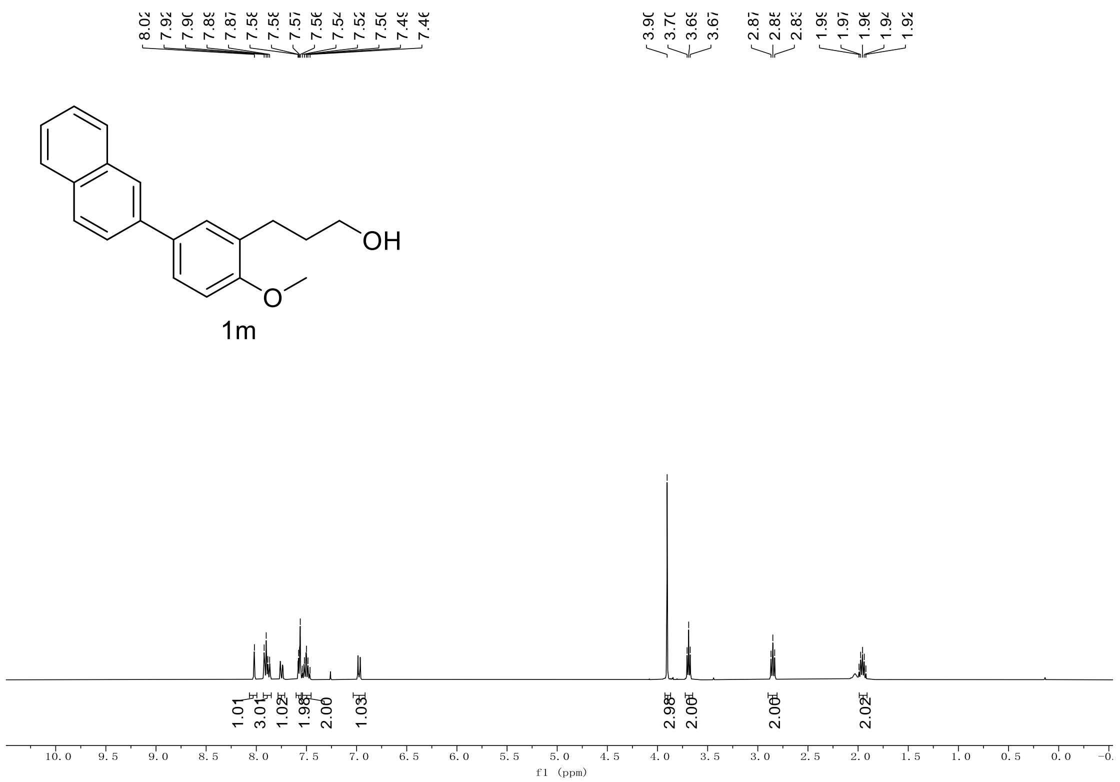

Supplementary Figure 59. ^{1}H NMR of compound **1m** (400 MHz, $CDCl_3$)

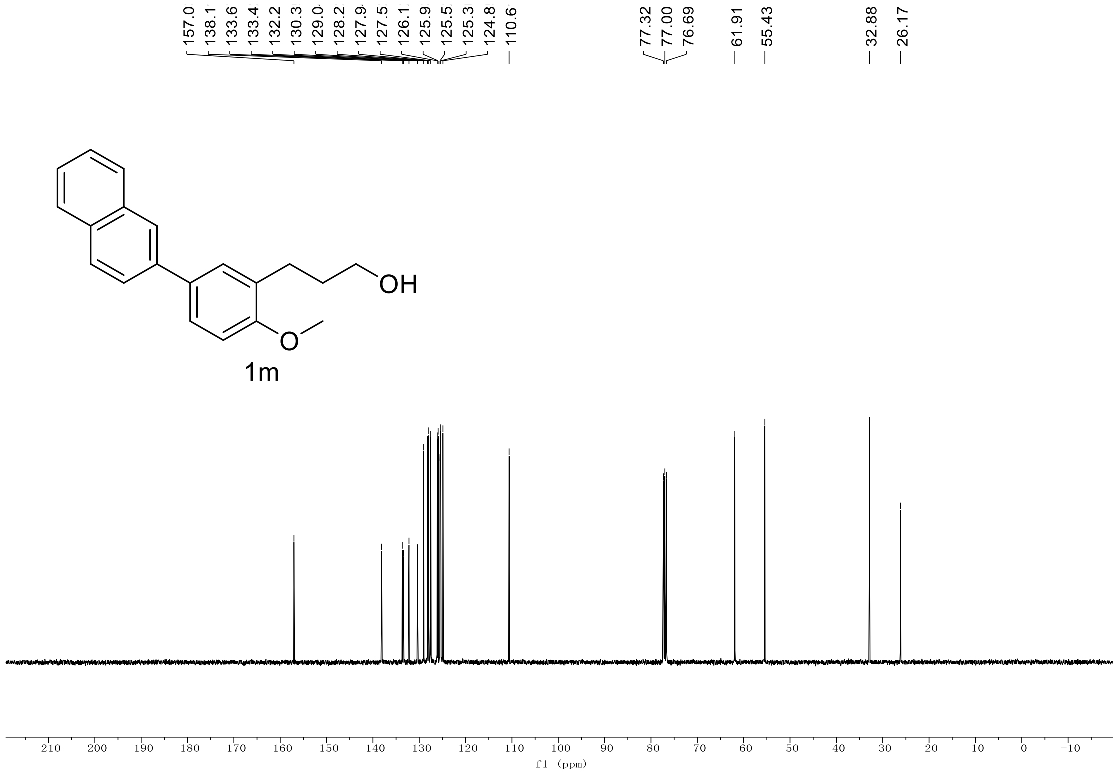

Supplementary Figure 60. ^{13}C NMR of compound **1m** (101 MHz, $CDCl_3$)

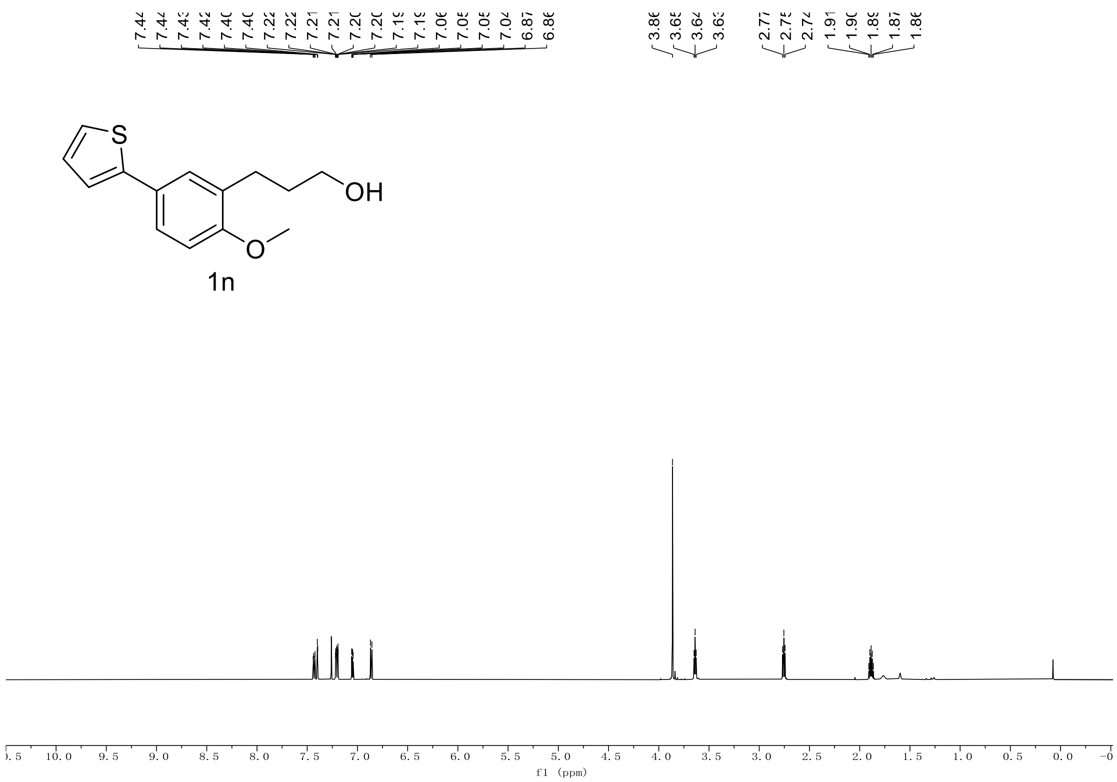

Supplementary Figure 61. ^{1}H NMR of compound **1n** (600 MHz, $CDCl_3$)

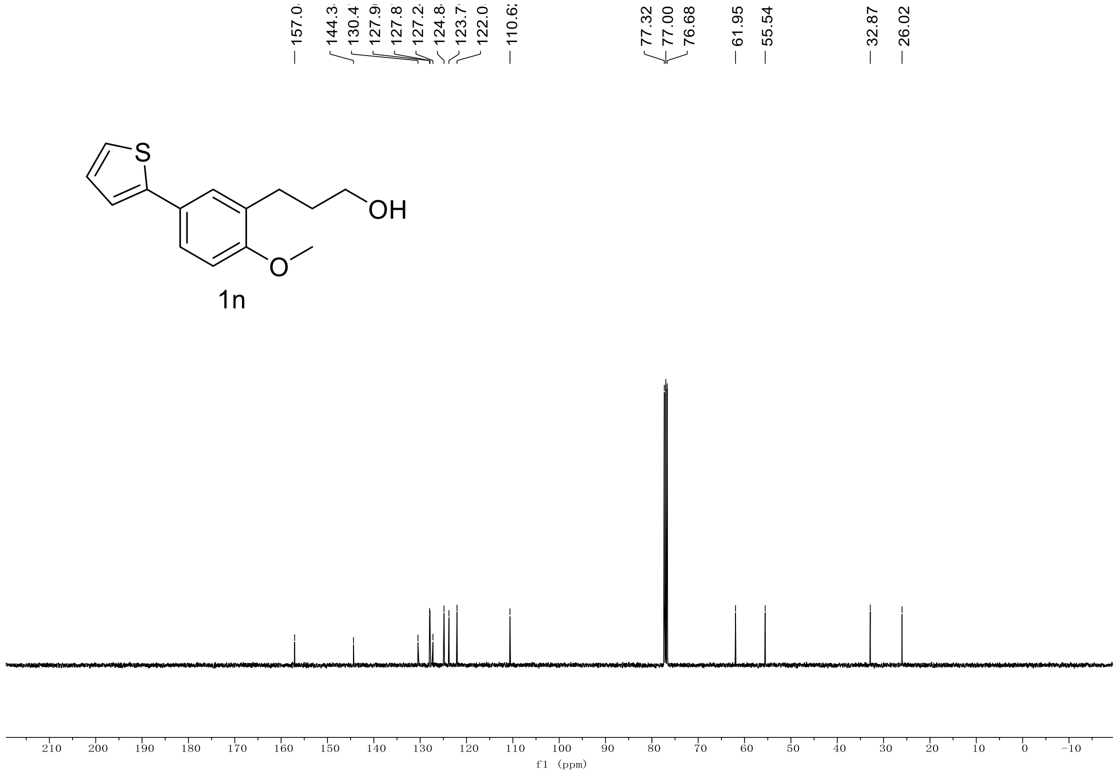

Supplementary Figure 62. ^{13}C NMR of compound **1n** (101 MHz, $CDCl_3$)

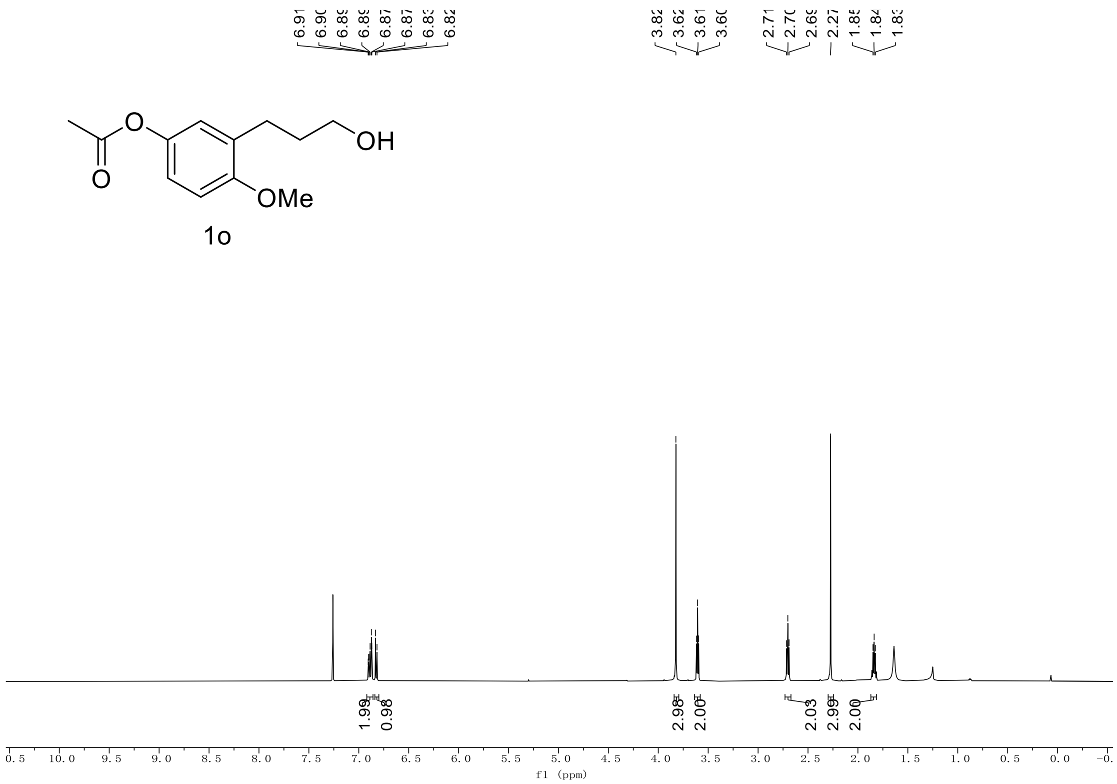

Supplementary Figure 63. ^{1}H NMR of compound **1o** (600 MHz, $CDCl_3$)

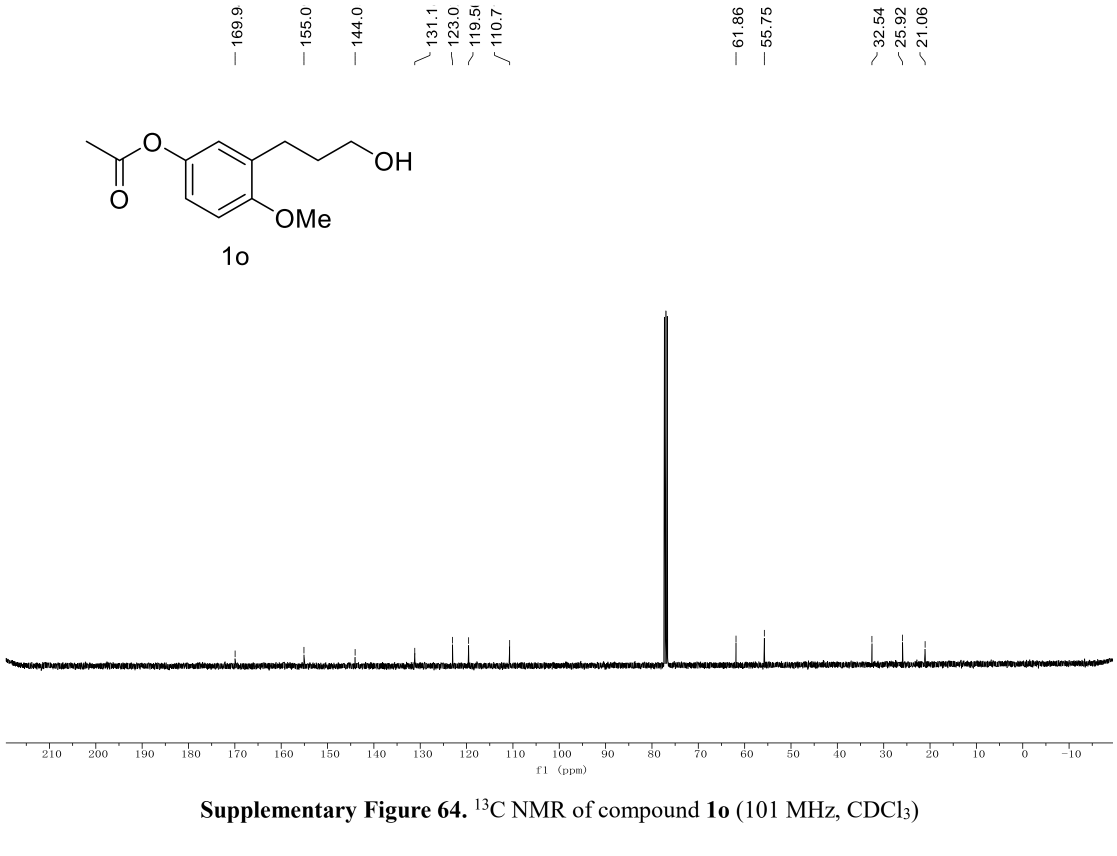

Supplementary Figure 64. ^{13}C NMR of compound **1o** (101 MHz, $CDCl_3$)

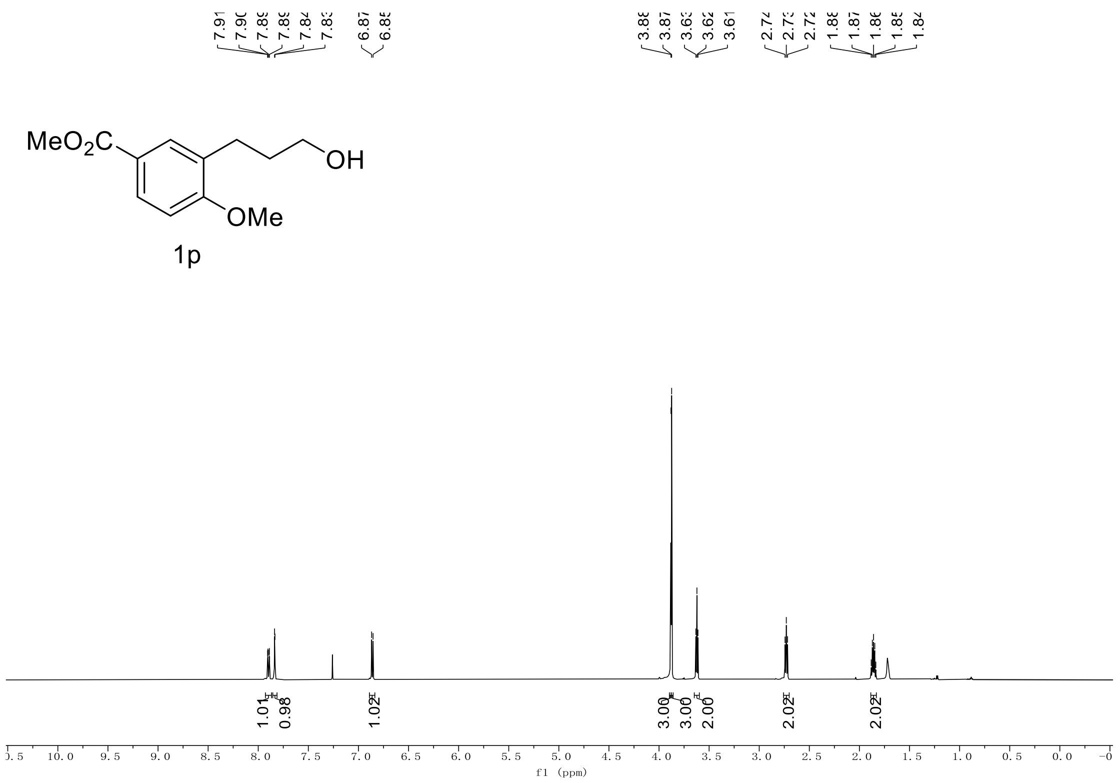

Supplementary Figure 65. ^{1}H NMR of compound **1p** (600 MHz, $CDCl_3$)

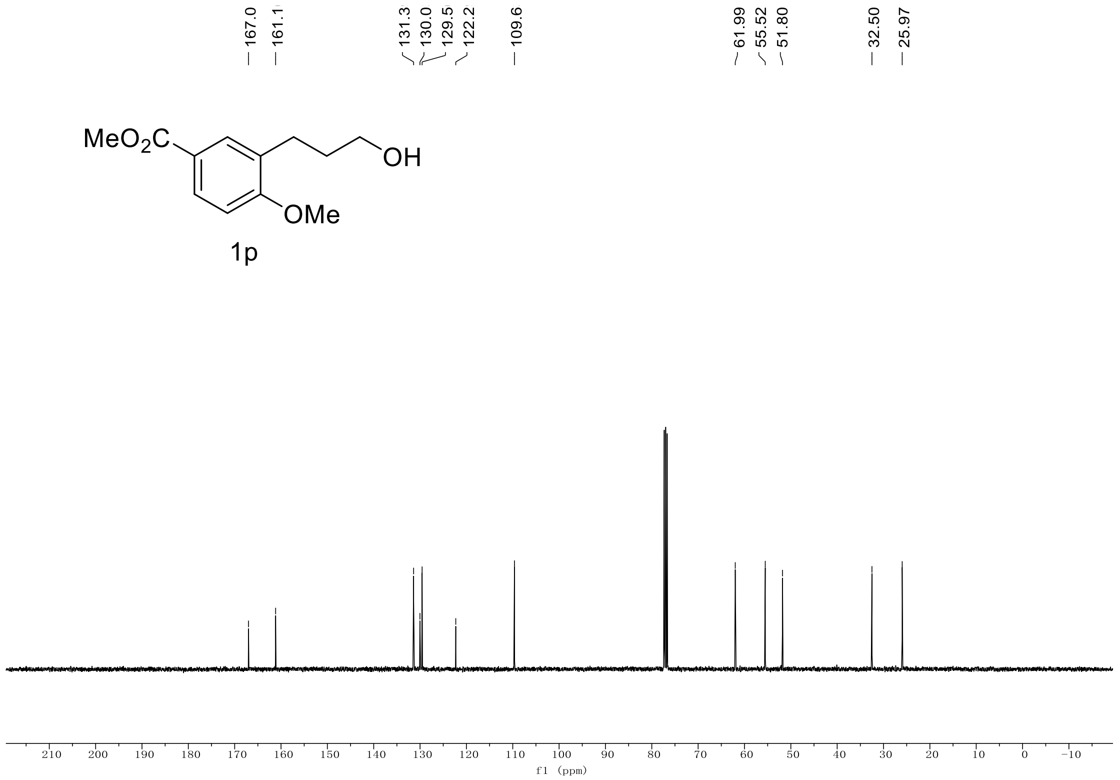

Supplementary Figure 66. ^{13}C NMR of compound **1p** (101 MHz, $CDCl_3$)

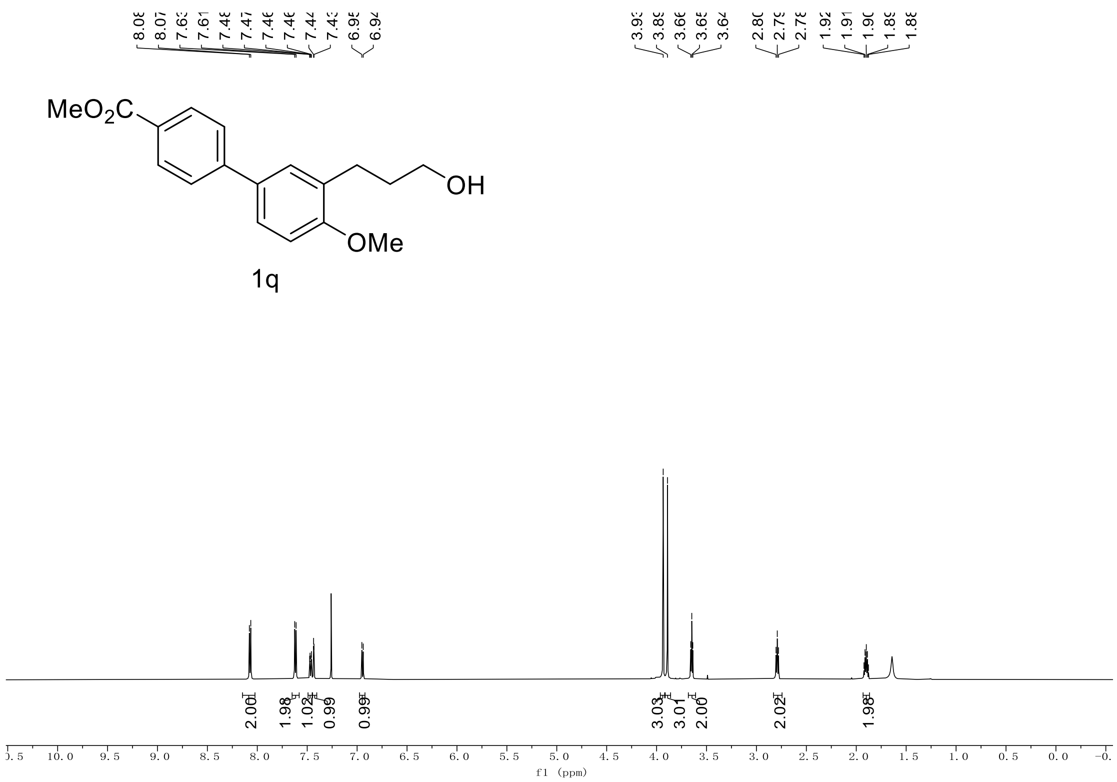

Supplementary Figure 67. ^{1}H NMR of compound **1q** (600 MHz, $CDCl_3$)

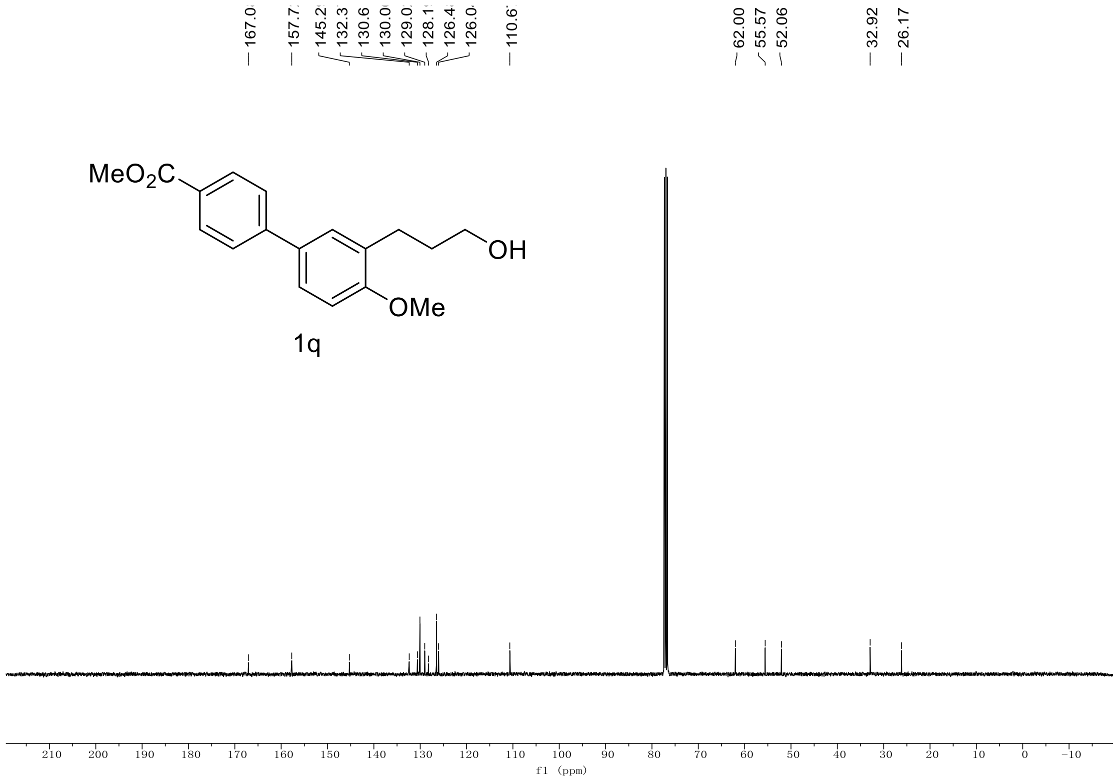

Supplementary Figure 68. ^{13}C NMR of compound **1q** (101 MHz, $CDCl_3$)

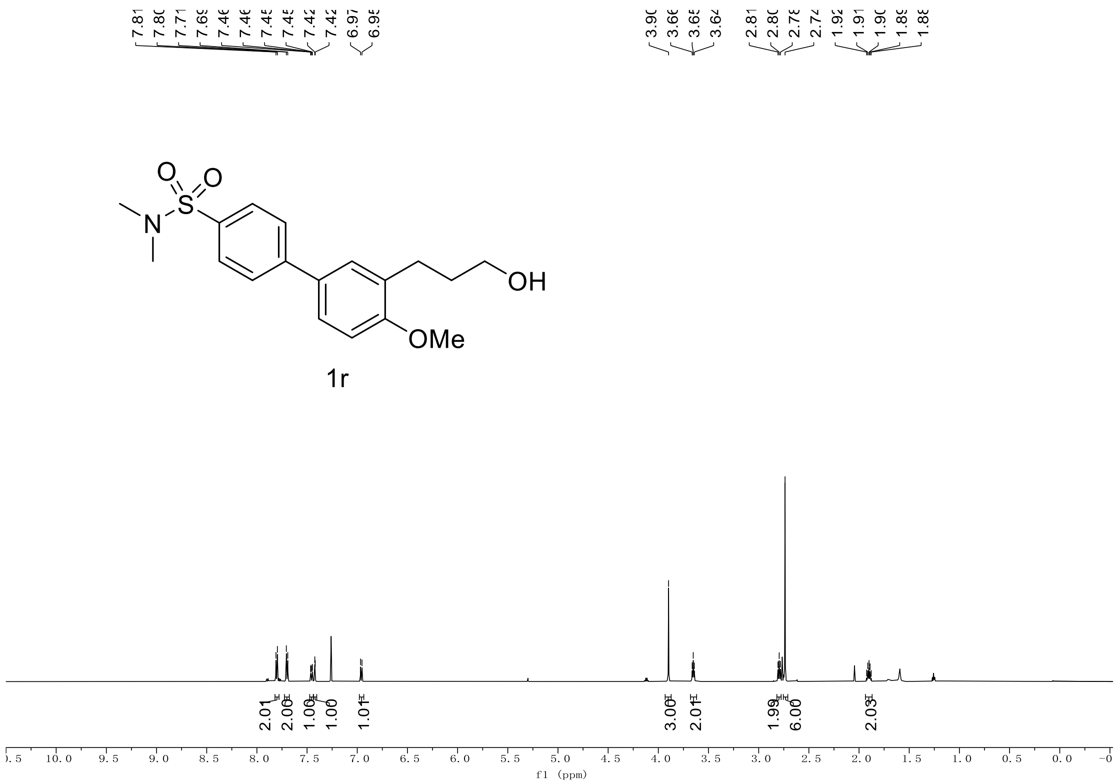

Supplementary Figure 69. ^{1}H NMR of compound **1r** (600 MHz, $CDCl_3$)

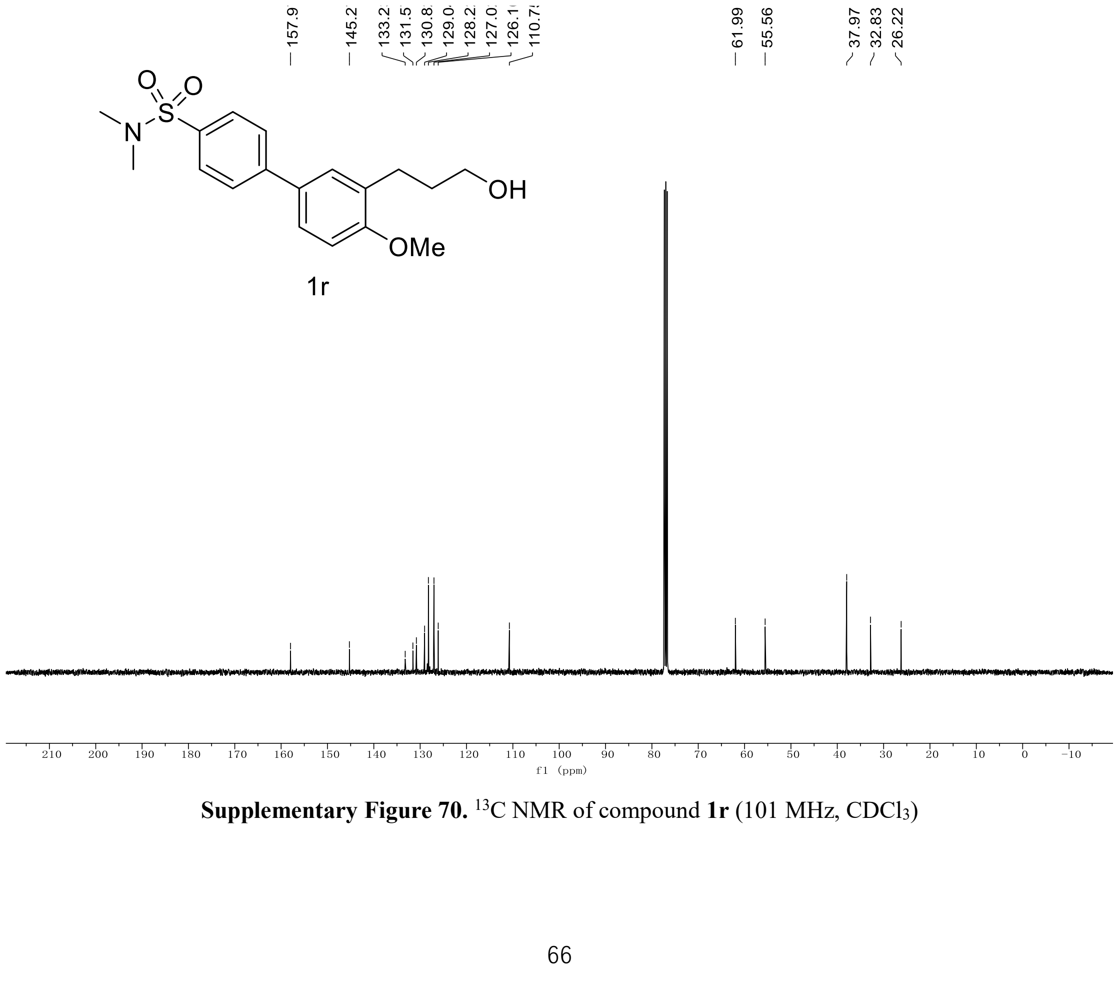

Supplementary Figure 70. ^{13}C NMR of compound **1r** (101 MHz, $CDCl_3$)

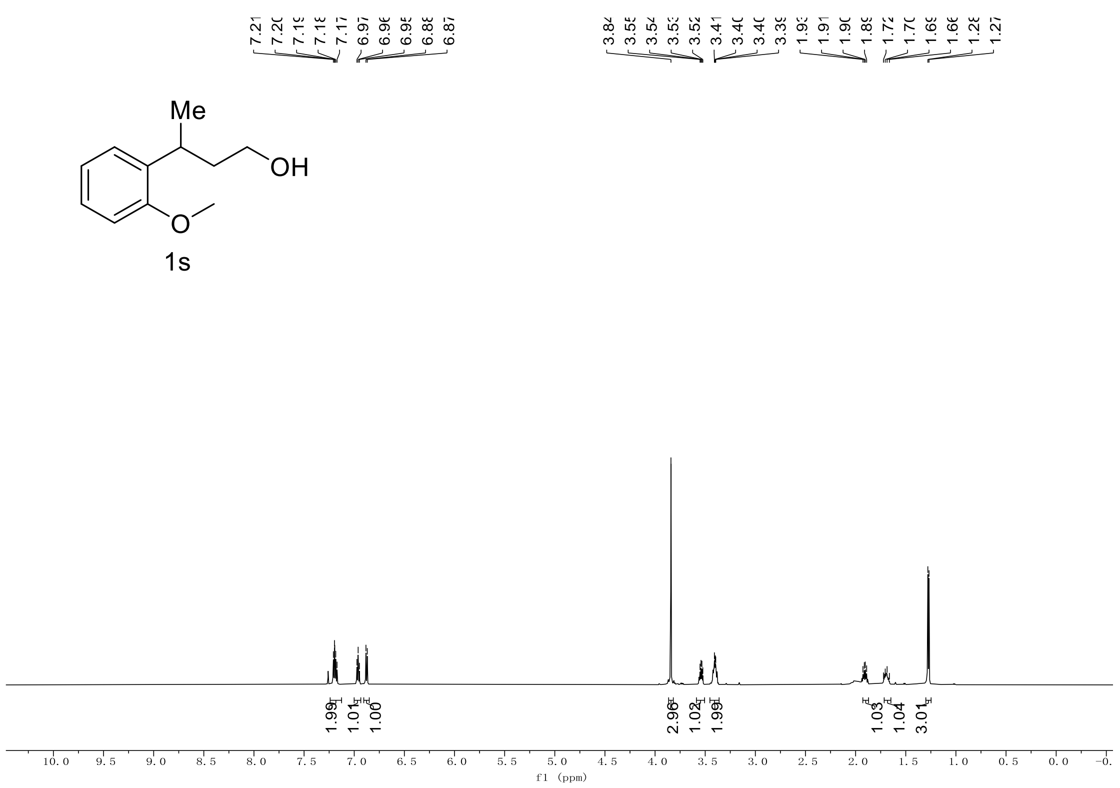

Supplementary Figure 71. ^{1}H NMR of compound **1ss** (600 MHz, $CDCl_3$)

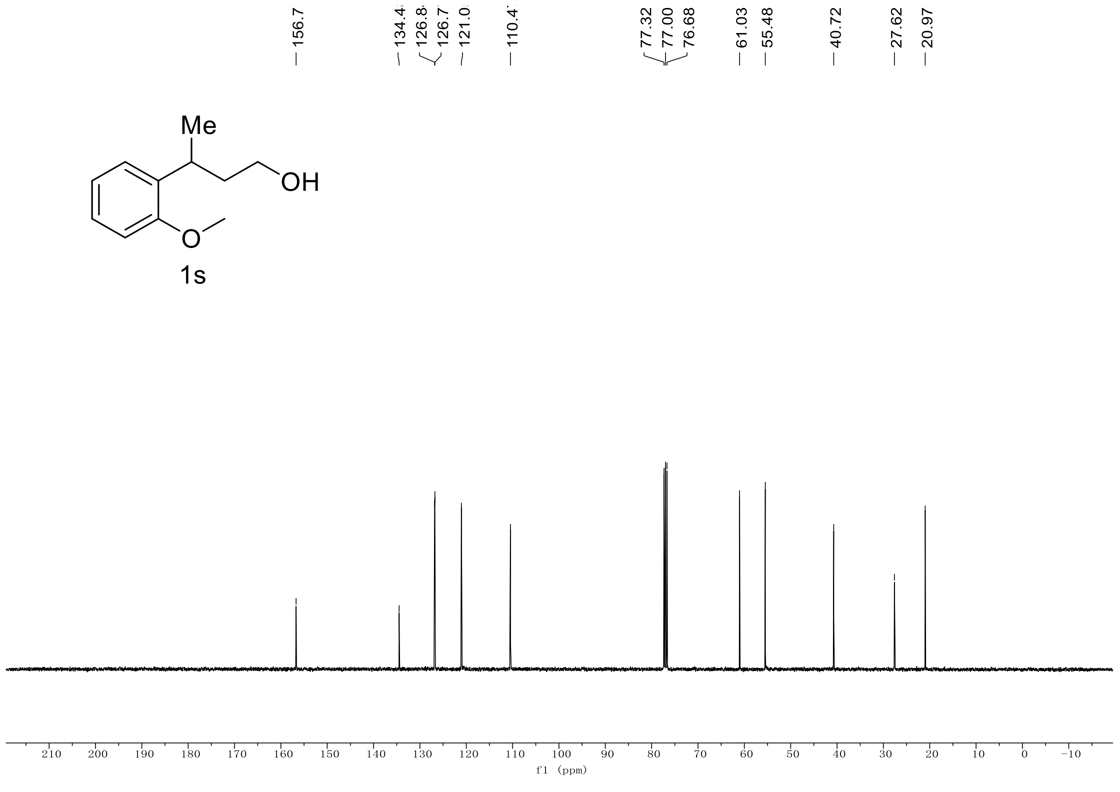

Supplementary Figure 72. ^{13}C NMR of compound **1s** (101 MHz, $CDCl_3$)

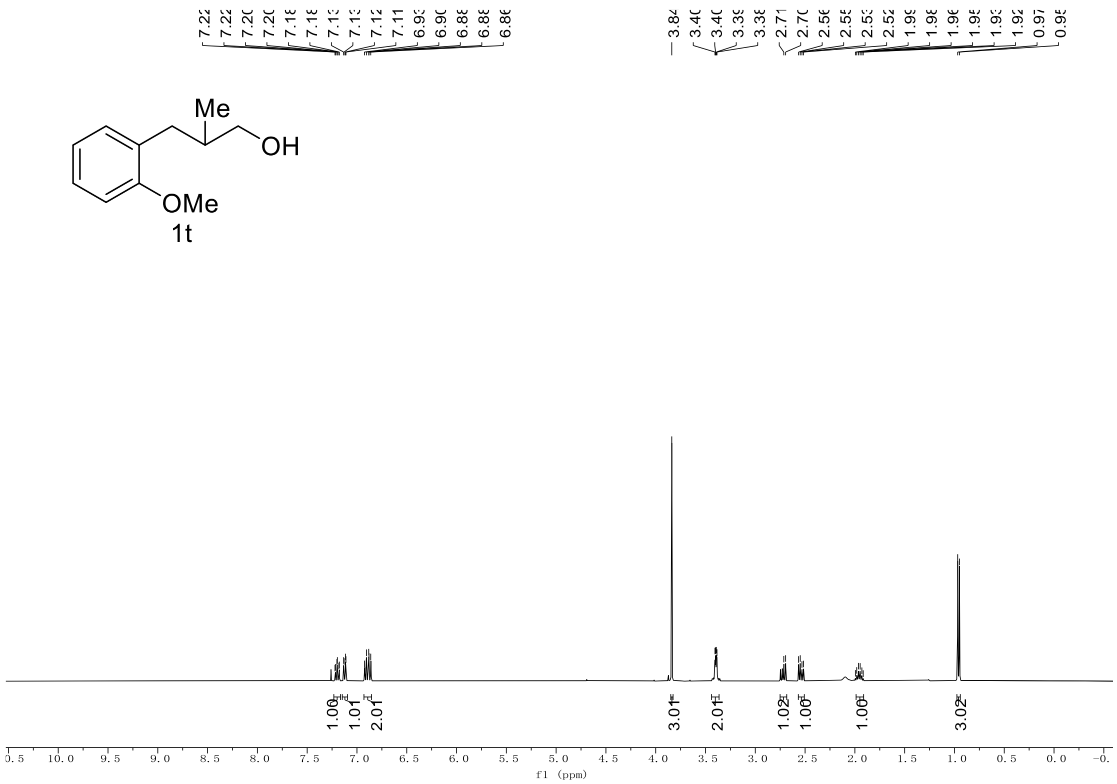

Supplementary Figure 73. ^{1}H NMR of compound **1t** (400 MHz, $CDCl_3$)

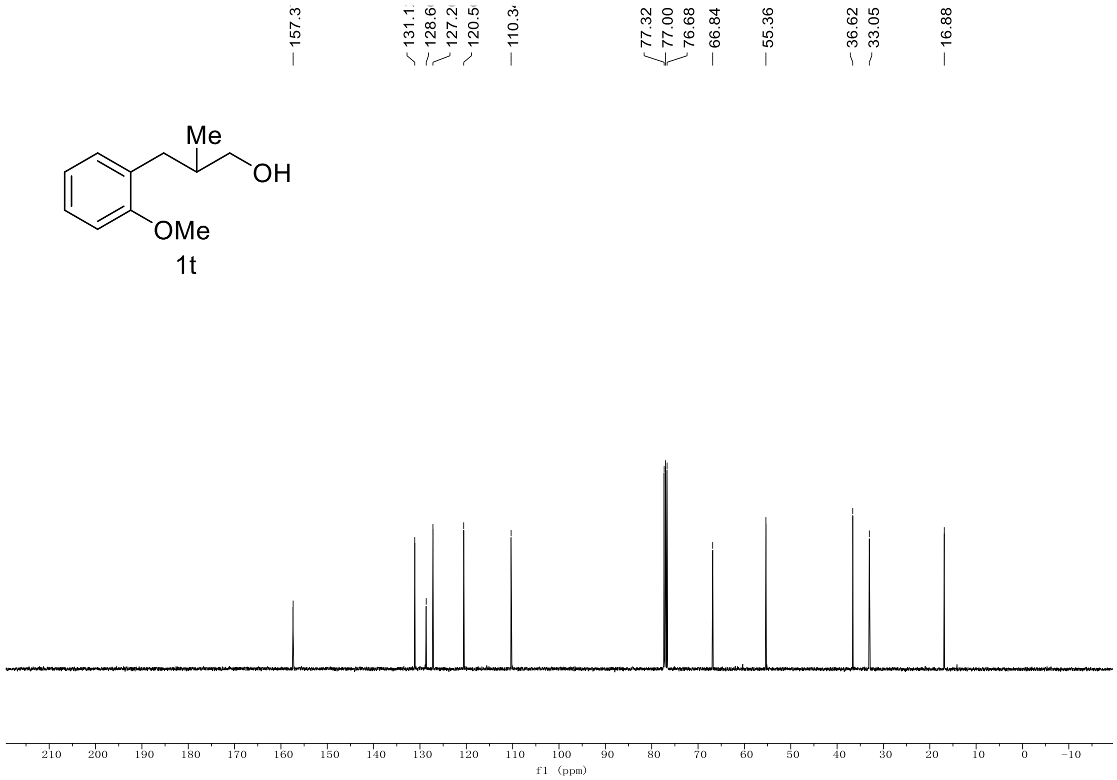

Supplementary Figure 74. ^{13}C NMR of compound **1t** (101 MHz, $CDCl_3$)

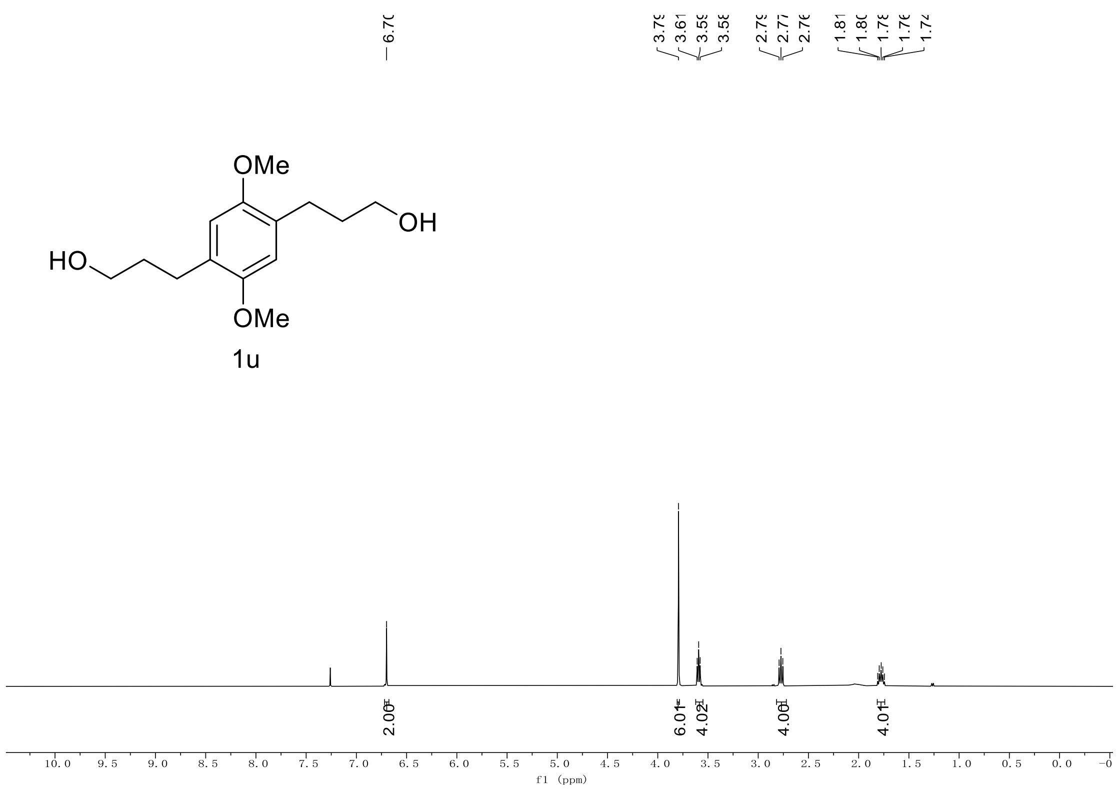

Supplementary Figure 75. ^{1}H NMR of compound **1u** (400 MHz, $CDCl_3$)

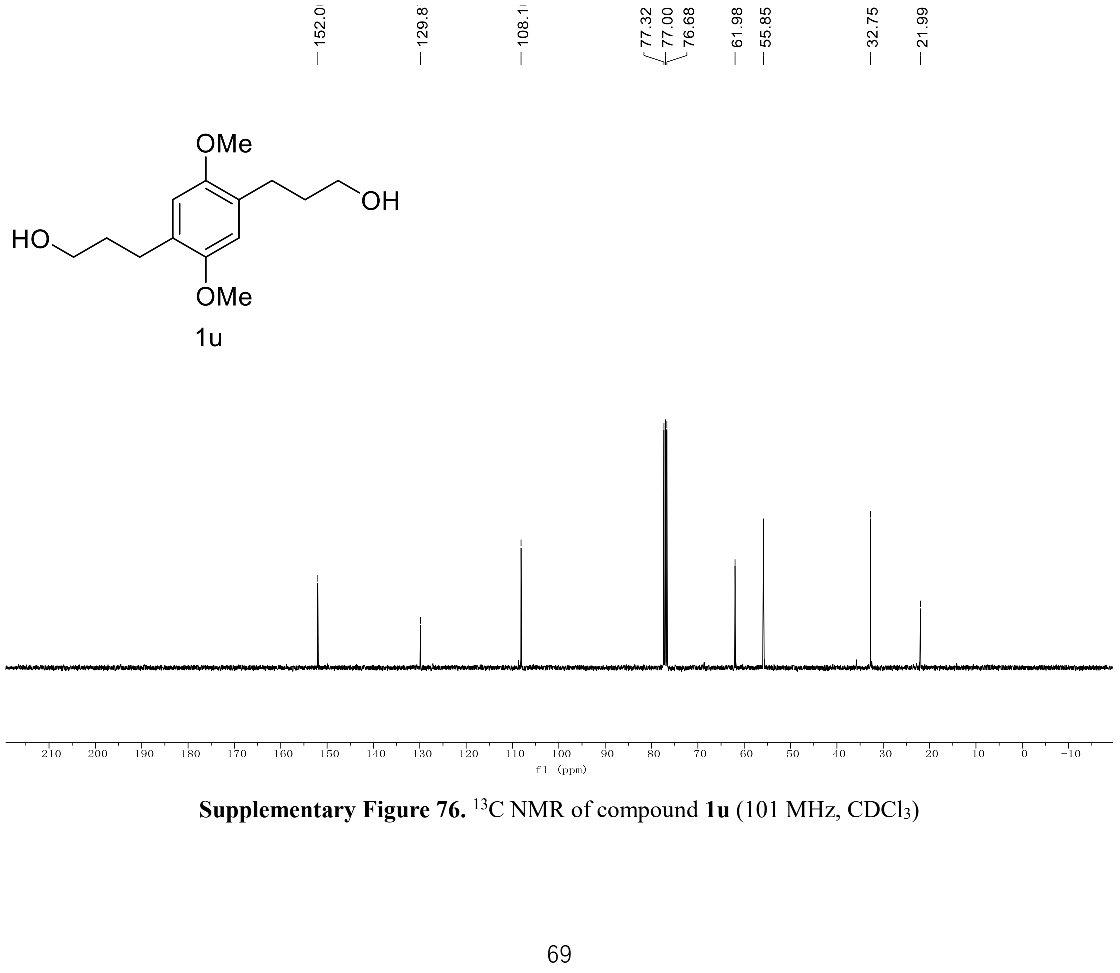

Supplementary Figure 76. ^{13}C NMR of compound **1u** (101 MHz, $CDCl_3$)

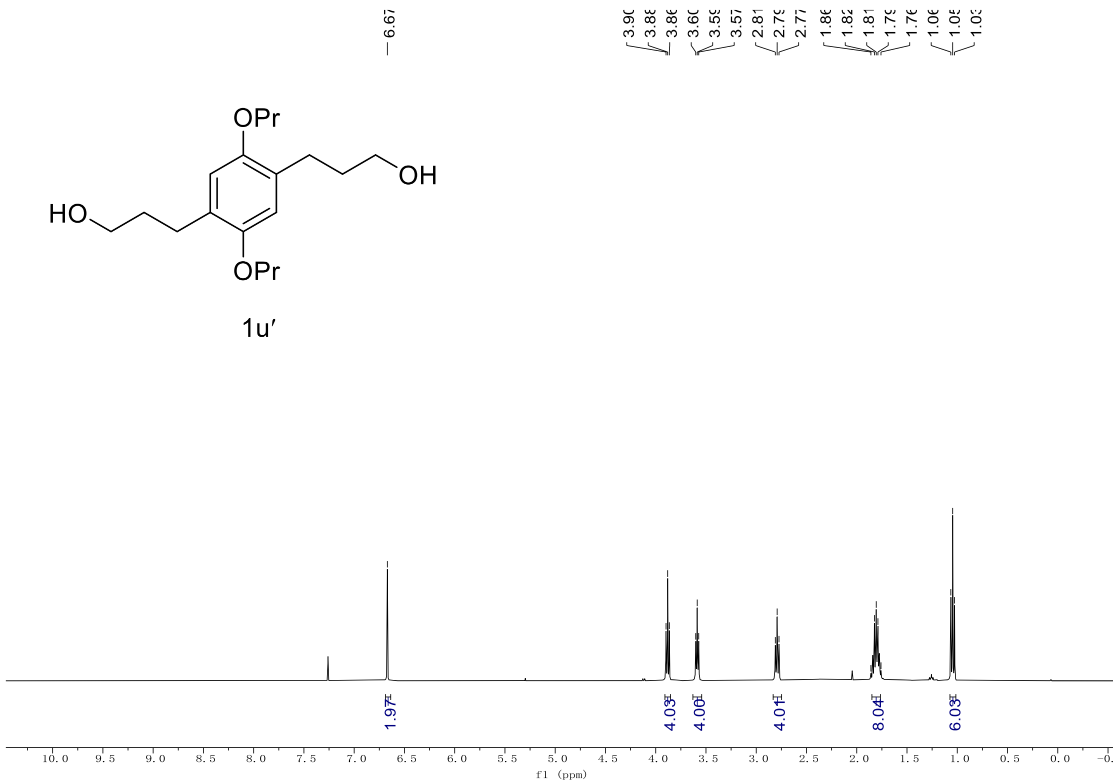

Supplementary Figure 77. ^{1}H NMR of compound **1u'** (400 MHz, $CDCl_3$)

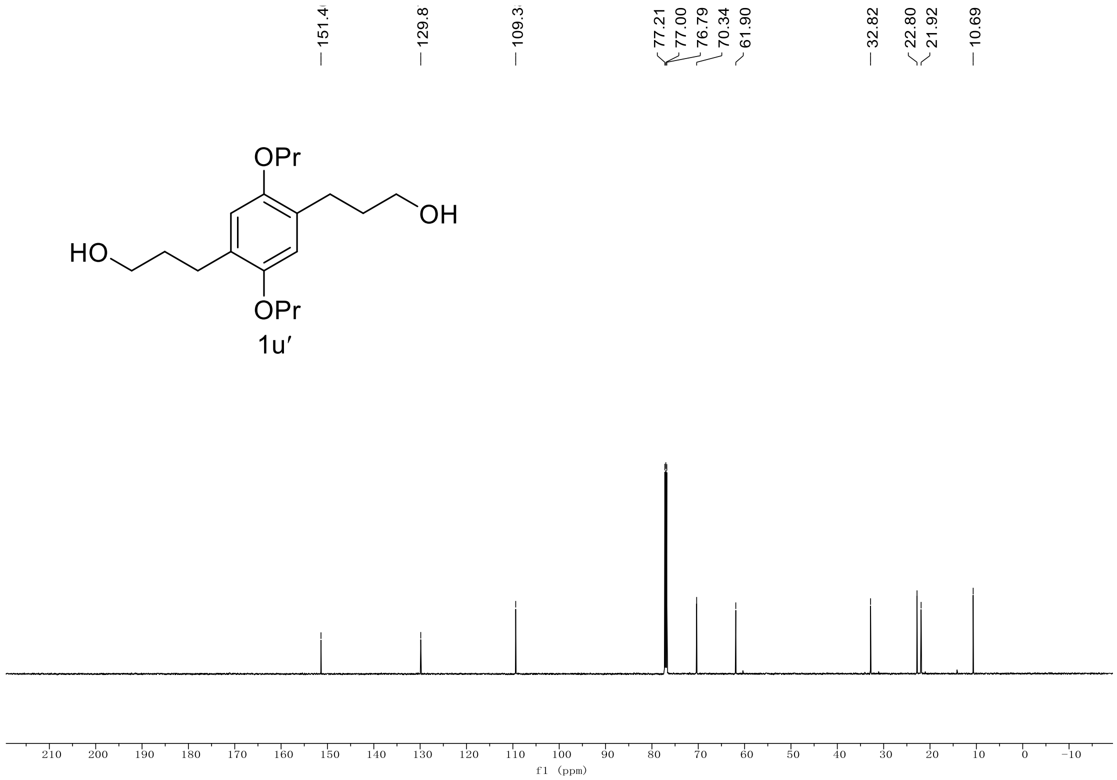

Supplementary Figure 78. ^{13}C NMR of compound **1 u'** (151 MHz, $CDCl_3$)

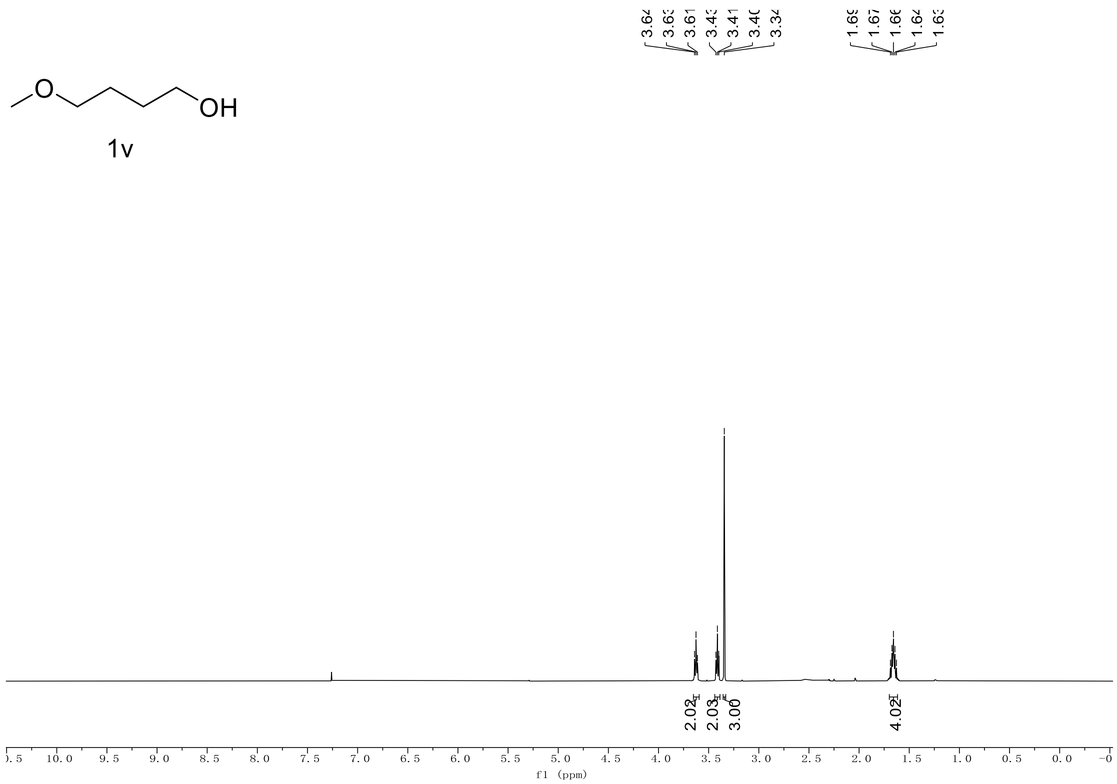

Supplementary Figure 79. ^{1}H NMR of compound **1v** (400 MHz, $CDCl_3$)

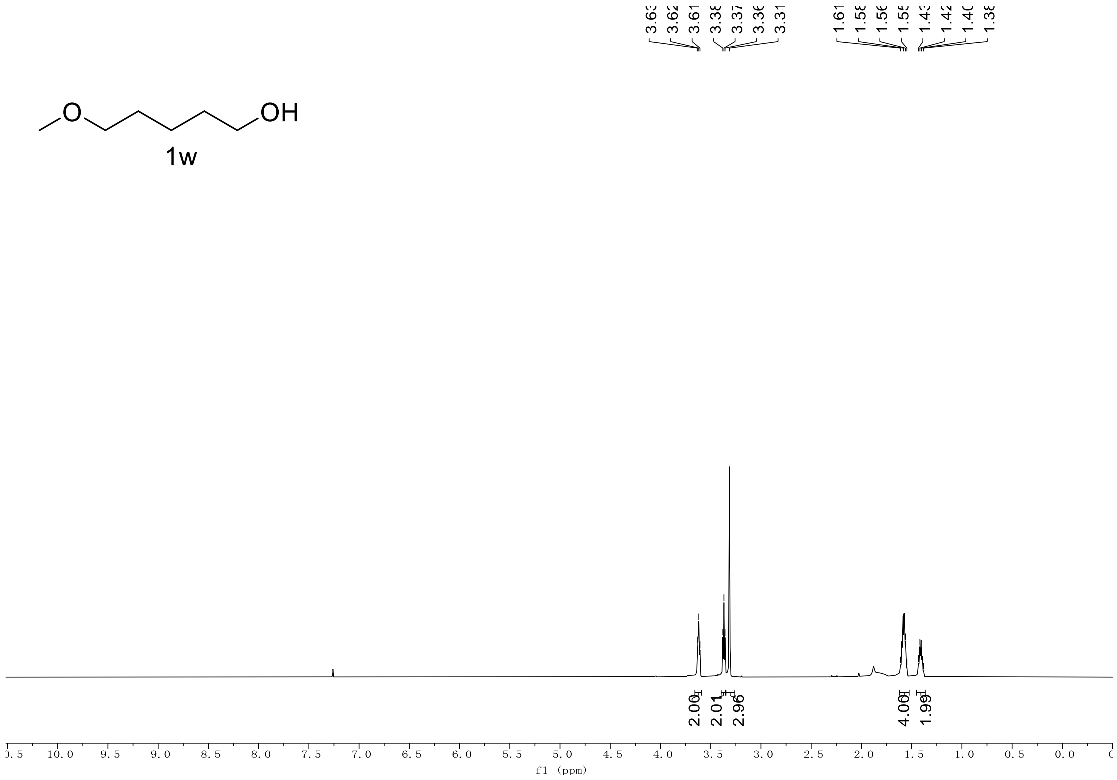

Supplementary Figure 80. ^{1}H NMR of compound **1w** (400 MHz, $CDCl_3$)

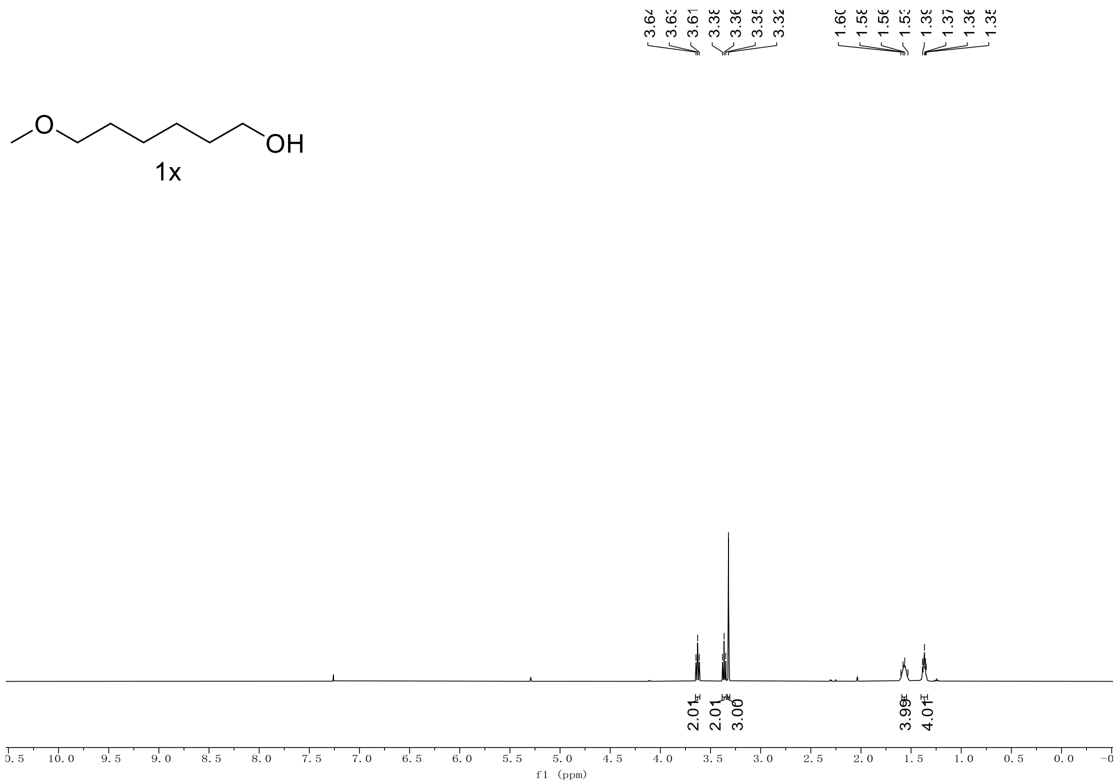

Supplementary Figure 81. ^{1}H NMR of compound **1x** (400 MHz, $CDCl_3$)

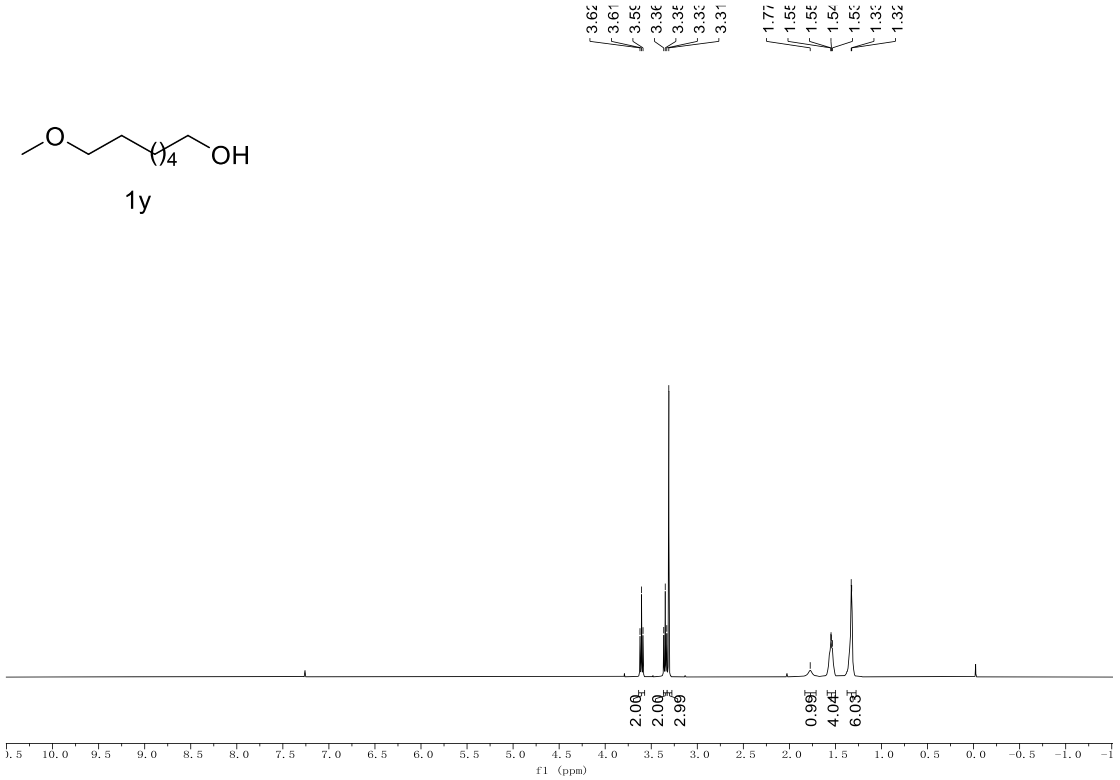

Supplementary Figure 82. ^{1}H NMR of compound **1y** (400 MHz, $CDCl_3$)

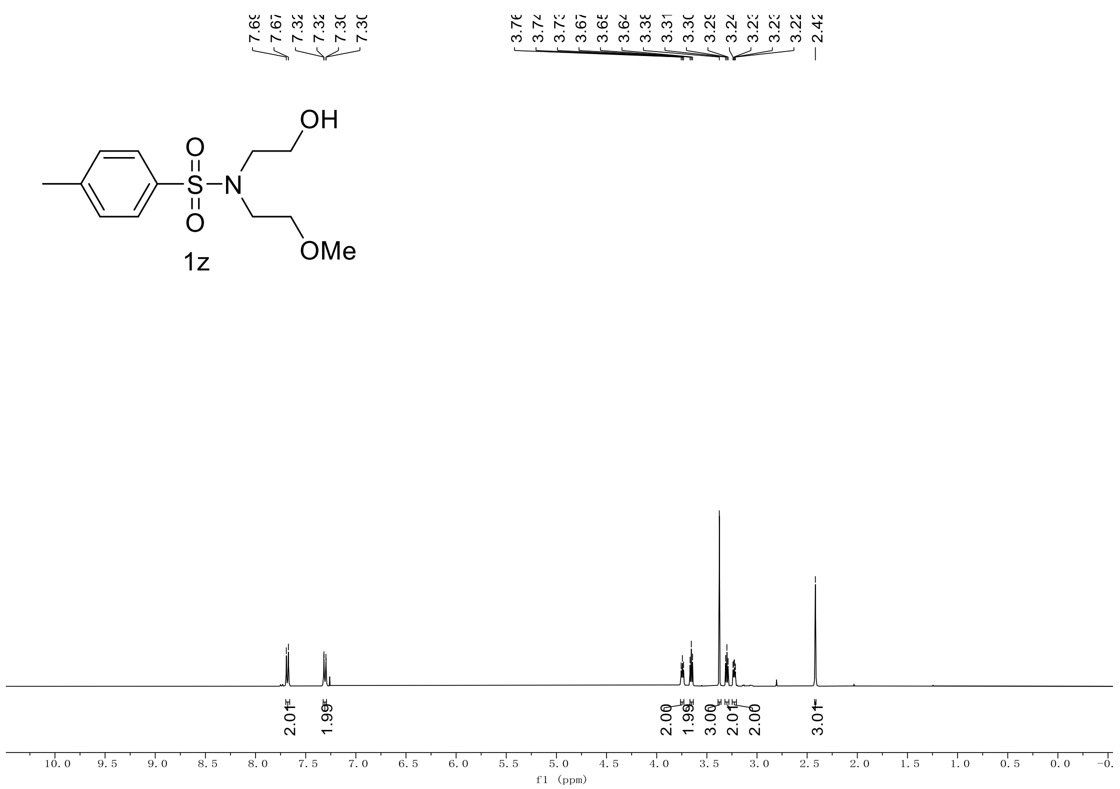

Supplementary Figure 83. ^{1}H NMR of compound **1z** (400 MHz, $CDCl_3$)

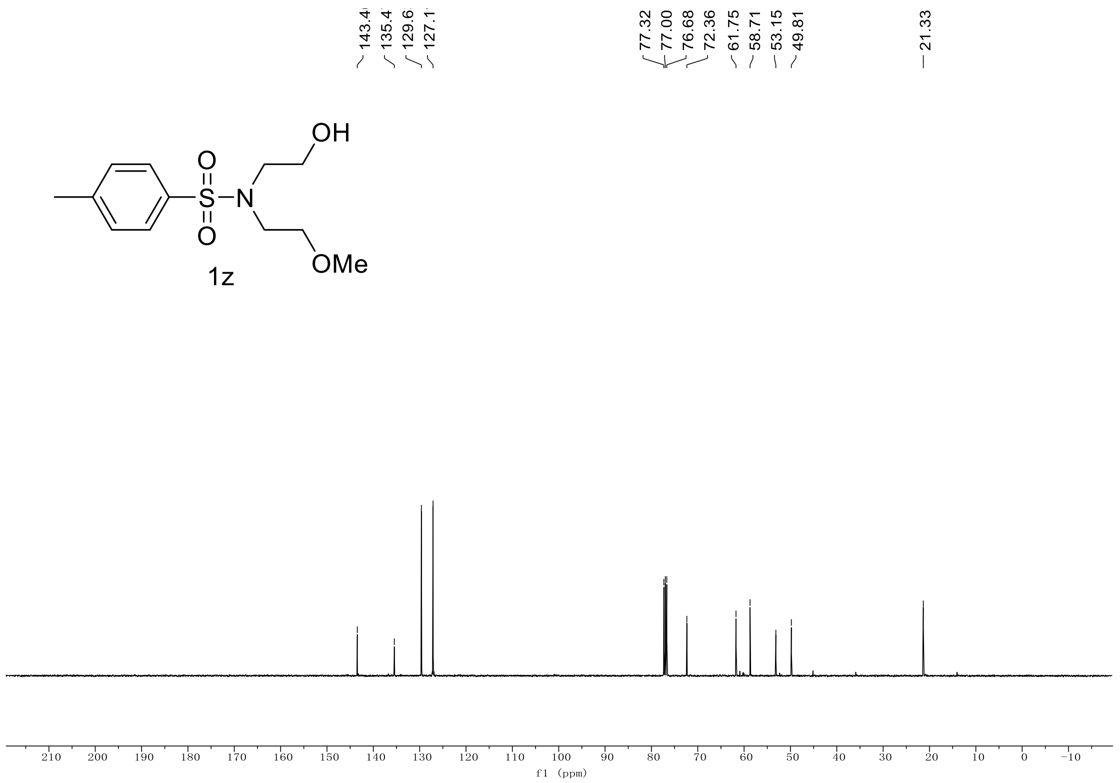

Supplementary Figure 84. ^{13}C NMR of compound **1z** (101 MHz, $CDCl_3$)

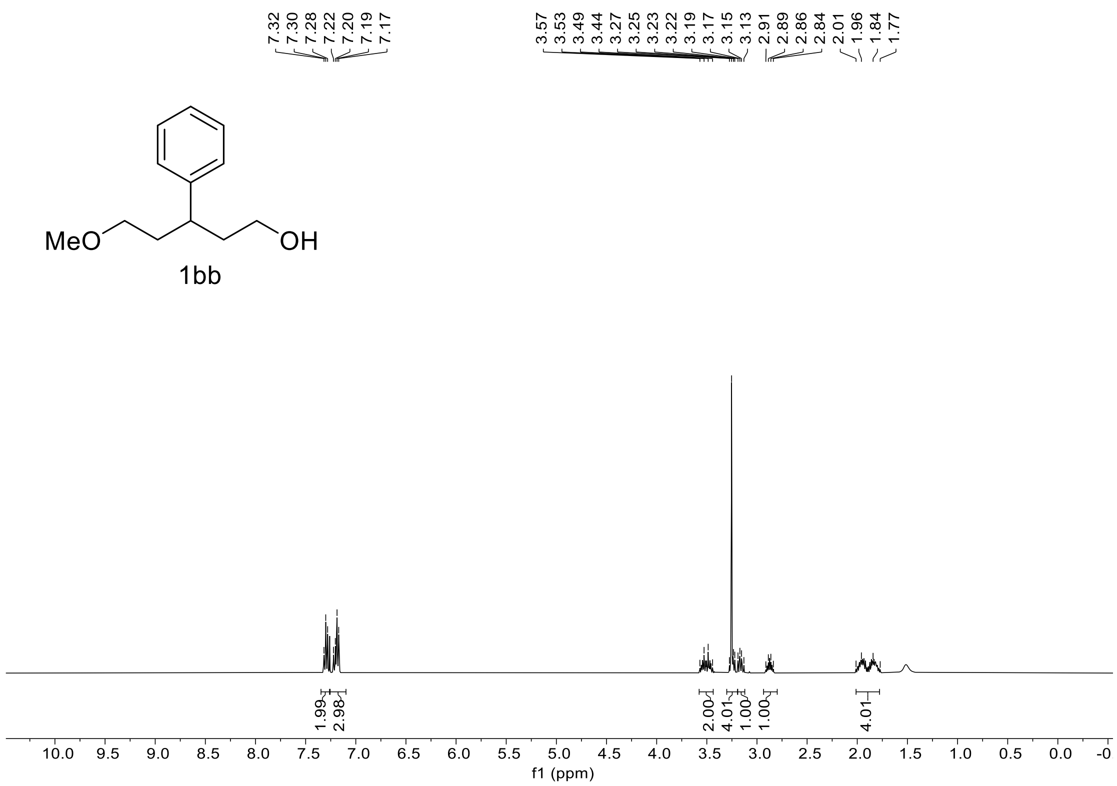

Supplementary Figure 85. ^{1}H NMR of compound **1bb** (400 MHz, $CDCl_3$)

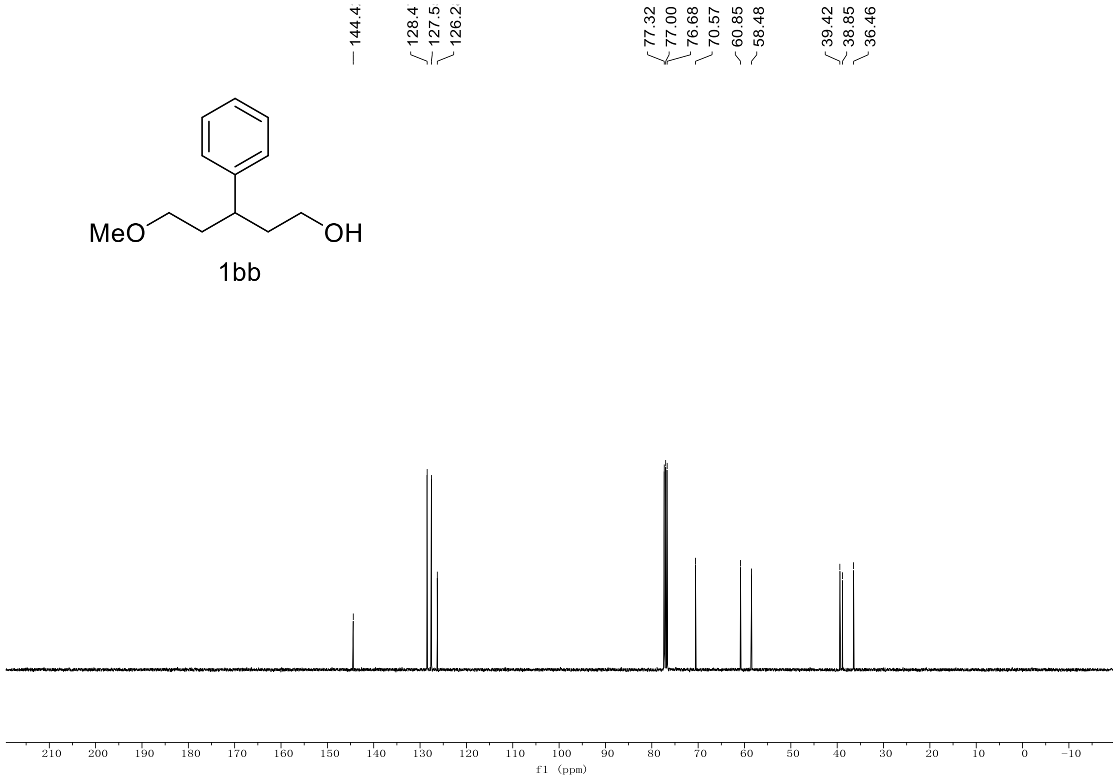

Supplementary Figure 86. ^{13}C NMR of compound **1bb** (101 MHz, $CDCl_3$)

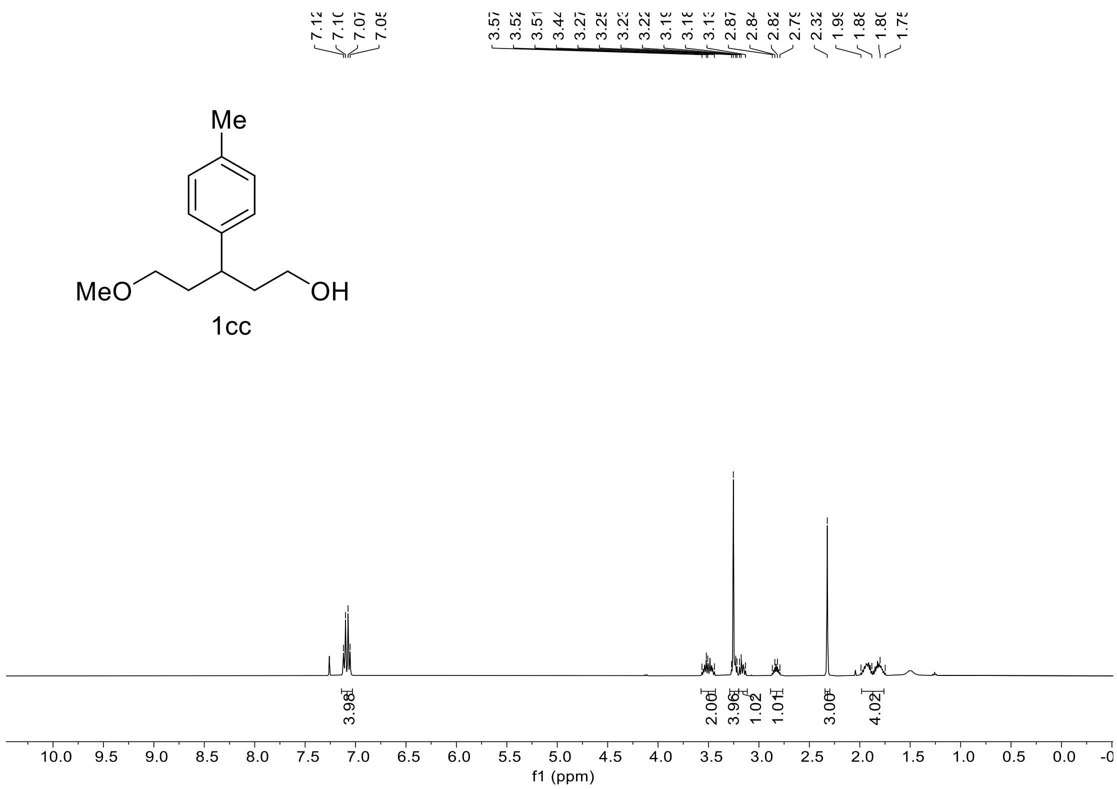

Supplementary Figure 87. ^{1}H NMR of compound **1cc** (400 MHz, $CDCl_3$)

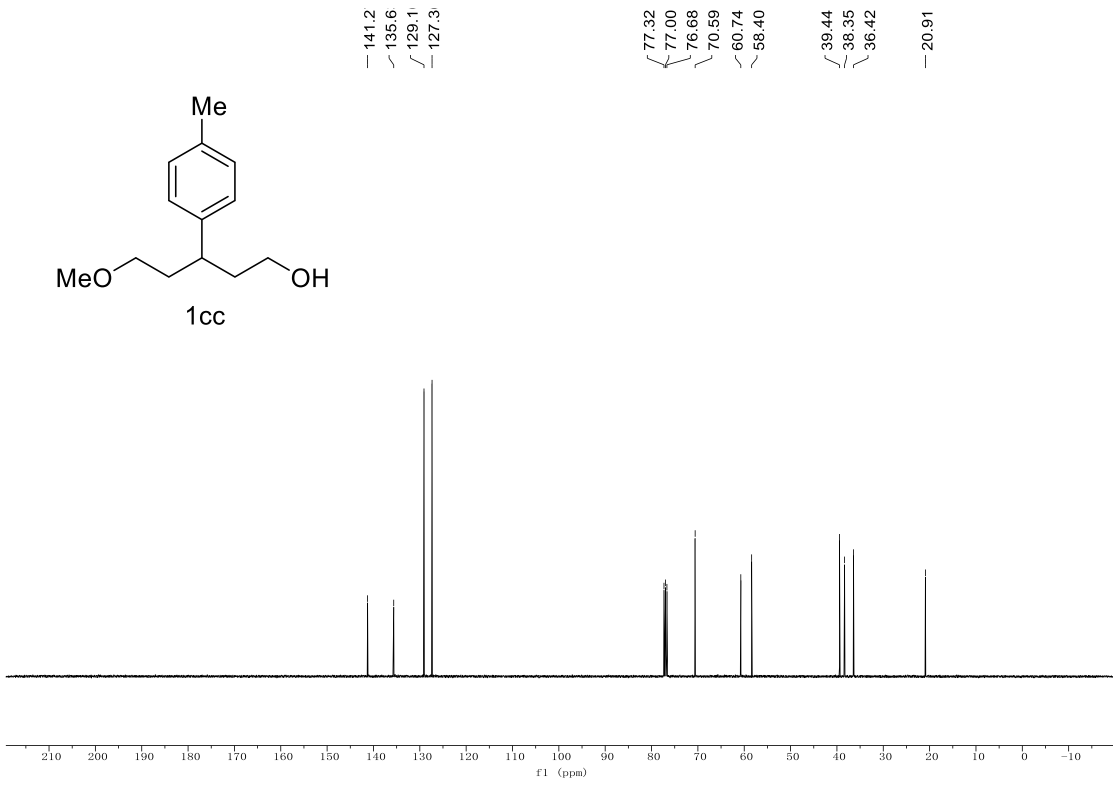

Supplementary Figure 88. ^{13}C NMR of compound **1cc** (101 MHz, $CDCl_3$)

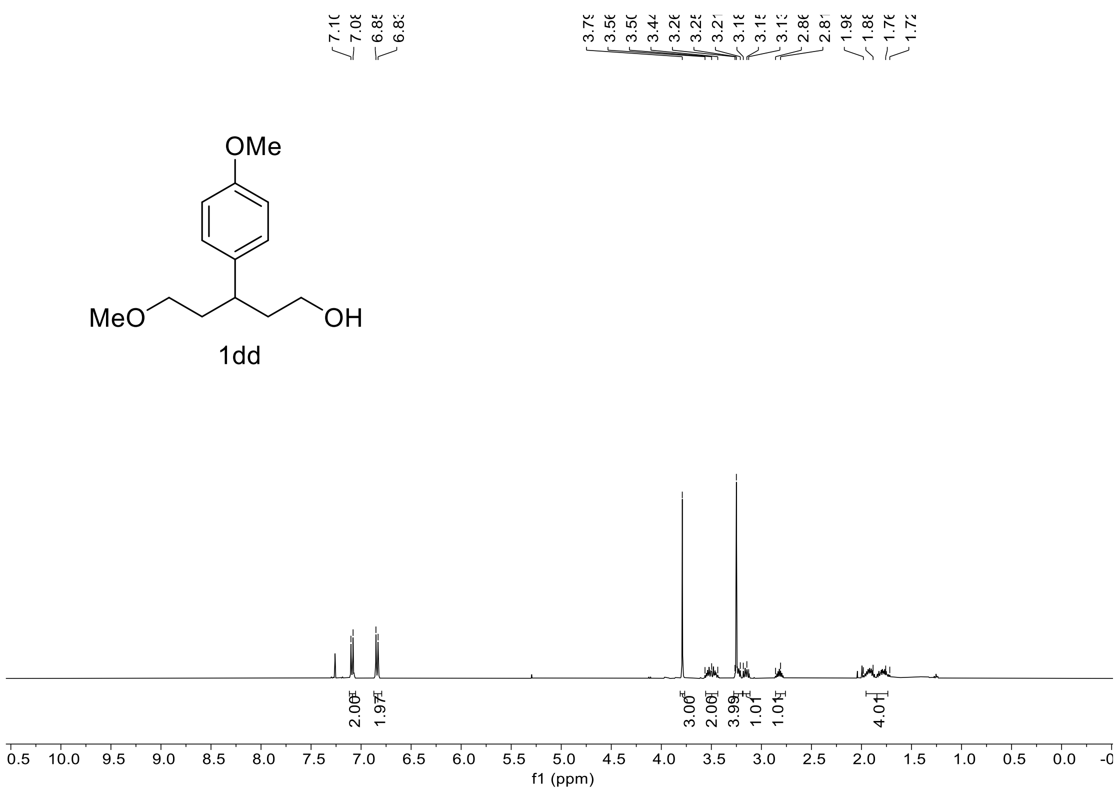

Supplementary Figure 89. ^{1}H NMR of compound **1dd** (400 MHz, $CDCl_3$)

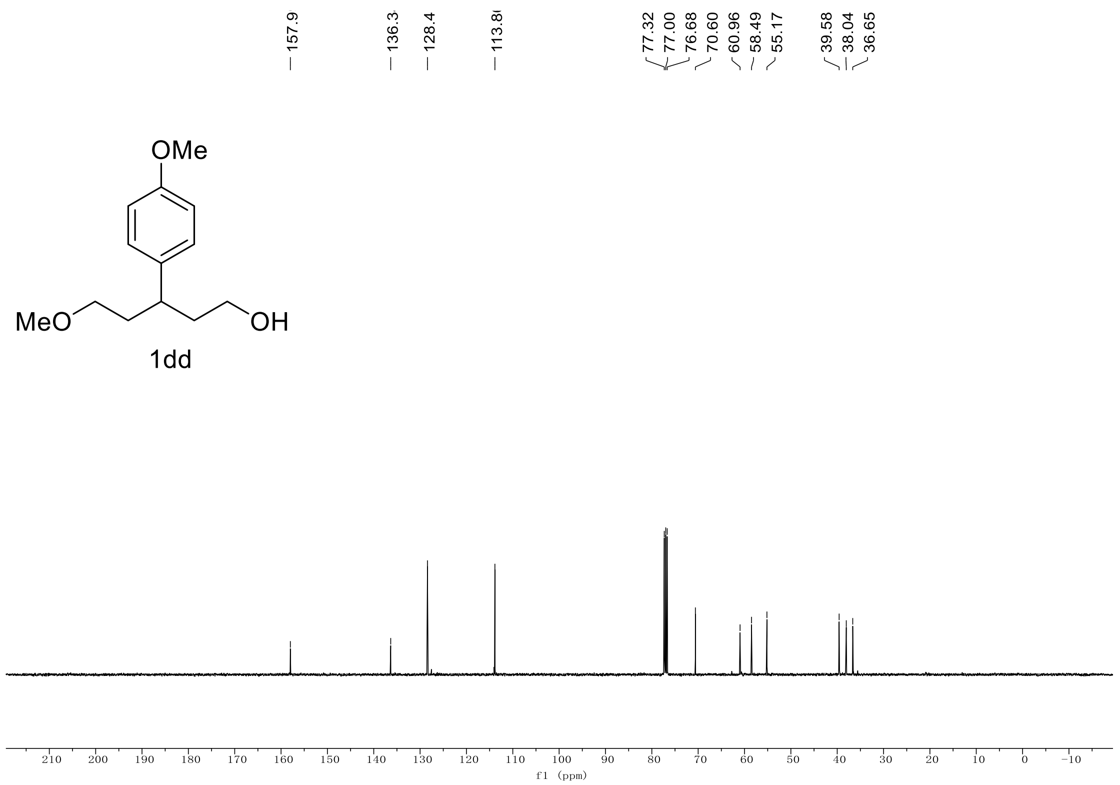

Supplementary Figure 90. ^{13}C NMR of compound **1dd** (101 MHz, $CDCl_3$)

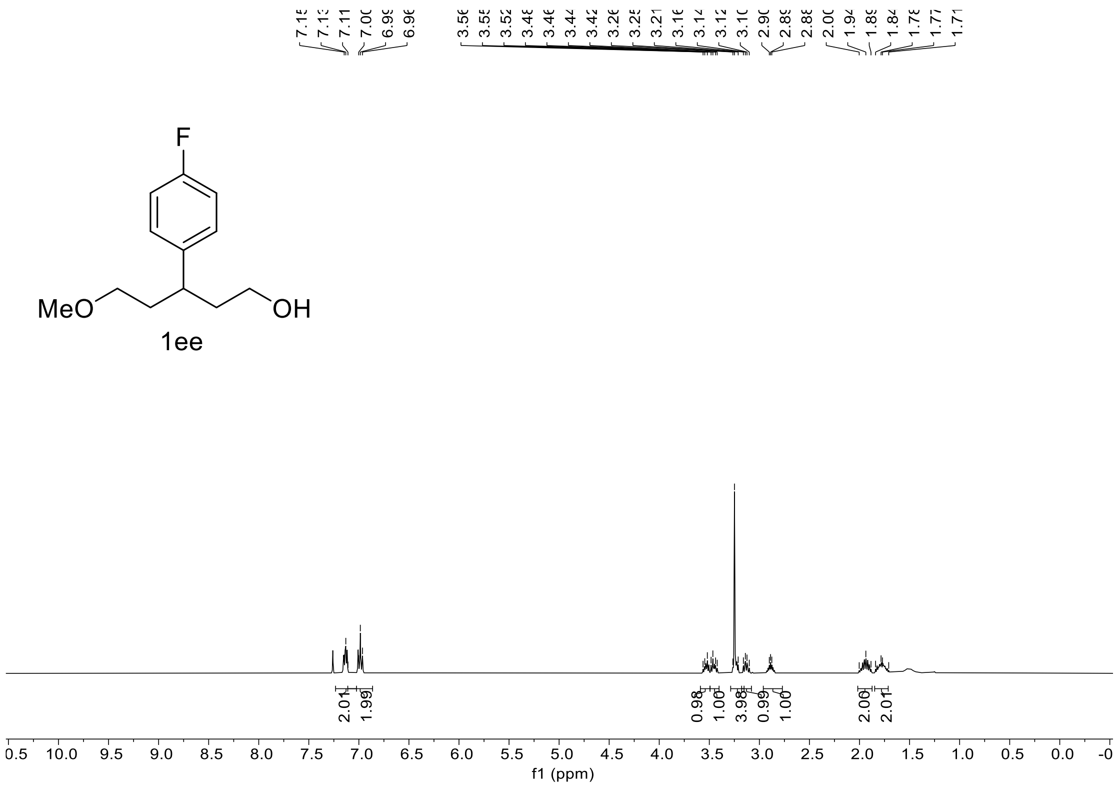

Supplementary Figure 91. ^{1}H NMR of compound **1ee** (400 MHz, $CDCl_3$)

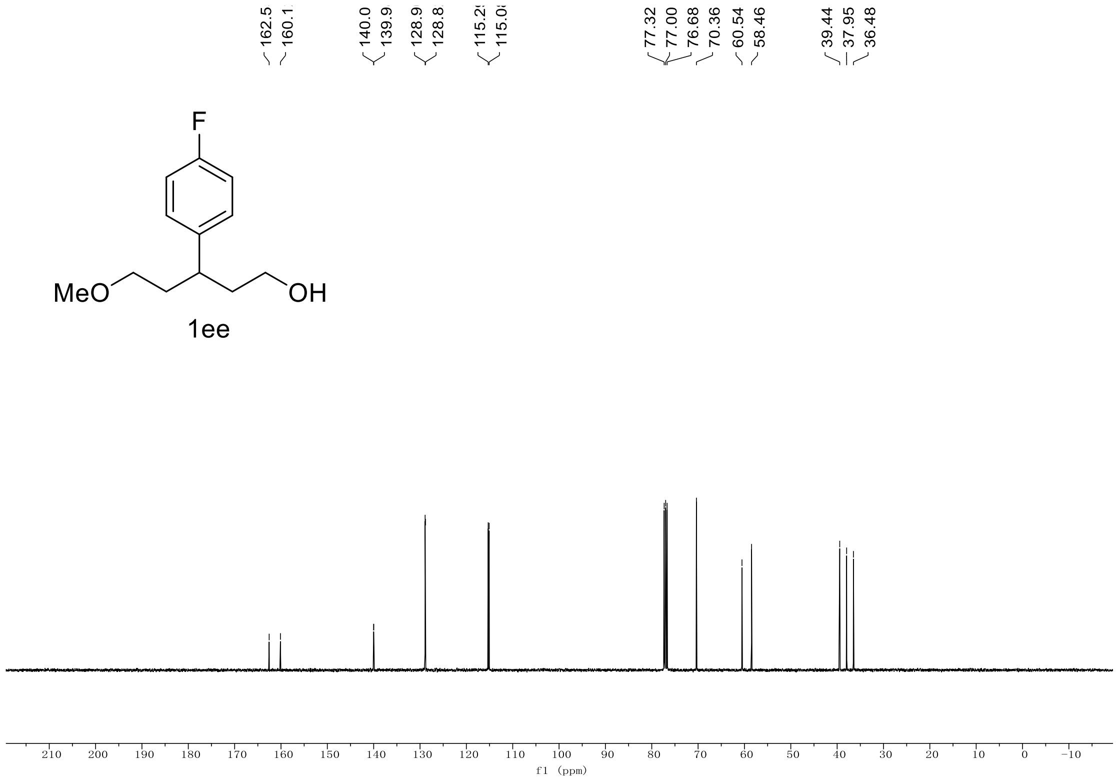

Supplementary Figure 92. ^{13}C NMR of compound **1ee** (101 MHz, $CDCl_3$)

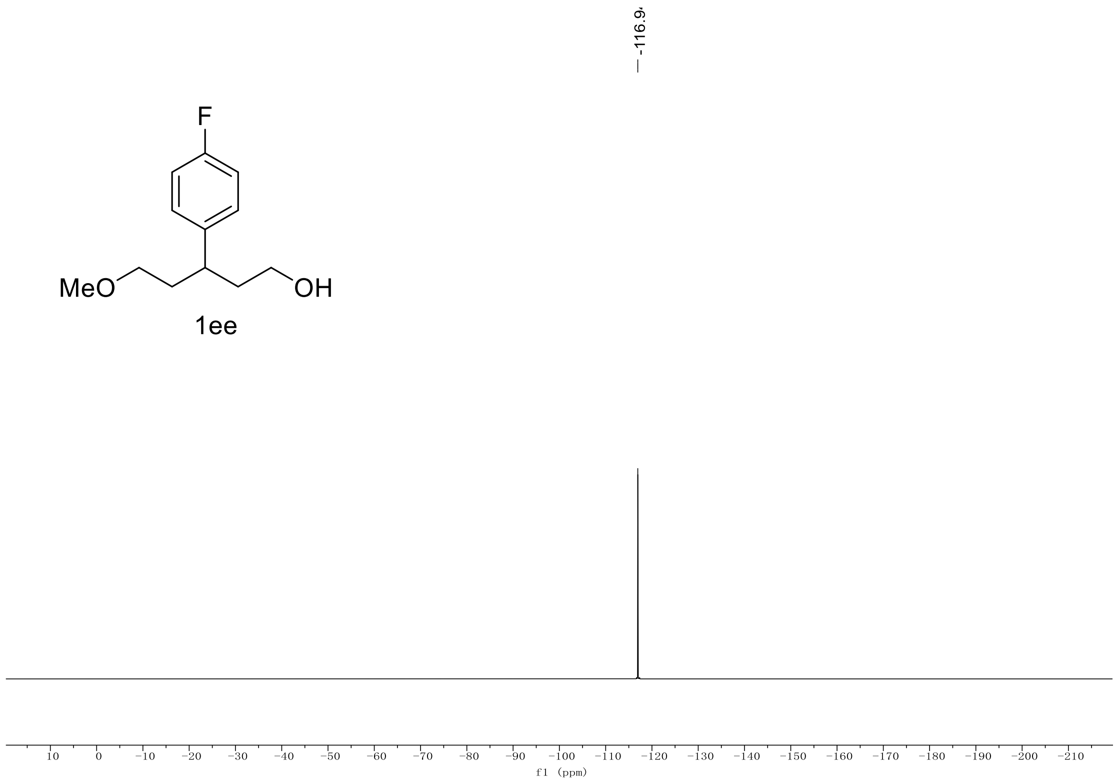

Supplementary Figure 93. ^{19}F NMR of compound **1ee** (565 MHz, $CDCl_3$)

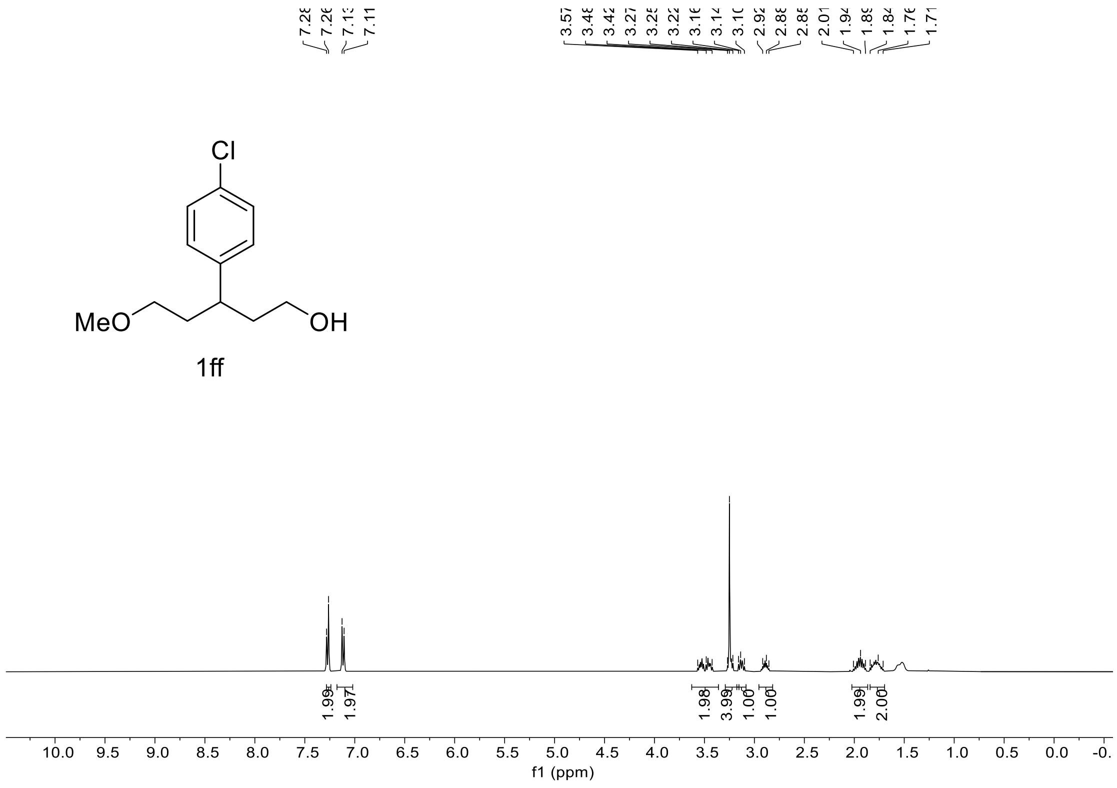

Supplementary Figure 94. ^{1}H NMR of compound **1ff** (400 MHz, $CDCl_3$)

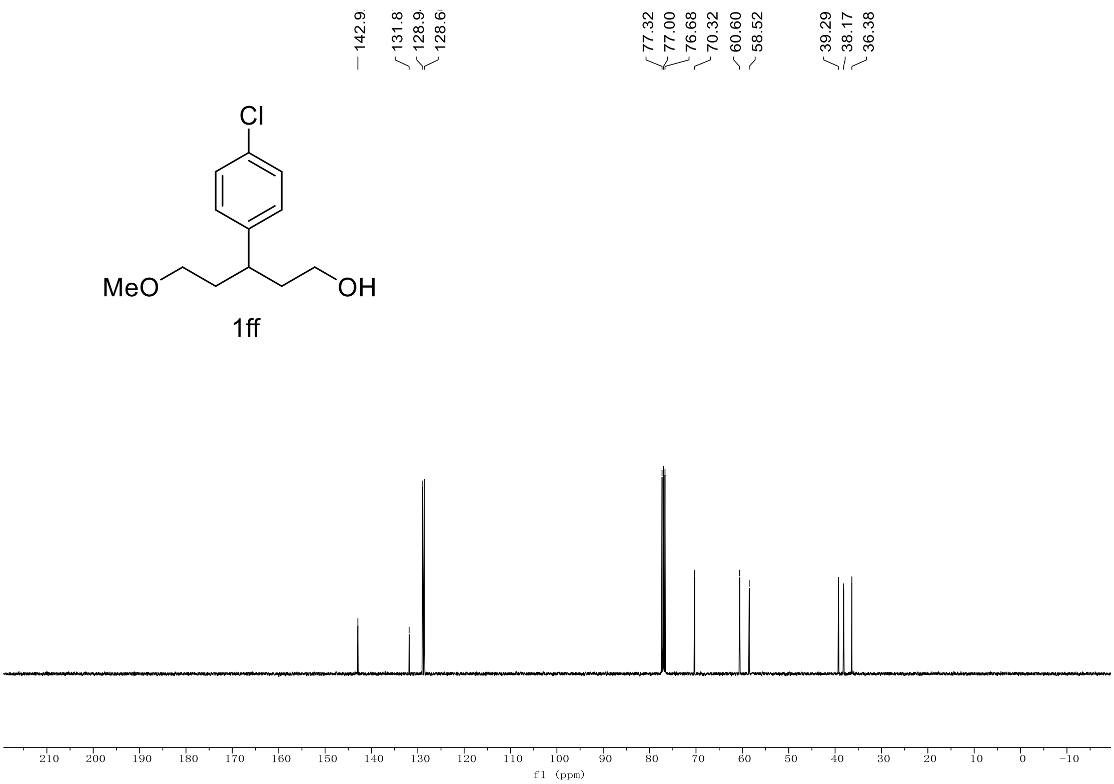

Supplementary Figure 95. ^{13}C NMR of compound **1ff** (101 MHz, $CDCl_3$)

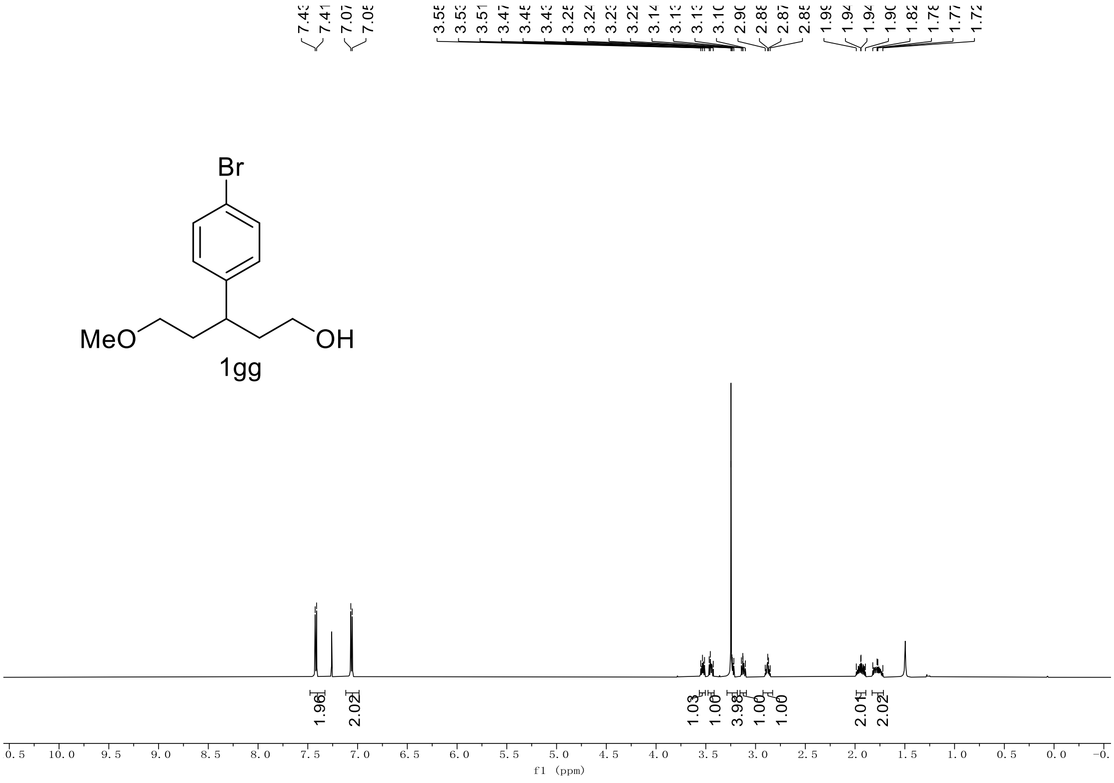

Supplementary Figure 96. ^{1}H NMR of compound **1gg** (600 MHz, $CDCl_3$)

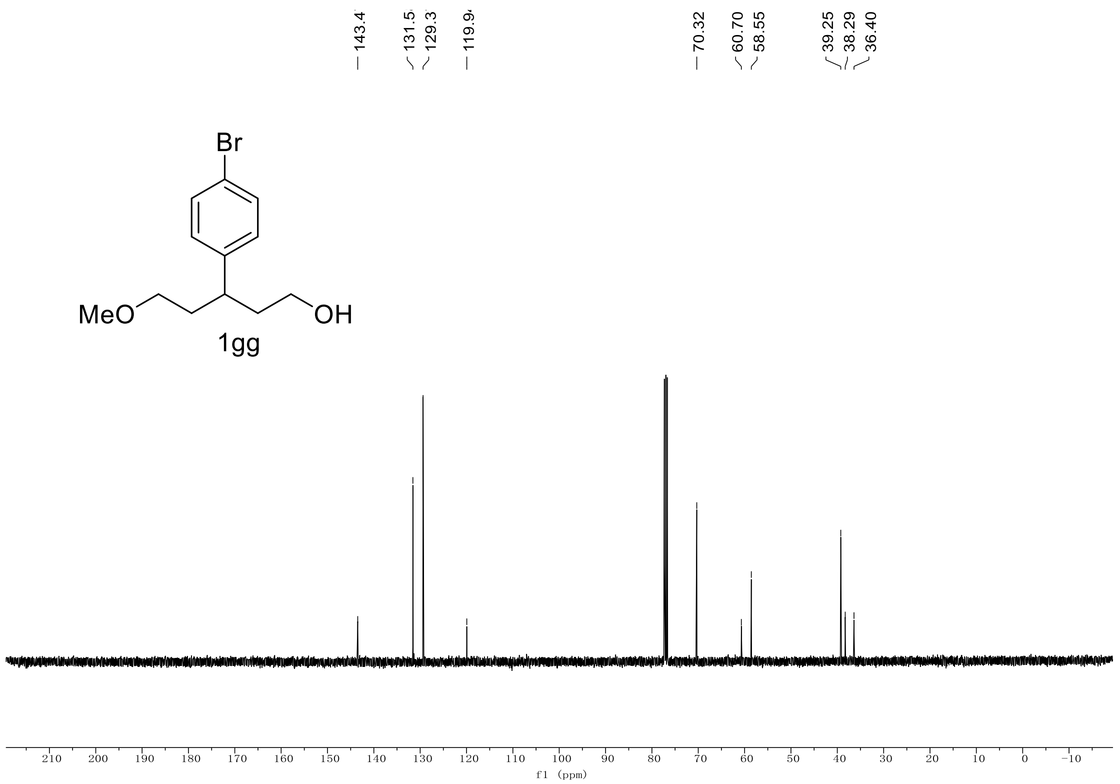

Supplementary Figure 97 ^{13}C NMR of compound **1gg** (101 MHz, $CDCl_3$)

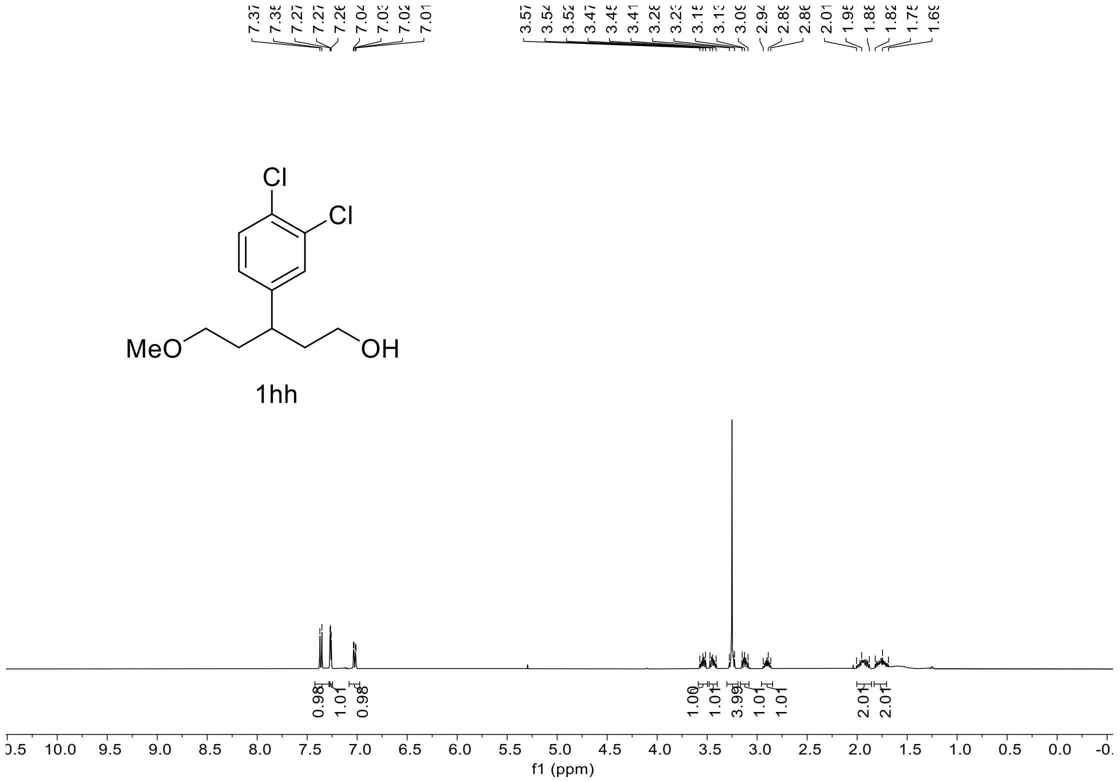

Supplementary Figure 98. ^{1}H NMR of compound **1hh** (400 MHz, $CDCl_3$)

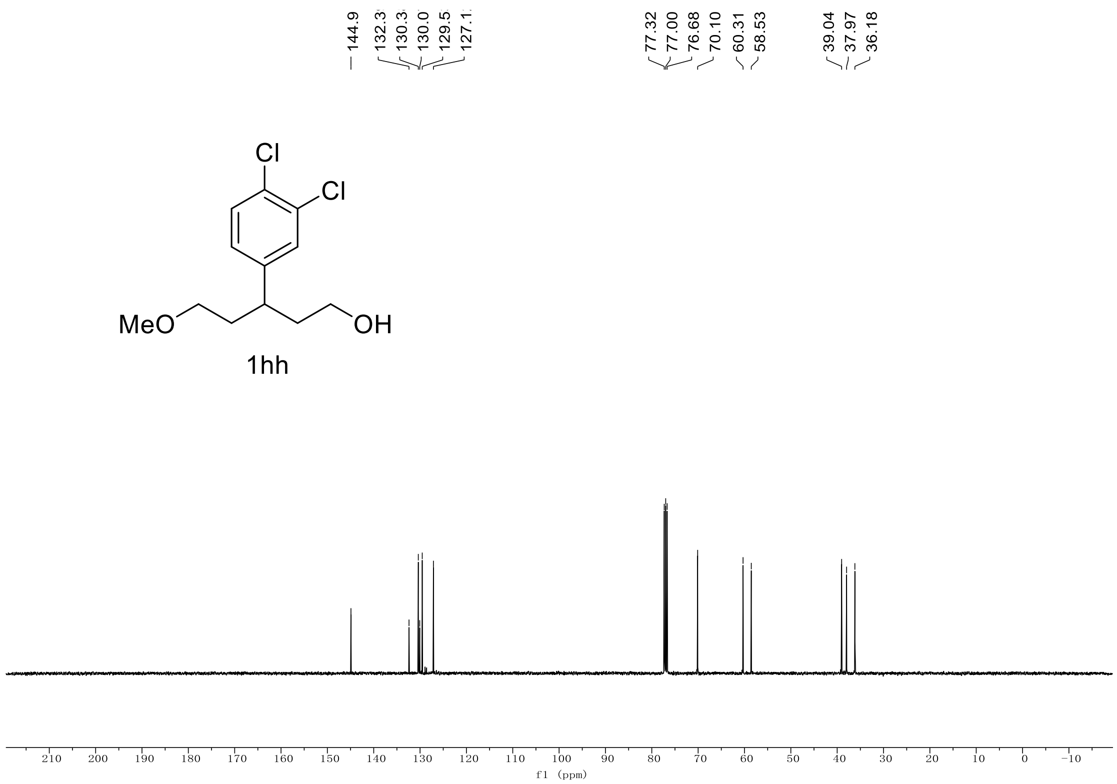

Supplementary Figure 99. ^{13}C NMR of compound **1hh**(101 MHz, $CDCl_3$

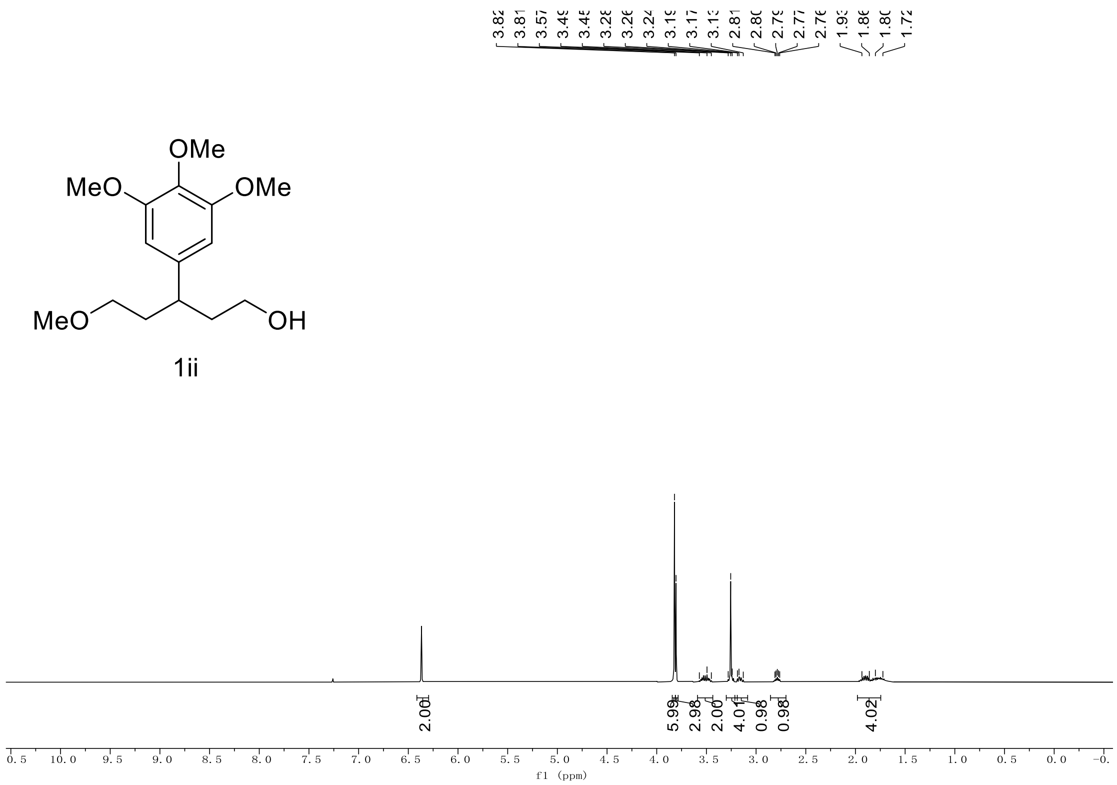

Supplementary Figure 100. ^{1}H NMR of compound **1ii** (400 MHz, $CDCl_3$)

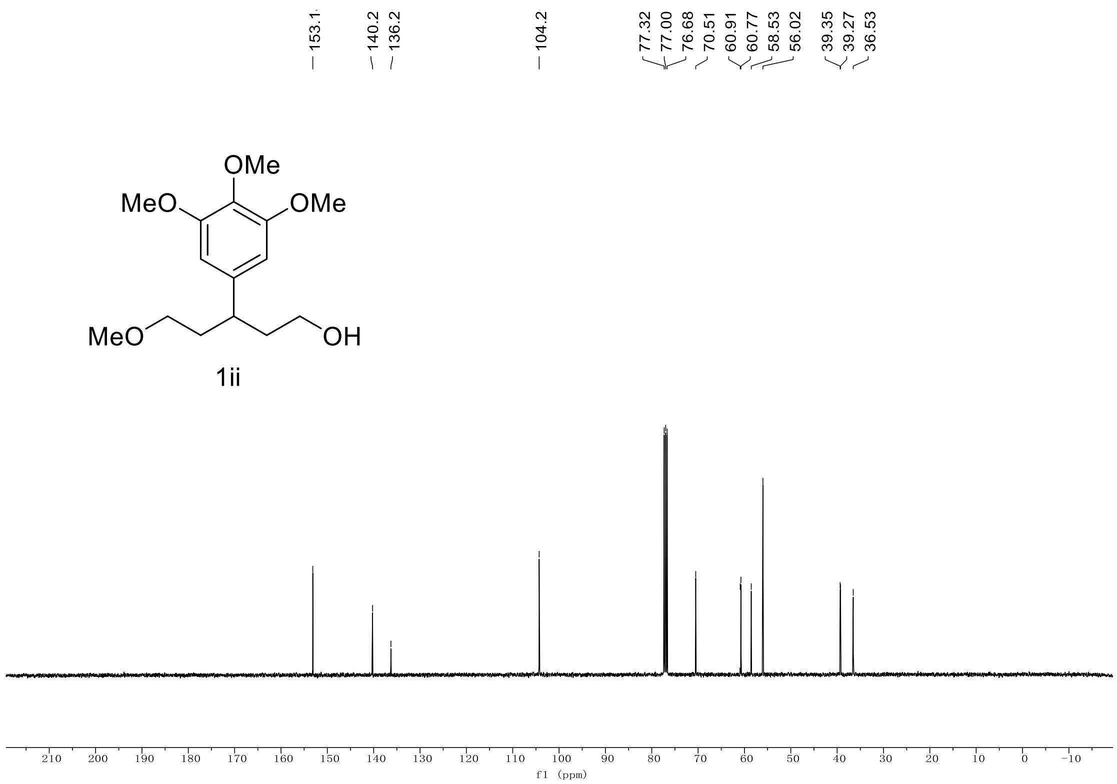

Supplementary Figure 101. ^{13}C NMR of compound **1ii** (101 MHz, $CDCl_3$)

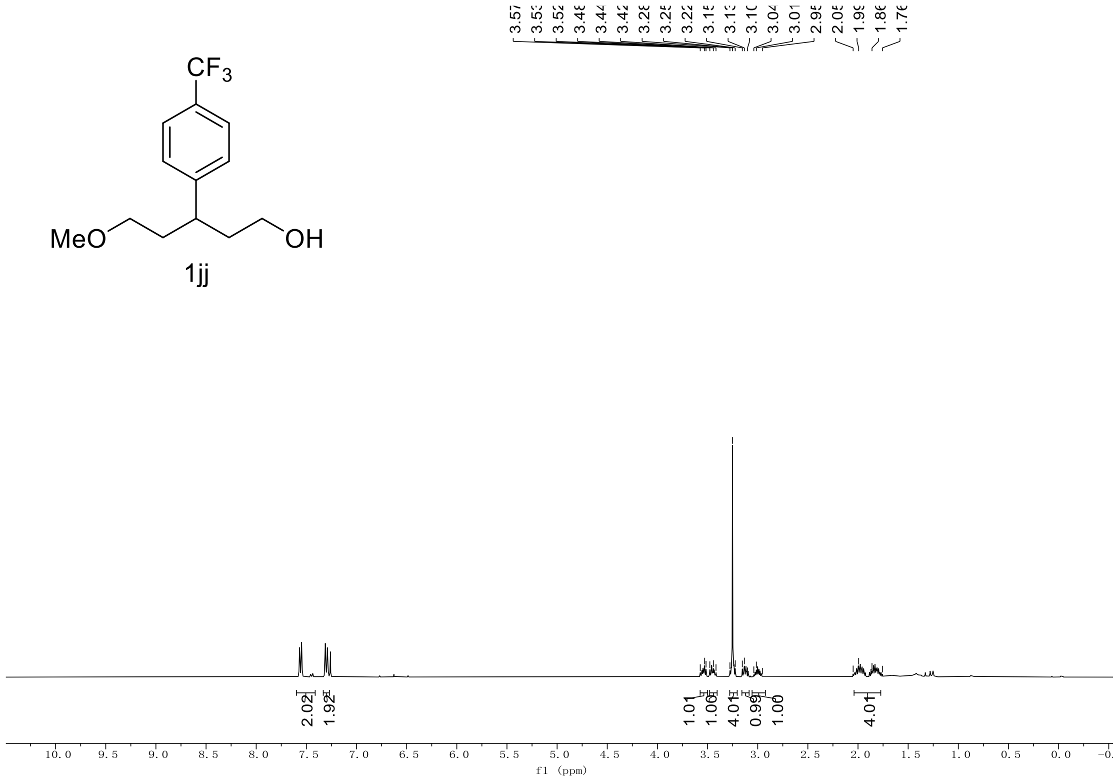

Supplementary Figure 102. ^{1}H NMR of compound **1jj** (400 MHz, $CDCl_3$)

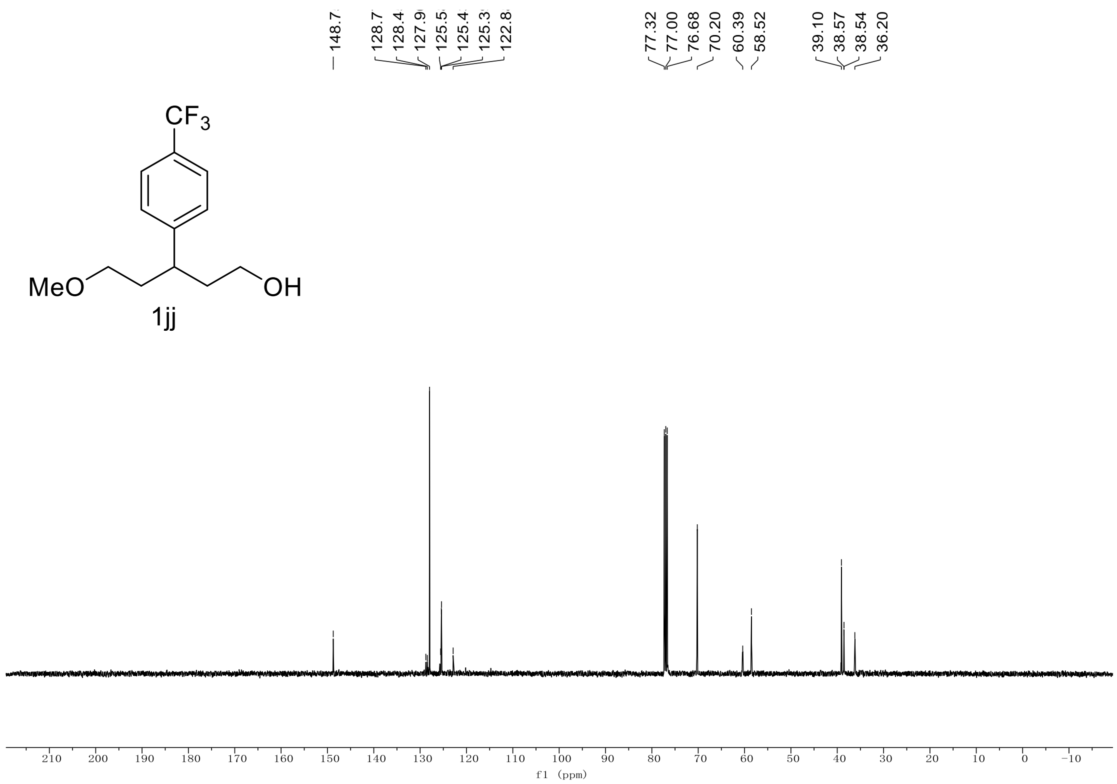

Supplementary Figure 103. ^{13}C NMR of compound **1jj** (101 MHz, $CDCl_3$)

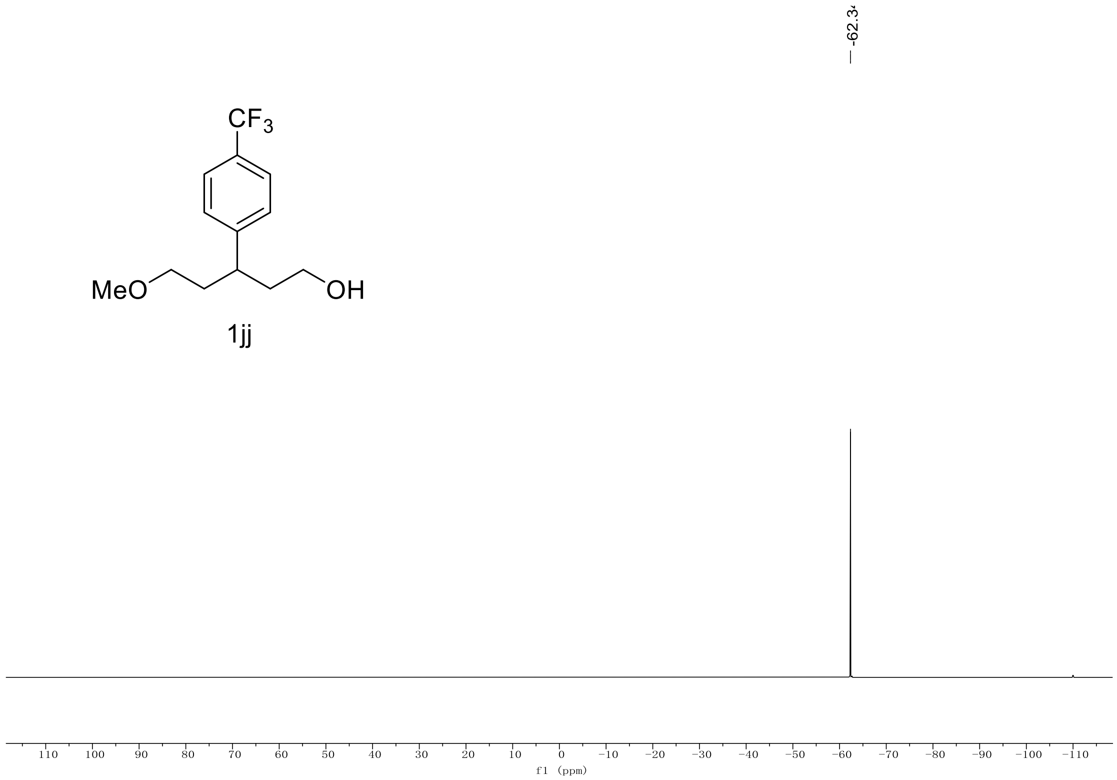

Supplementary Figure 104. ^{19}F NMR of compound **1jj** (565 MHz, $CDCl_3$)

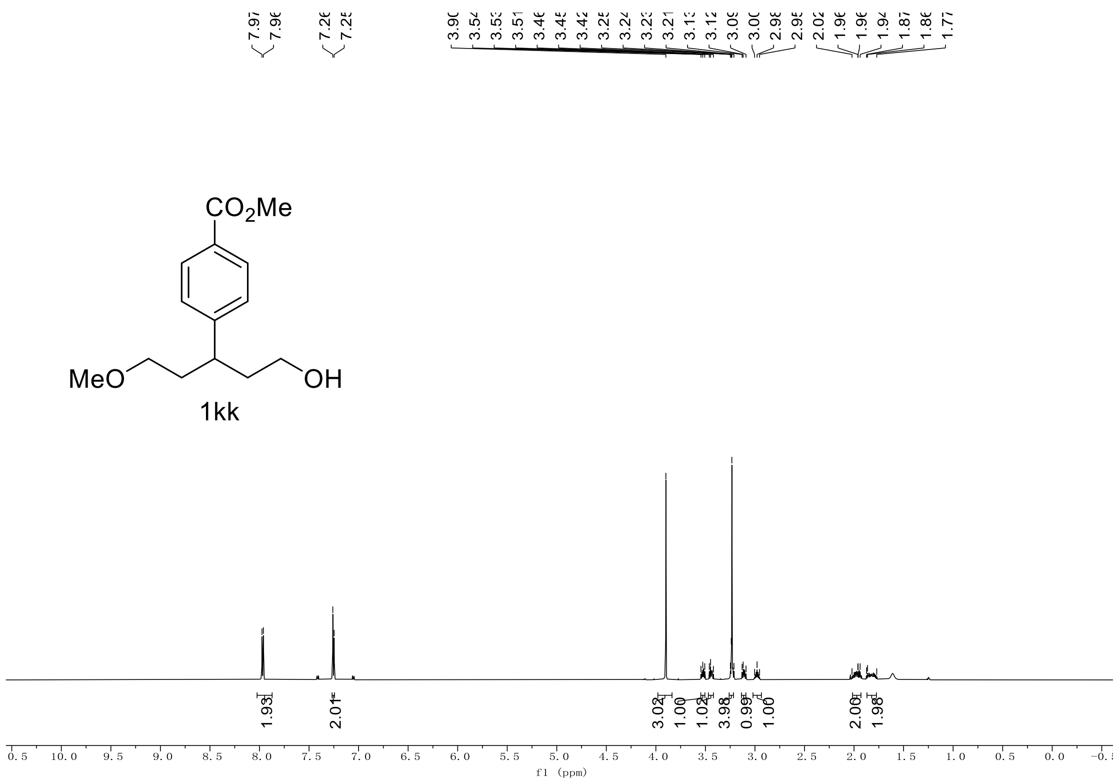

Supplementary Figure 105. ^{1}H NMR of compound **1kk** (600 MHz, $CDCl_3$)

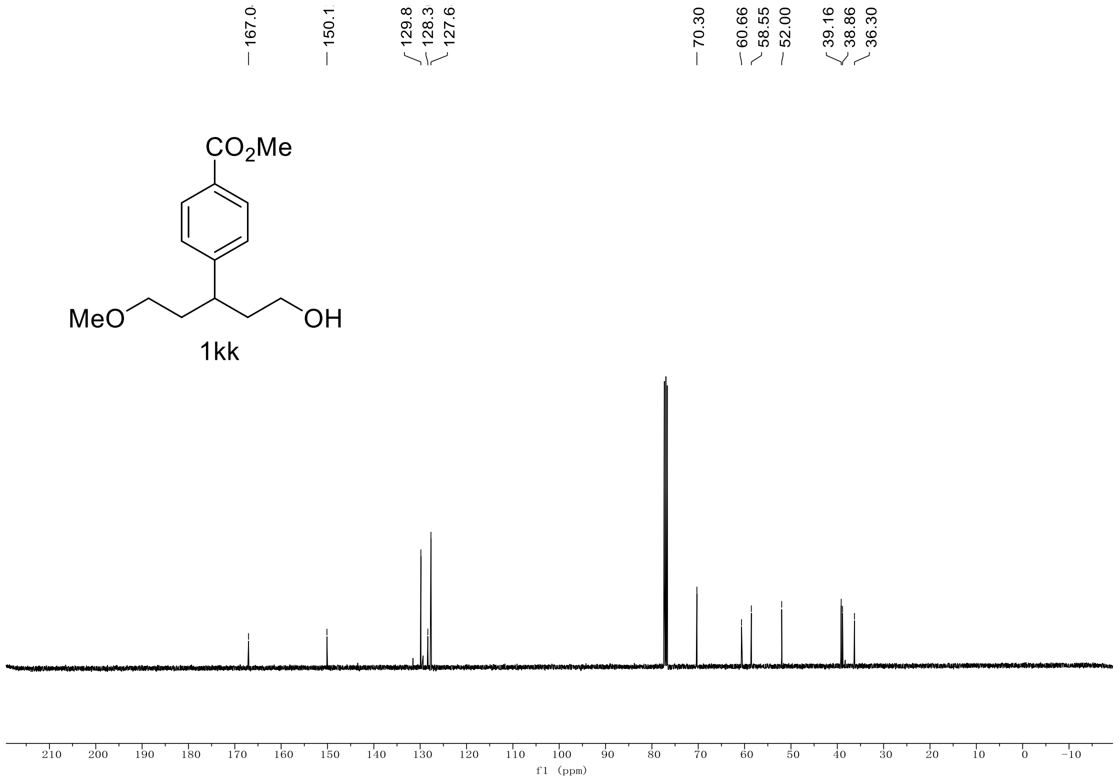

Supplementary Figure 106. ^{13}C NMR of compound **1kk** (101 MHz, $CDCl_3$)

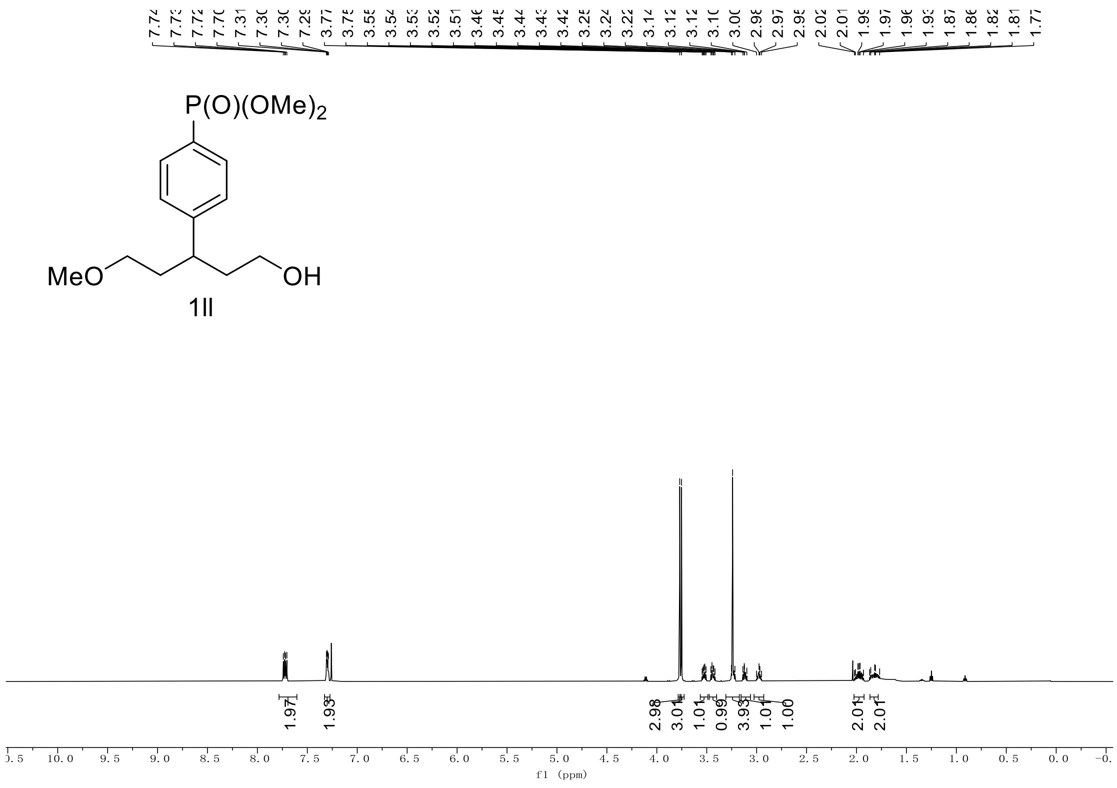

Supplementary Figure 107. ^{1}H NMR of compound **1ll** (600 MHz, $CDCl_3$)

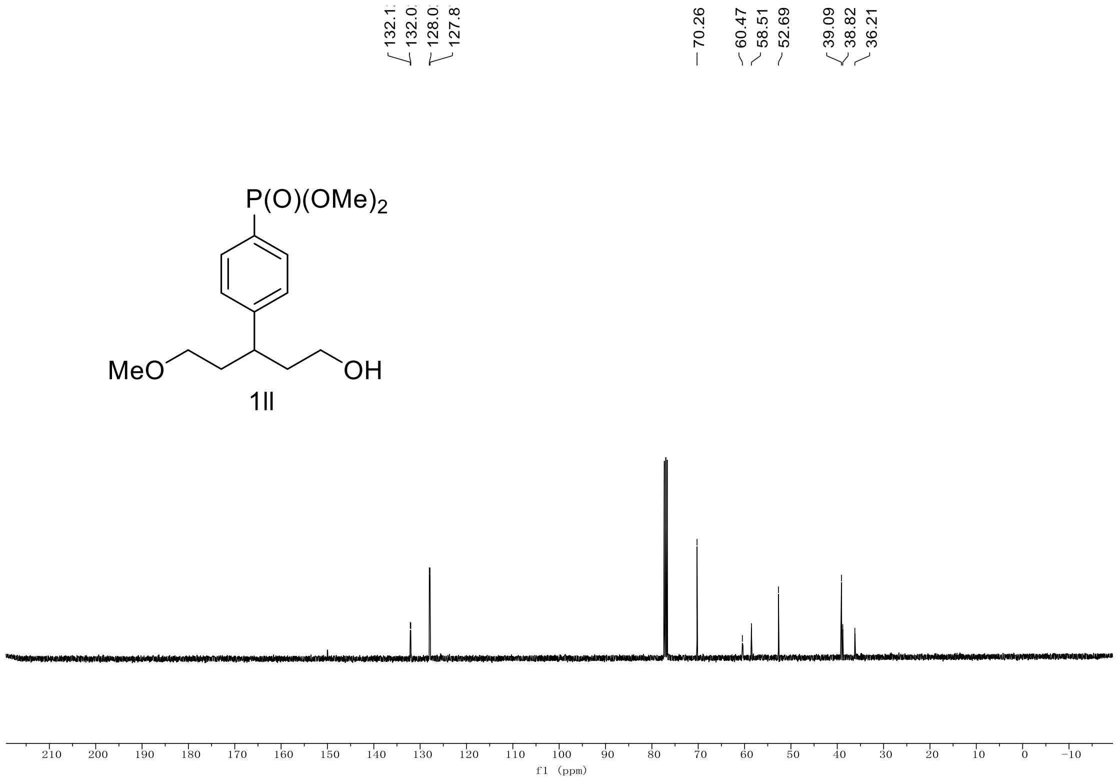

Supplementary Figure 108. ^{13}C NMR of compound **1ll** (101 MHz, $CDCl_3$)

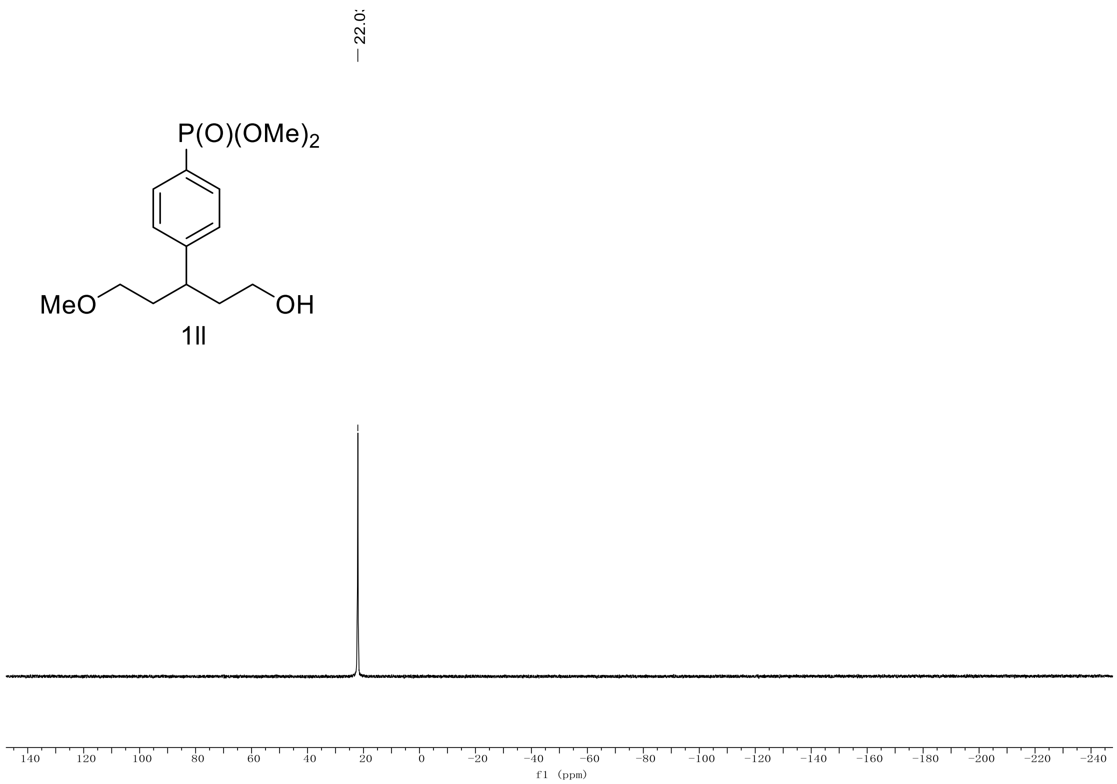

Supplementary Figure 109. ^{31}P NMR of compound **1ll** (243 MHz, $CDCl_3$)

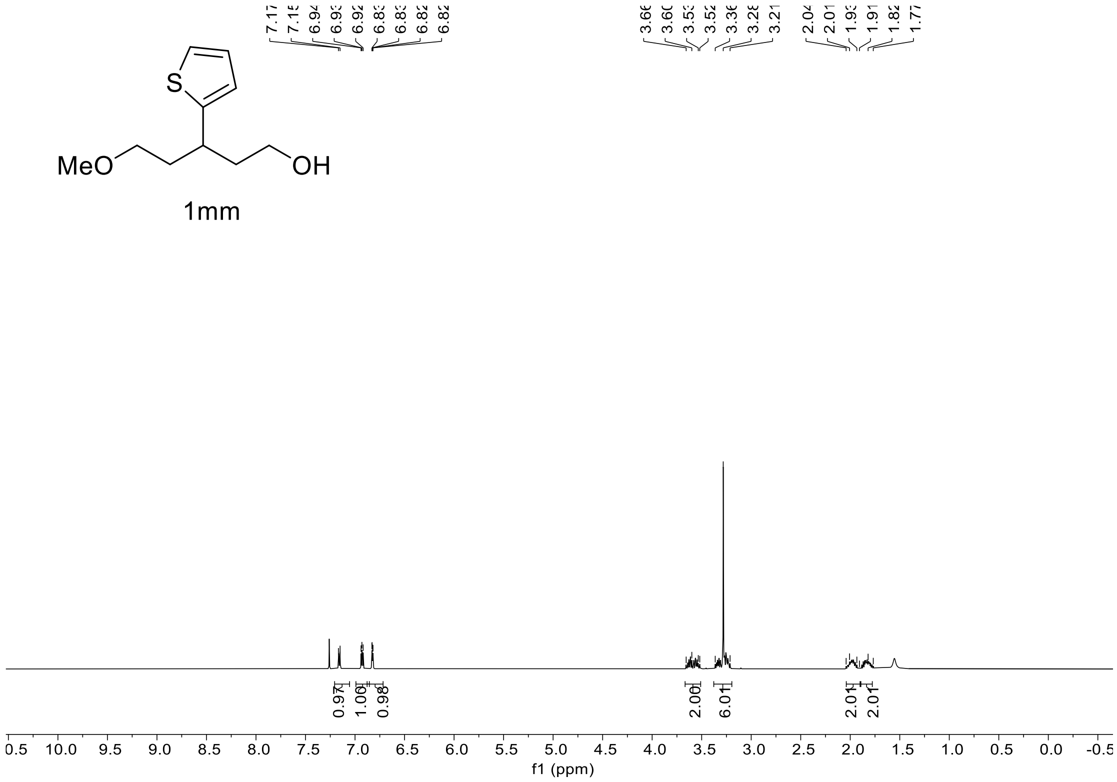

Supplementary Figure 110. ^{1}H NMR of compound **1mm** (400 MHz, $CDCl_3$)

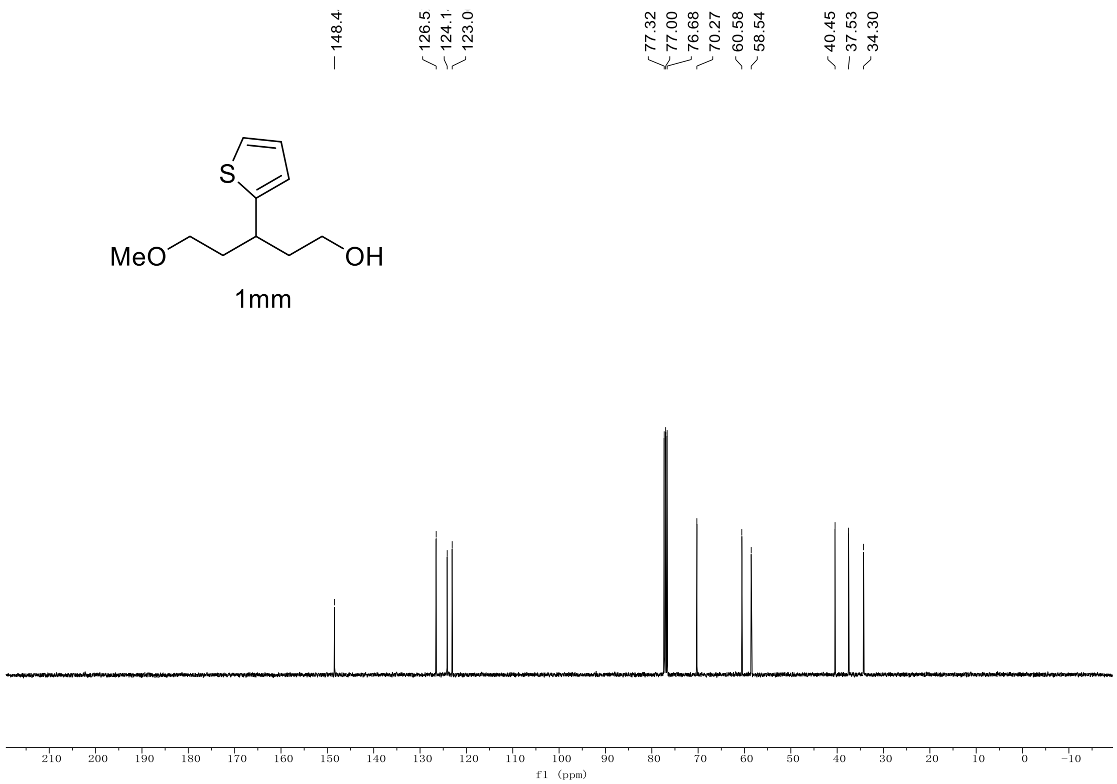

Supplementary Figure 111. ^{13}C NMR of compound **1mm** (101 MHz, $CDCl_3$)

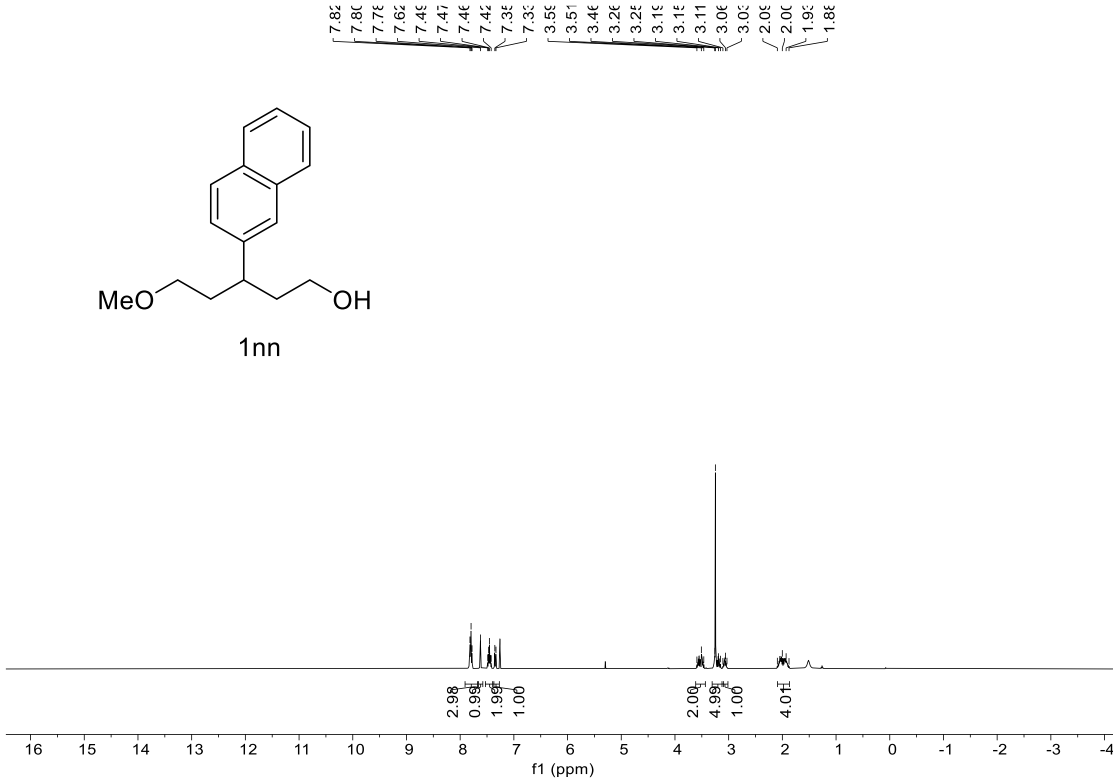

Supplementary Figure 112. ^{1}H NMR of compound **1nn** (400 MHz, $CDCl_3$)

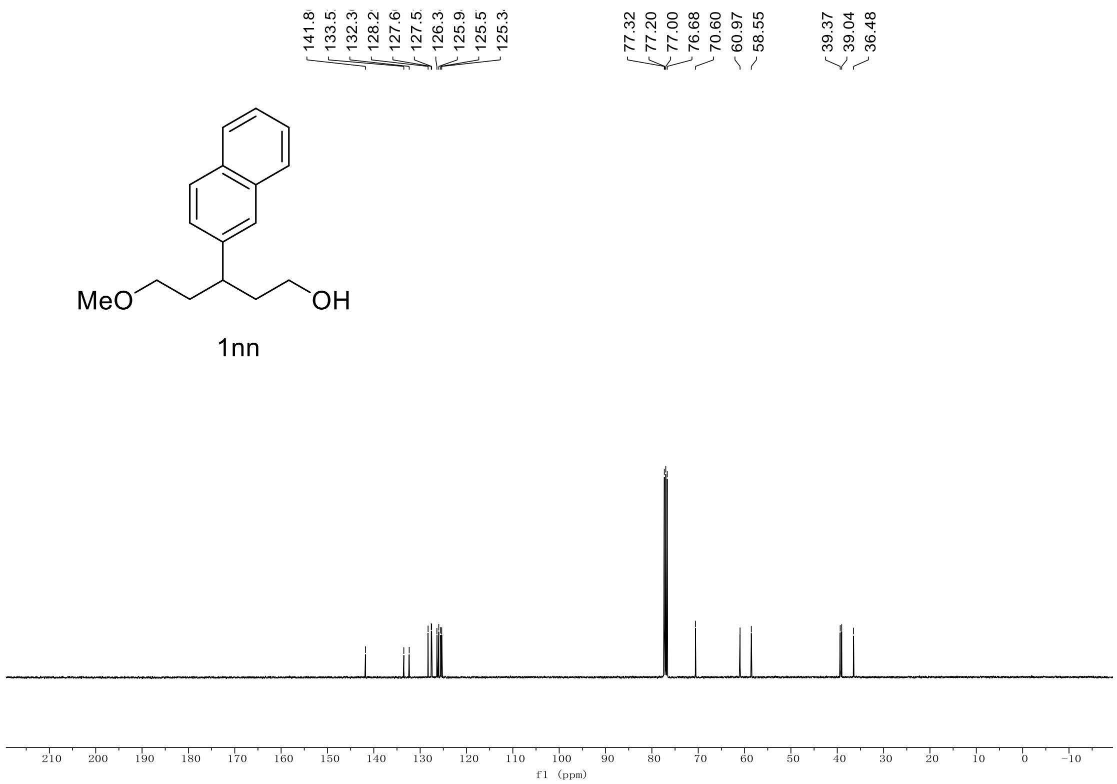

Supplementary Figure 113. ^{13}C NMR of compound **1nn** (101 MHz, $CDCl_3$)

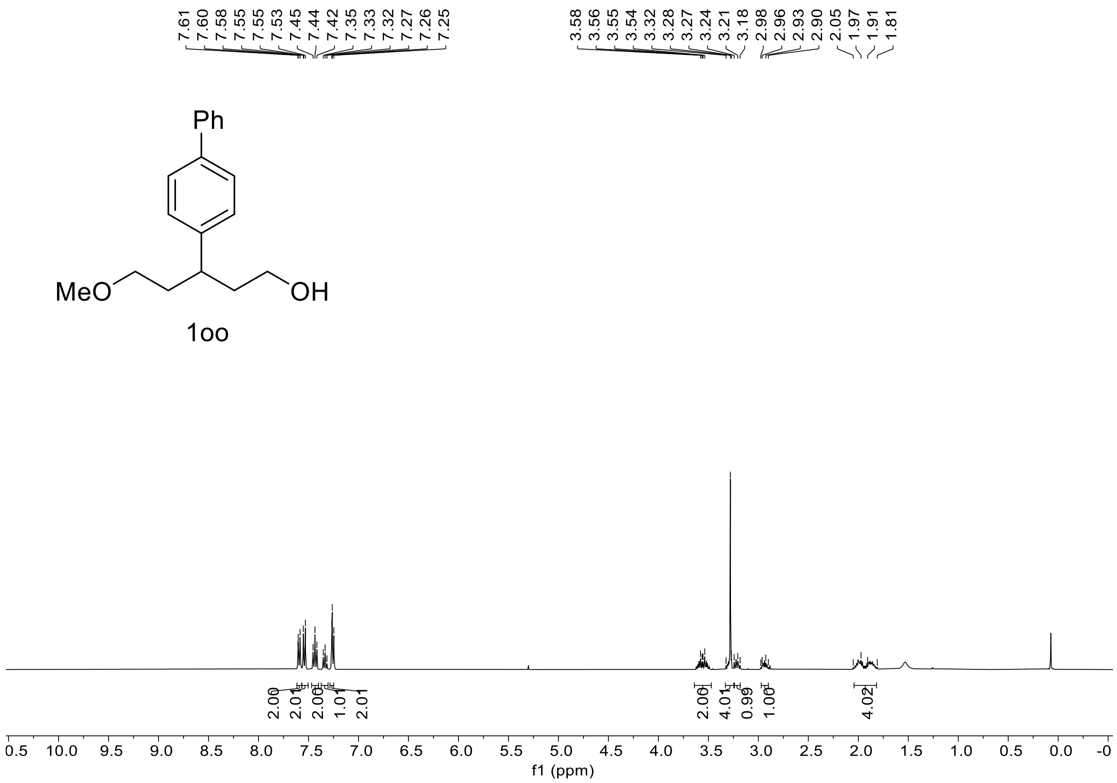

Supplementary Figure 114. ^{1}H NMR of compound **1oo** (400 MHz, $CDCl_3$)

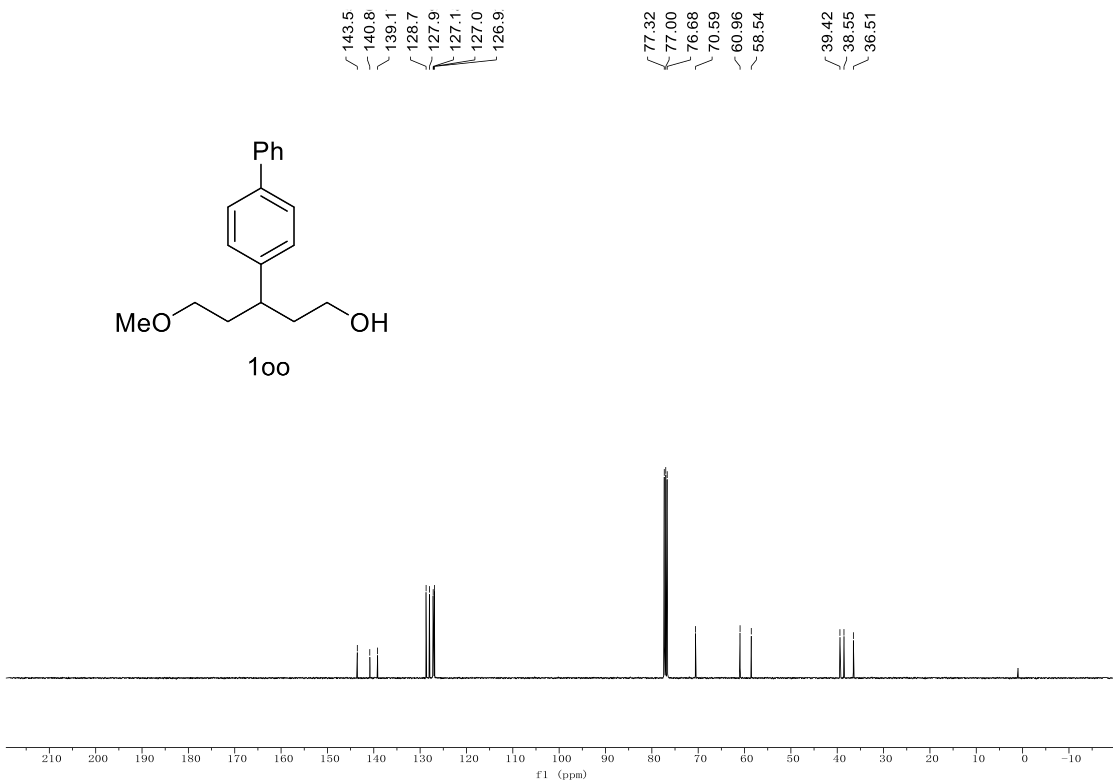

Supplementary Figure 115. ^{13}C NMR of compound **1oo** (101 MHz, $CDCl_3$)

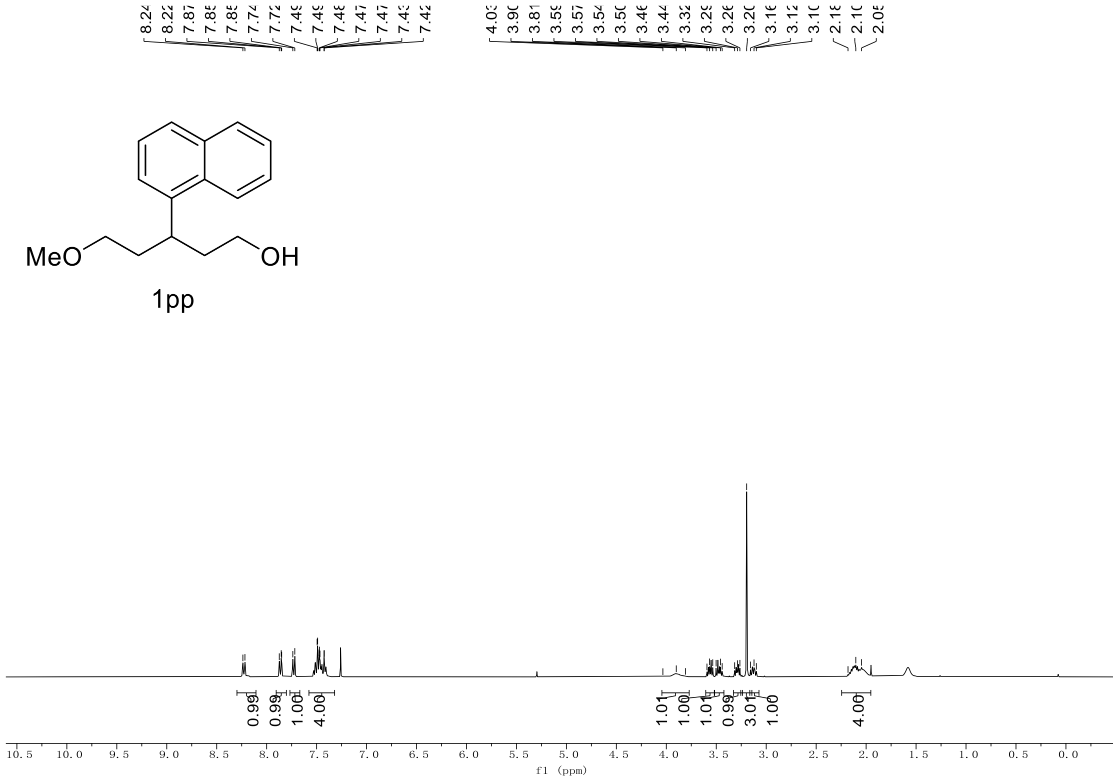

Supplementary Figure 116. ^{1}H NMR of compound **1pp** (400 MHz, $CDCl_3$)

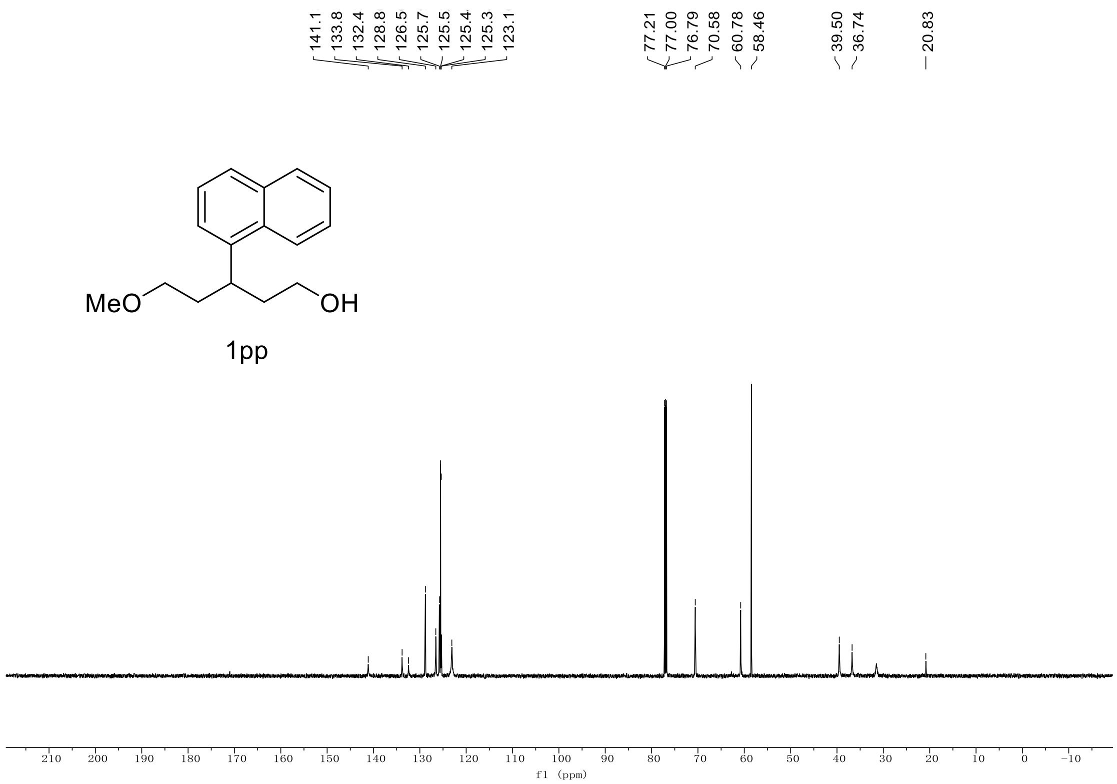

Supplementary Figure 117. ^{13}C NMR of compound **1pp** (151 MHz, $CDCl_3$)

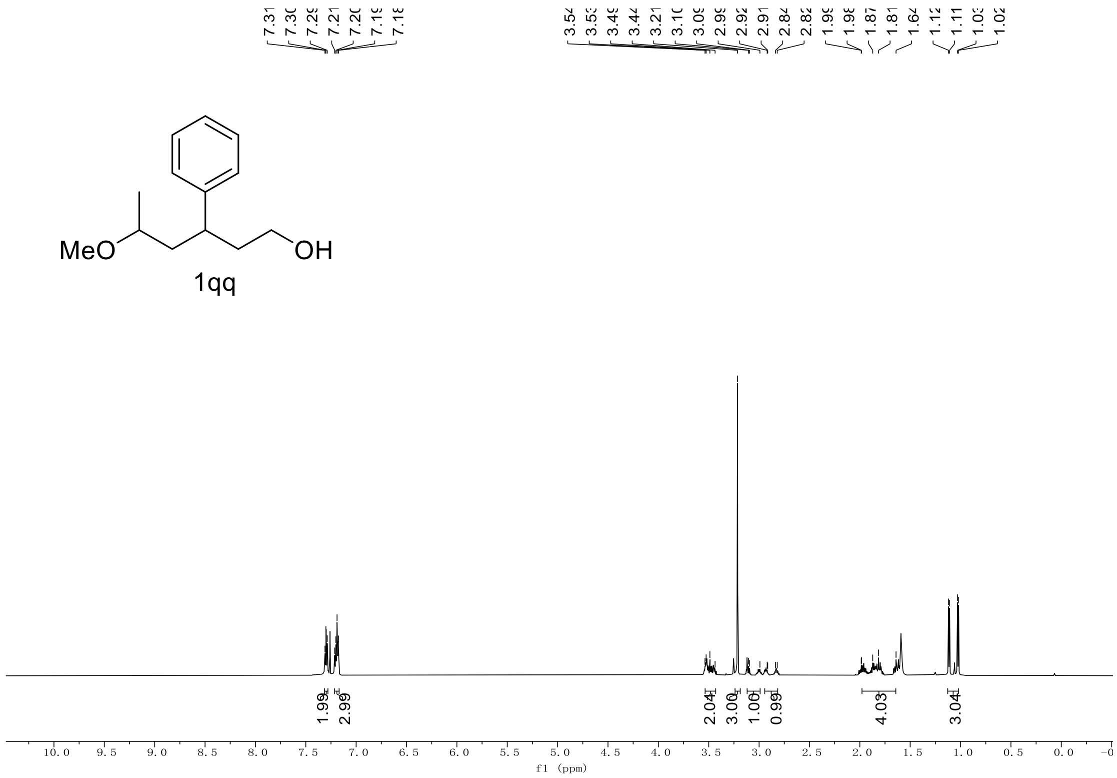

Supplementary Figure 118. ^{1}H NMR of compound **1qq** (600 MHz, $CDCl_3$)

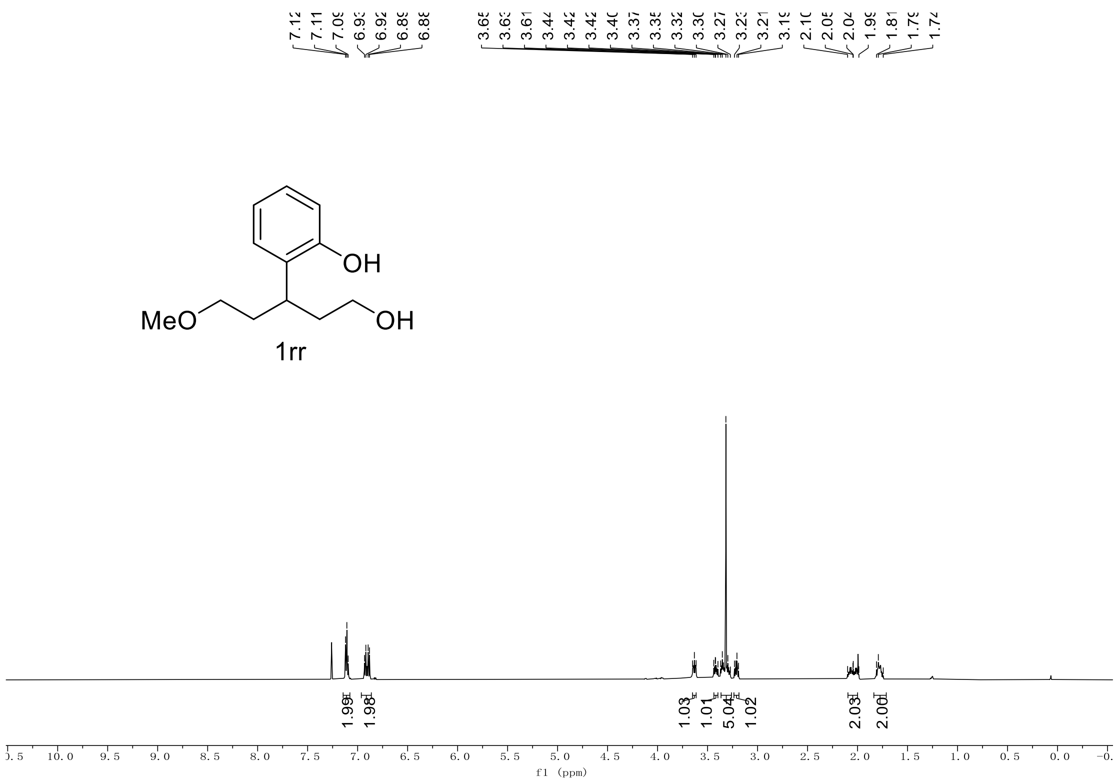

Supplementary Figure 119. ^{1}H NMR of compound **1rr** (600 MHz, $CDCl_3$)

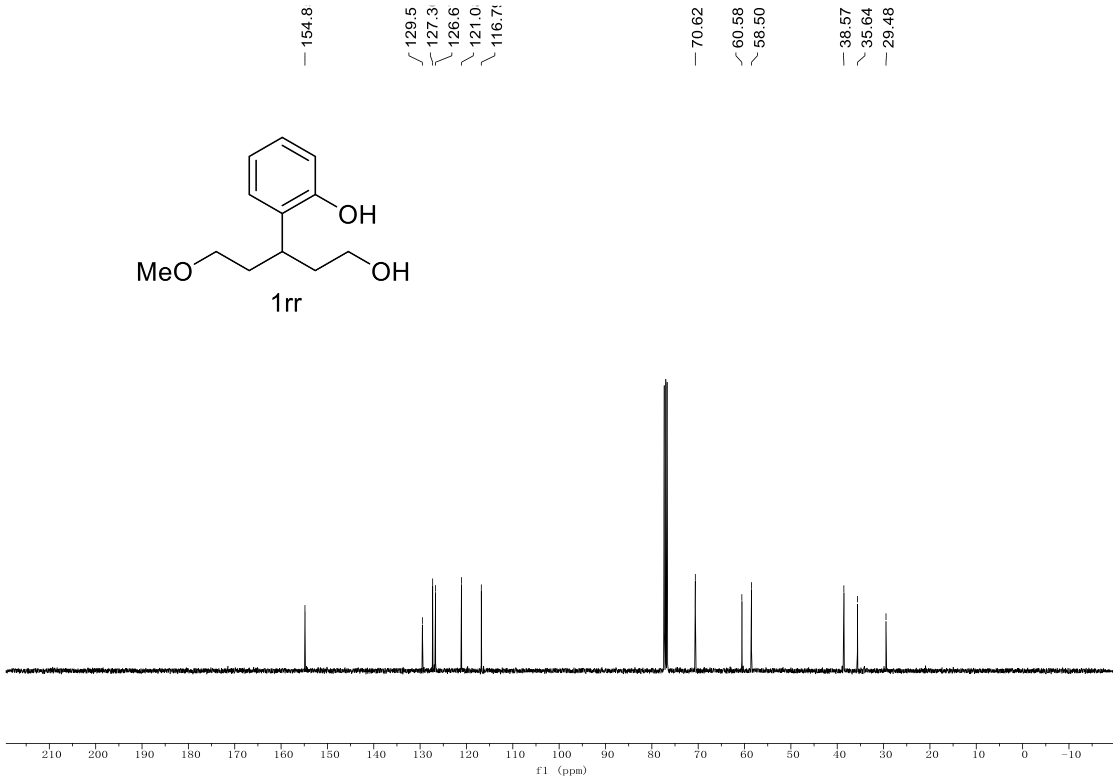

Supplementary Figure 120. ^{13}C NMR of compound **1rr** (101 MHz, $CDCl_3$)

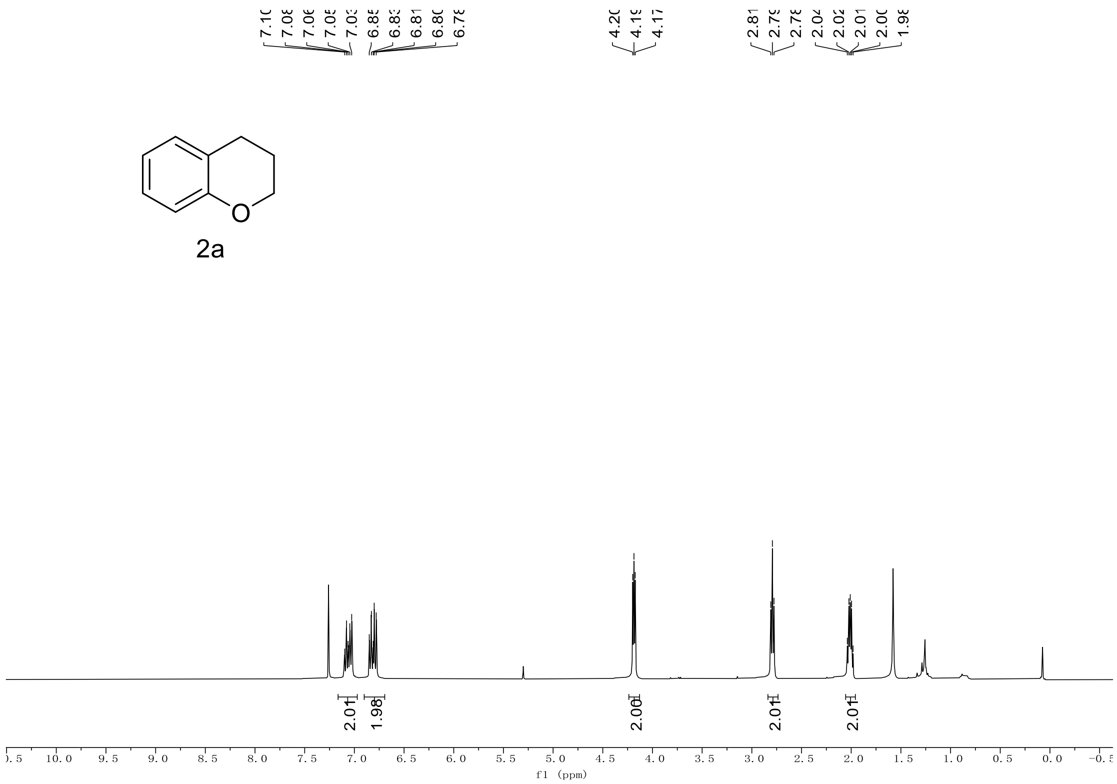

Supplementary Figure 121. ^{1}H NMR of compound **2a** (400 MHz, $CDCl_3$)

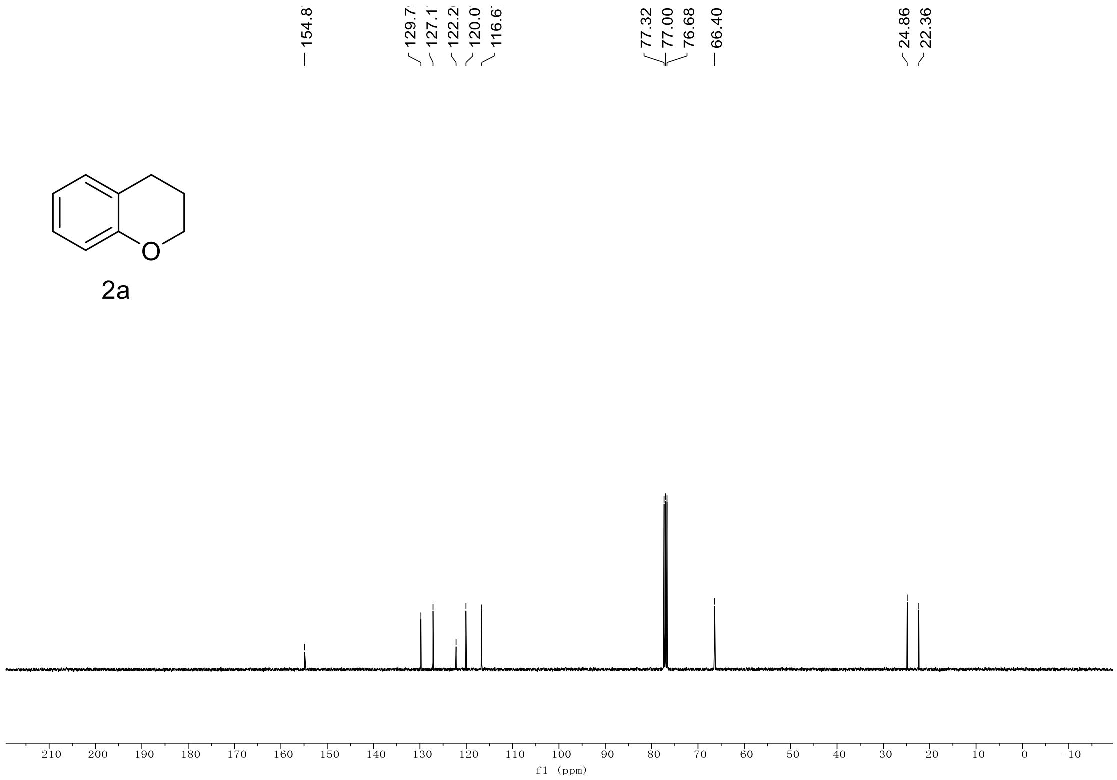

Supplementary Figure 122. ^{13}C NMR of compound **2a** (101 MHz, $CDCl_3$)

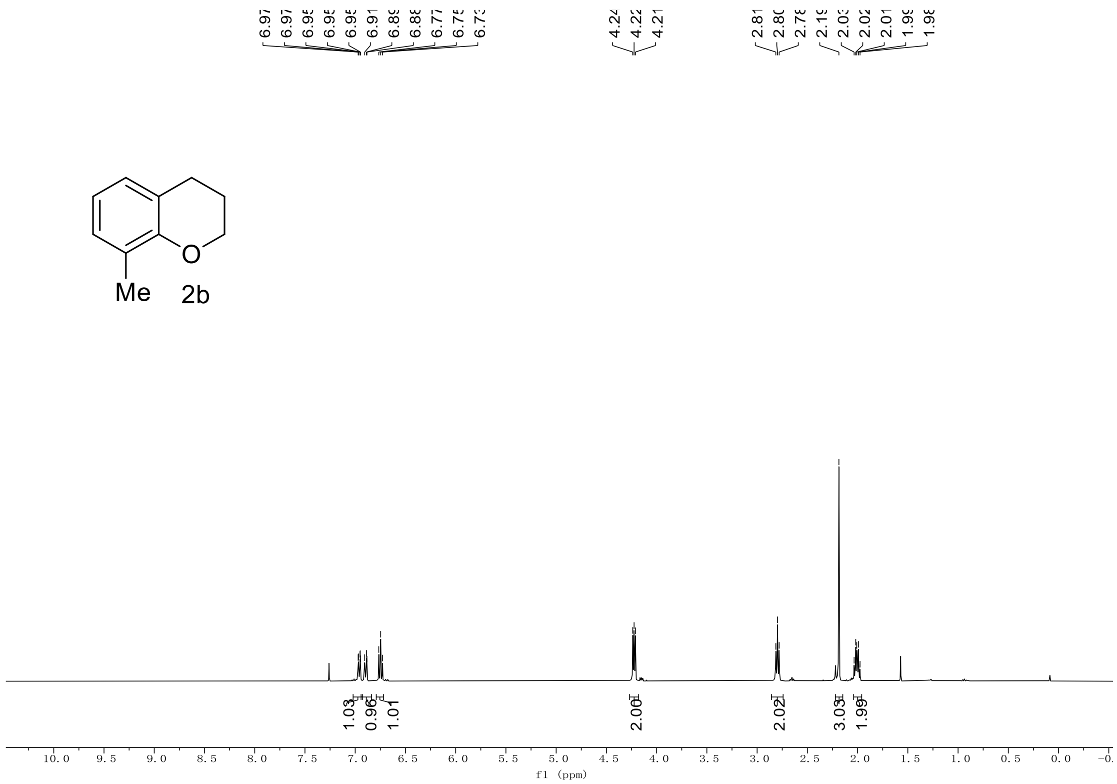

Supplementary Figure 123. ^{1}H NMR of compound **2b** (400 MHz, $CDCl_3$)

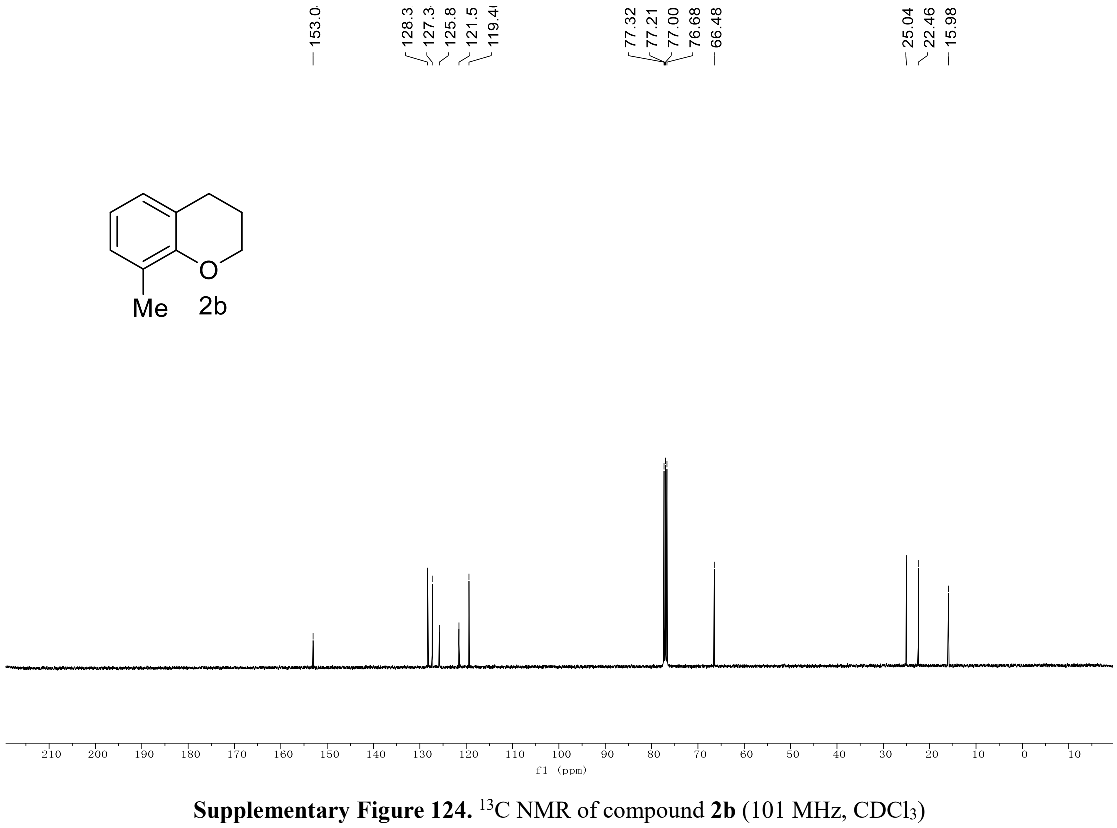

Supplementary Figure 124. ^{13}C NMR of compound **2b** (101 MHz, $CDCl_3$)

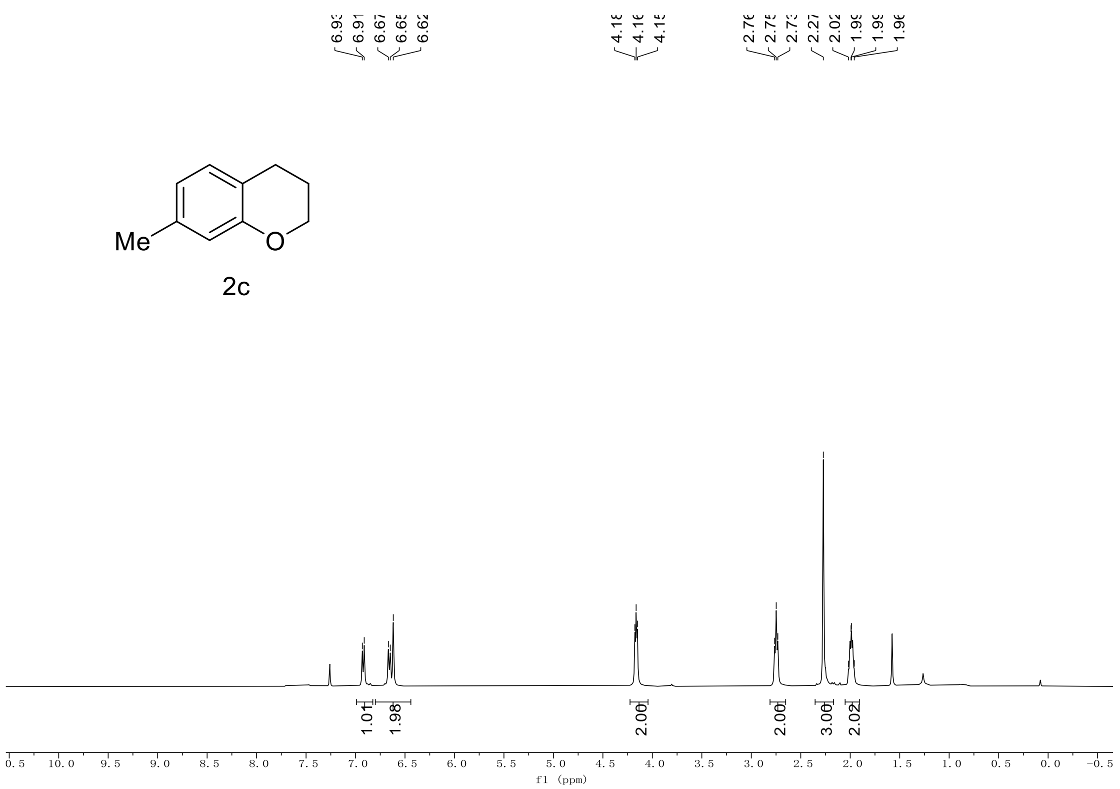

Supplementary Figure 125. ^{1}H NMR of compound **2c** (400 MHz, $CDCl_3$)

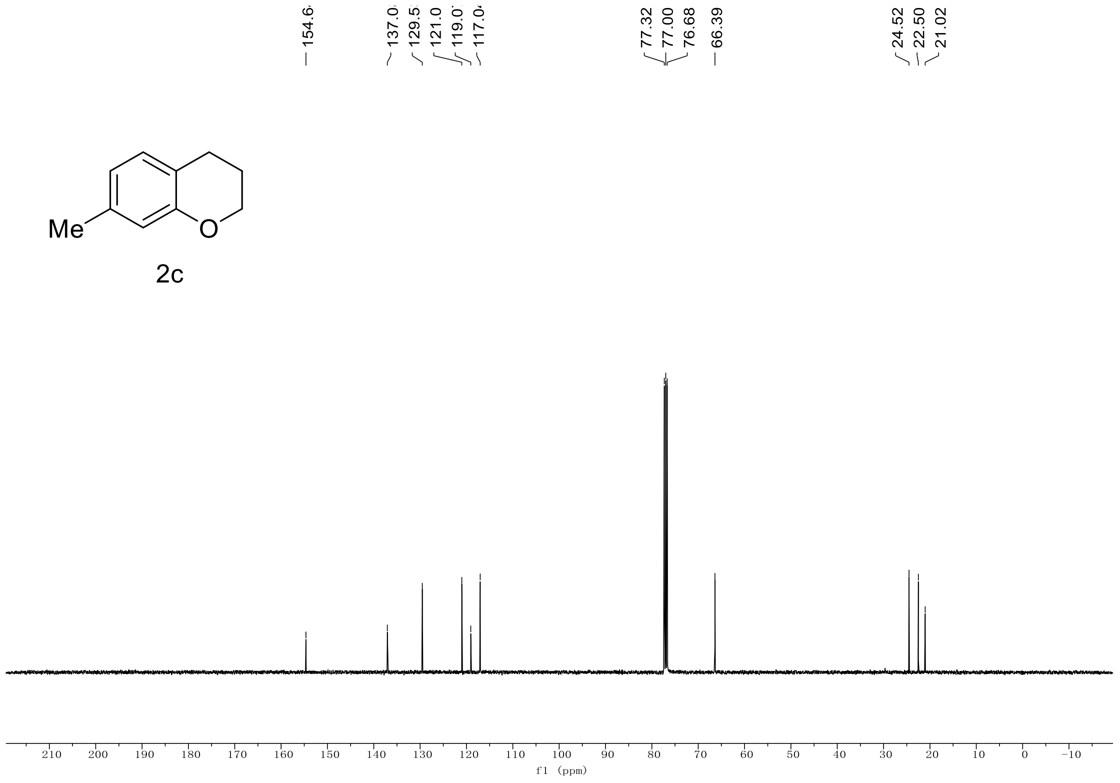

Supplementary Figure 126. ^{13}C NMR of compound **2c** (101 MHz, $CDCl_3$)

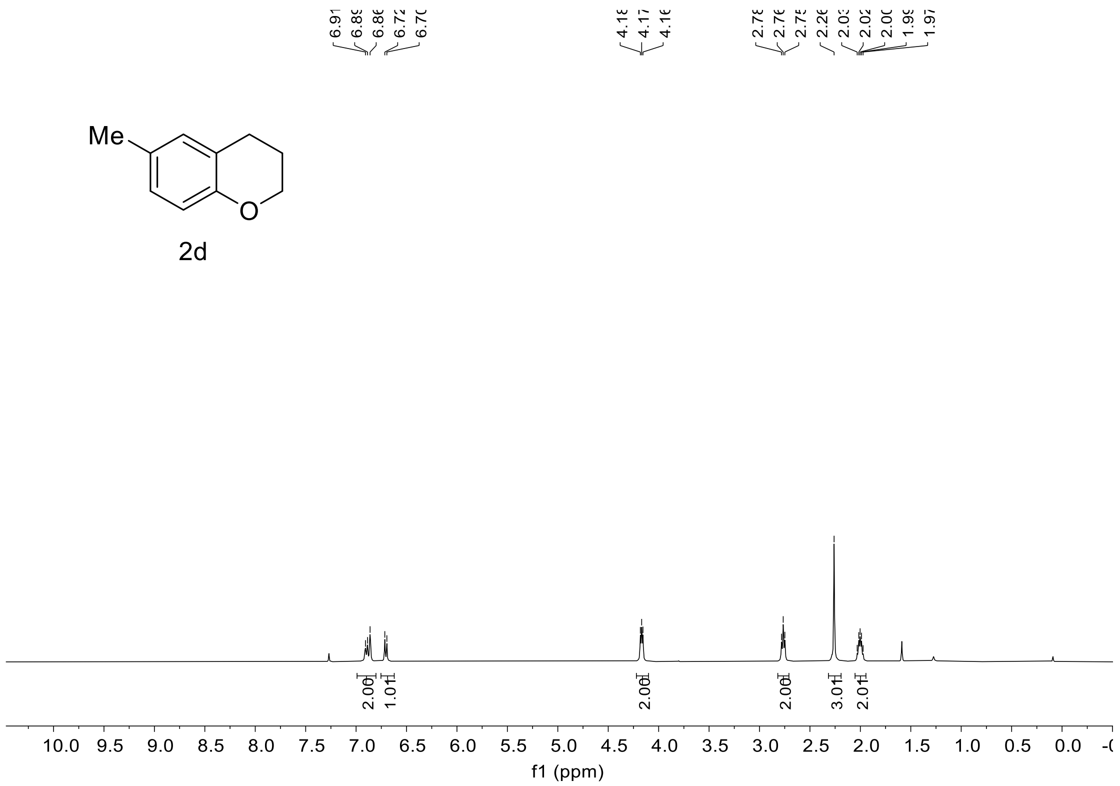

Supplementary Figure 127. ^{1}H NMR of compound **2d** (400 MHz, $CDCl_3$)

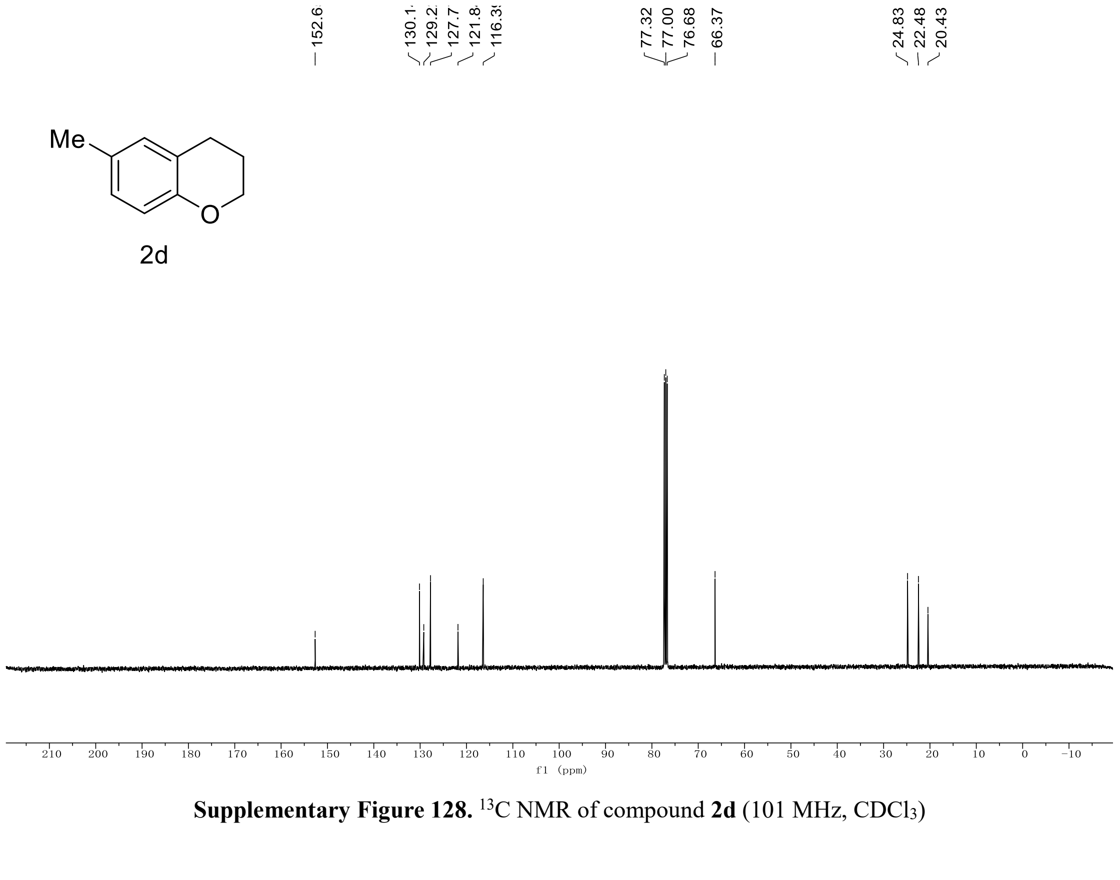

Supplementary Figure 128. ^{13}C NMR of compound **2d** (101 MHz, $CDCl_3$)

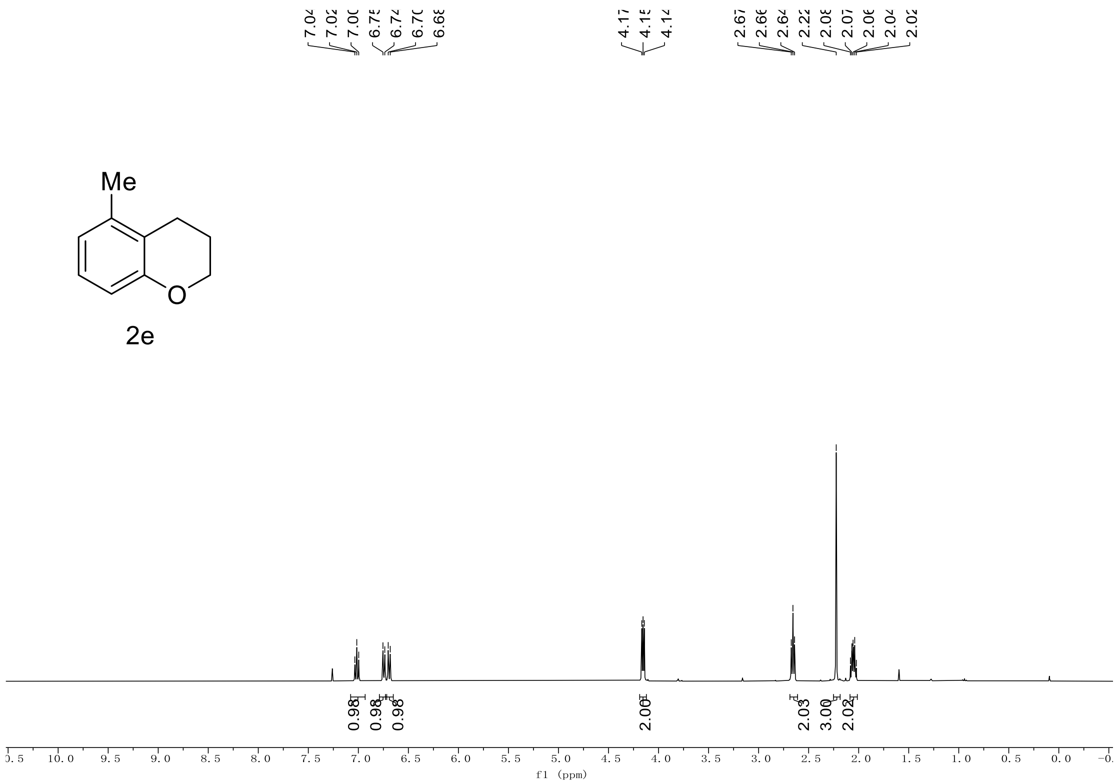

Supplementary Figure 129 ^{1}H NMR of compound **2e** (400 MHz, $CDCl_3$)

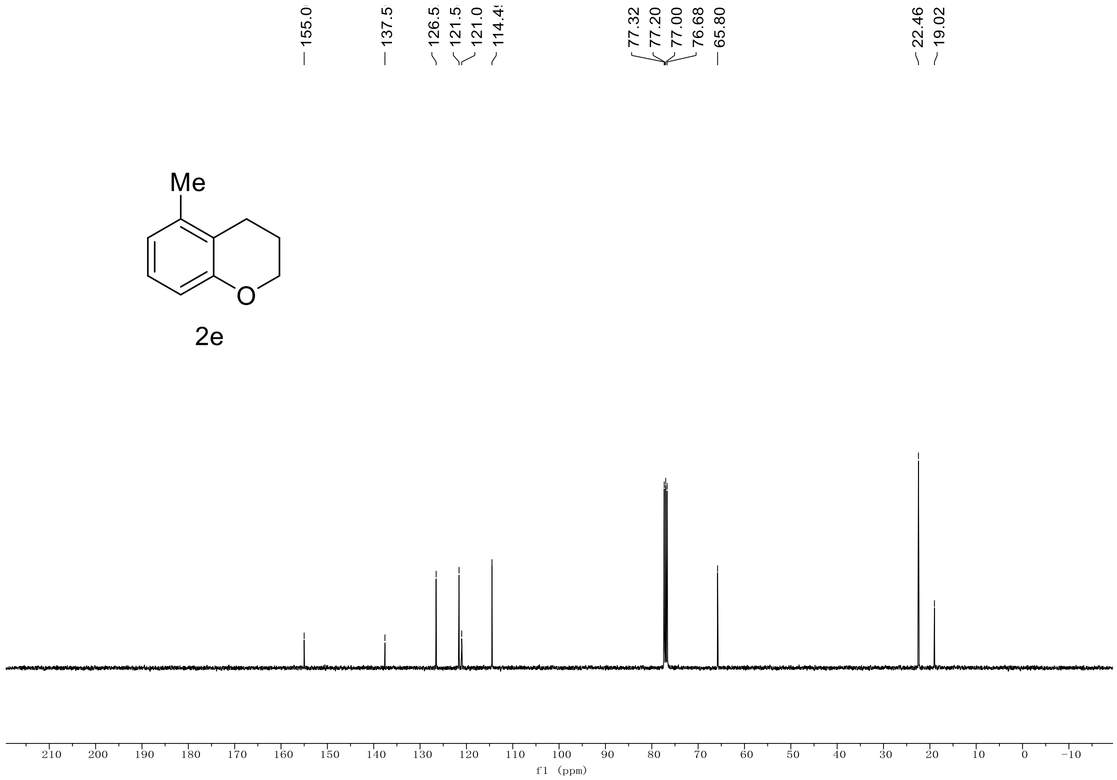

Supplementary Figure 130. ^{13}C NMR of compound **2e** (101 MHz, $CDCl_3$)

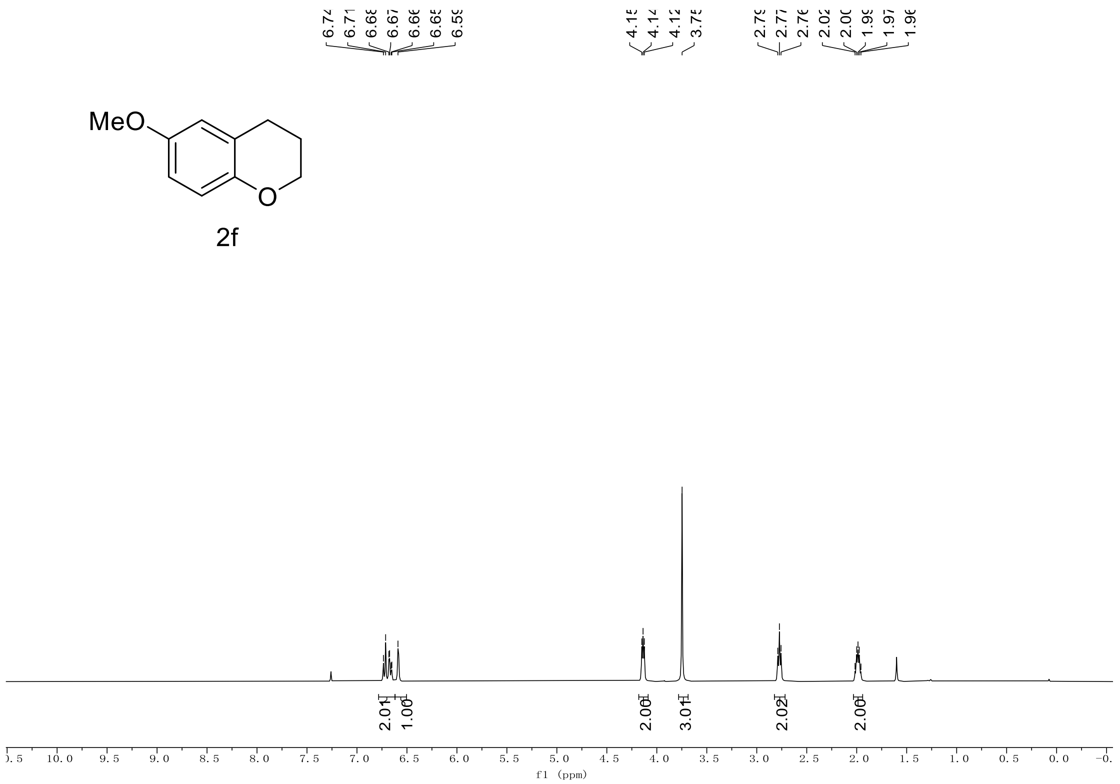

Supplementary Figure 131. ^{1}H NMR of compound **2f** (400 MHz, $CDCl_3$)

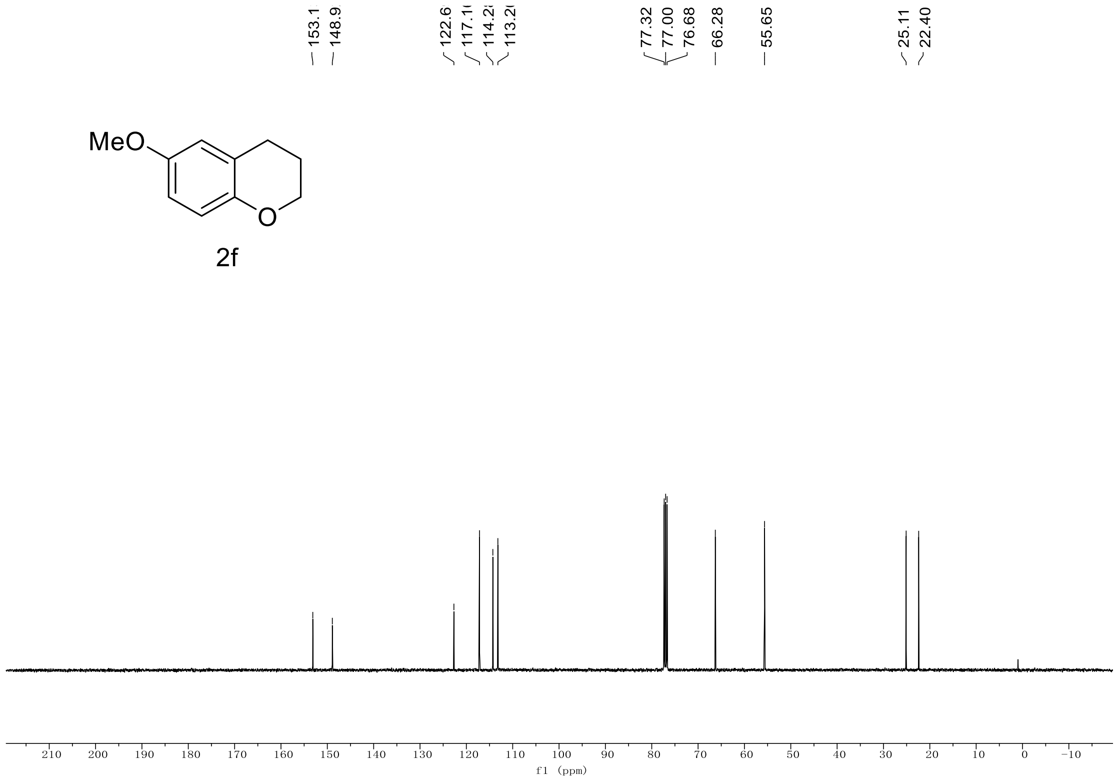

Supplementary Figure 132. ^{13}C NMR of compound **2f** (101 MHz, $CDCl_3$)

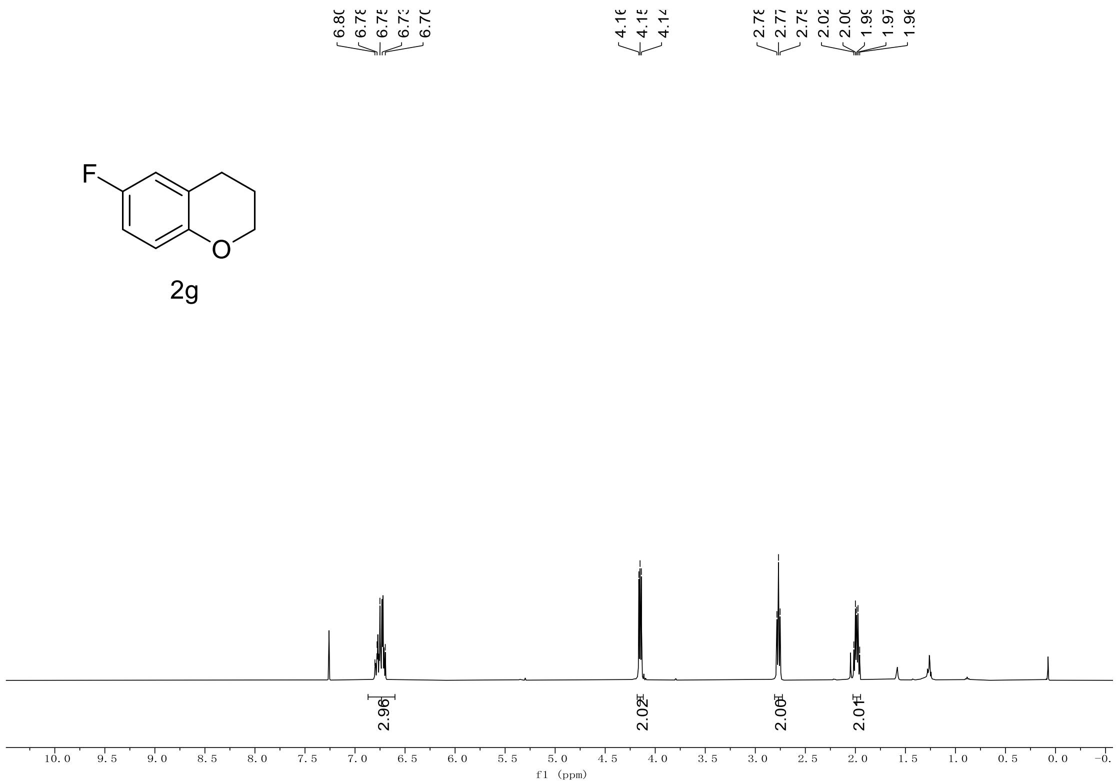

Supplementary Figure 133. ^{1}H NMR of compound **2g**(400 MHz, $CDCl_3$)

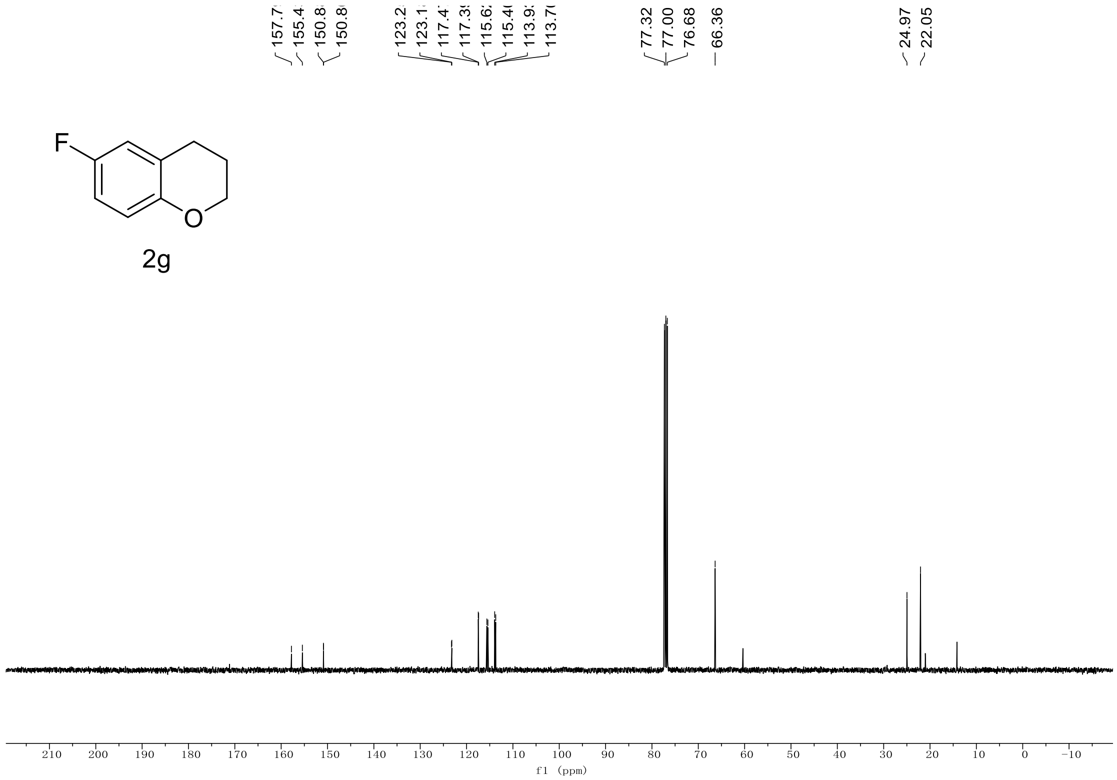

Supplementary Figure 134. ^{13}C NMR of compound **2g** (101 MHz, $CDCl_3$)

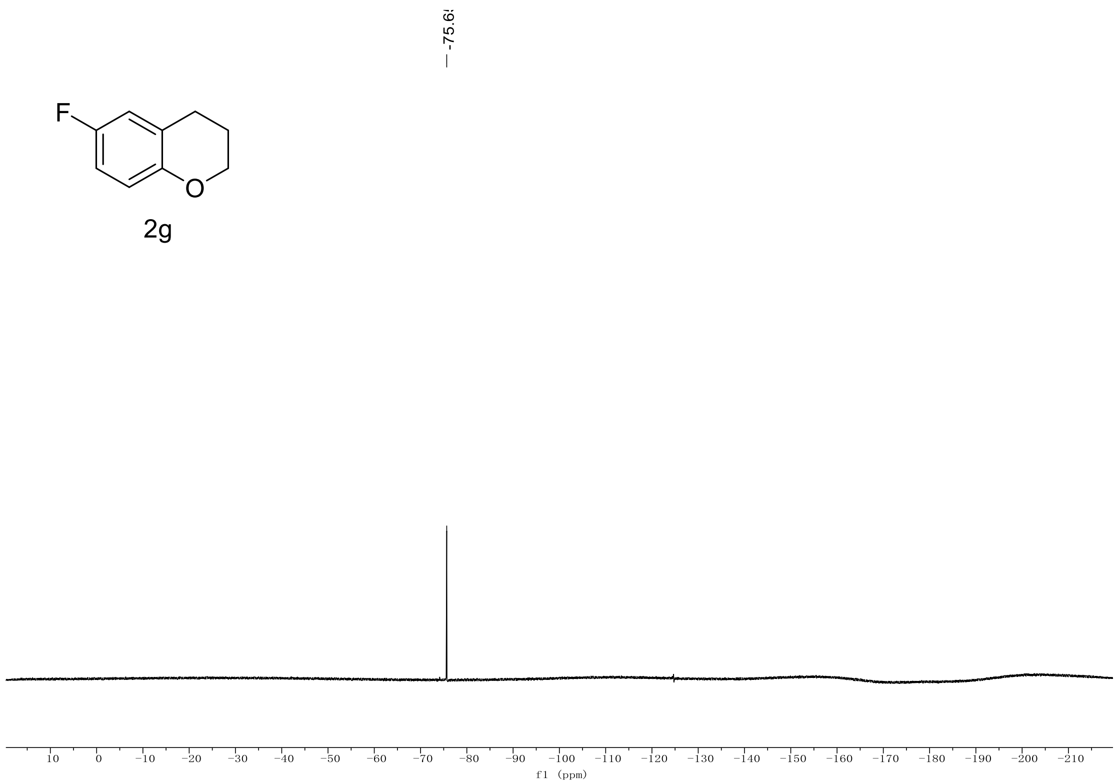

Supplementary Figure 135. ^{19}F NMR of compound **2g** (565 MHz, $CDCl_3$)

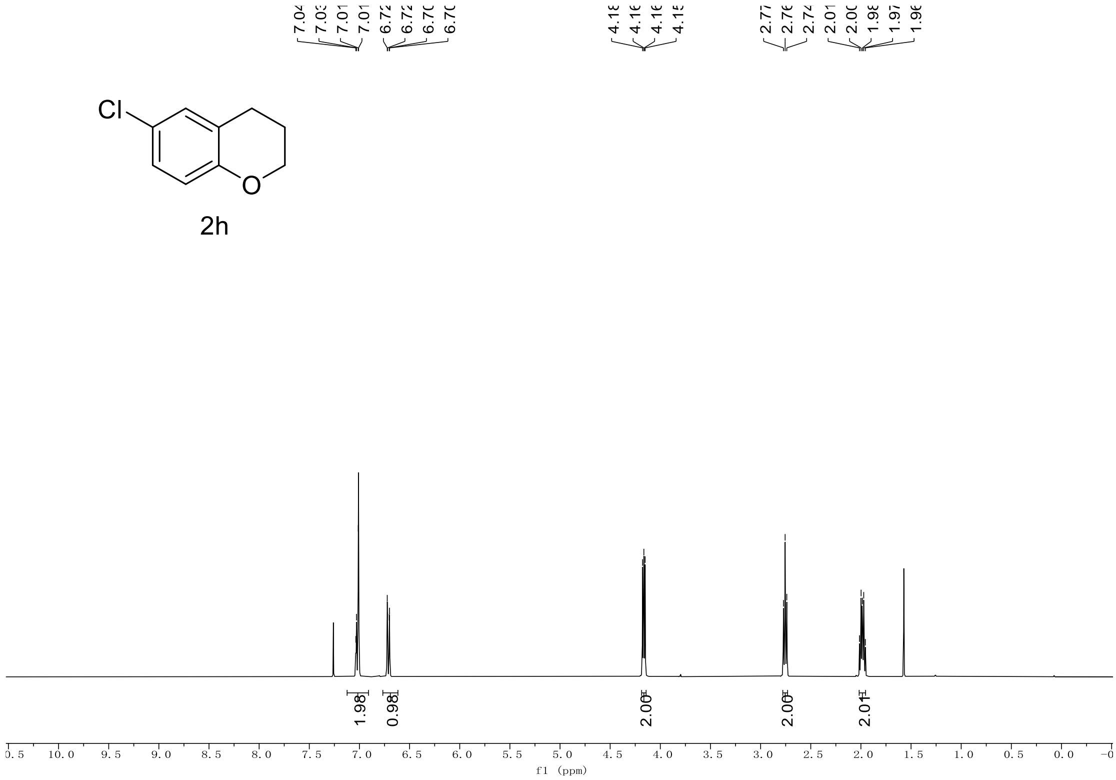

Supplementary Figure 136. ^{1}H NMR of compound **2h** (400 MHz, $CDCl_3$)

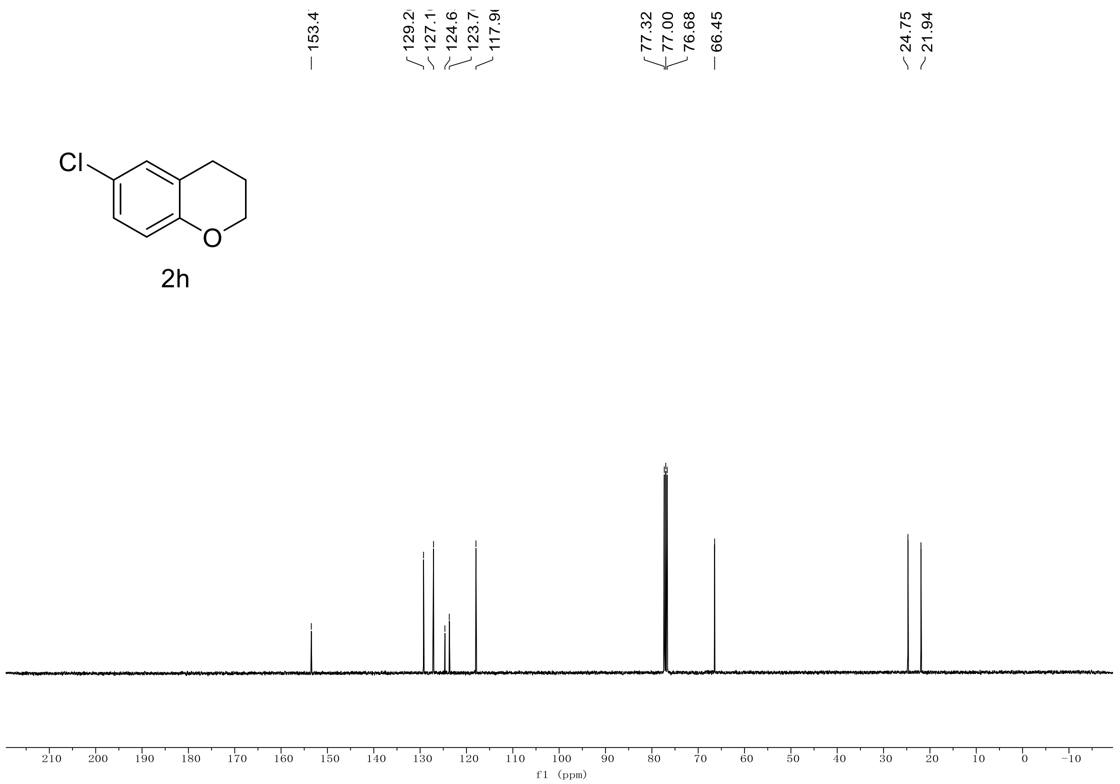

Supplementary Figure 137. ^{13}C NMR of compound **2h** (101 MHz, $CDCl_3$)

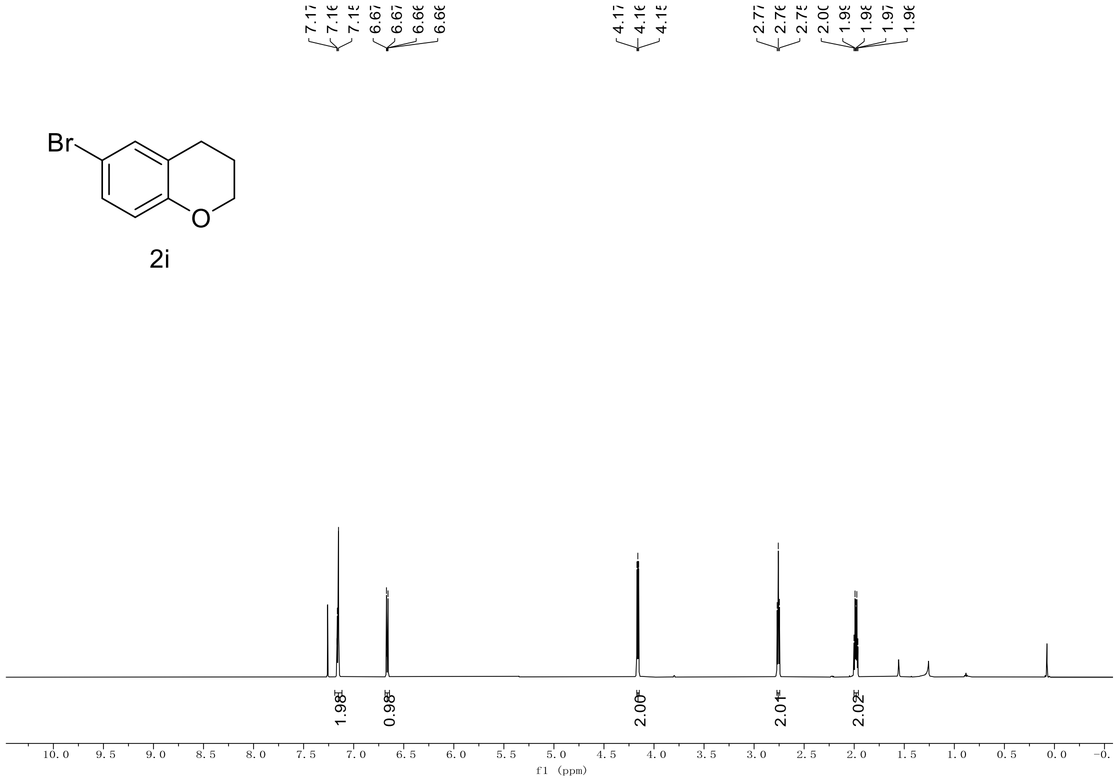

Supplementary Figure 138. ^{1}H NMR of compound **2i** (600 MHz, $CDCl_3$)

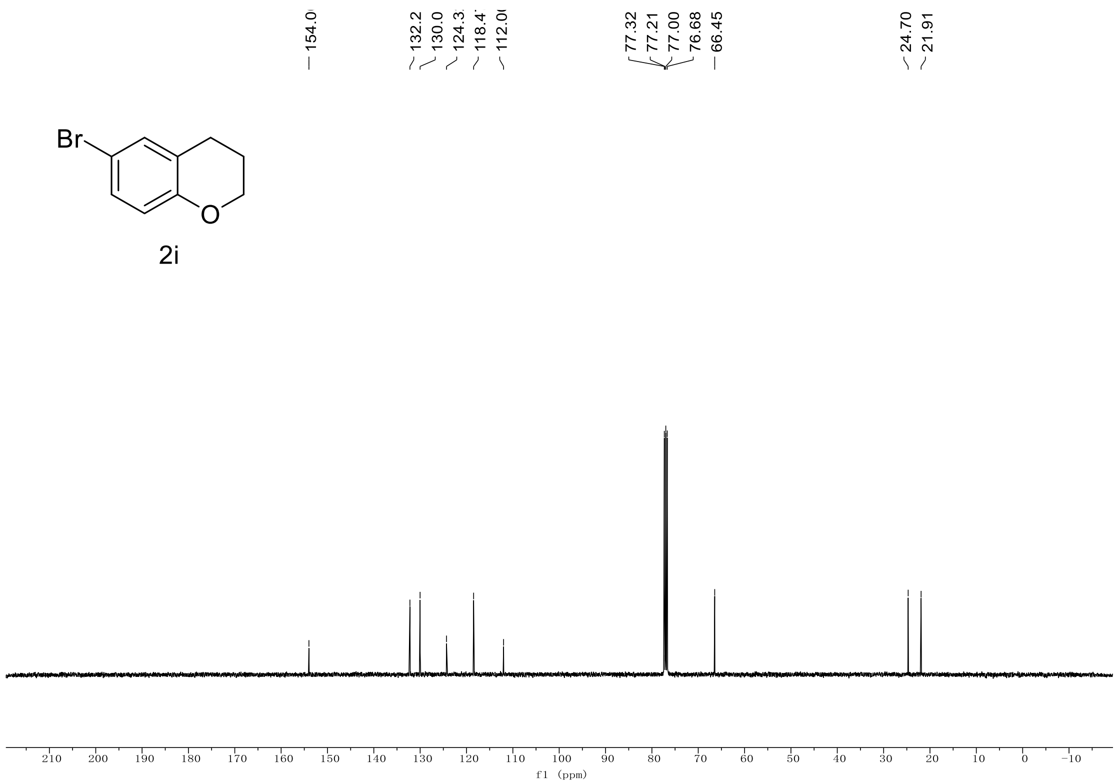

Supplementary Figure 139. ^{13}C NMR of compound **2i** (101 MHz, $CDCl_3$)

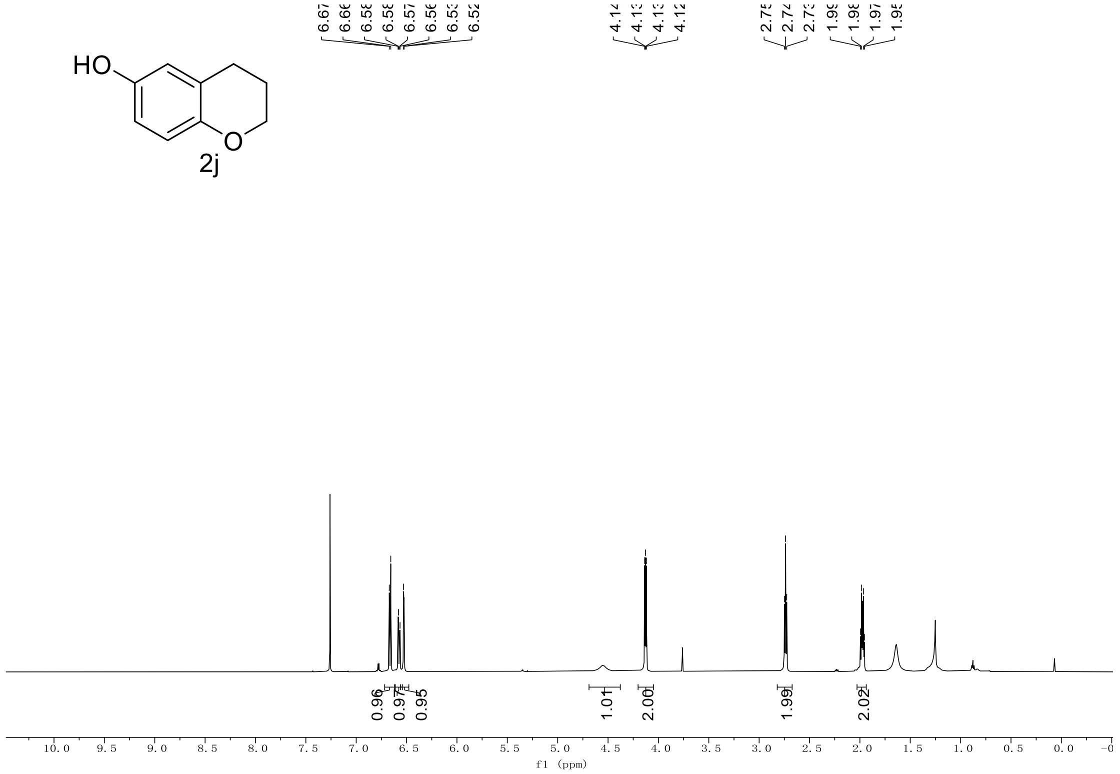

Supplementary Figure 140. ^{1}H NMR of compound **2j** (600 MHz, $CDCl_3$)

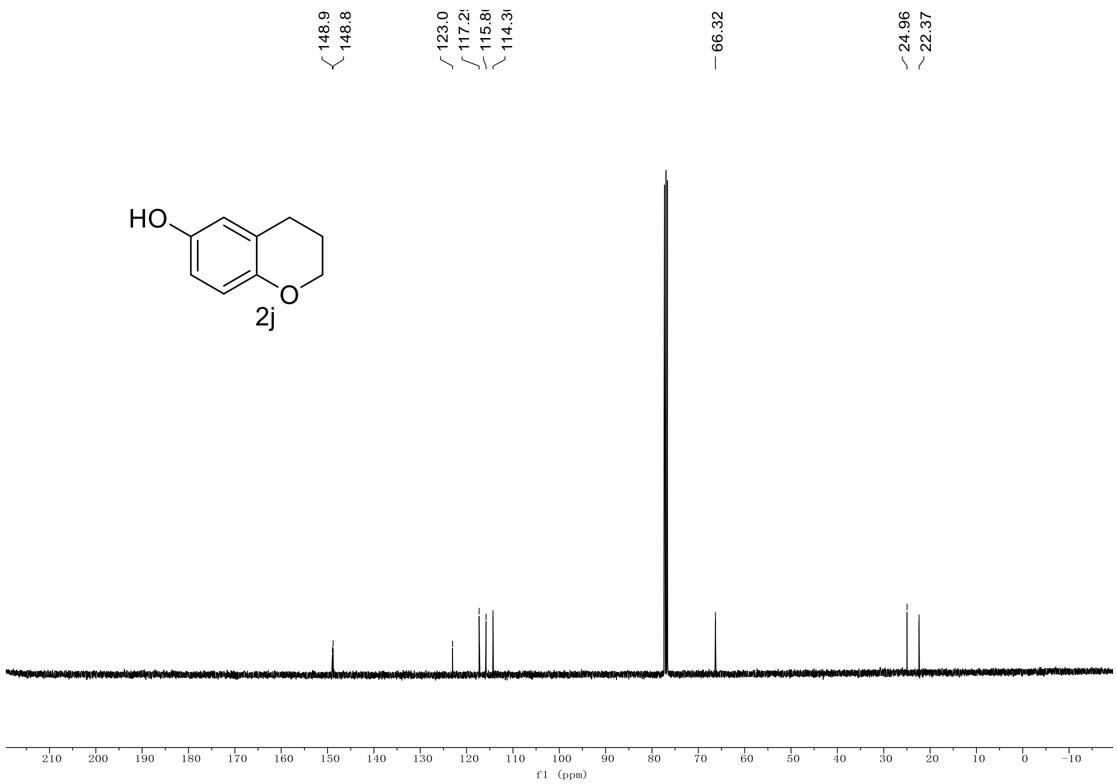

Supplementary Figure 141. ^{13}C NMR of compound **2j** (101 MHz, $CDCl_3$)

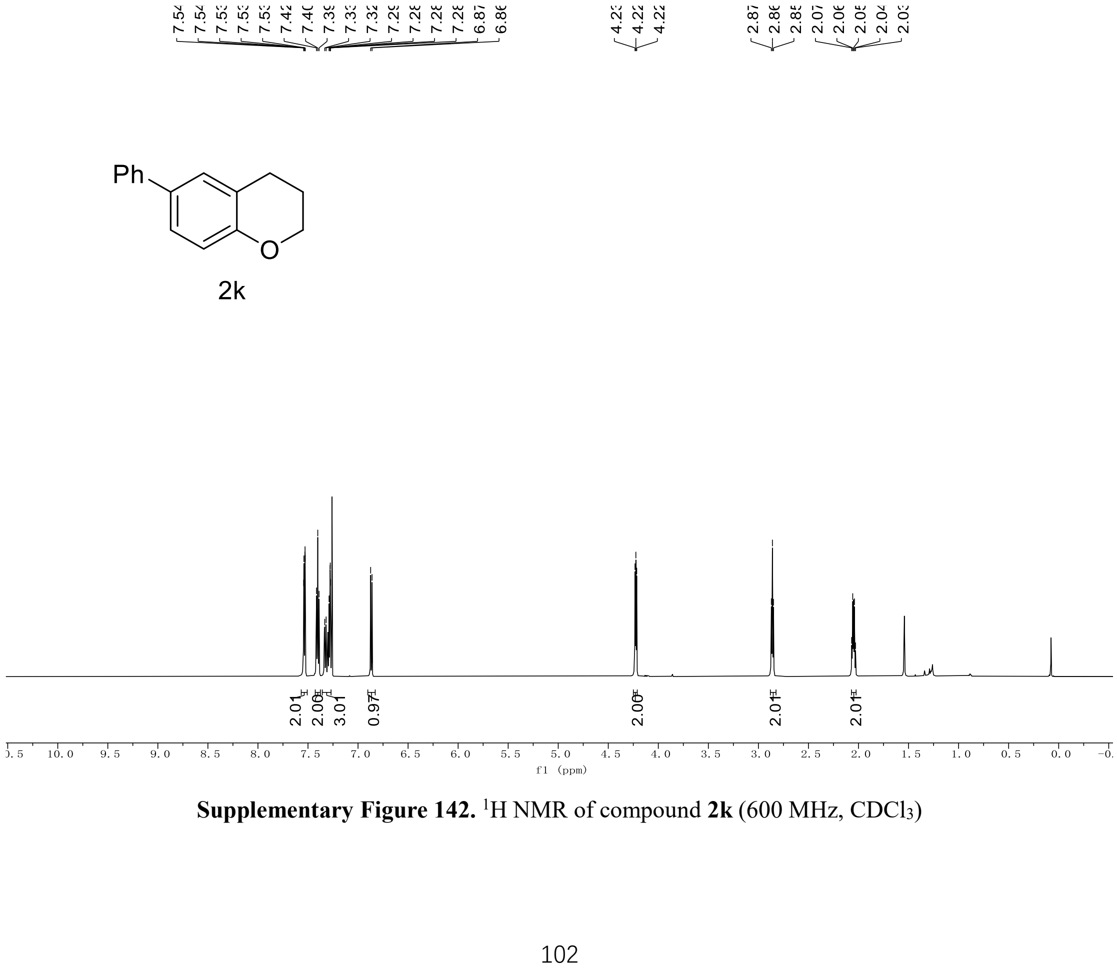

Supplementary Figure 142. ^{1}H NMR of compound **2k** (600 MHz, $CDCl_3$)

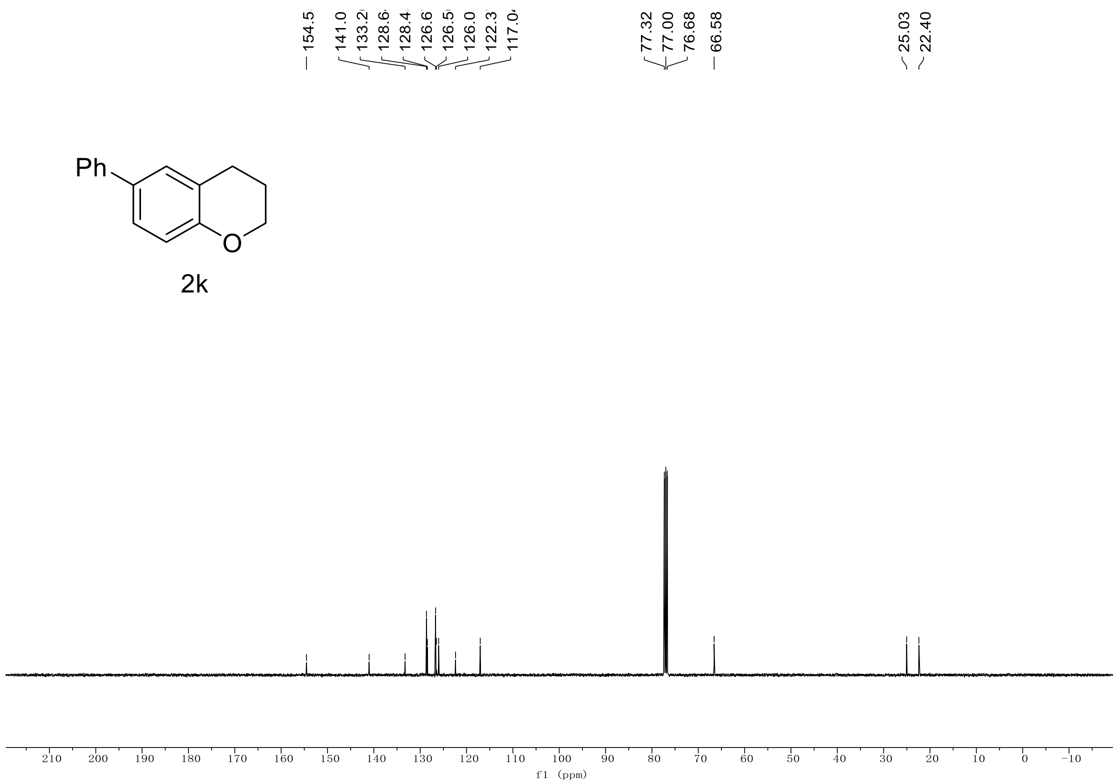

Supplementary Figure 143. ^{13}C NMR of compound **2k** (101 MHz, $CDCl_3$)

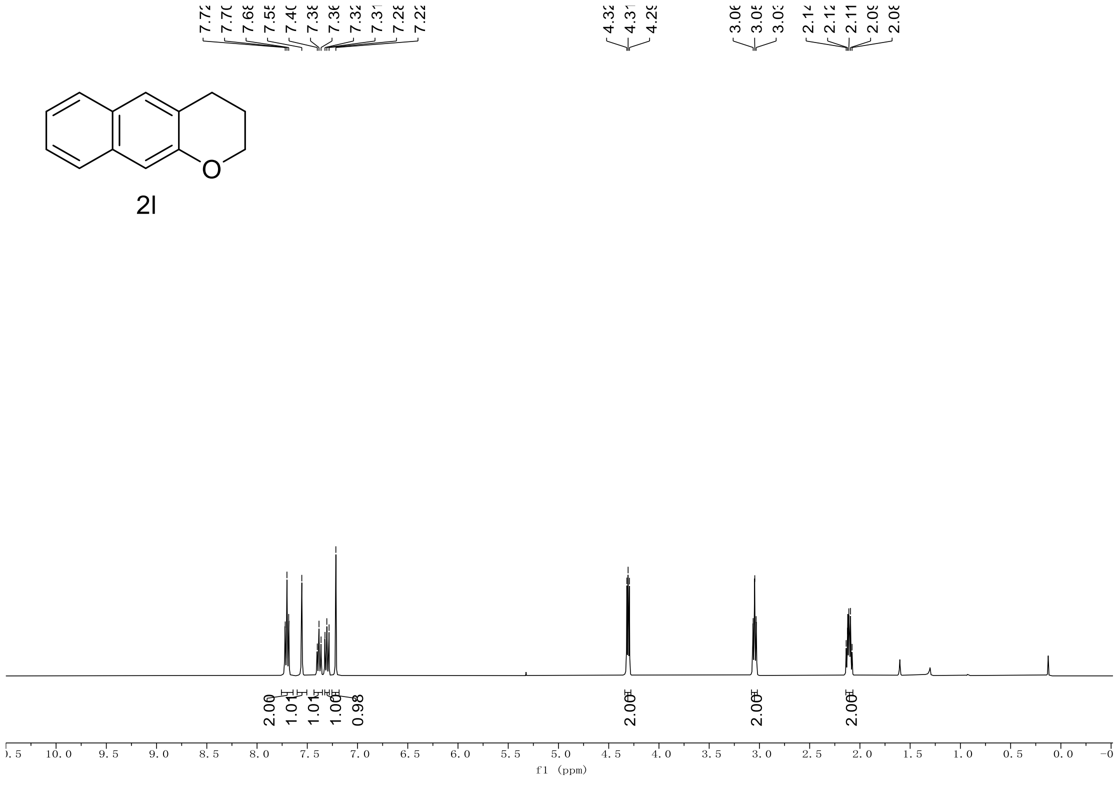

Supplementary Figure 144. ^{1}H NMR of compound **2l** (400 MHz, $CDCl_3$)

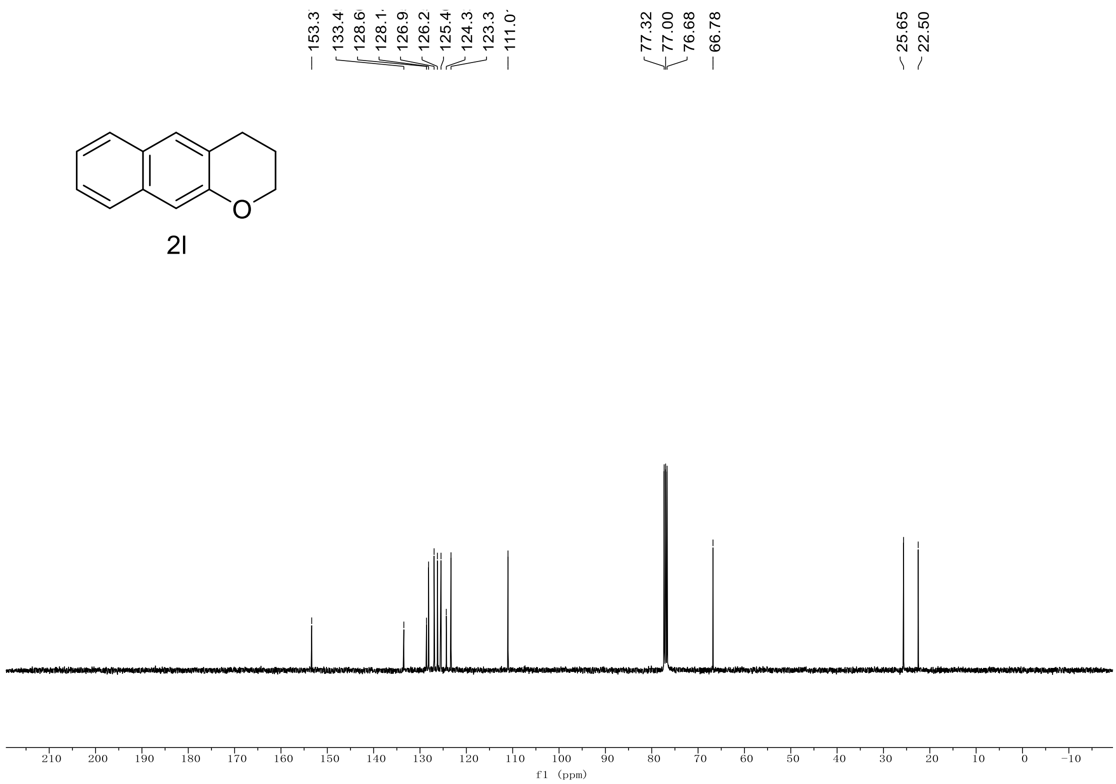

Supplementary Figure 145. ^{13}C NMR of compound **2l** (101 MHz, $CDCl_3$)

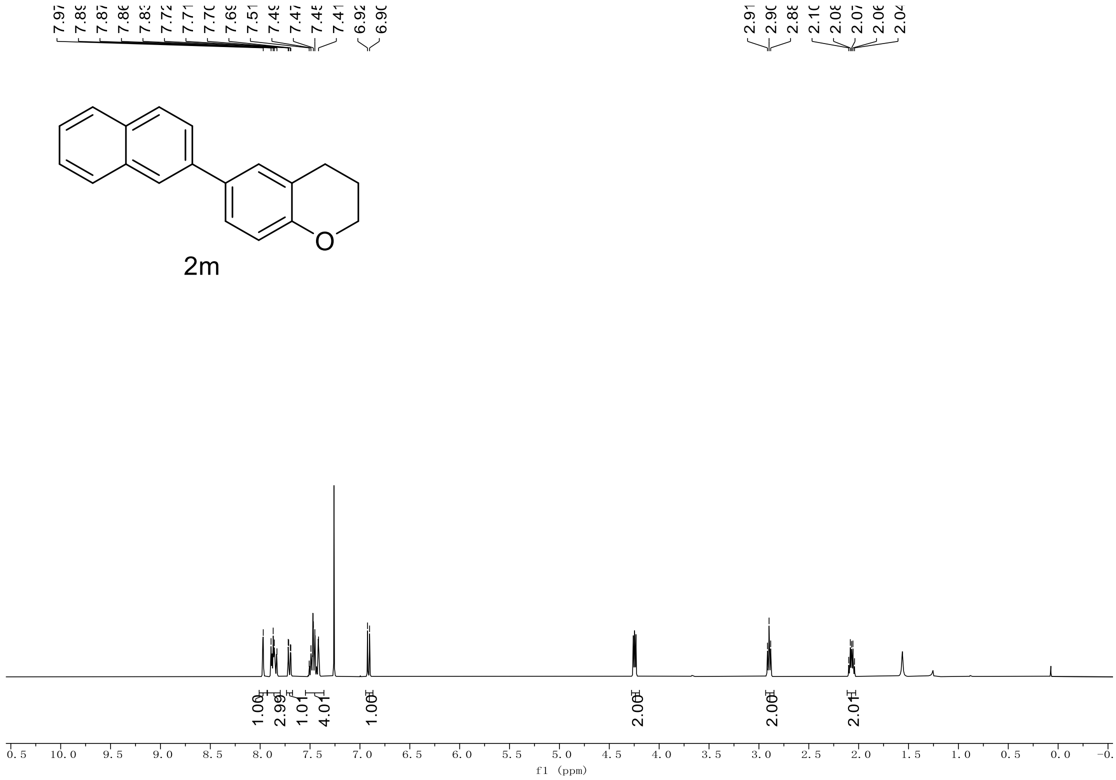

Supplementary Figure 146. ^{1}H NMR of compound **2m** (400 MHz, $CDCl_3$)

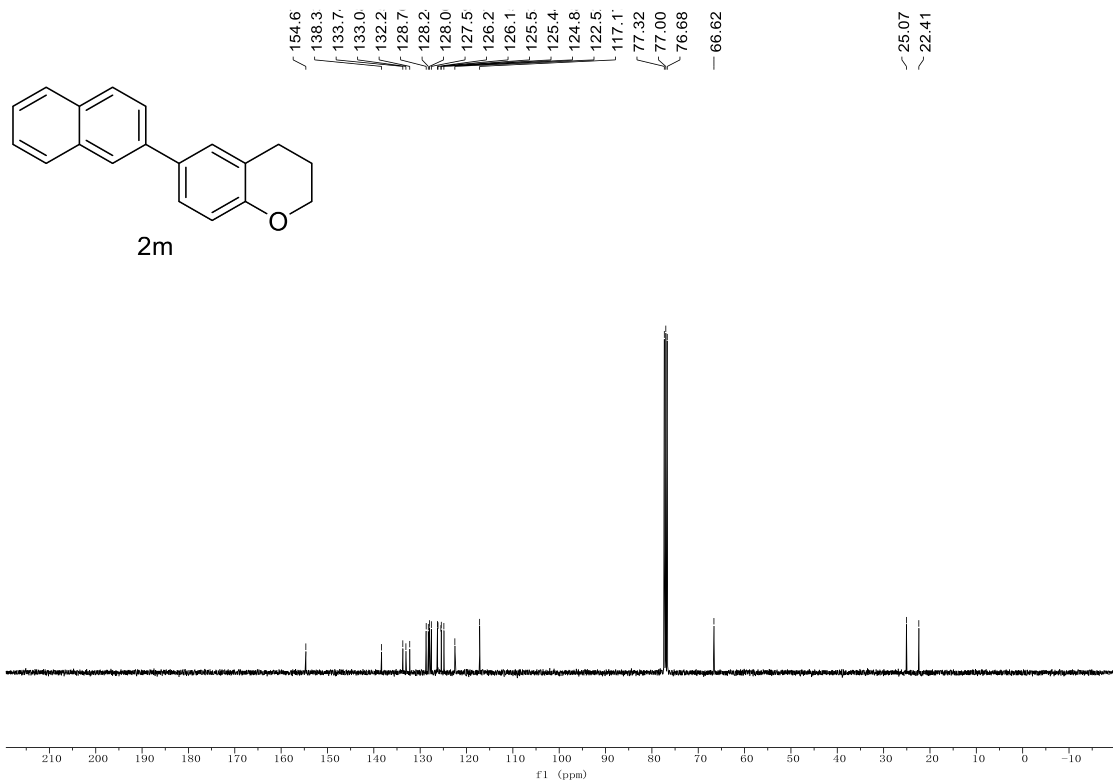

Supplementary Figure 147. ^{13}C NMR of compound **2m** (101 MHz, $CDCl_3$)

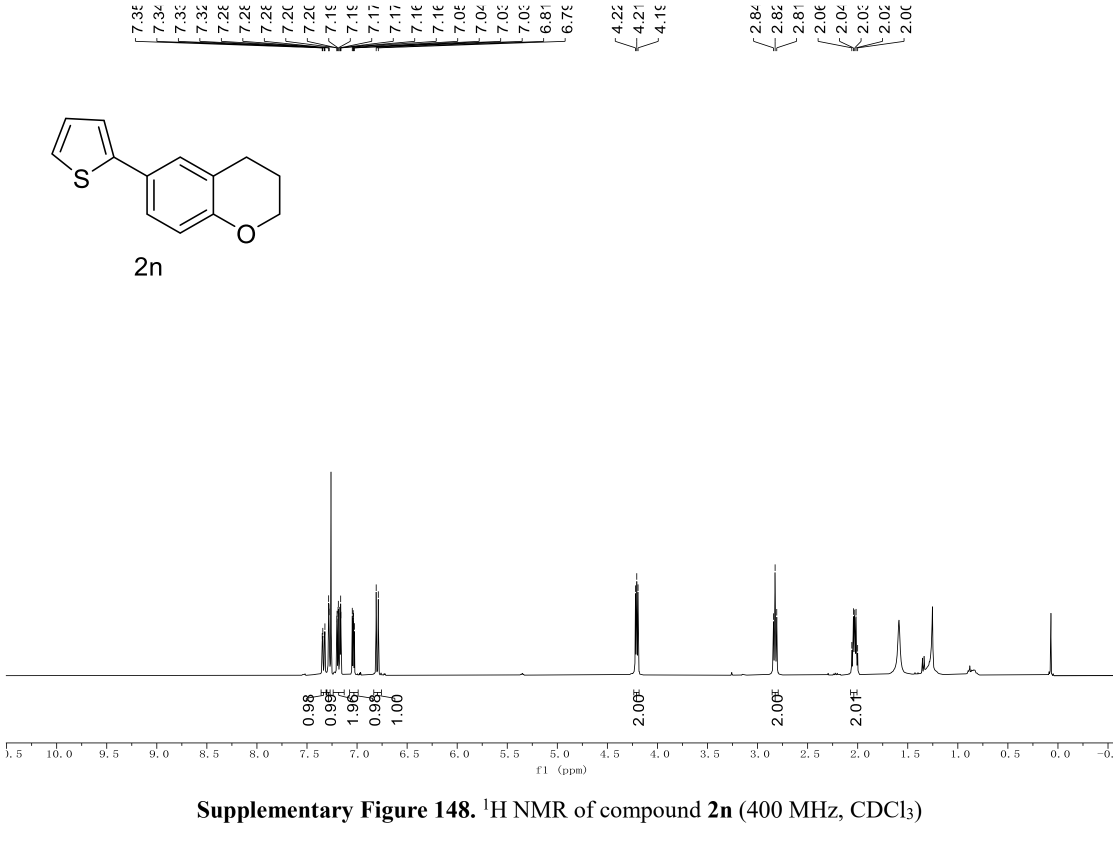

Supplementary Figure 148. ^{1}H NMR of compound **2n** (400 MHz, $CDCl_3$)

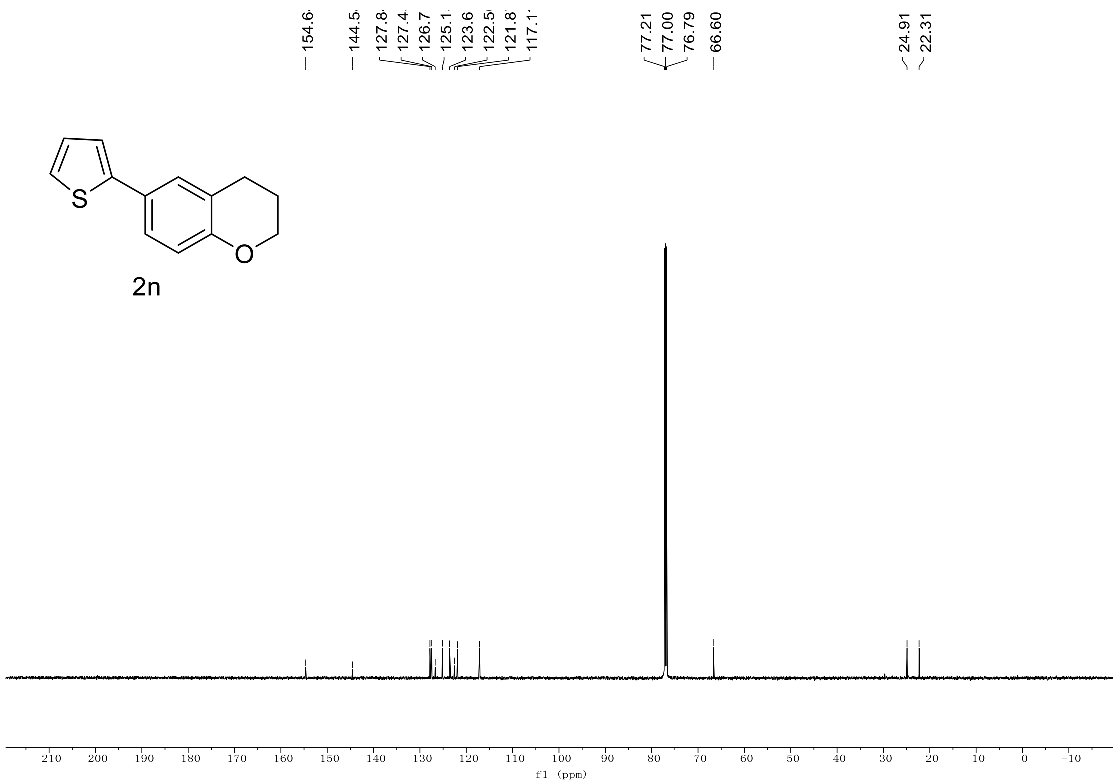

Supplementary Figure 149. ^{13}C NMR of compound **2n** (151 MHz, $CDCl_3$)

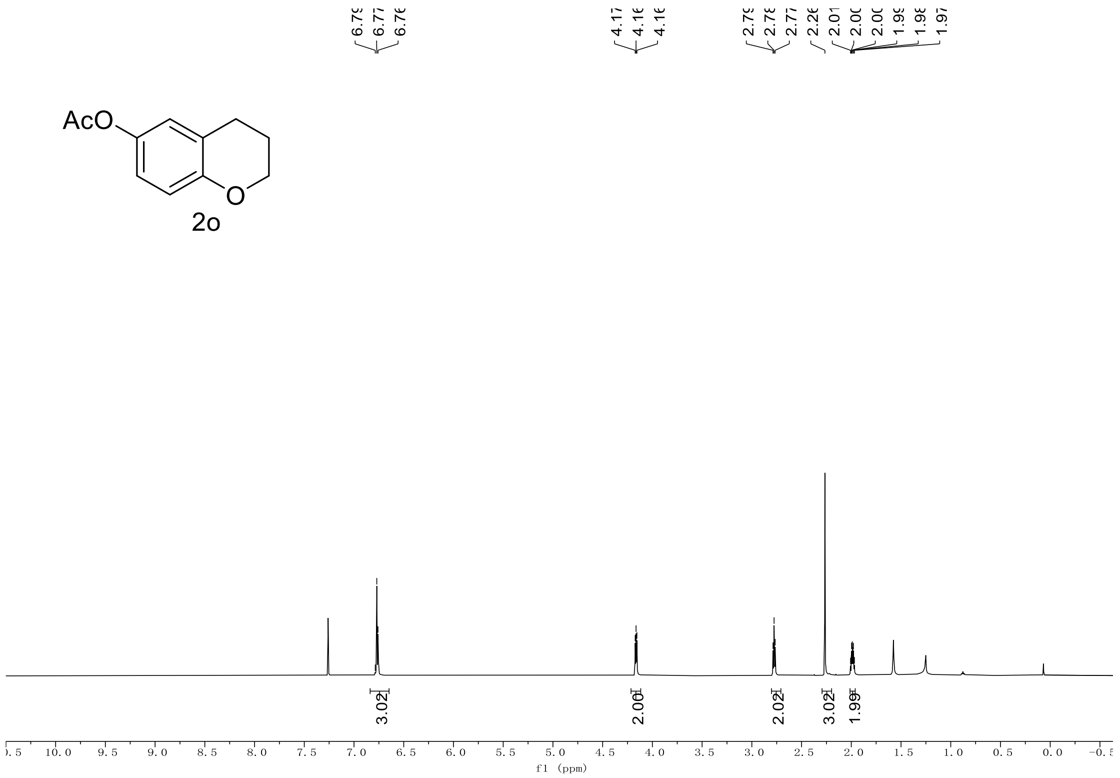

Supplementary Figure 150. ^{1}H NMR of compound **2o** (600 MHz, $CDCl_3$)

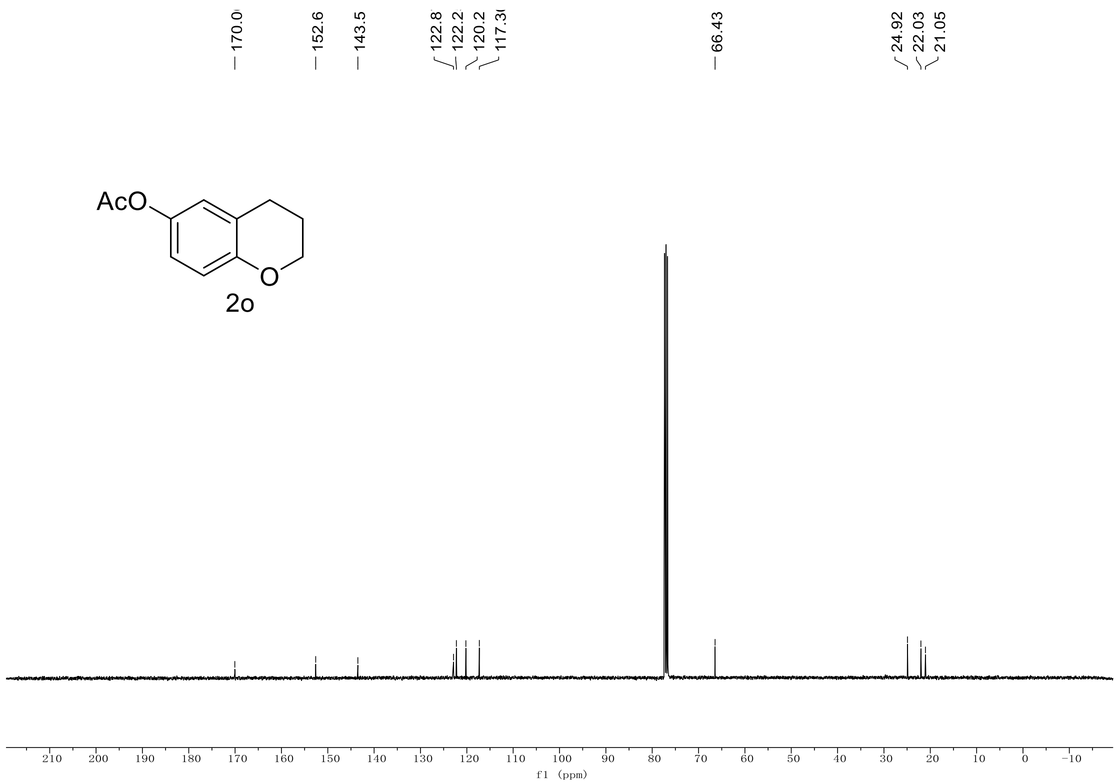

Supplementary Figure 151. ^{13}C NMR of compound **2o** (101 MHz, $CDCl_3$)

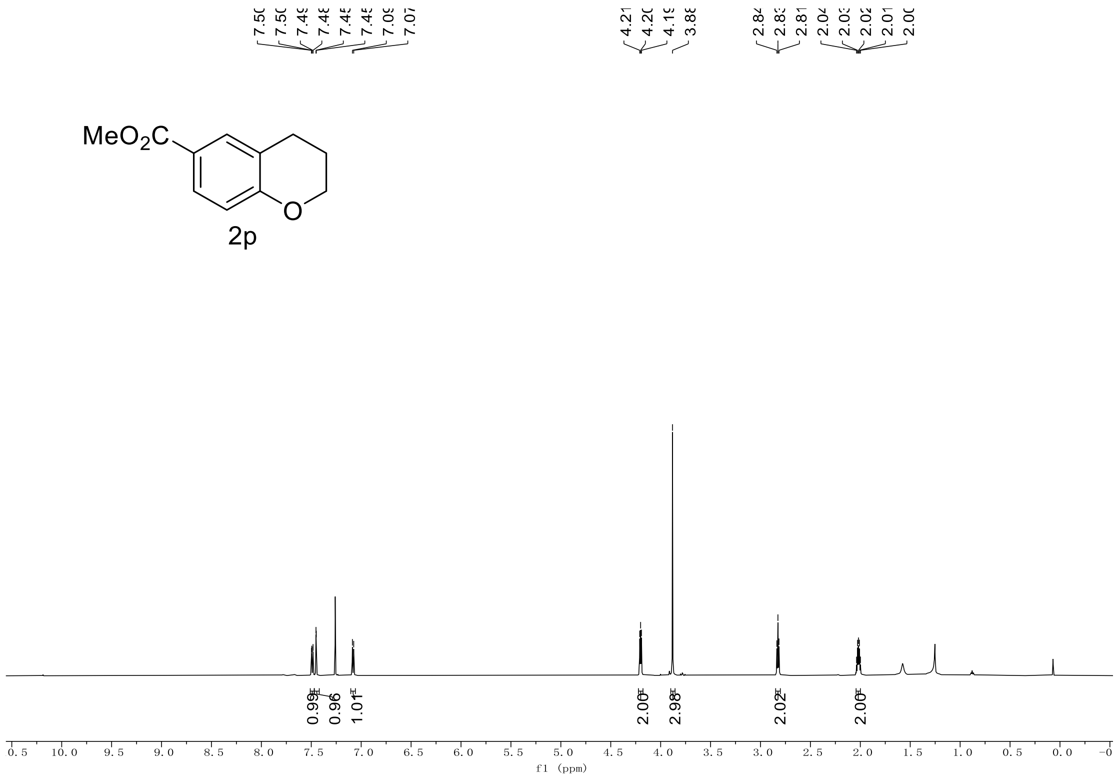

Supplementary Figure 152. ^{1}H NMR of compound **2p** (600 MHz, $CDCl_3$)

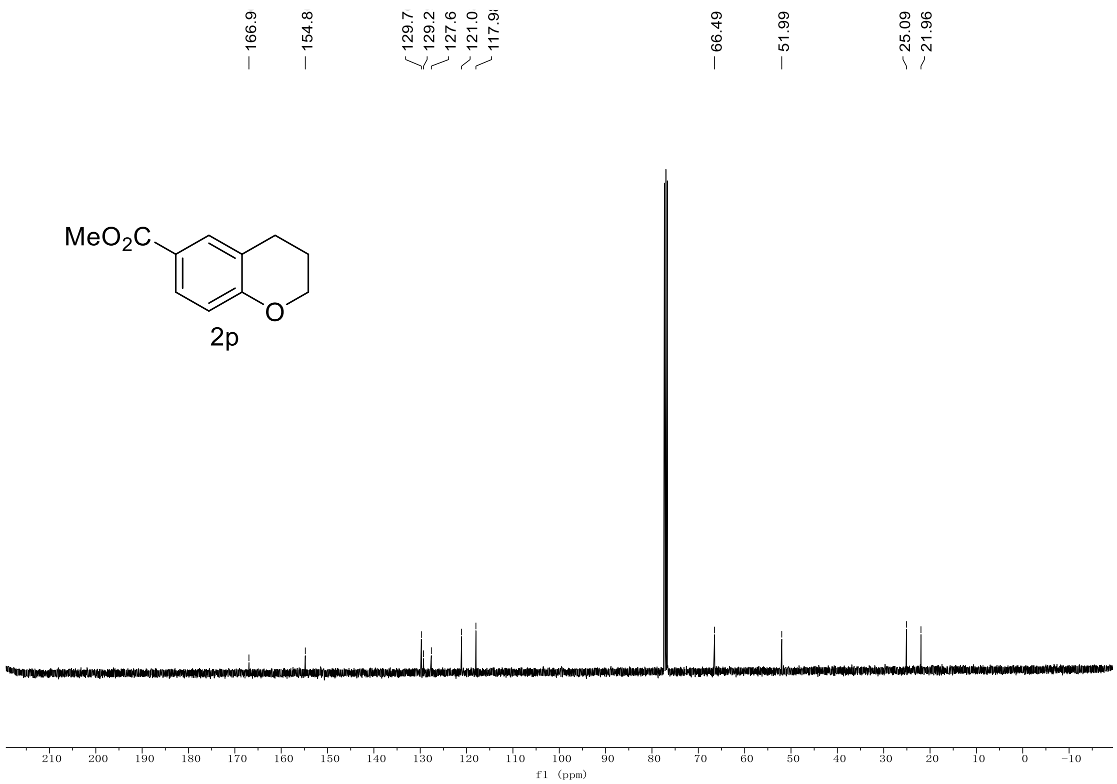

Supplementary Figure 153. ^{13}C NMR of compound **2p** (101 MHz, $CDCl_3$)

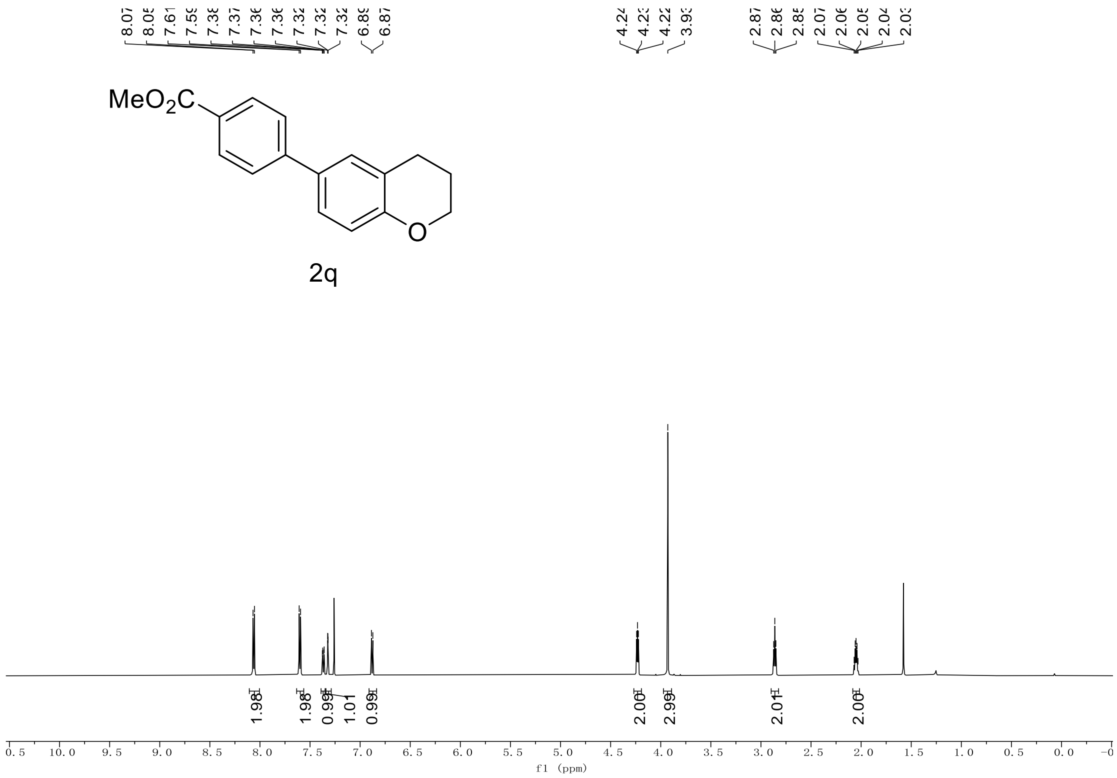

Supplementary Figure 154. ^{1}H NMR of compound **2q** (600 MHz, $CDCl_3$)

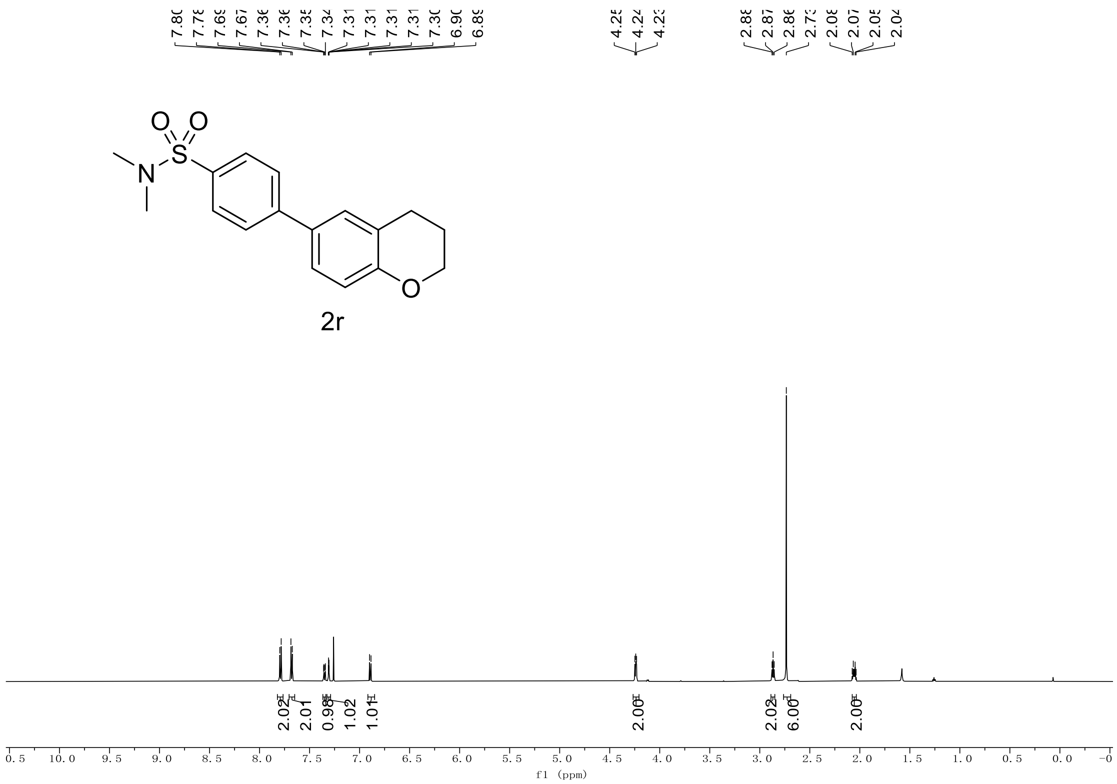

Supplementary Figure 155. ^{1}H NMR of compound **2r** (600 MHz, $CDCl_3$)

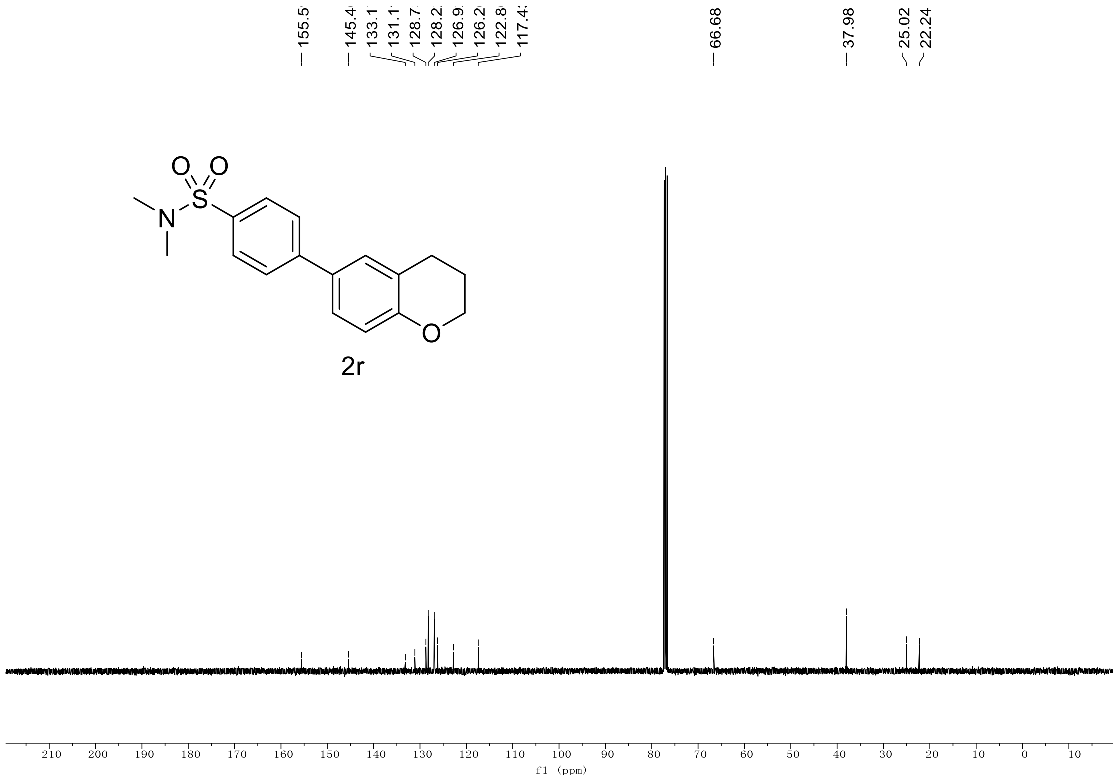

Supplementary Figure 156. ^{13}C NMR of compound **2r** (101 MHz, $CDCl_3$)

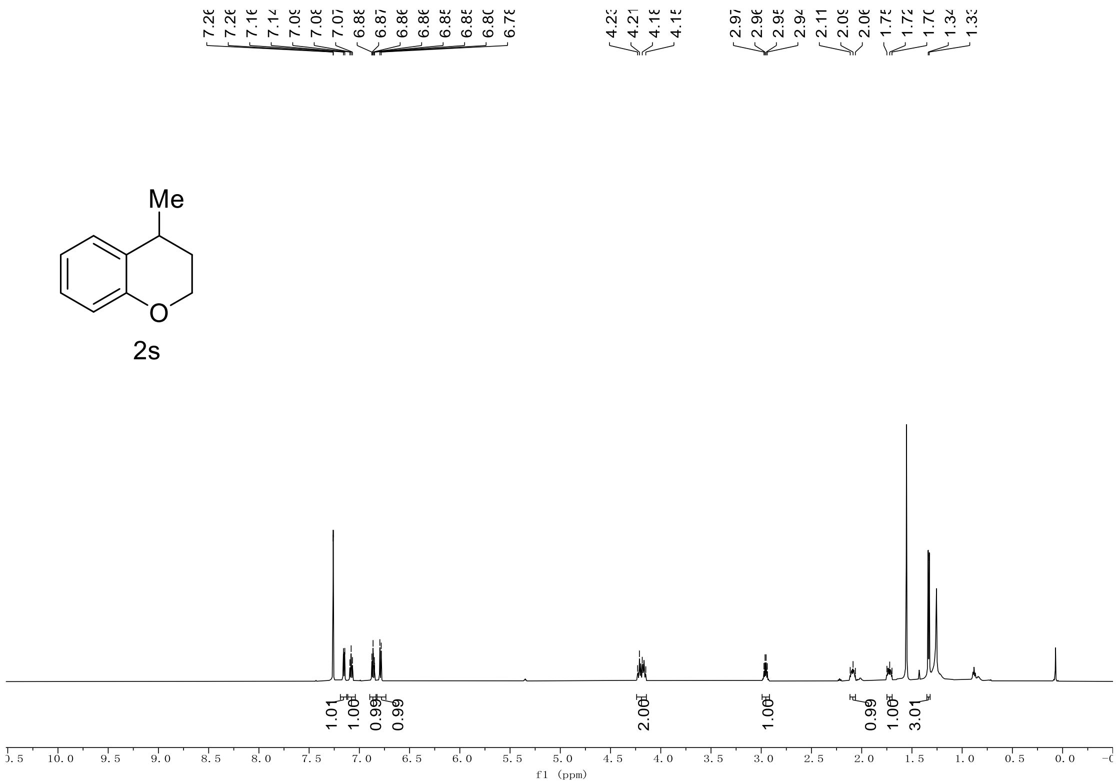

Supplementary Figure 157. ^{1}H NMR of compound **2s** (600 MHz, $CDCl_3$)

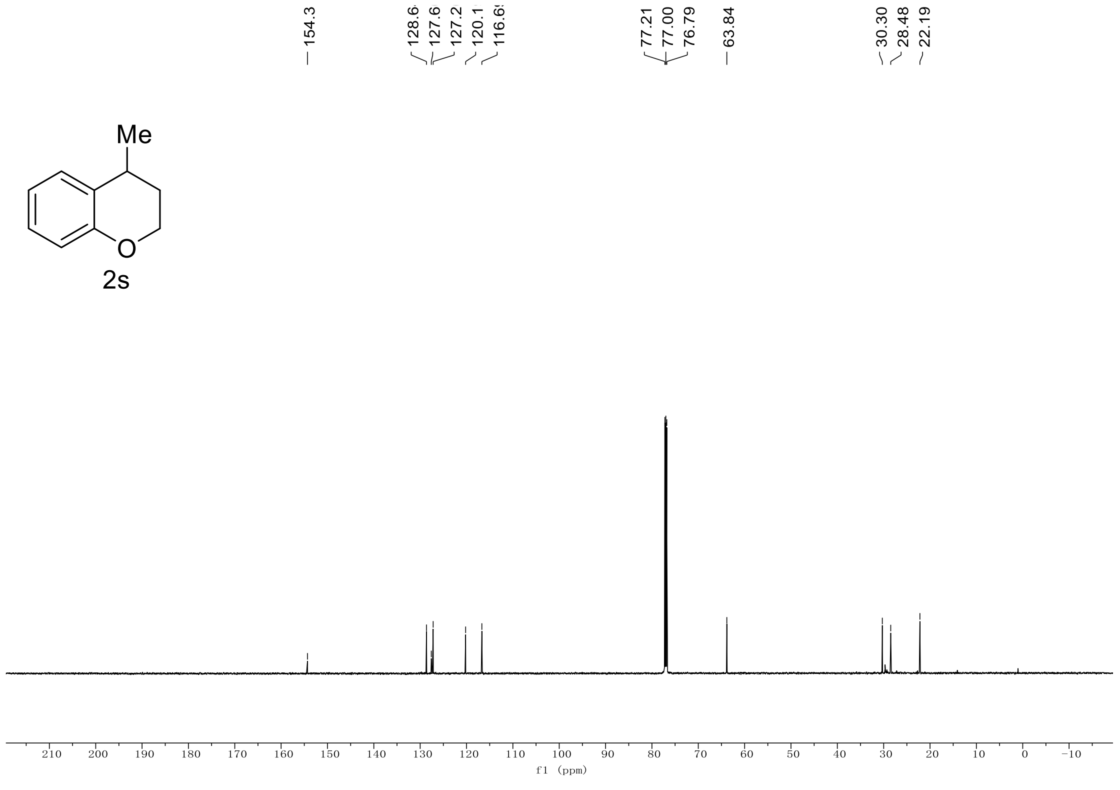

Supplementary Figure 158. ^{13}C NMR of compound **2s** (151 MHz, $CDCl_3$)

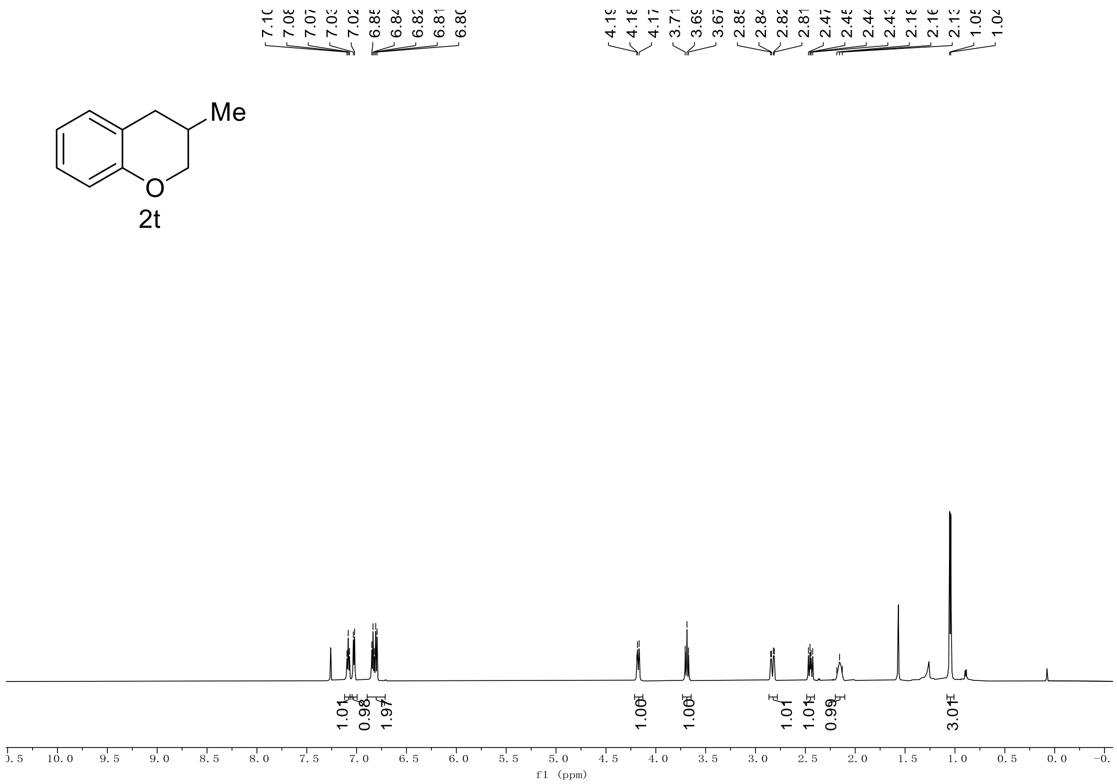

Supplementary Figure 159. ^{1}H NMR of compound **2t** (600 MHz, $CDCl_3$)

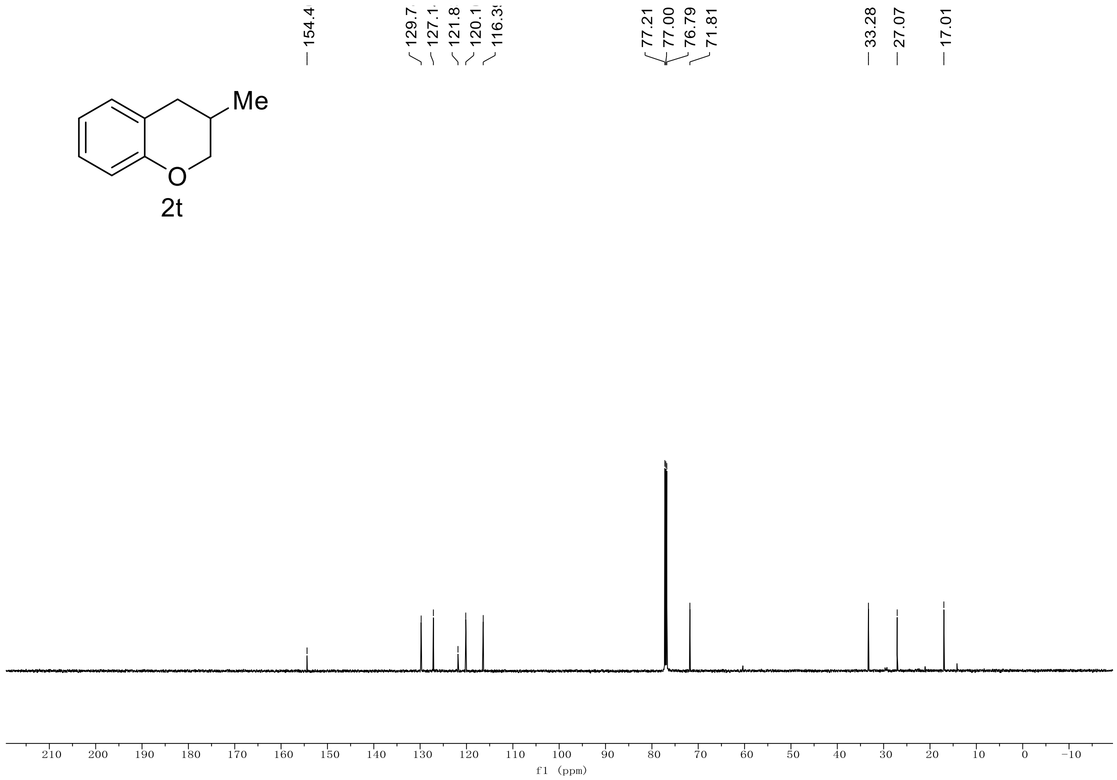

Supplementary Figure 160. ^{13}C NMR of compound **2t** (151 MHz, $CDCl_3$)

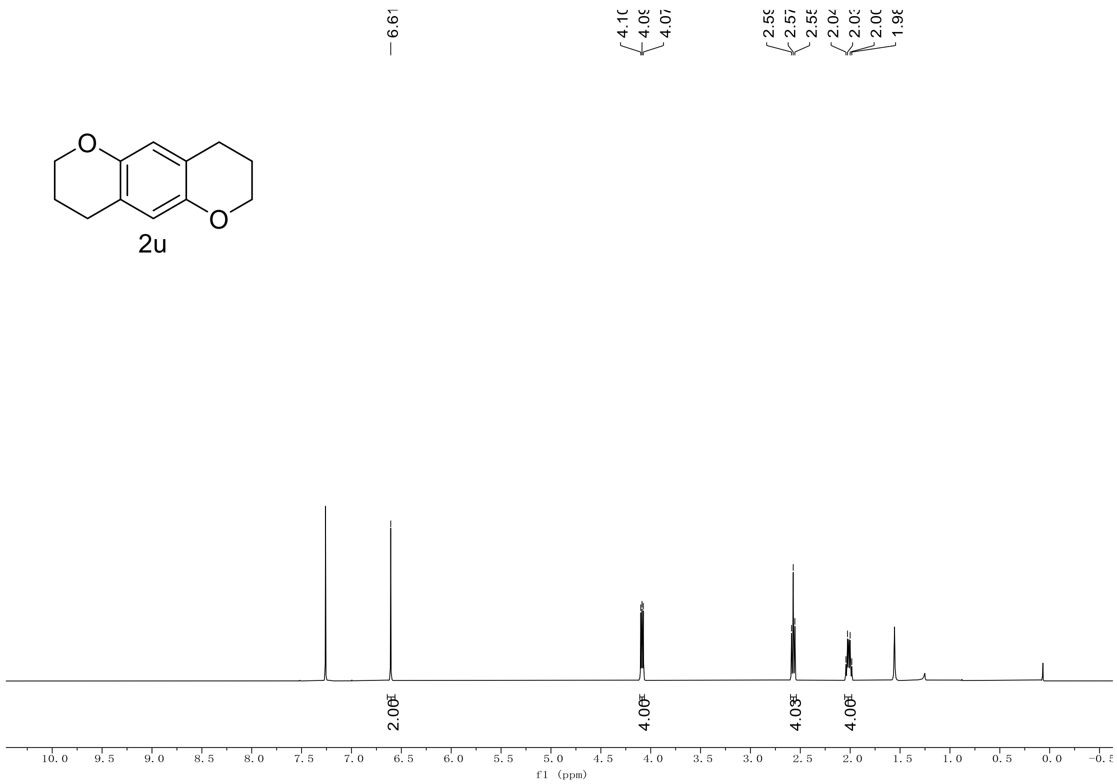

Supplementary Figure 161. ^{1}H NMR of compound **2u** (400 MHz, $CDCl_3$)

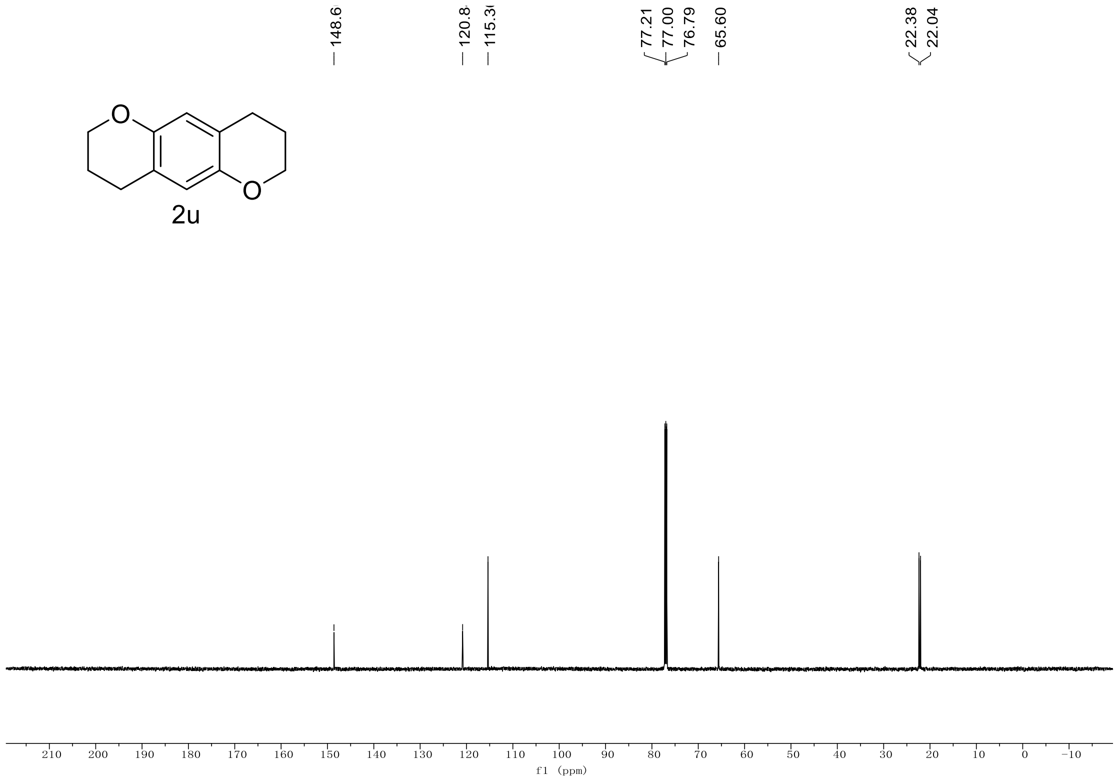

Supplementary Figure 162. ^{13}C NMR of compound **2u** (151 MHz, $CDCl_3$)

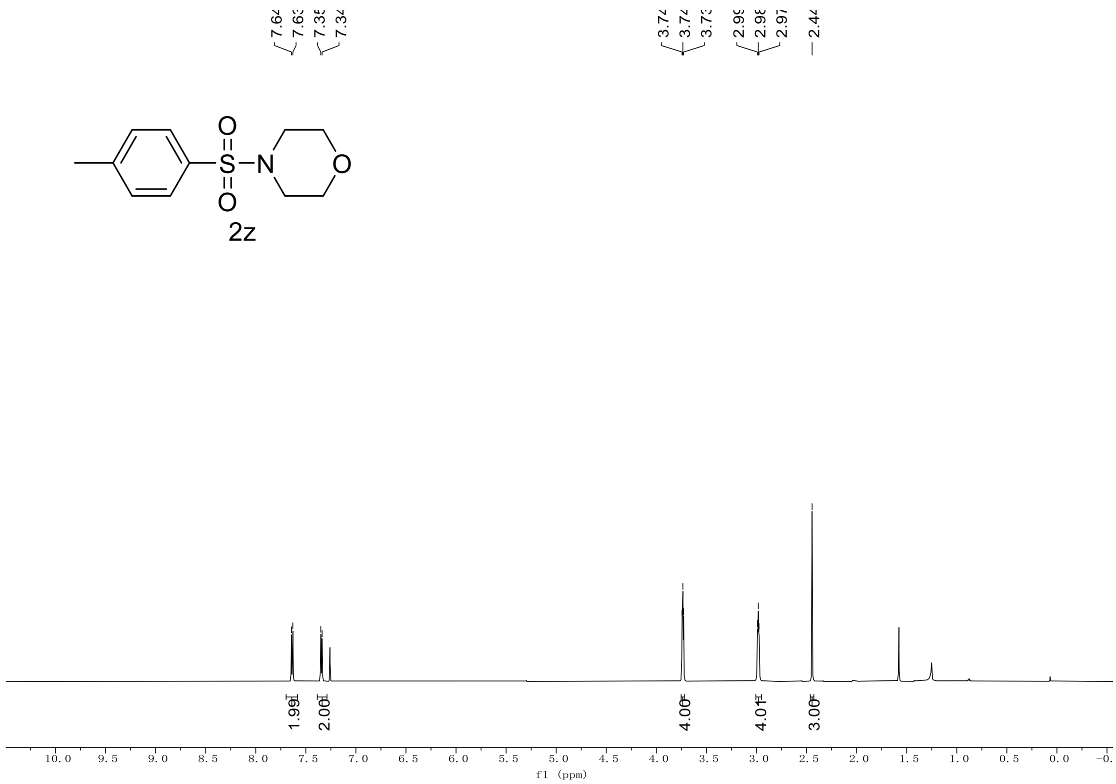

Supplementary Figure 163. ^{1}H NMR of compound **2z** (600 MHz, $CDCl_3$)

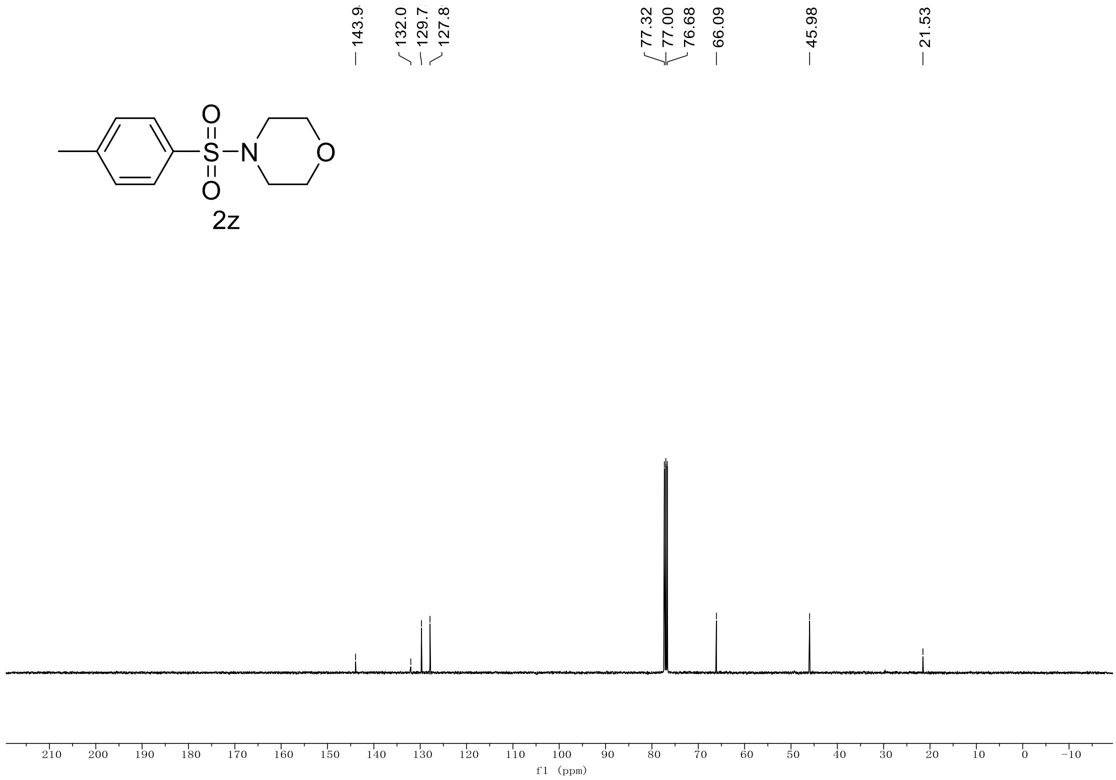

Supplementary Figure 164. ^{13}C NMR of compound **2z** (101 MHz, $CDCl_3$)

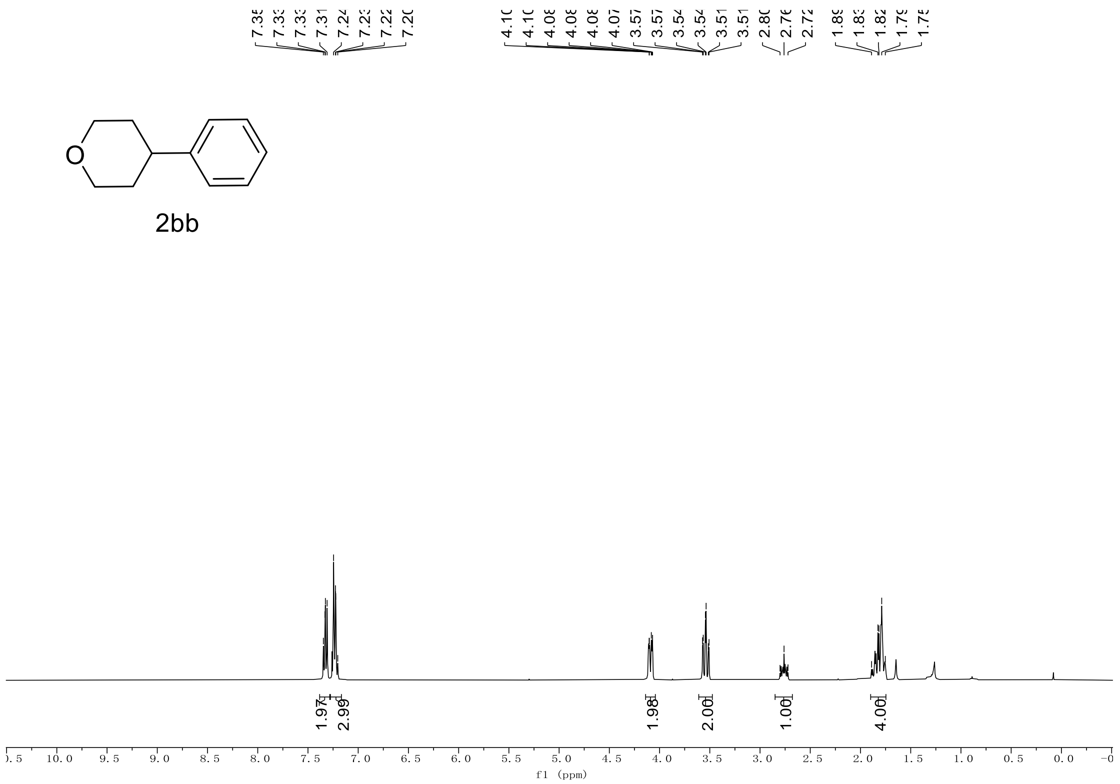

Supplementary Figure 165. ^{1}H NMR of compound **2bb** (400 MHz, $CDCl_3$)

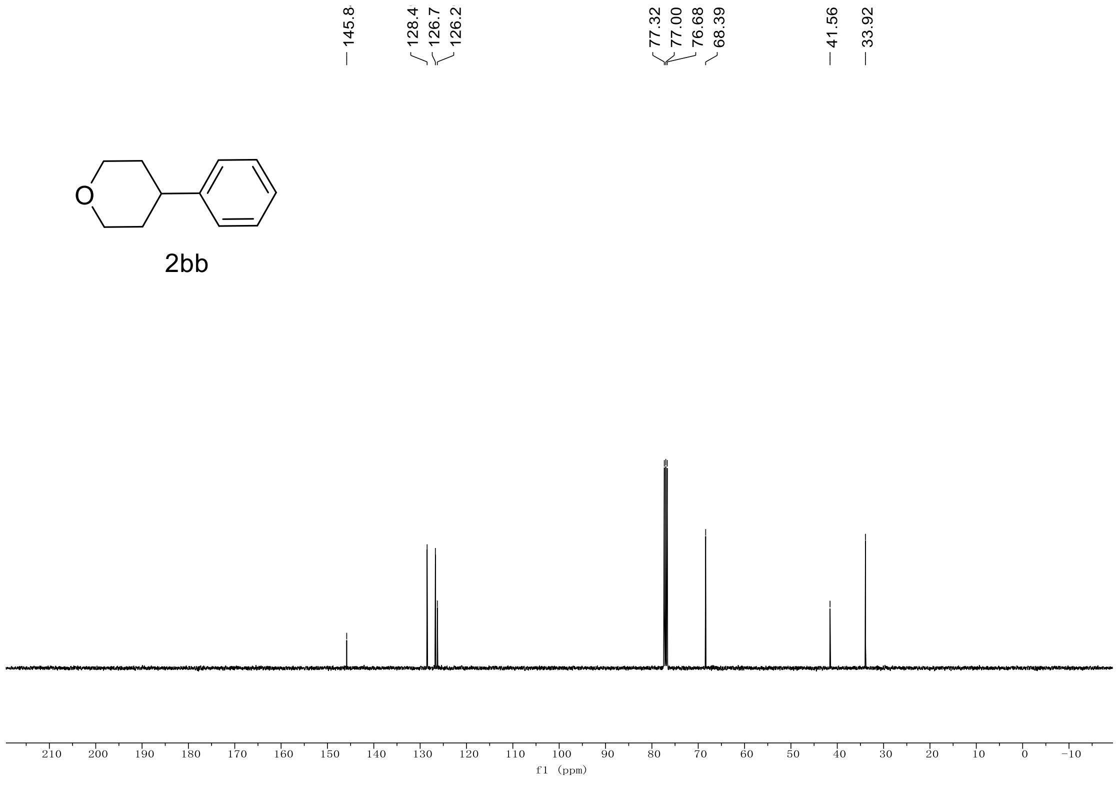

Supplementary Figure 166. ^{13}C NMR of compound **2bb** (101 MHz, $CDCl_3$)

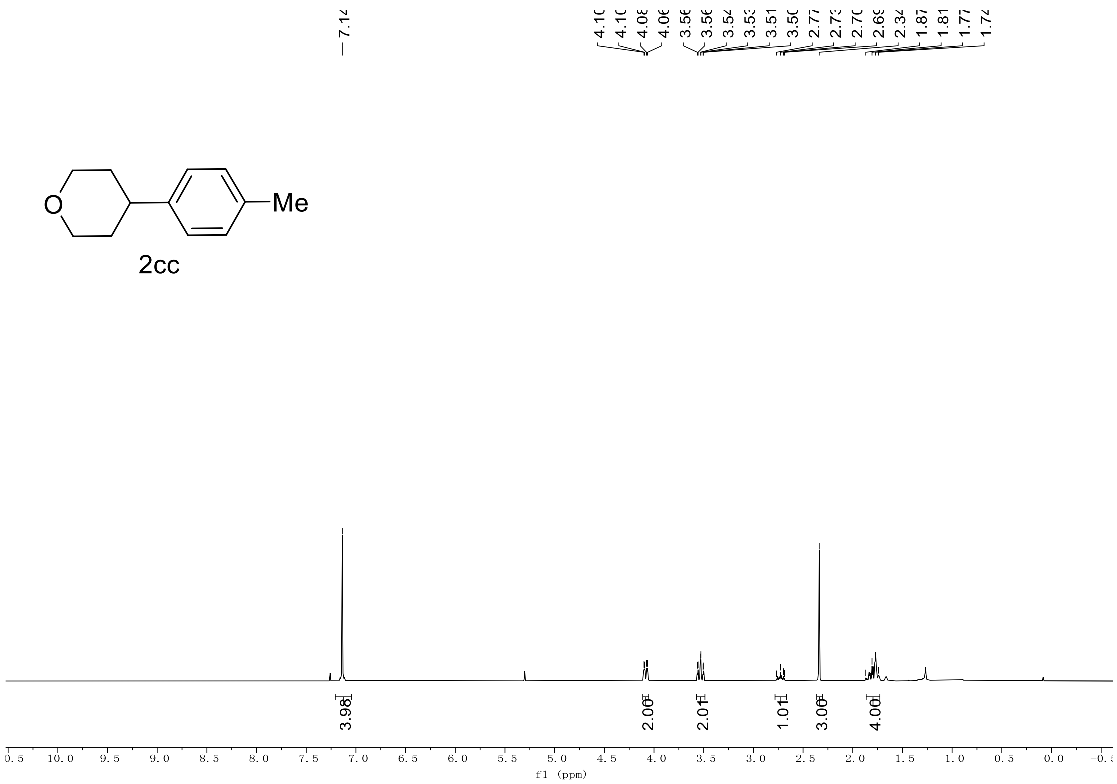

Supplementary Figure 167. ^{1}H NMR of compound **2cc** (400 MHz, $CDCl_3$)

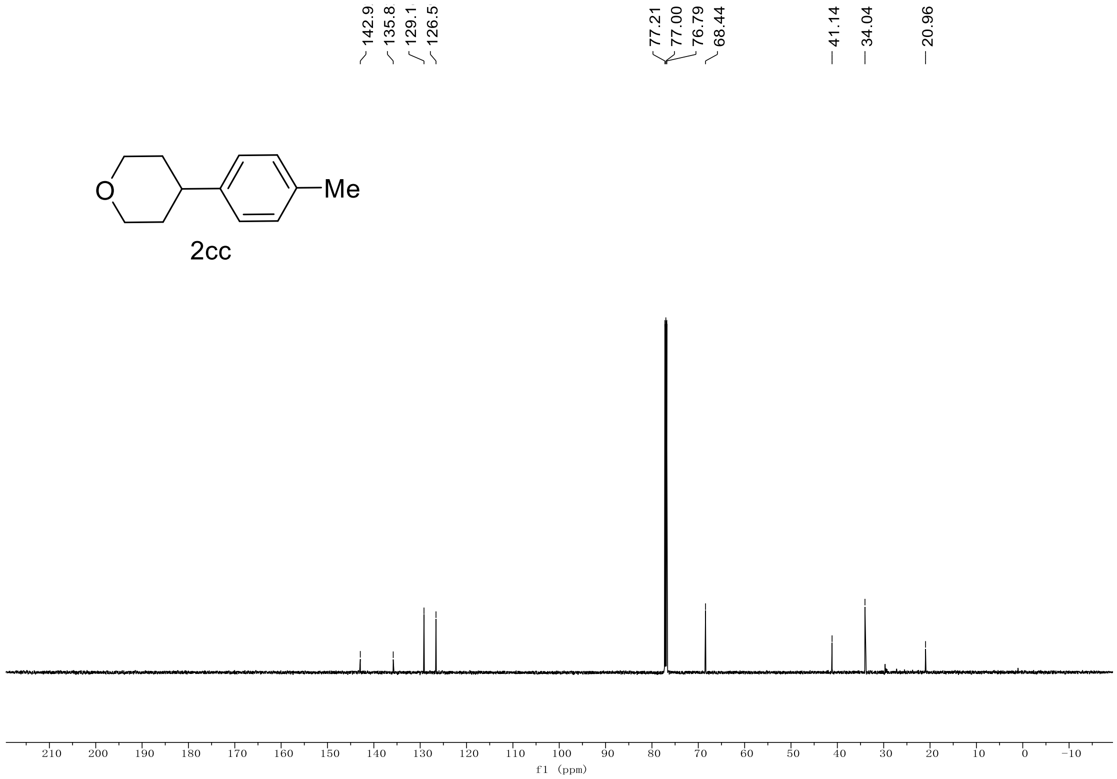

Supplementary Figure 168. ^{13}C NMR of compound **2cc** (101 MHz, $CDCl_3$)

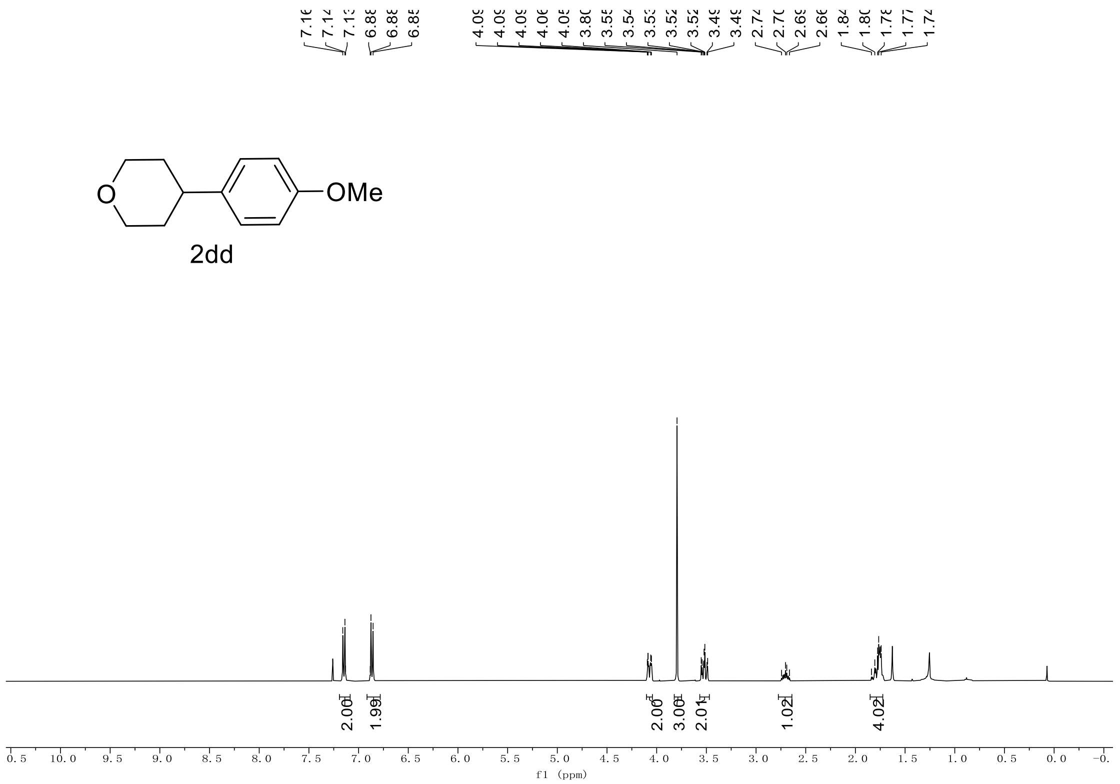

Supplementary Figure 169. ^{1}H NMR of compound **2dd** (400 MHz, $CDCl_3$)

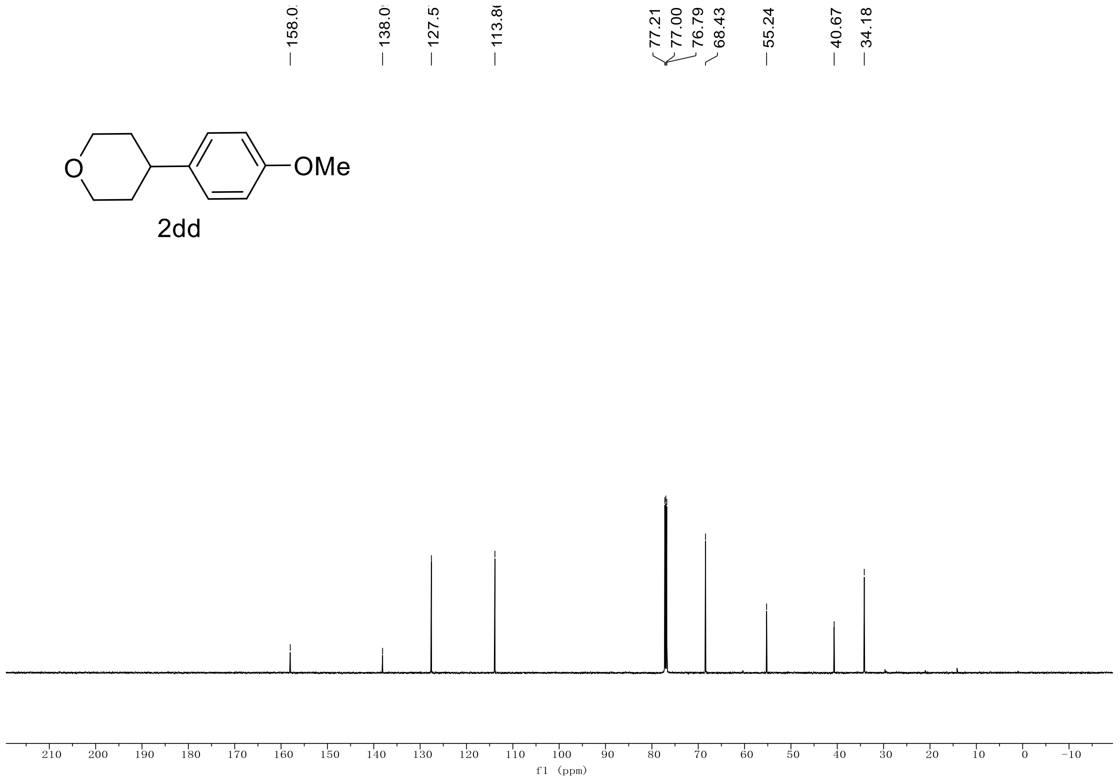

Supplementary Figure 170. ^{13}C NMR of compound **2dd** (151 MHz, $CDCl_3$)

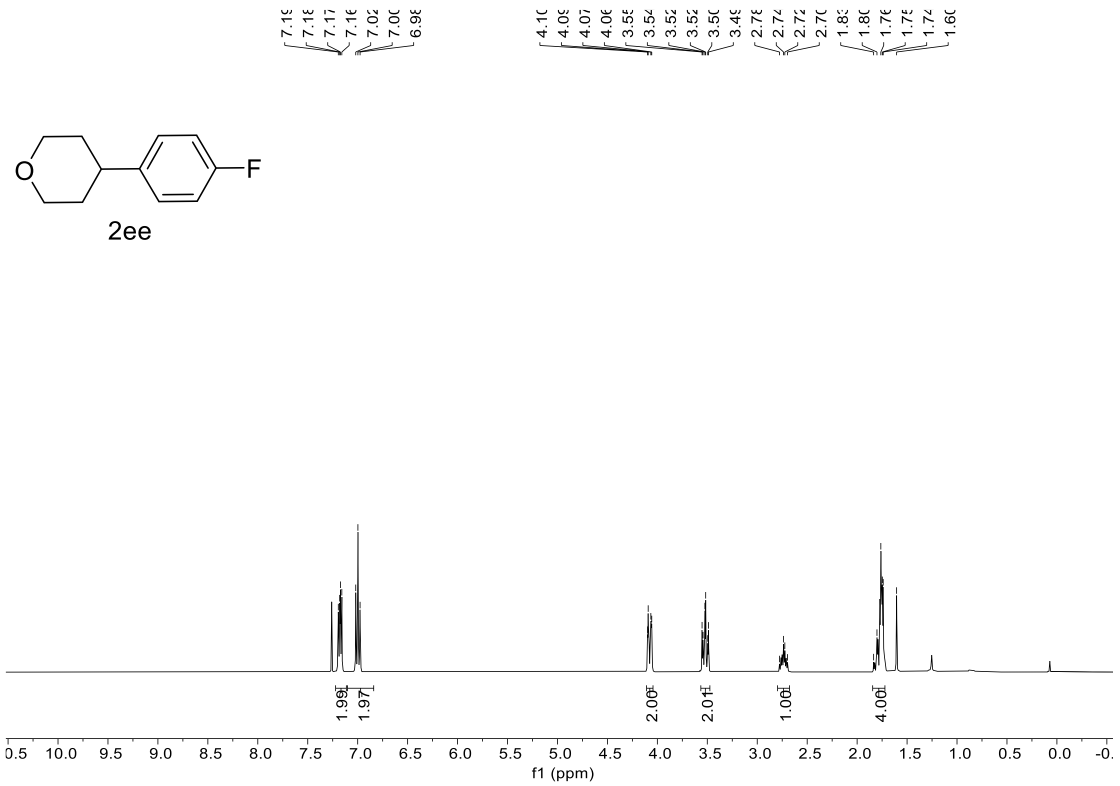

Supplementary Figure 171. ^{1}H NMR of compound **2ee** (400 MHz, $CDCl_3$)

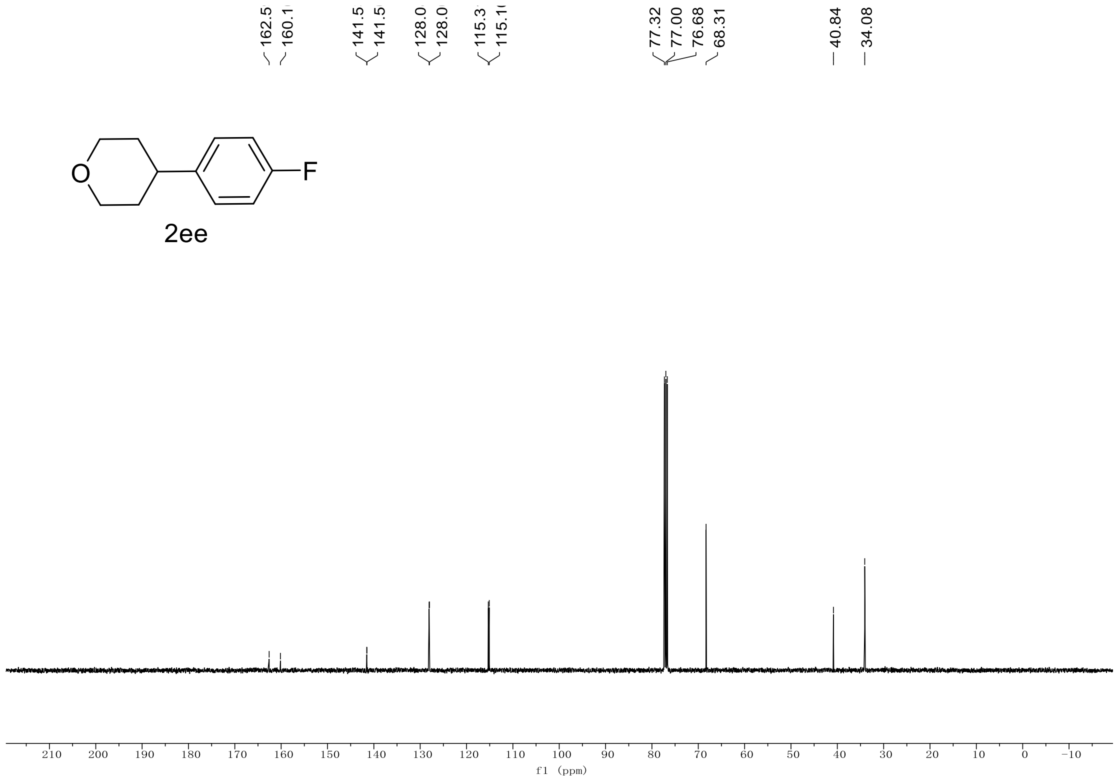

Supplementary Figure 172. ^{13}C NMR of compound **2ee** (101 MHz, $CDCl_3$)

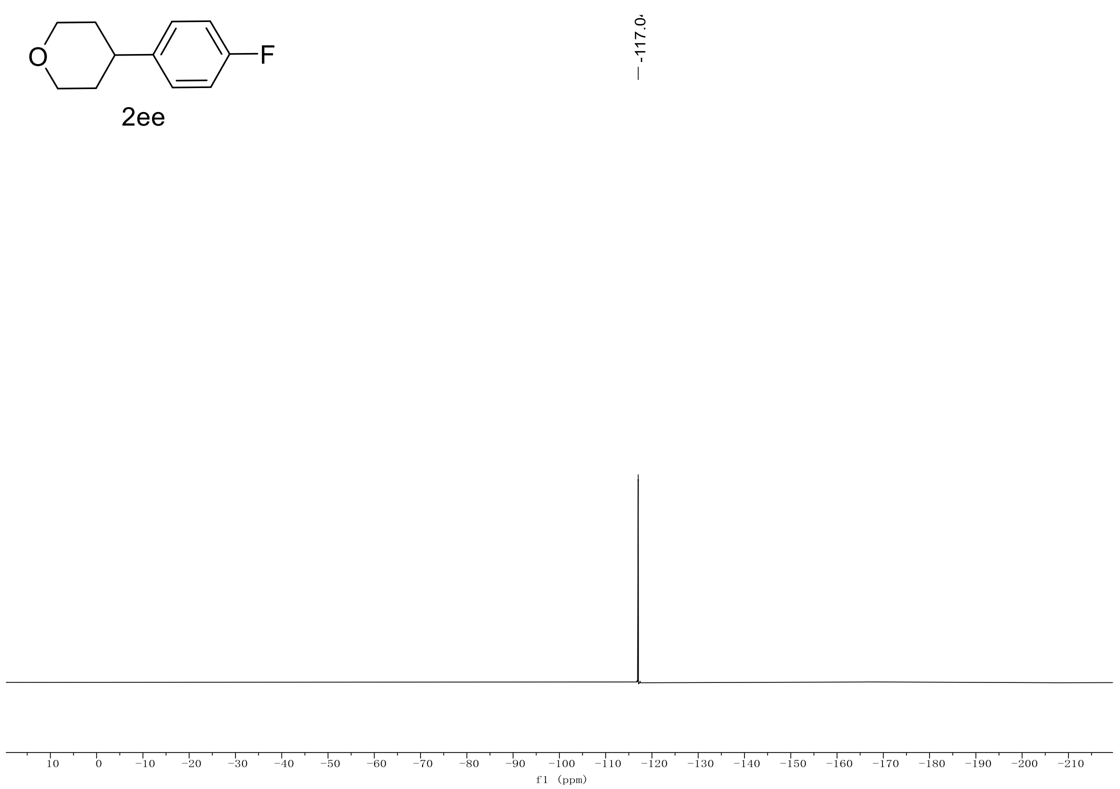

Supplementary Figure 173. ^{19}F NMR of compound **2ee** (565 MHz, $CDCl_3$)

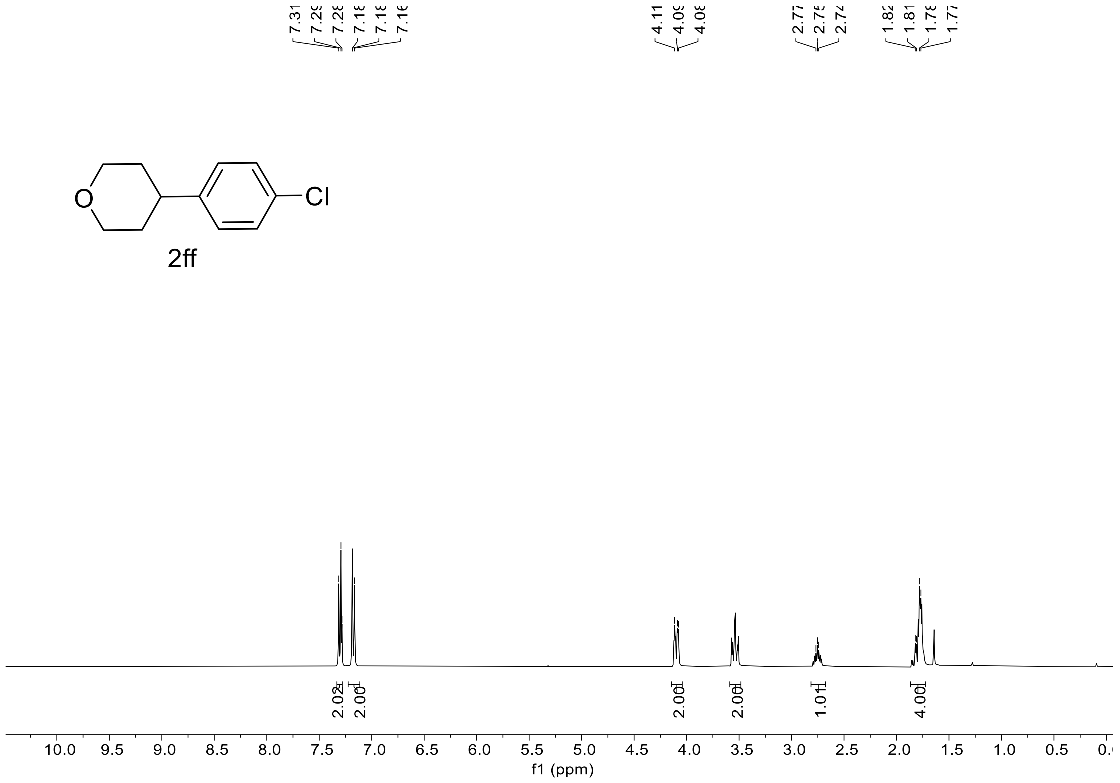

Supplementary Figure 174. ^{1}H NMR of compound **2ff** (400 MHz, $CDCl_3$)

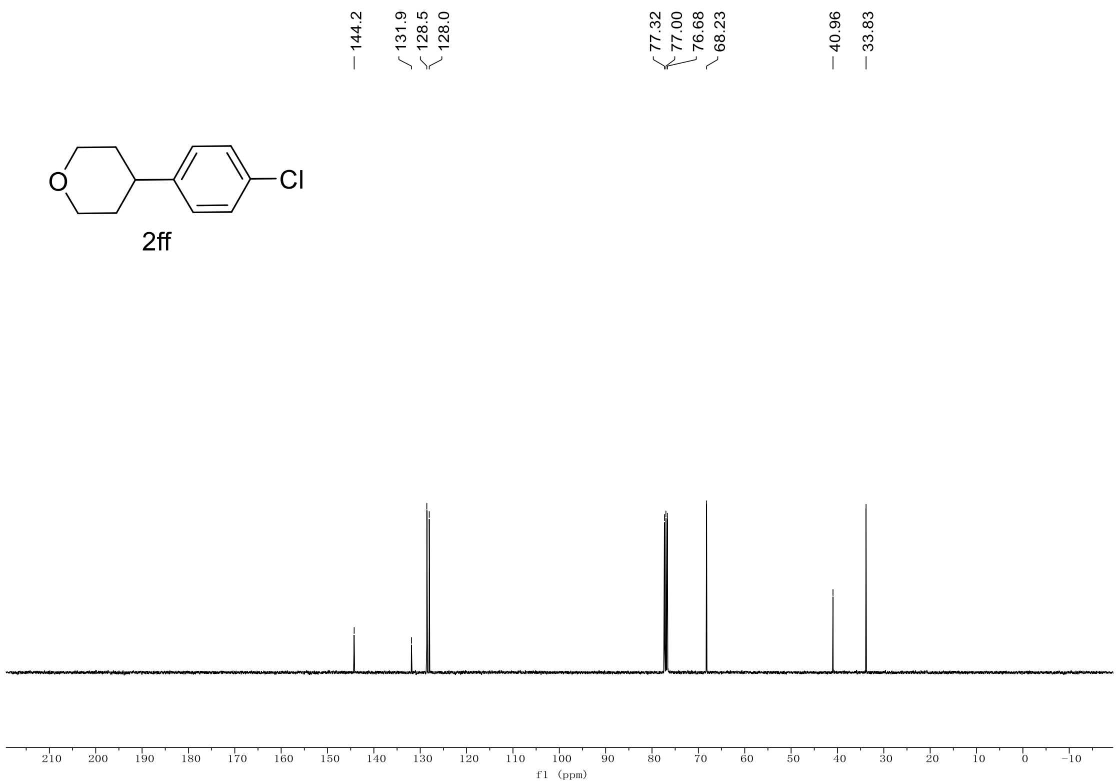

Supplementary Figure 175. ^{13}C NMR of compound **2ff** (101 MHz, $CDCl_3$)

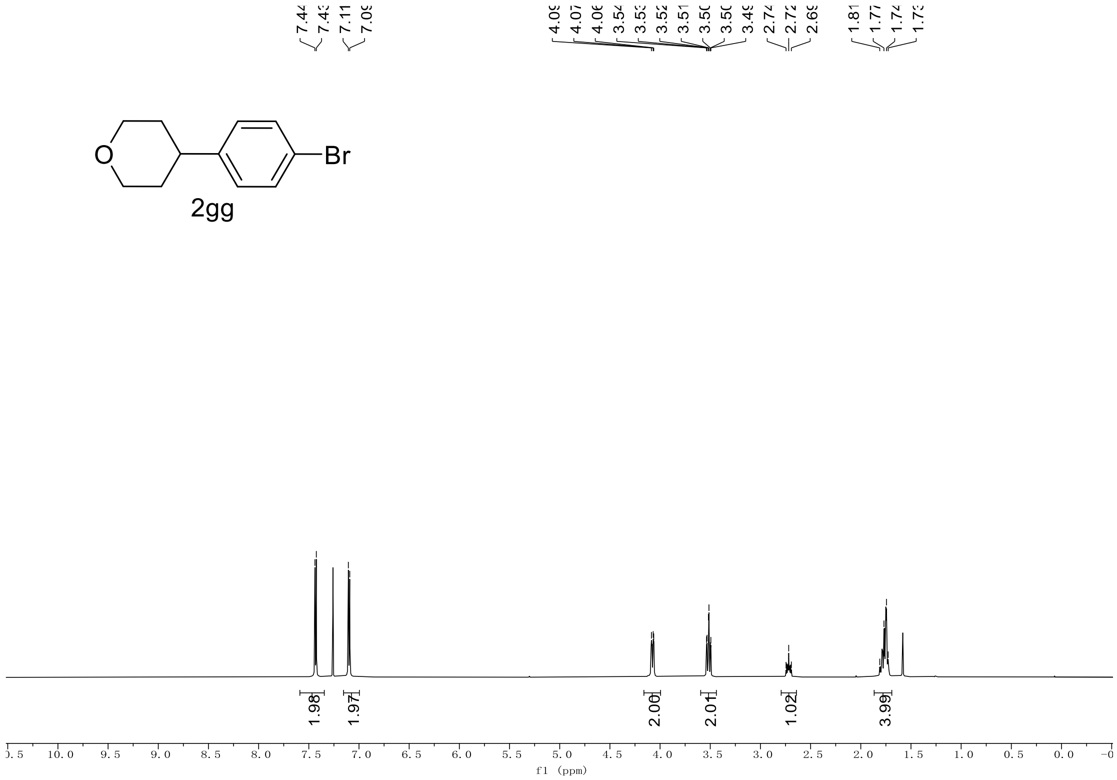

Supplementary Figure 176. ^{1}H NMR of compound **2gg** (600 MHz, $CDCl_3$)

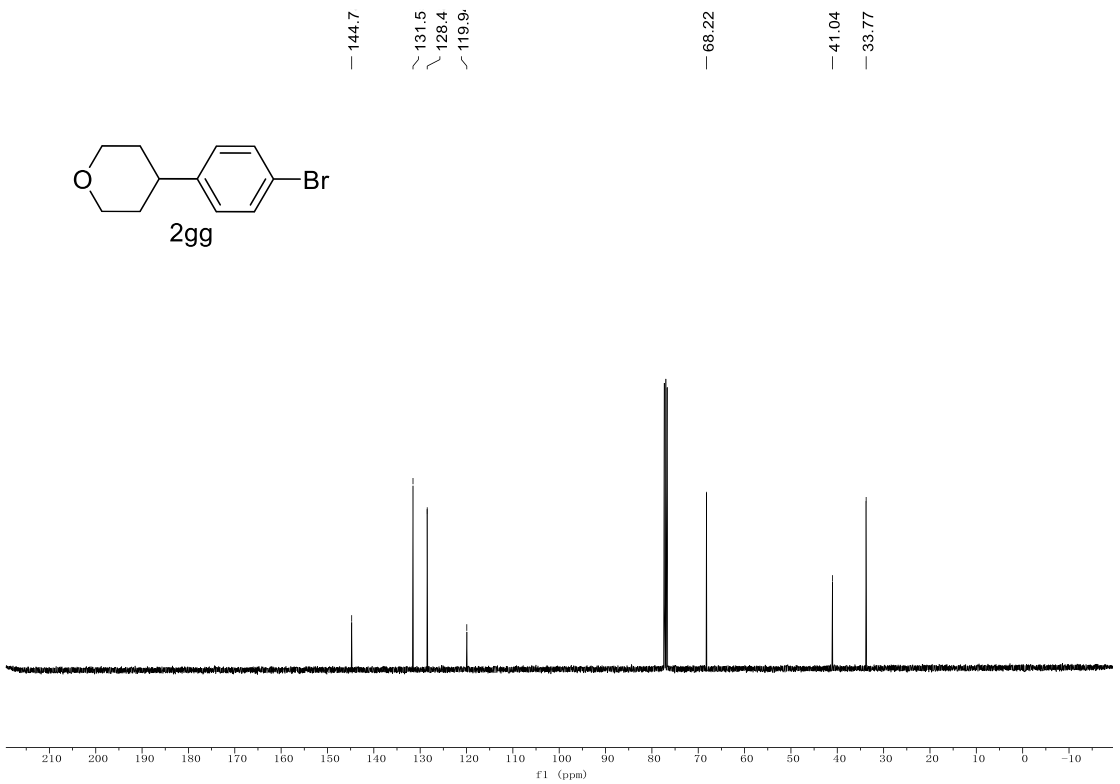

Supplementary Figure 177. ^{13}C NMR of compound **2gg** (101 MHz, $CDCl_3$)

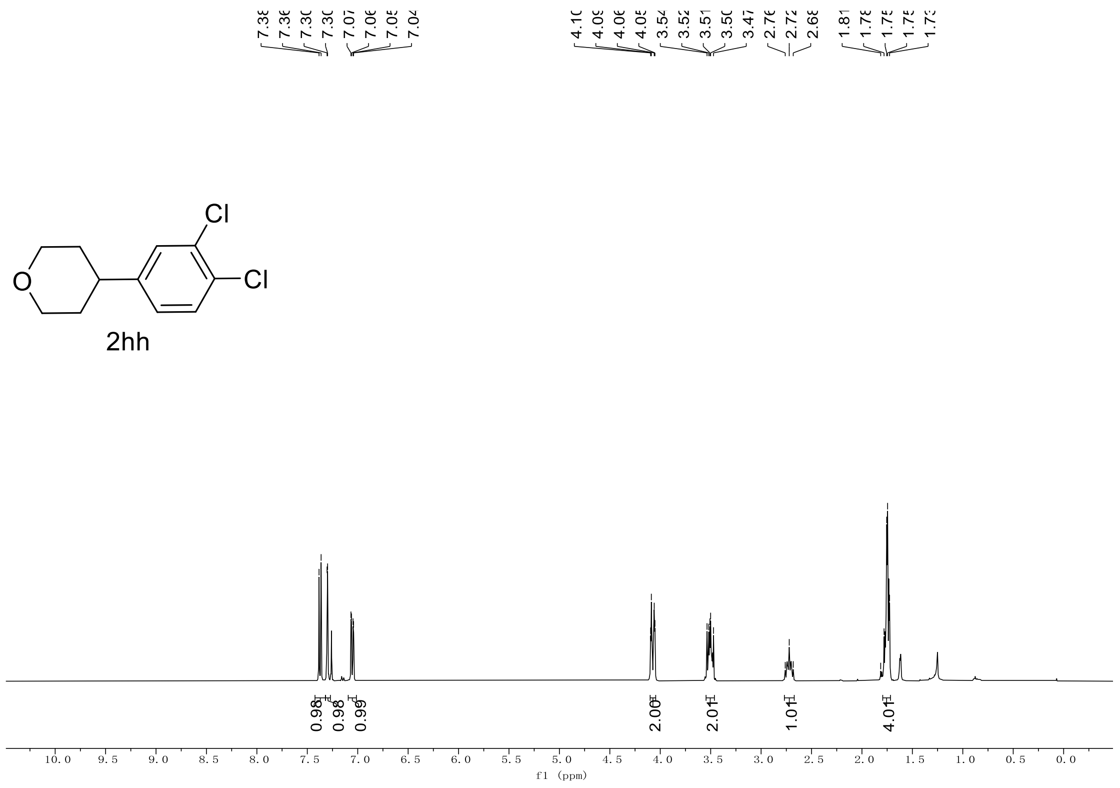

Supplementary Figure 178. ^{1}H NMR of compound **2hh** (400 MHz, $CDCl_3$)

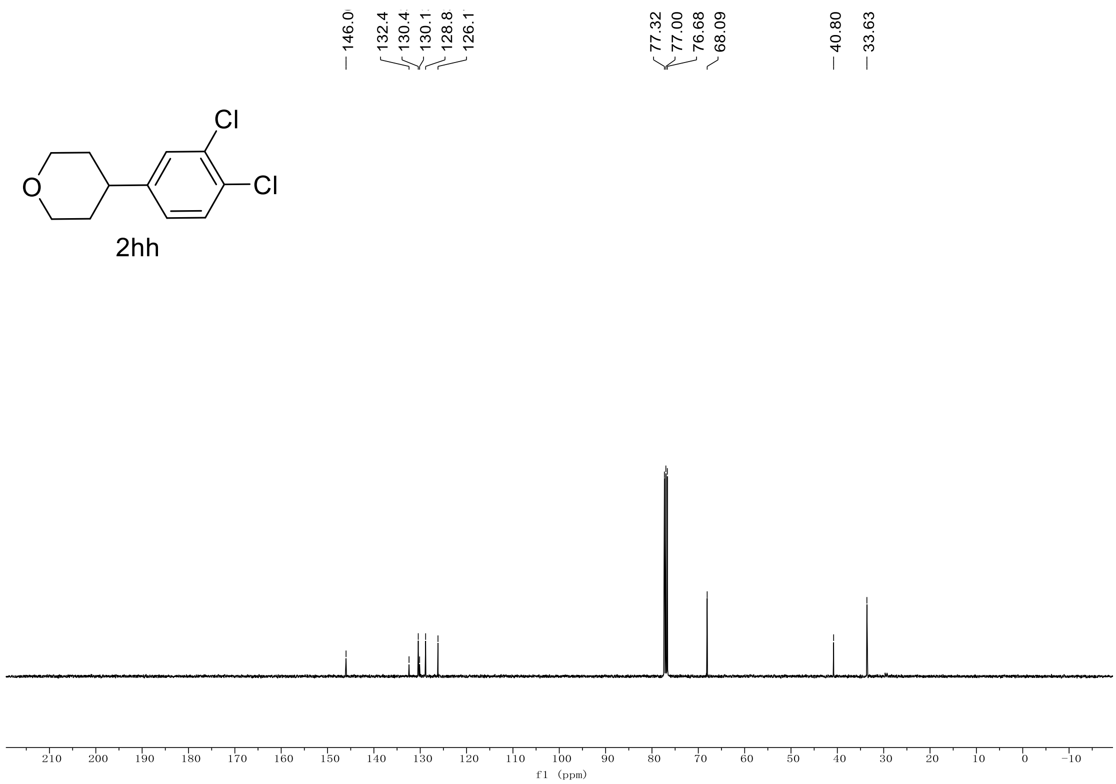

Supplementary Figure 179. ^{13}C NMR of compound **2hh** (101 MHz, $CDCl_3$)

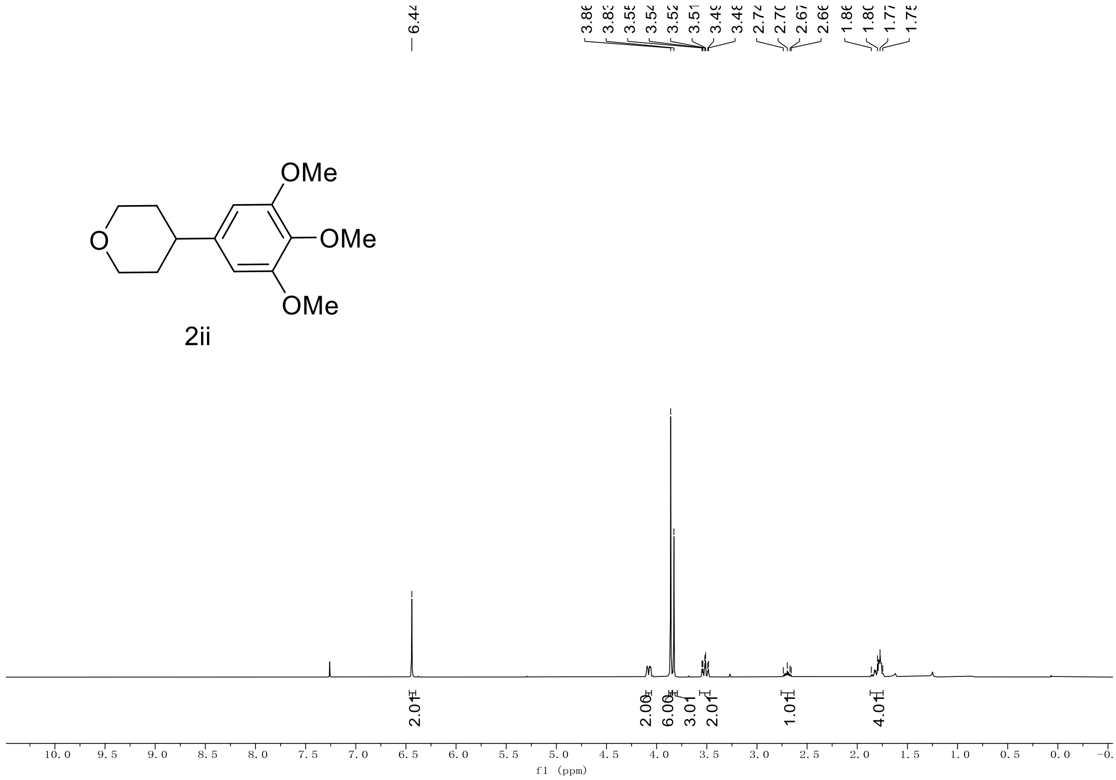

Supplementary Figure 180. ^{1}H NMR of compound **2ii** (400 MHz, $CDCl_3$)

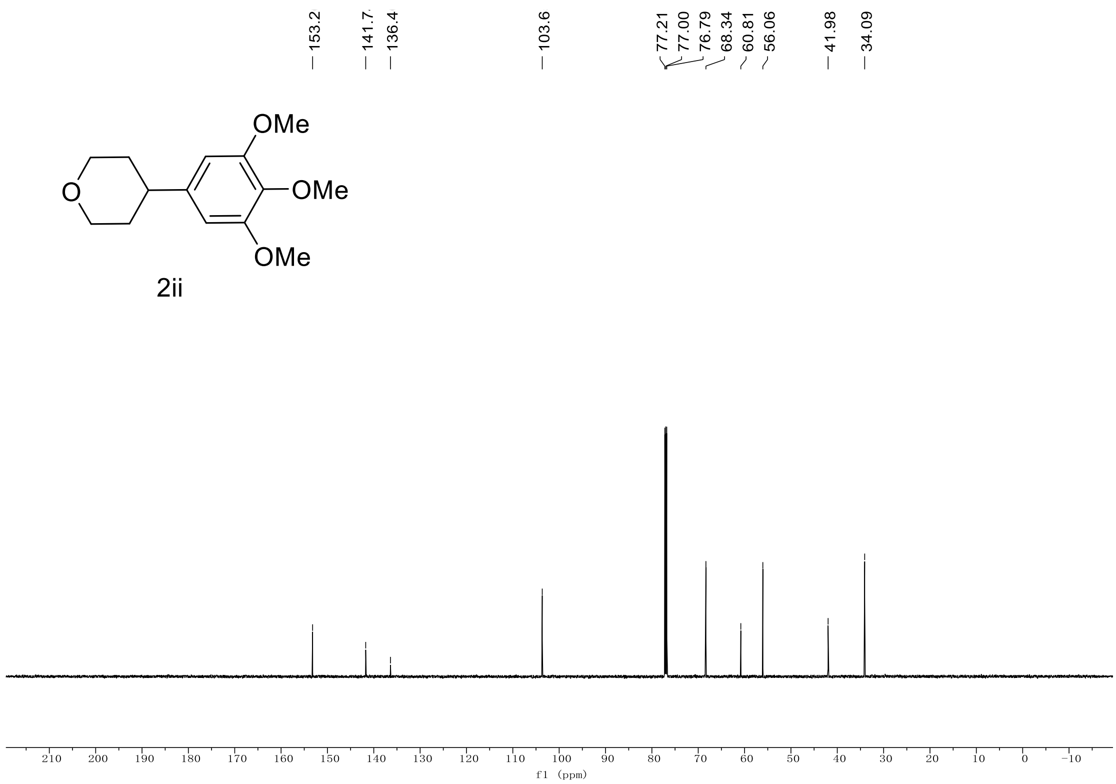

2ii

Supplementary Figure 181. ^{13}C NMR of compound **2ii** (151 MHz, $CDCl_3$)

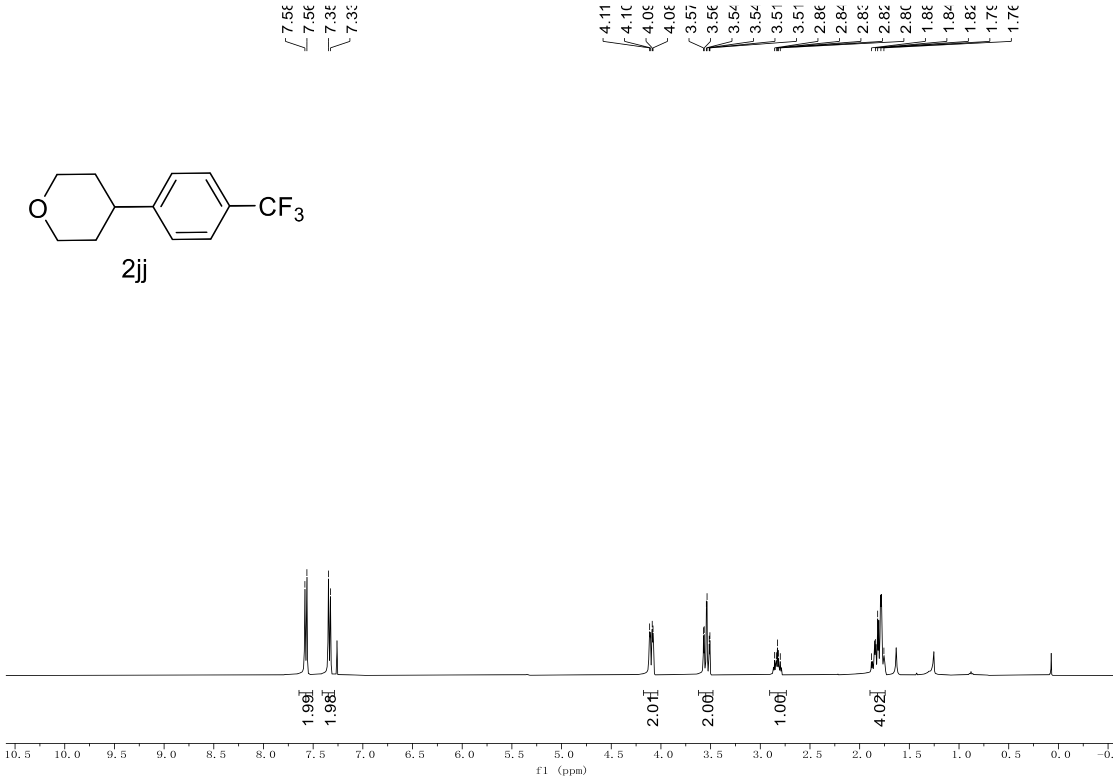

2jj

Supplementary Figure 182. ^{1}H NMR of compound **2jj** (400 MHz, $CDCl_3$)

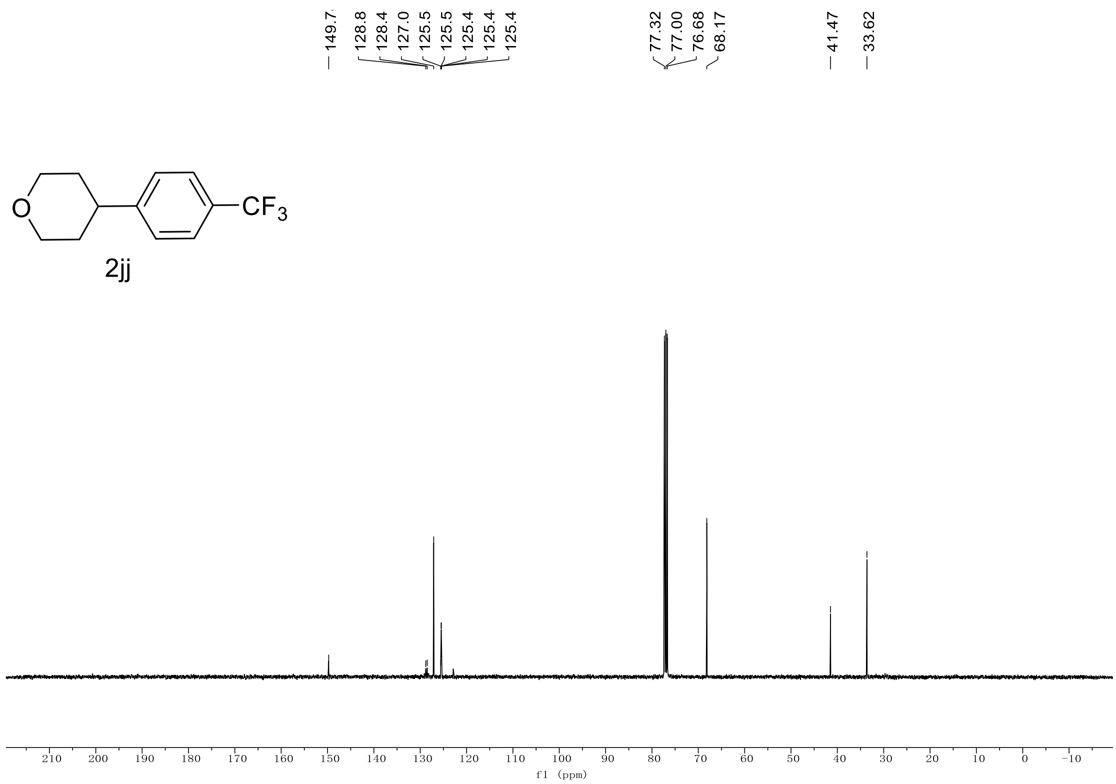

Supplementary Figure 183. ^{13}C NMR of compound **2jj** (101 MHz, $CDCl_3$)

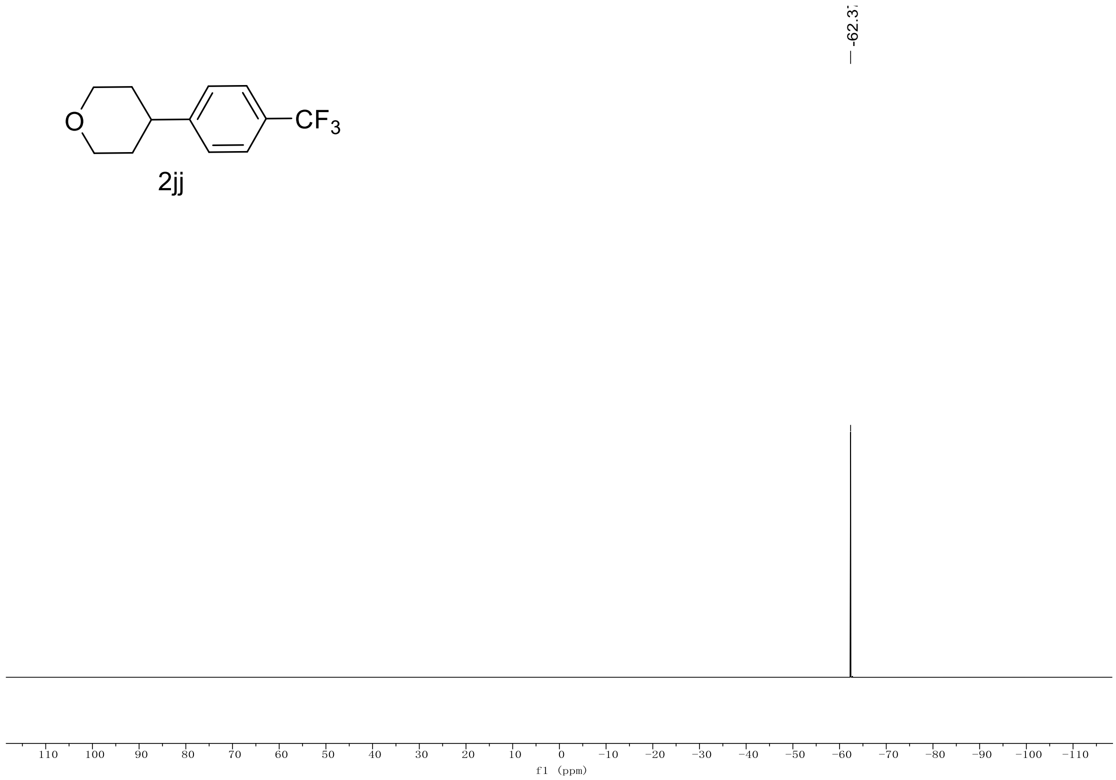

Supplementary Figure 184. ^{19}F NMR of compound **2jj** (565 MHz, $CDCl_3$)

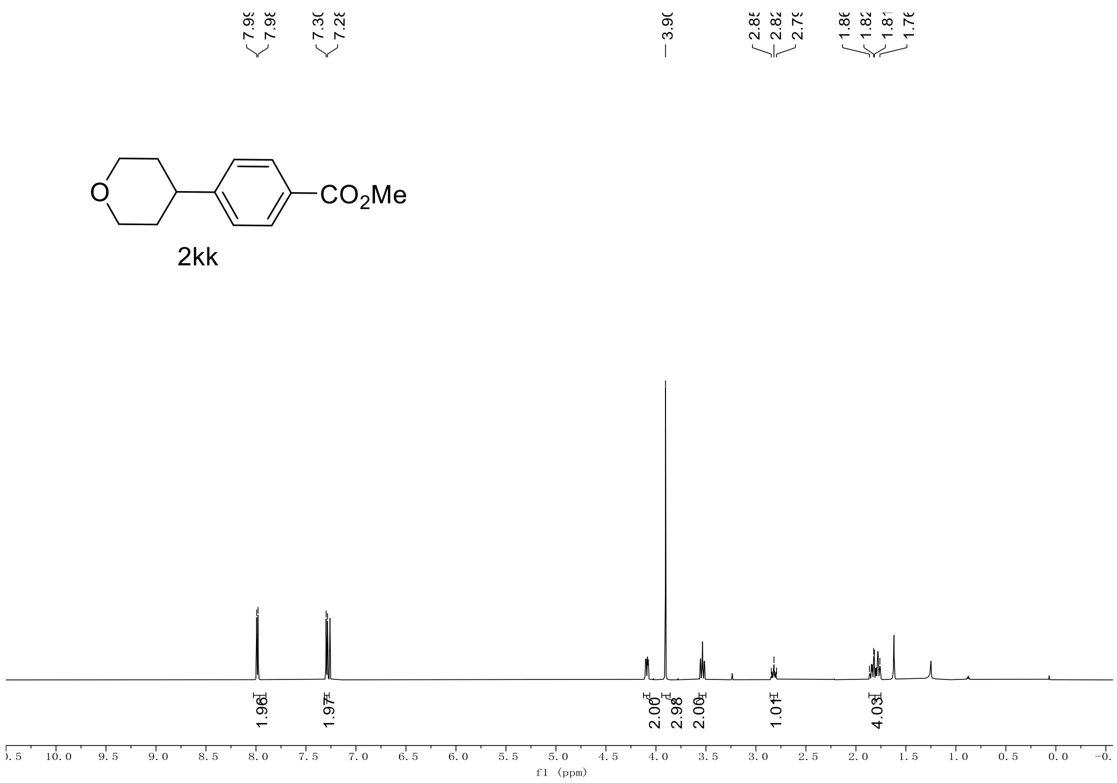

Supplementary Figure 185. ^{1}H NMR of compound **2kk** (600 MHz, $CDCl_3$)

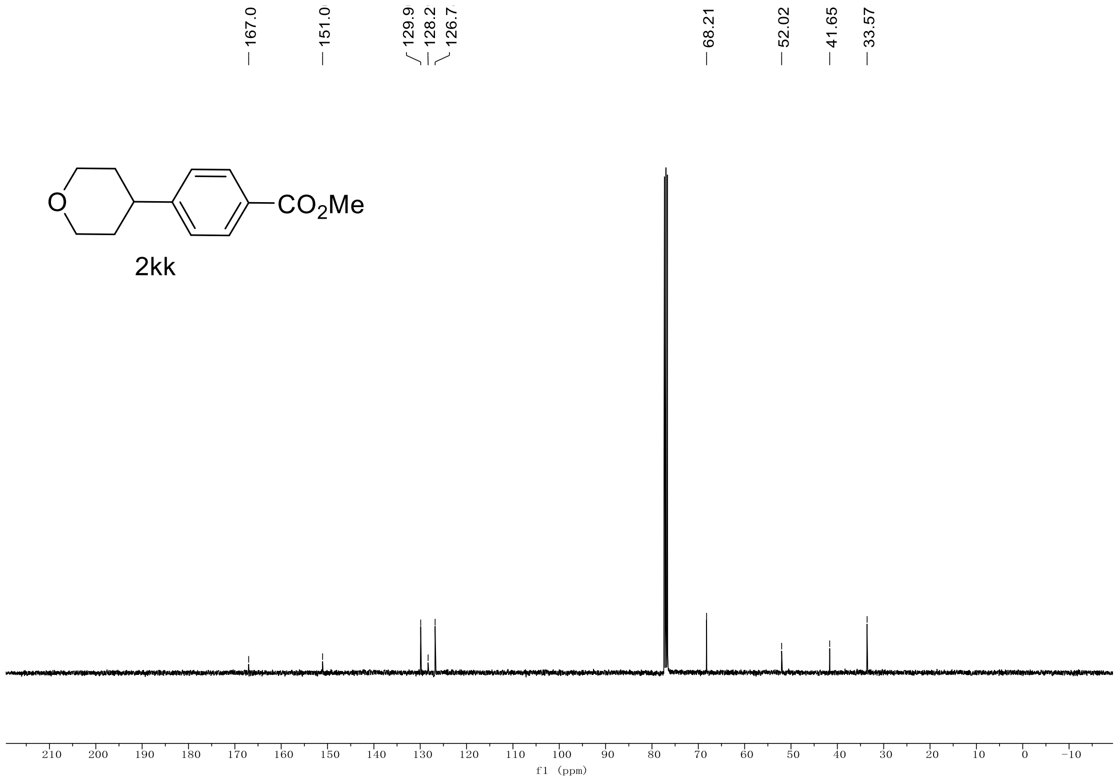

Supplementary Figure 186. ^{13}C NMR of compound **2kk** (101 MHz, $CDCl_3$)

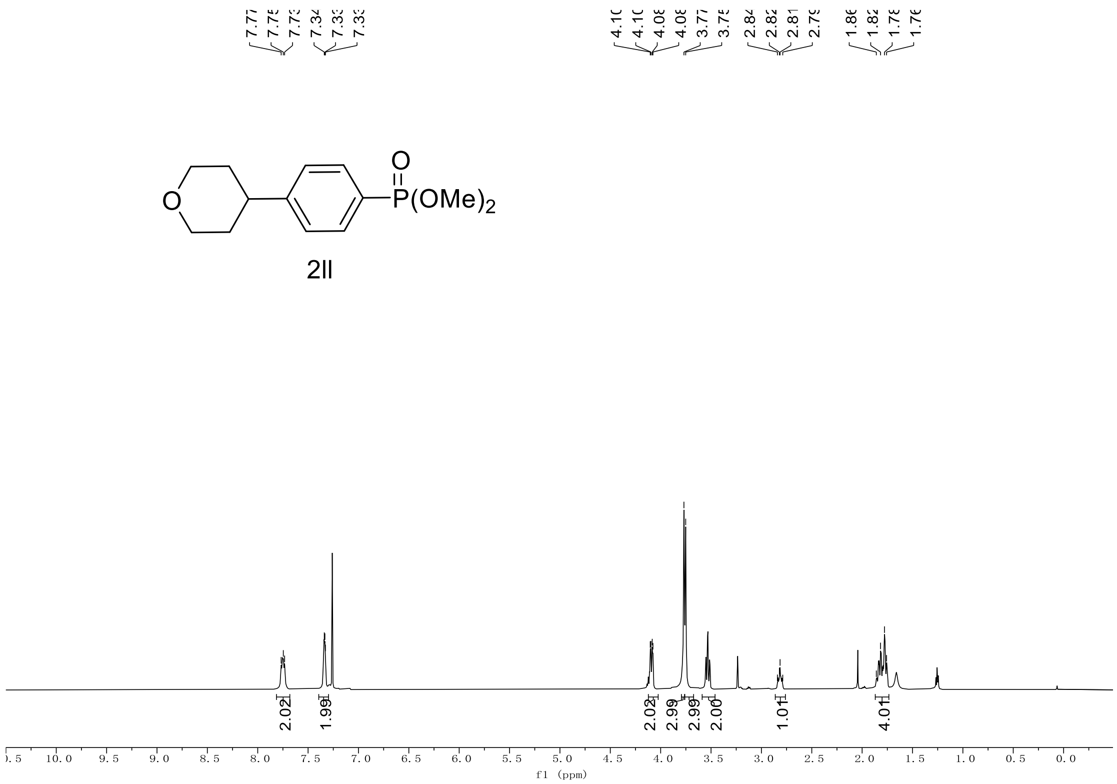

Supplementary Figure 187. ^{1}H NMR of compound **2ll** (600 MHz, $CDCl_3$)

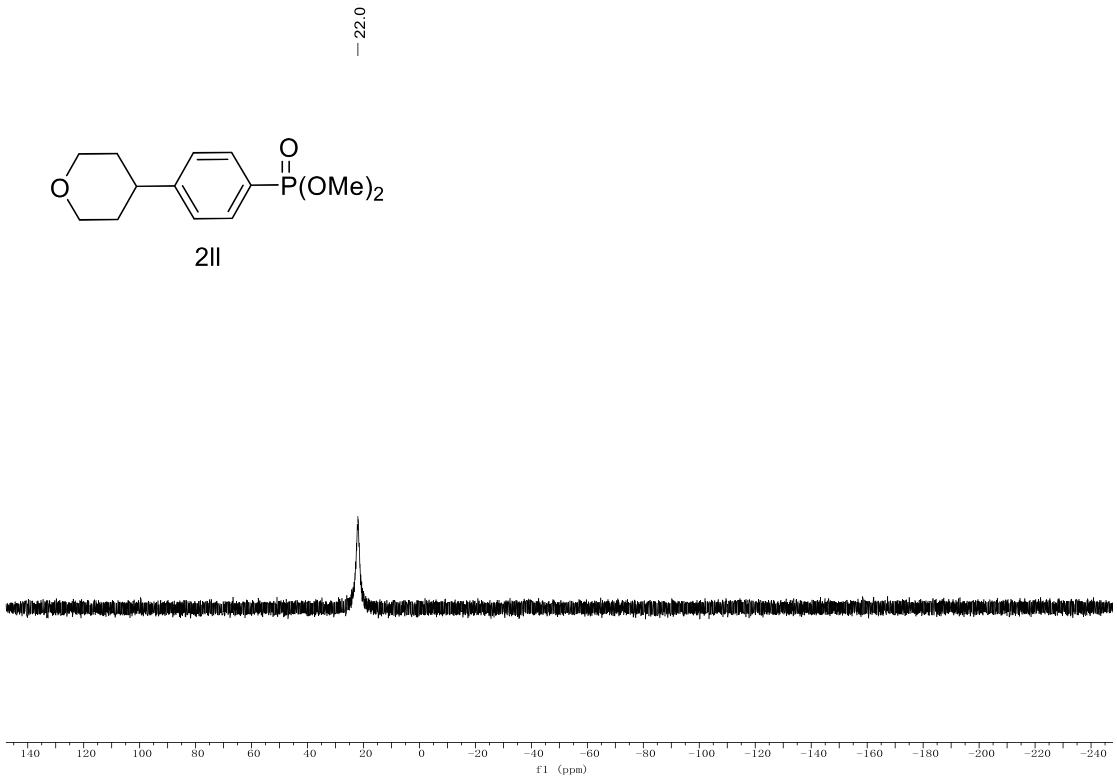

Supplementary Figure 188. ^{31}P NMR of compound **2ll** (243 MHz, $CDCl_3$)

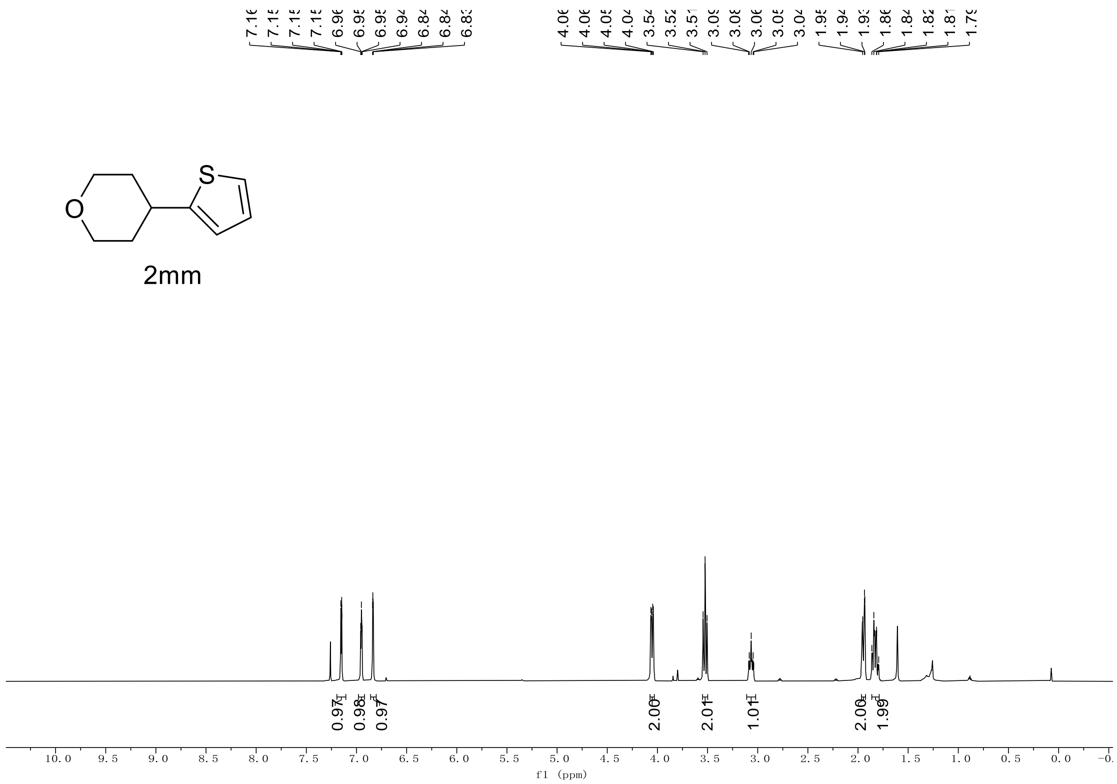

Supplementary Figure 189. ^{1}H NMR of compound **2mm** (600 MHz, $CDCl_3$)

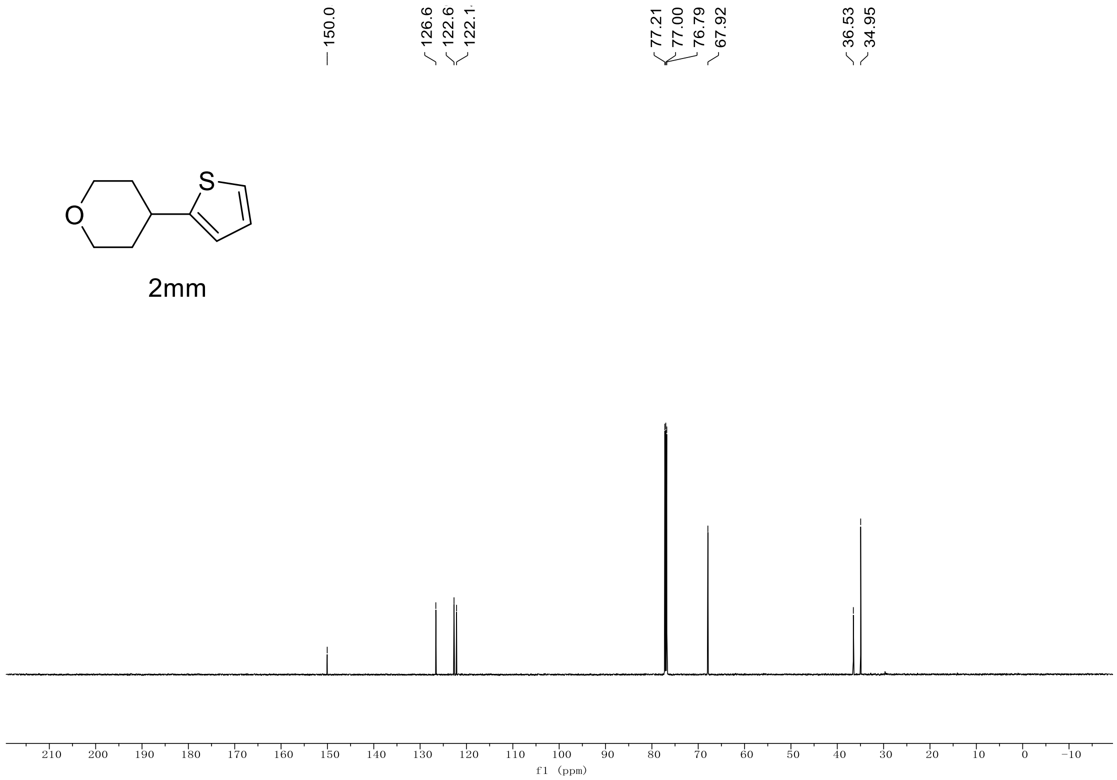

Supplementary Figure 190. ^{13}C NMR of compound **2mm** (101 MHz, $CDCl_3$)

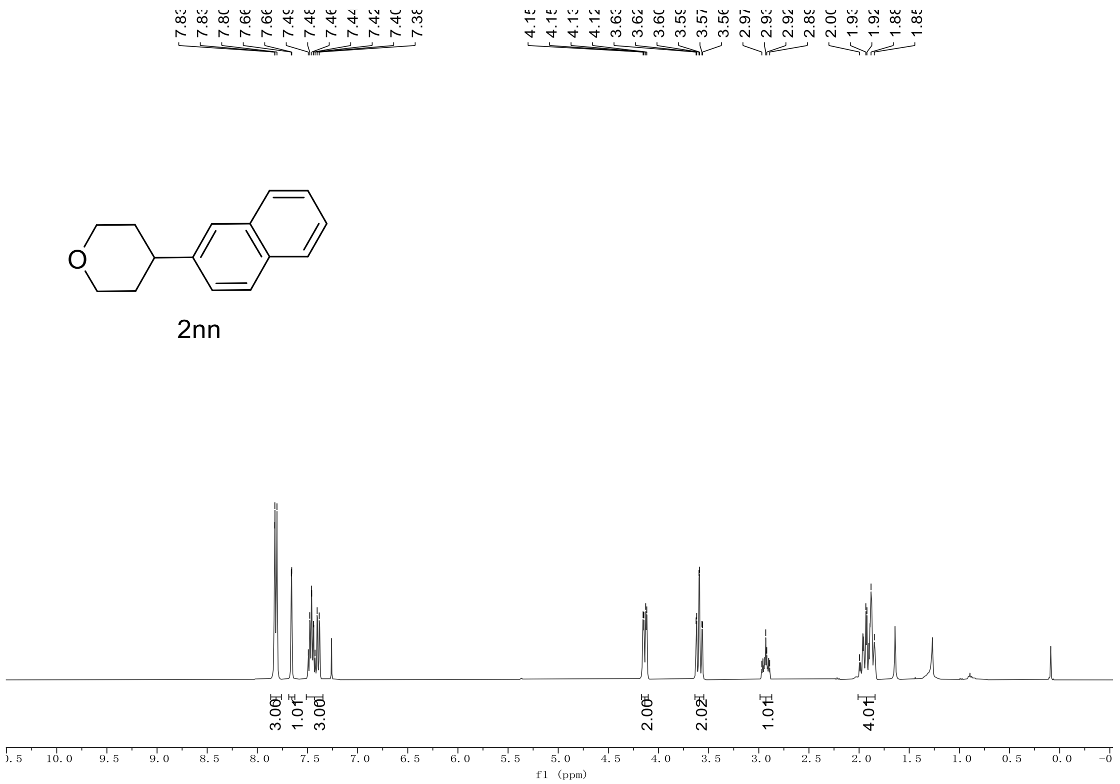

Supplementary Figure 191. ^{1}H NMR of compound **2nn** (400 MHz, $CDCl_3$)

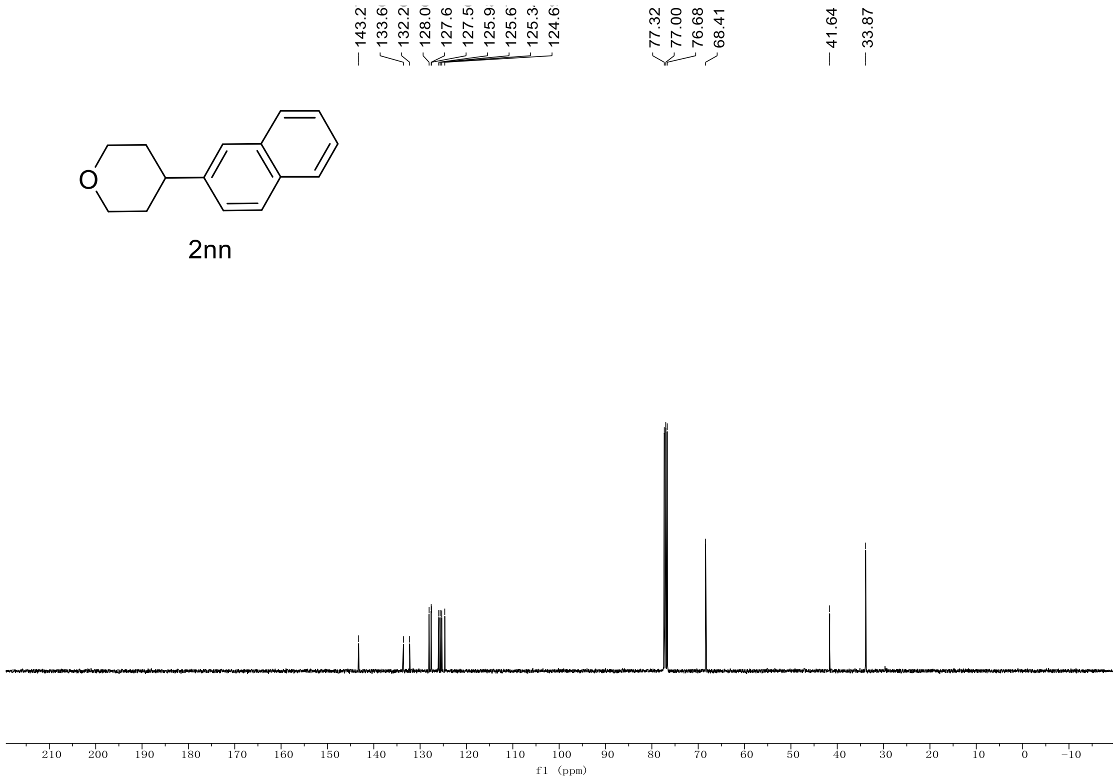

Supplementary Figure 192. ^{13}C NMR of compound **2nn** (101 MHz, $CDCl_3$)

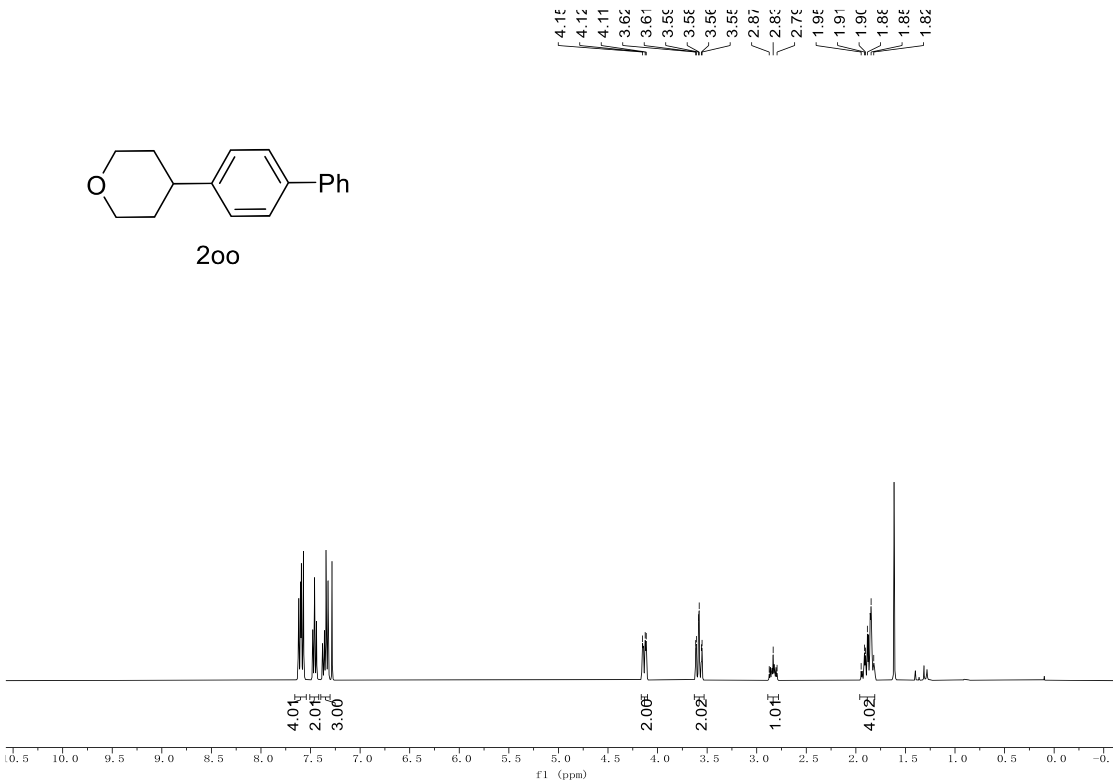

Supplementary Figure 193. ^{1}H NMR of compound **2oo** (400 MHz, $CDCl_3$)

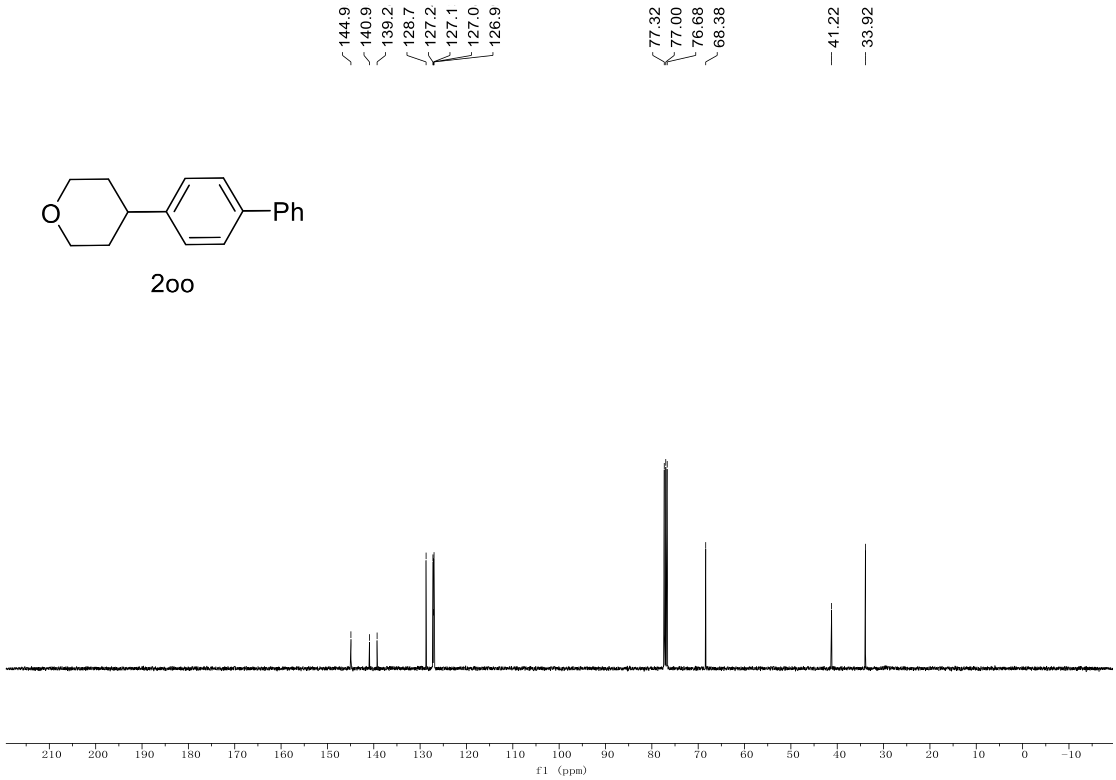

Supplementary Figure 194. ^{13}C NMR of compound **2oo** (101 MHz, $CDCl_3$)

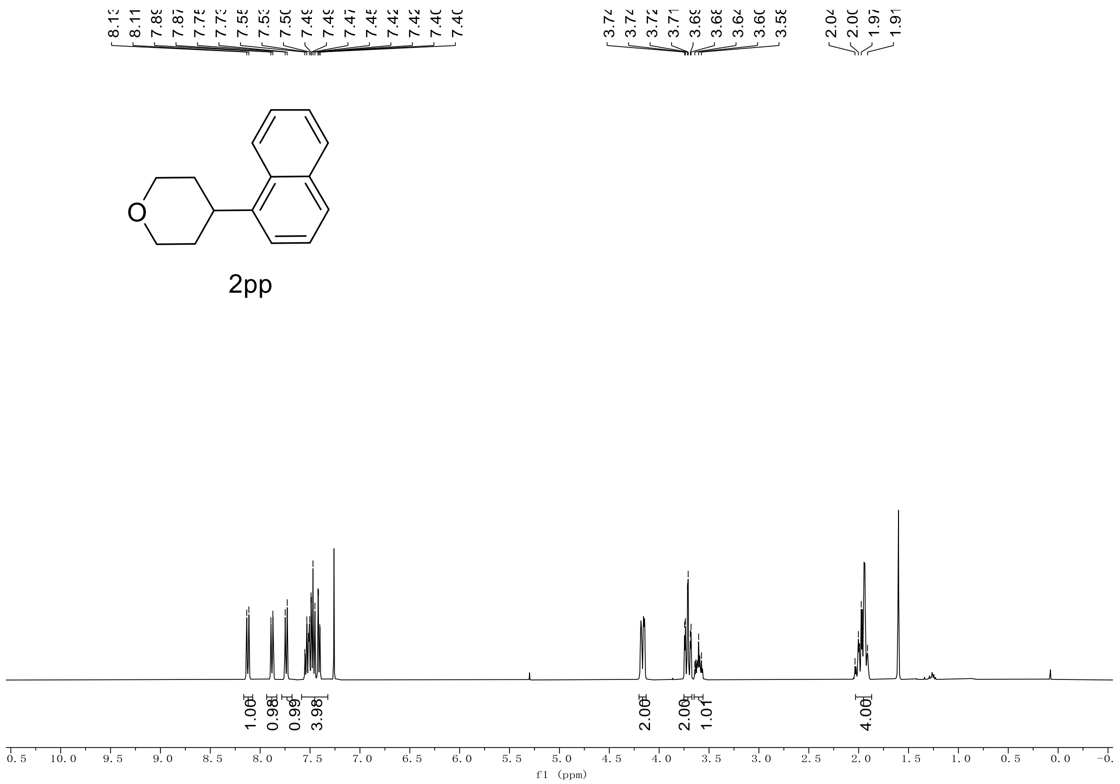

Supplementary Figure 195. ^{1}H NMR of compound **2pp** (400 MHz, $CDCl_3$)

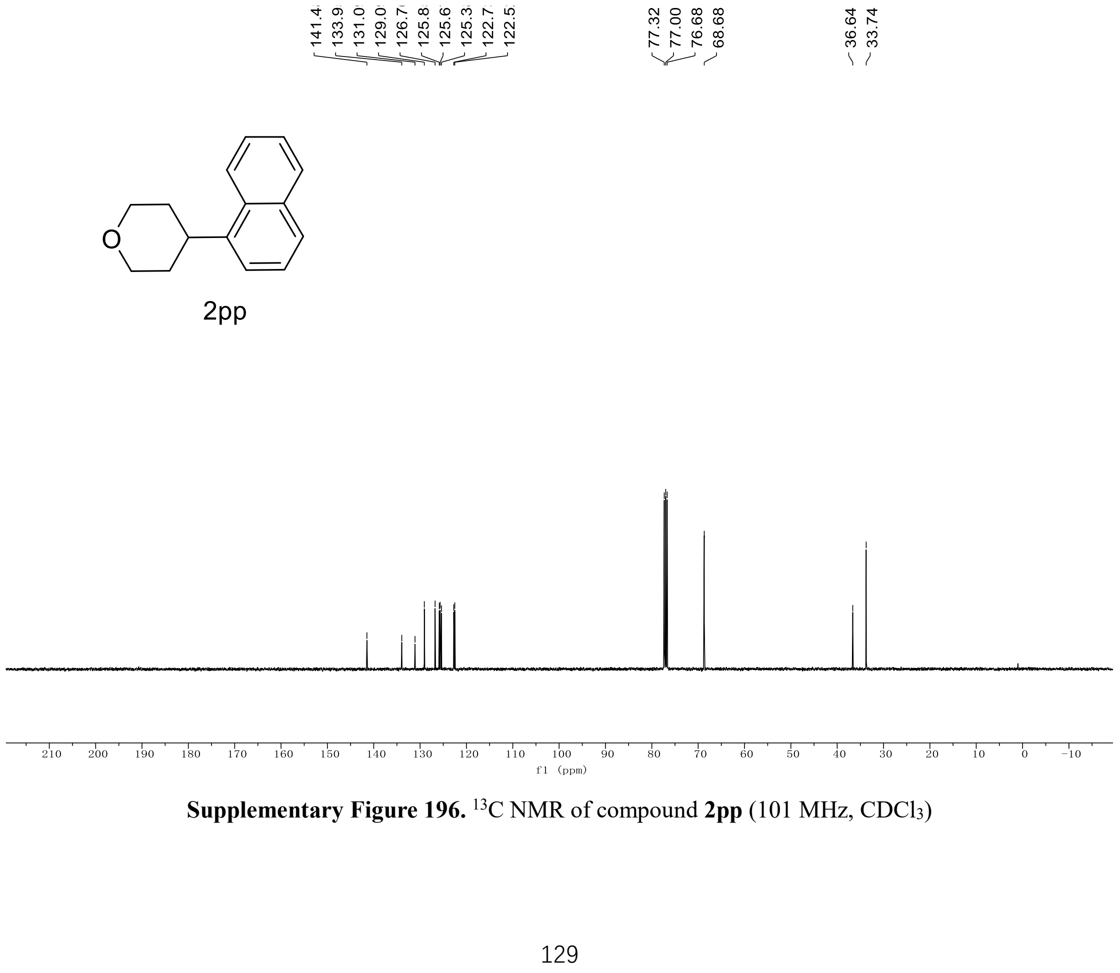

Supplementary Figure 196. ^{13}C NMR of compound **2pp** (101 MHz, $CDCl_3$)

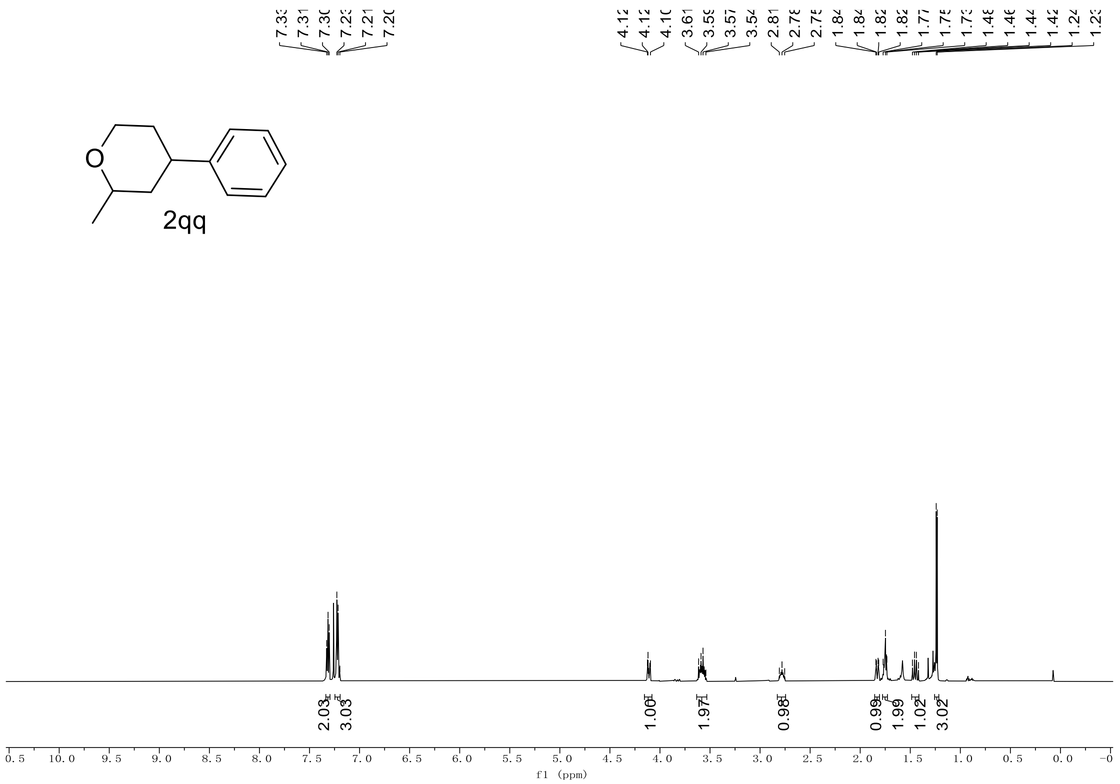

Supplementary Figure 197. ^{1}H NMR of compound **2qq** (600 MHz, $CDCl_3$)

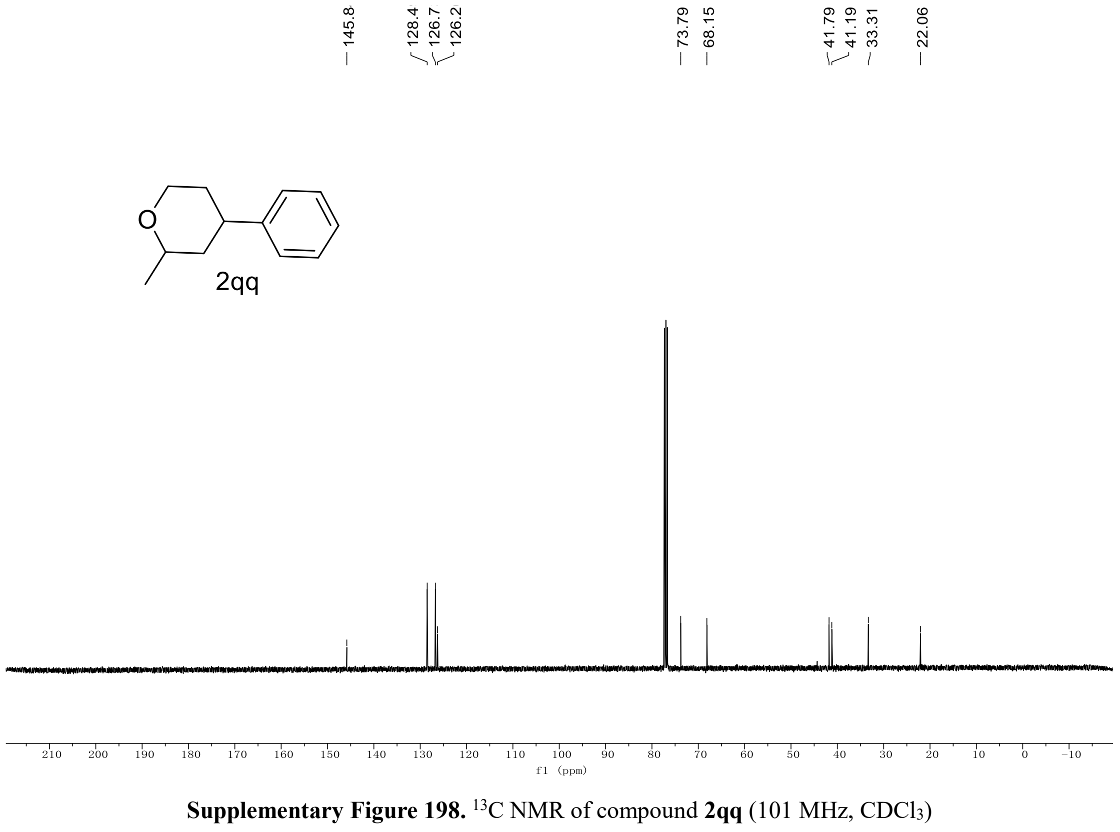

Supplementary Figure 198. ^{13}C NMR of compound **2qq** (101 MHz, $CDCl_3$)

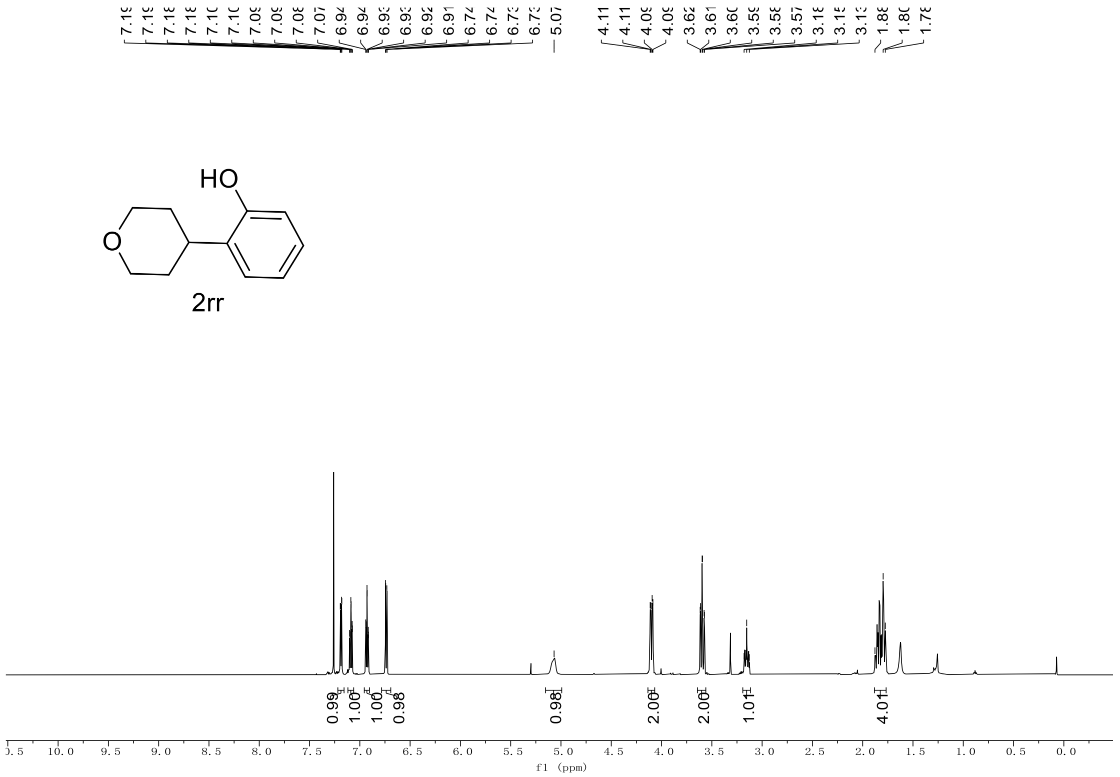

Supplementary Figure 199. ^{1}H NMR of compound **2rr** (600 MHz, $CDCl_3$)

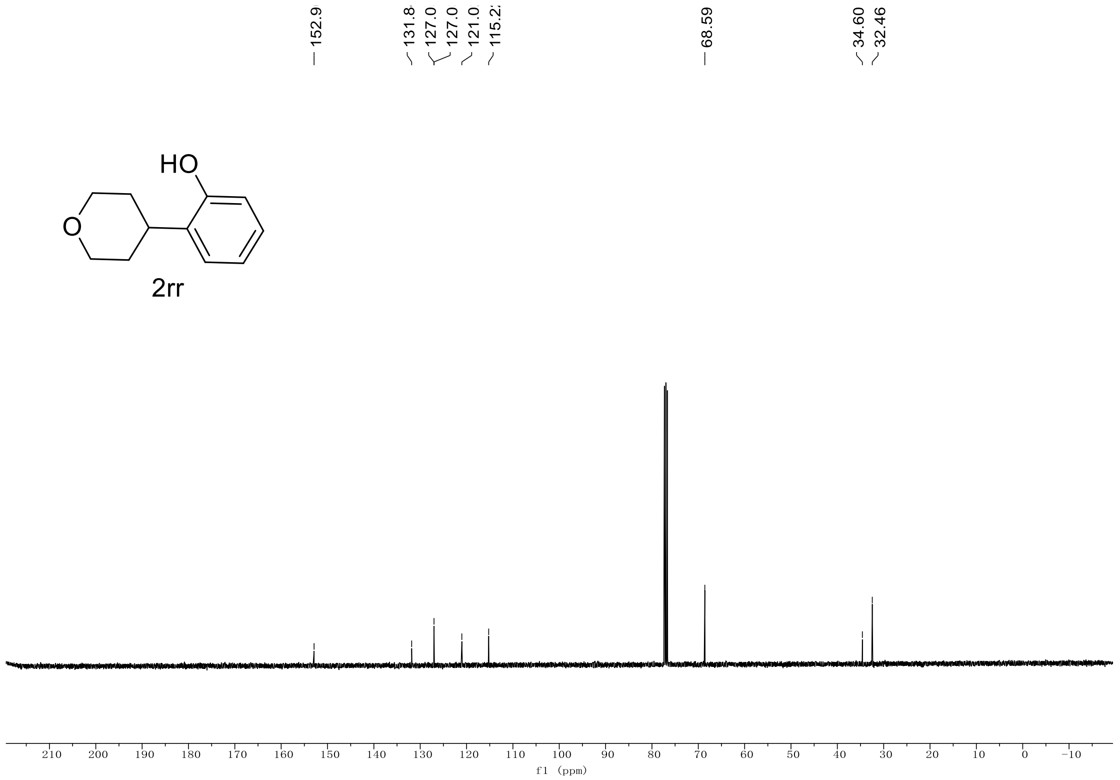

Supplementary Figure 200. ^{13}C NMR of compound **2rr** (101 MHz, $CDCl_3$)

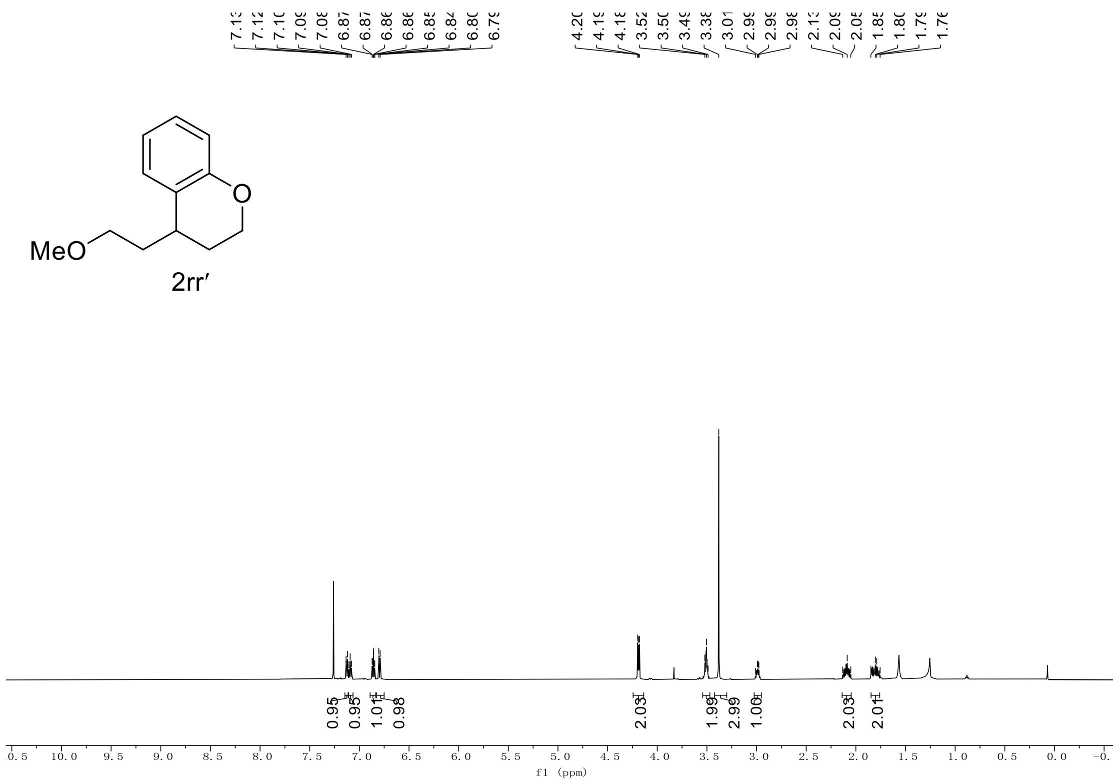

Supplementary Figure 201. ^{1}H NMR of compound **2rr'** (600 MHz, $CDCl_3$)

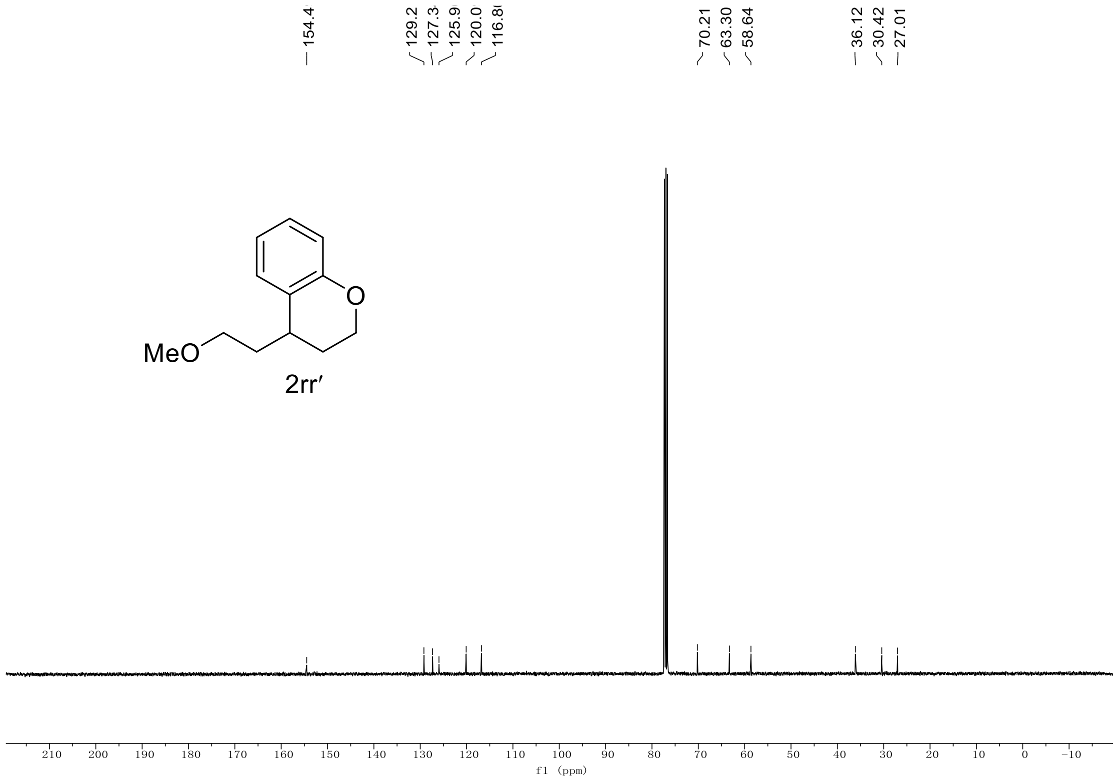

Supplementary Figure 202. ^{13}C NMR of compound **2rr'** (101 MHz, $CDCl_3$)

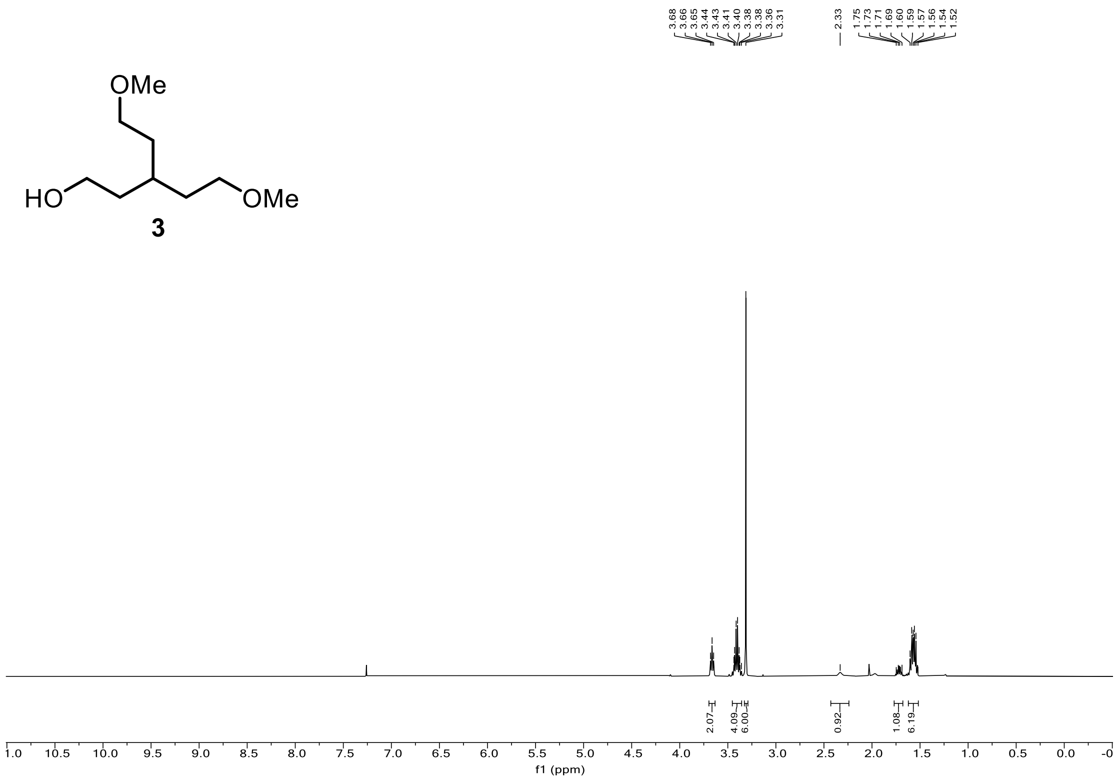

Supplementary Figure 203. ^{1}H NMR of compound **3** (400 MHz, $CDCl_3$)

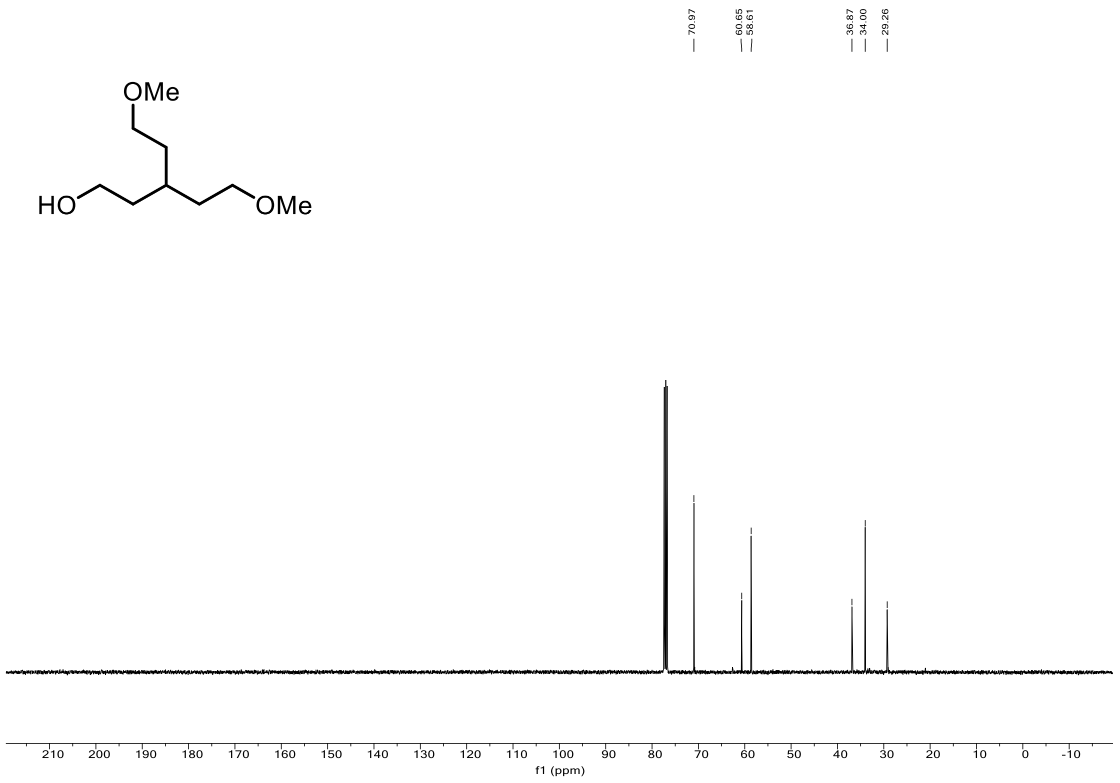

Supplementary Figure 204. ^{13}C NMR of compound **3** (101 MHz, $CDCl_3$)

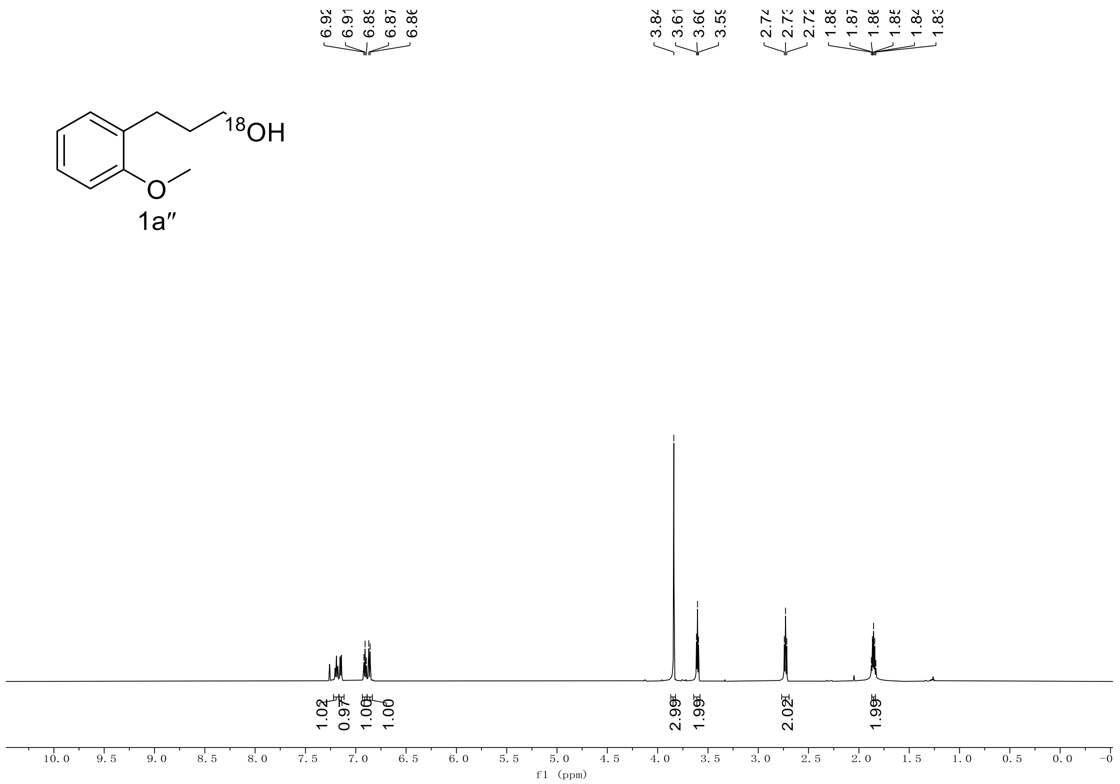

Supplementary Figure 205. ^{1}H NMR of compound **1a''** (600 MHz, $CDCl_3$)

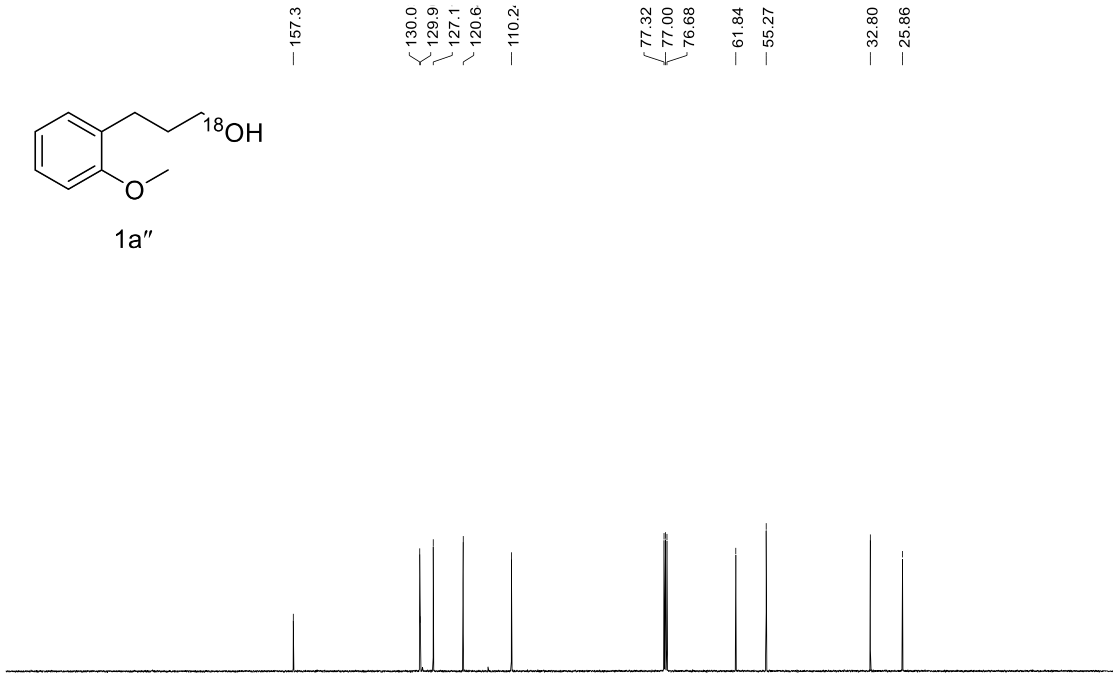

Supplementary Figure 206. ^{13}C NMR of compound **1a''** (101 MHz, $CDCl_3$)

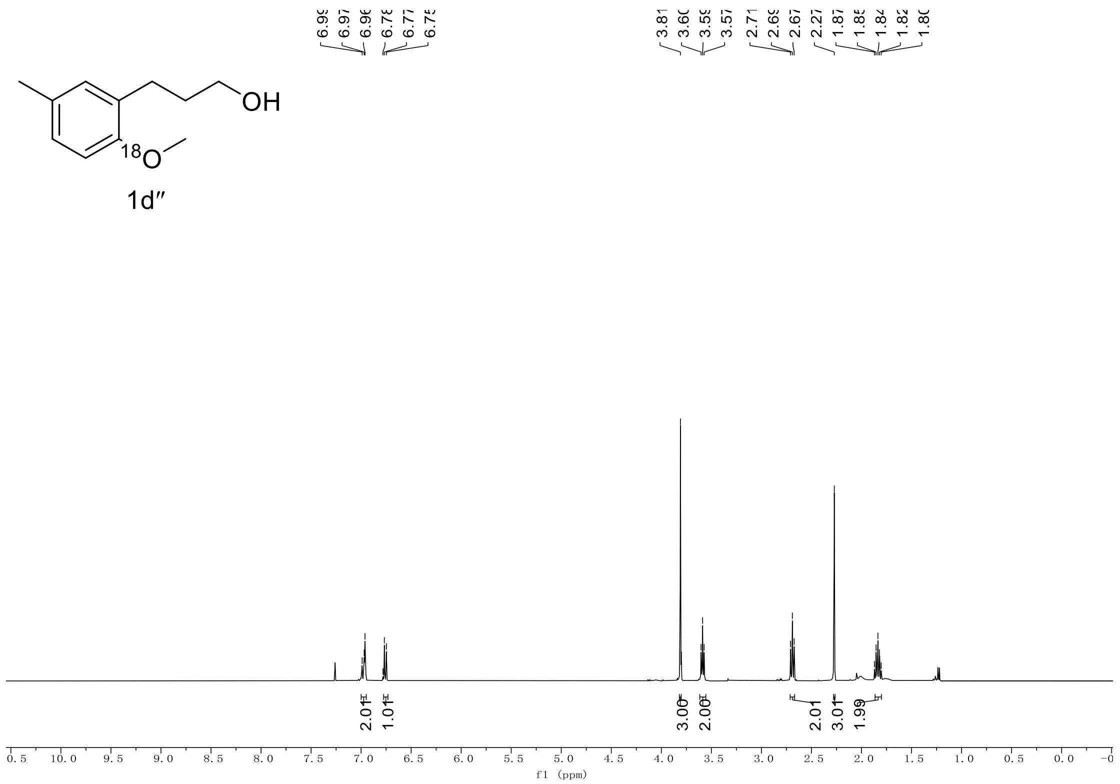

Supplementary Figure 207. ^{1}H NMR of compound **1d''** (600 MHz, $CDCl_3$)

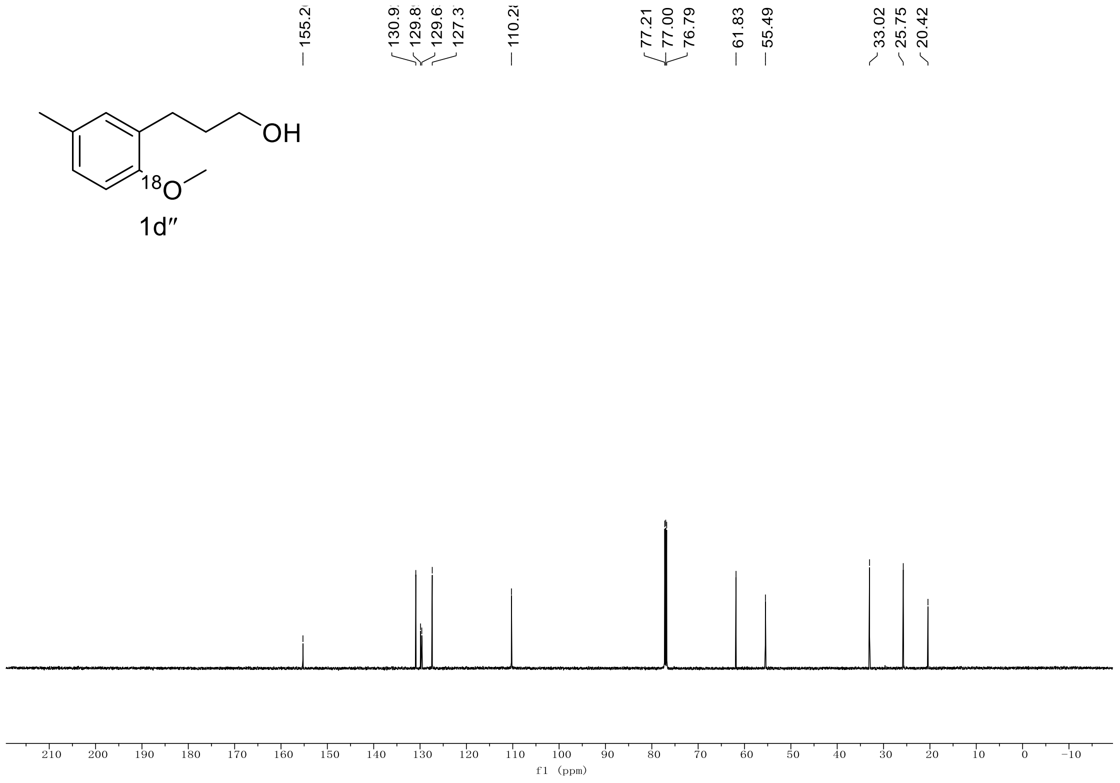

Supplementary Figure 208. ^{13}C NMR of compound **1d''** (151 MHz, $CDCl_3$)

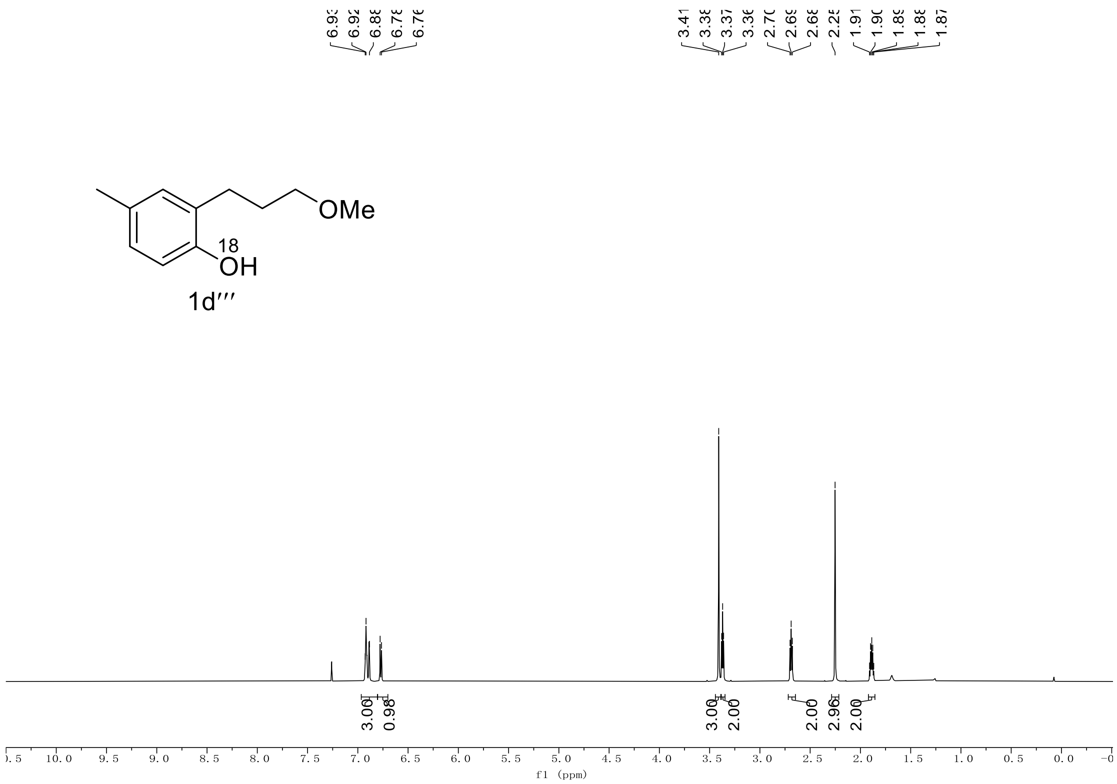

Supplementary Figure 209. ^{1}H NMR of compound **1d'''** (600 MHz, $CDCl_3$)

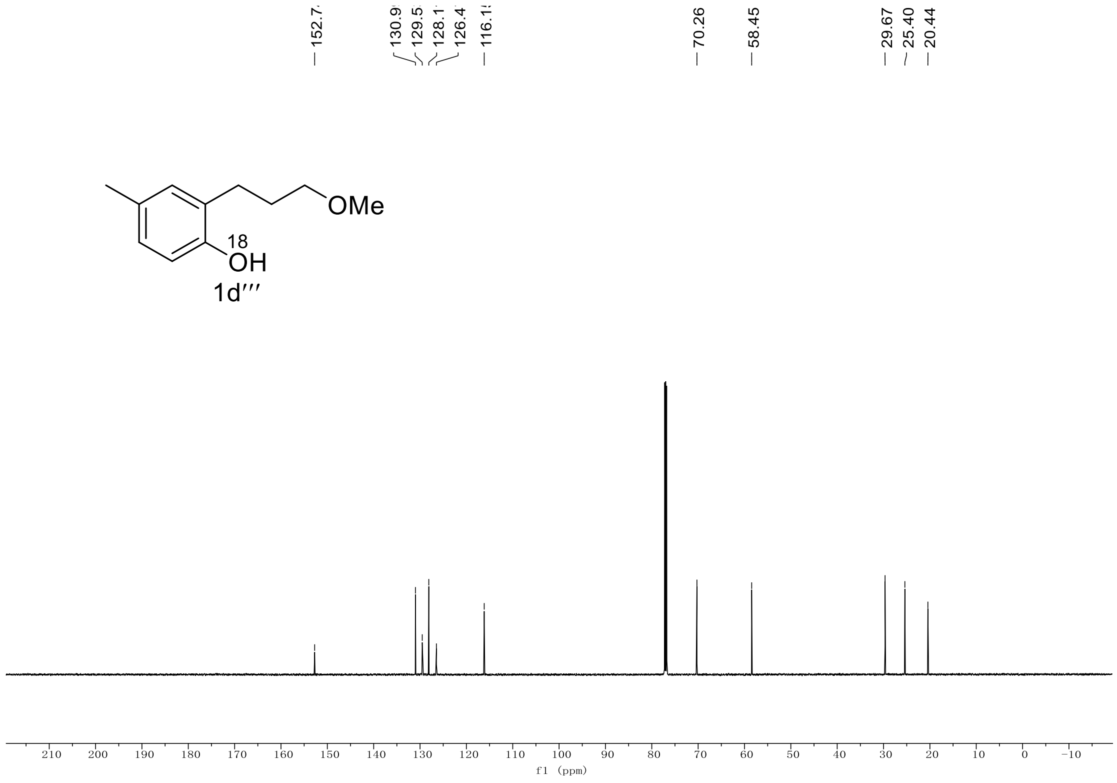

Supplementary Figure 210. ^{13}C NMR of compound **1d'''** (151 MHz, $CDCl_3$)

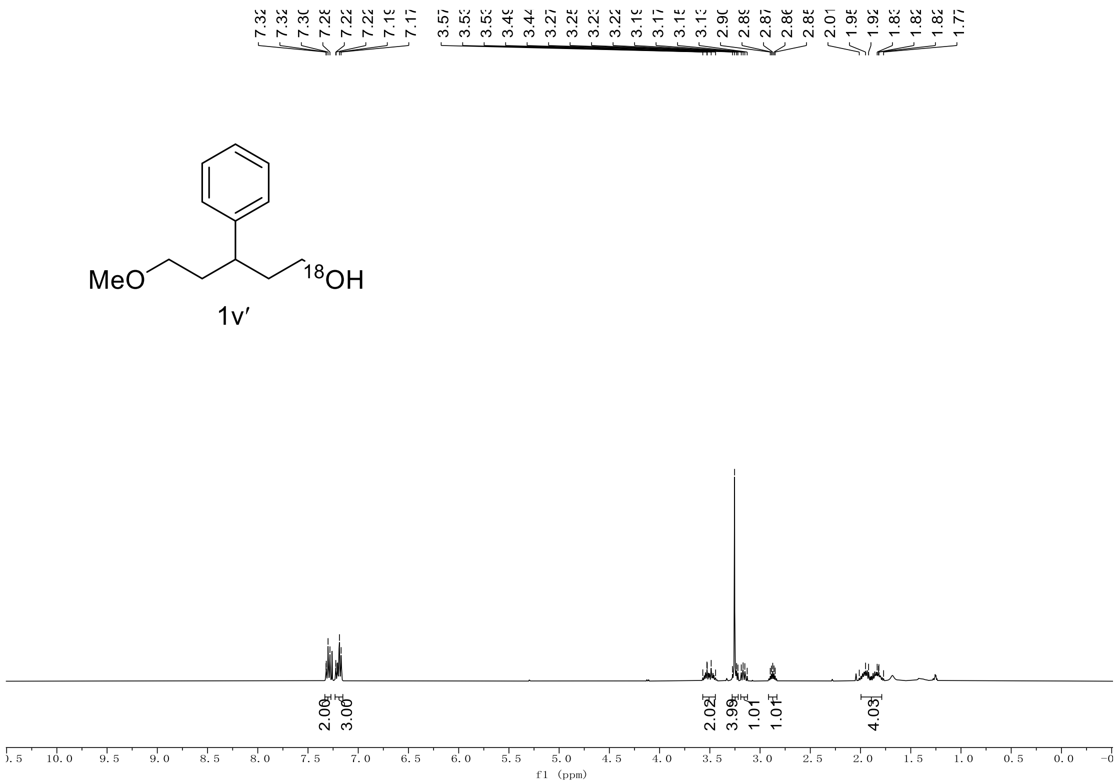

Supplementary Figure 211. ^{1}H NMR of compound **1v'** (400 MHz, $CDCl_3$)

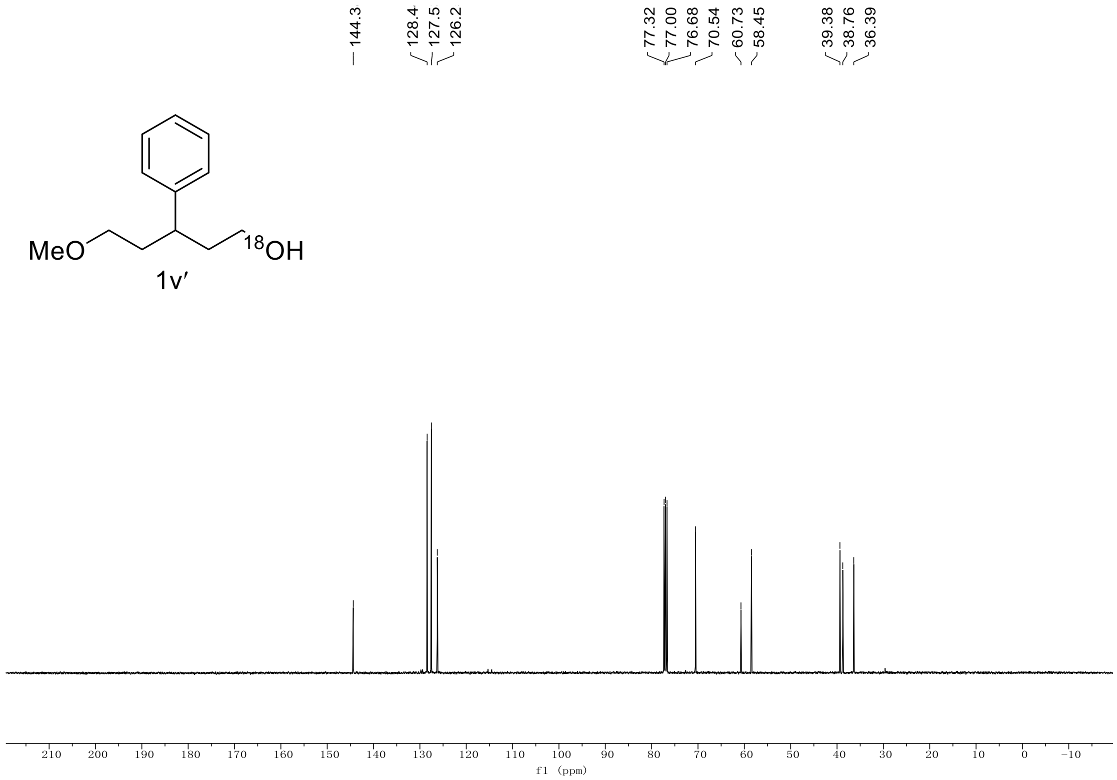

Supplementary Figure 212. ^{13}C NMR of compound **1v'** (101 MHz, $CDCl_3$)

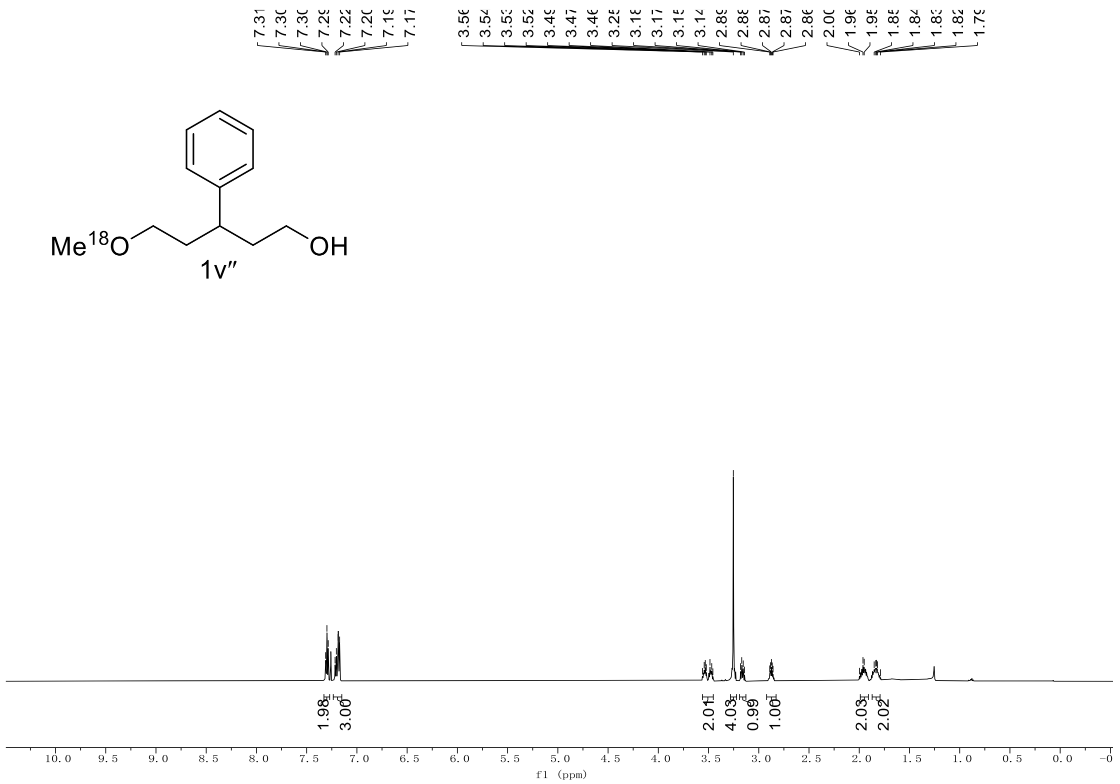

Supplementary Figure 213. ^{1}H NMR of compound **1v''** (600 MHz, $CDCl_3$)

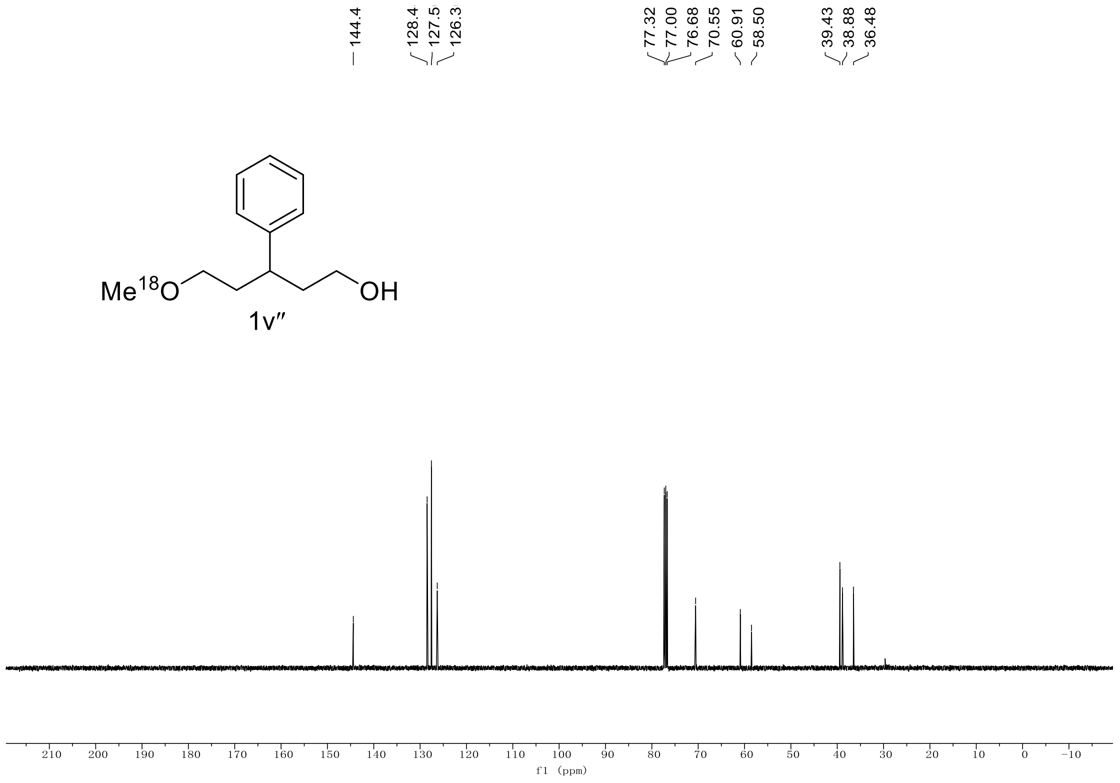

Supplementary Figure 214. ^{13}C NMR of compound **1v''** (101 MHz, $CDCl_3$)

6 Supplementary References

1. Bernasconi, M., Ramella, V., Tosatti, P. & Pfaltz, A. Iridium-Catalyzed Asymmetric Hydrogenation of 3,3-Disubstituted Allylic Alcohols in Ethereal Solvents. *Chem. Eur. J.* **20**, 2440-2444 (2014).

2. Knudsen, M. M., Kalashnyk, N., FMasini, F., Cramer, J. R., Lægsgaard, E., Besenbacher, F., Linderoth, T. R. & Gothelf, K. G. Controlling Chiral Organization of Molecular Rods on Au(Ш) by Molecular Design. *J. Am. Chem. Soc*. **133**, 4896-4905 (2011).

3. Gallagher, B. D., Taft, B. R. & Lipshutz, B. H. Asymmetric Conjugate Reductions of Coumarins. A New Route to Tolterodine and Related Coumarin Derivatives. *Org. Lett.* **11**, 5374-5377 (2009).

4. Fujita, T., Takahashi, I., Hayashi, M., Wang, J., Fuchibe, K. & Ichikawa, J. Facile Synthesis of Polycyclic Aromatic Hydrocarbons: BrØsted Acid Catalyzed Dehydrative Cycloaromatization of Carbonyl Compounds in 1,1,1,3,3,3-Hexafluoropropan-2-ol. *Eur. J. Org. Chem*. **2017**, 262-265 (2016).

5. Biberger, T., Makai, S., Lian, Z. & Morandi, B. Iron-Catalyzed Ring-Closing C-O/C-O Metathesis of Aliphatic Ethers. *Angew. Chem. Int. Ed*. **57**, 6940-6944 (2018)

6. Zhu, Y., Huang, K., Pan, J., Qiu, X., Luo, X., Qin, Q., Wei, J., Wen, X., Zhang, L. & Jiao, N. Silver-catalyzed remote Csp3-H functionalization of aliphatic alcohols. *Nat Commun*. **9**, 2625-2631 (2018).

7. Liang, H. & Ciufolini, M. A. Tandem Phenolic Oxidative Amidation-Intramolecular Diels-Alder Reaction: An Approach to the Himandrine Core *Org. Lett.***12**, 1760-1763 (2010).

8. Bang, H. B., Han, S. Y., Choi, D. H. Hwang, J. W. & Jun, J.-G. High yield total syntheses of XH-14 derivatives using Sonogashira coupling reaction. *Arkivoc Archive for Organic Chemistry*. ii, 112-125 (2009).

9. Atsushi Kaga, A., Hayashi, H., Hakamata, H. Oi, M., Uchiyama, M. Takita, R. & Chiba, S. Nucleophilic Amination of Methoxy Arenes Promoted by a Sodium Hydride/Iodide Composite. *Angew. Chem. Int. Ed*. **56**, 11807-11811 (2017)

10. Ramachandran, P. V., Drolet, M. P. & Kulkarni, A. S. A non-dissociative open-flask hydroboration with ammonia borane: ready synthesis of ammonia–trialkylboranes and aminodialkylboranes. *Chem. Commun.* **52**, 11897-11900 (2016)

11. Kulkarni, B. A., Sharma, A., Gamre, S. & Chattopadhyay, S. Synthesis of the Marine Compound (2R,5Z,9Z)-2-Methoxyhexacosa-5,9- dienoic Acid via a Lipase-Catalyzed Resolution and a Novel O-Alkylation Protocol. *Synthesis*. **4**, 595–599 (2004)

12. Tao, L., Yang, W. & Zhao, W. Synthesis of Carboxylic Acids, Esters, and Amides from 1,1-Dibromoalkenes via Oxidation of Alkynyl Boronate Intermediates. *ChemistrySelect*. **6**, 8532-8536 (2021)

13. Wang, H., Zhao, Y., Zhang, F., Wu, Y., Li, R., Xiang, J., Wang, Z., Han, B. & Liu, Z. Hydrogen-Bonding Catalyzed Ring-Closing C-O/C-O Metathesis of Aliphatic Ethers over Ionic Liquid under Metal-Free Conditions *Angew. Chem. Int. Ed*. **59**, 11850-11855 (2020).

14. Hou, T.; Zhang, J.; Wang, C. & Jun Luo, J. A facile method to construct a 2,4,9-triazaadamantane skeleton and synthesize nitramine derivatives. *Org. Chem. Front.* **4**, 1819–1823 (2017)

15. Han, Y., Zheng, B. & Penga, Y. Construction of Chiral 2-Substituted Octahydroindoles from Cyclic Ketones and Nitroolefins Bearing only One a-Substituent. *Adv. Synth. Catal.* **357**, 1136-1142 (2015)

16. Liu, H., Liu, J., Cheng, X., Jia, X., Lei Yu, L. & Qing Xu, Q. DMSO-Triggered Complete Oxygen Transfer Leading to Accelerated Aqueous Hydrolysis of Organohalides under Mild Conditions. ChemSusChem. **12**, 2994-2998 (2019); Bao, Y.-S., Chen, C.-Y. & Huang, Z.-Z. Transesterification for Synthesis of Carboxylates Using Aldehydes as Acyl Donors via C−H and C−O Bond Activations. *J. Org. Chem*. **77**, 8344−8349 (2012)

17. Tanii, S., Arisawa, M. & Yamaguchi, M. Acid-Catalyzed Synthesis of Condensed Polycyclic Diaryl Ethers from Arenols. *Chem. Commun*. **55**, 14078-14080 (2019)

18. Han, C., Fu, Z., Guo, S., Fang, X., Lin, A. & Yao, H. Palladium-Catalyzed Remote 1,n-Arylamination of Unactivated Terminal Alkenes. *ACS Catal*. **9**, 4196−4202 (2019)

19. Gaussian 16, Revision C.01, Frisch, M. J., Trucks, G. W., Schlegel, H. B., Scuseria, G. E., Robb, M. A., Cheeseman, J. R., Scalmani, G., Barone, V., Petersson, G. A., Nakatsuji, H., Li, X., Caricato, M., Marenich, A. V., Bloino, J., Janesko, B. G., Gomperts, R., Mennucci, B., Hratchian, H. P., Ortiz, J. V., Izmaylov, A. F., Sonnenberg, J. L., Williams-Young, D., Ding, F.; Lipparini, F., Egidi, F., Goings, J., Peng, B., Petrone, A.; Henderson, T., Ranasinghe, D., Zakrzewski, V. G., Gao, J.; Rega, N., Zheng, G.; Liang, W., Hada, M., Ehara, M., Toyota, K., Fukuda, R., Hasegawa, J., Ishida, M., Nakajima, T., Honda, Y., Kitao, O., Nakai, H., Vreven, T., Throssell, K., Montgomery, J. A., Jr., Peralta, J. E., Ogliaro, F., Bearpark, M. J., Heyd, J. J., Brothers, E. N., Kudin, K. N., Staroverov, V. N., Keith, T. A., Kobayashi, R., Normand, J., Raghavachari, K., Rendell, A. P., Burant, J. C., Iyengar, S. S., Tomasi, J., Cossi, M., Millam, J. M., Klene, M.; Adamo, C, Cammi, R., Ochterski, J. W., Martin, R. L., Morokuma, K., Farkas, O., Foresman, J. B., Fox, D. J. Gaussian, Inc., Wallingford CT, 2016.

20. Becke, A. D. Density-functional thermochemistry. III. The role of exact exchange. *J. Chem. Phys.* **98**, 5648−5652 (1993).

21. Grimme, S., Antony, J., Ehrlich, S. & Krieg, H. A consistent and accurate *ab initio* parametrization of density functional dispersion correction (DFT-D) for the 94 elements H-Pu. *J. Chem. Phys.* **132**, 154104 (2010).

22. Andrae, D., U. Häußermann, Dolg, M., Stoll, H. & Preuß, H. Energy-adjusted *ab initio* pseudopotentials for the second and third row transition elements. *Theor. Chim. Acta.* **77**, 123−141 (1990).

23. Marenich, A. V., Cramer, C. J. & Truhlar, D. G. Universal Solvation Model Based on Solute Electron Density and on a Continuum Model of the Solvent Defined by the Bulk Dielectric Constant and Atomic Surface Tensions. *J. Phys. Chem. B.* **113**, 6378−6396 (2009).
