# Supplementary material for: Ring-closing C–O/C–O metathesis of ethers with primary aliphatic alcohols
Source: Nat Commun. 2023 Apr 5;14:1883. doi: 10.1038/s41467-023-37538-1 (PMC10076310; doi:10.1038/s41467-023-37538-1)
Supplement: Supplementary file 3 — Description of Additional Supplementary Files [file 41467_2023_37538_MOESM3_ESM.docx]

**Description of Additional Supplementary Files**

**File Name: Supplementary Data 1**

**Description:** Cartesian Coordinates of the Computed Structures for DFT calculations.
