# Supplementary material for: Ring-closing C–O/C–O metathesis of ethers with primary aliphatic alcohols
Source: Nat Commun. 2023 Apr 5;14:1883. doi: 10.1038/s41467-023-37538-1 (PMC10076310; doi:10.1038/s41467-023-37538-1)
Supplement: Supplementary file 4 — Supplementary Data 1 [file 41467_2023_37538_MOESM4_ESM.pdf]

Supplementary Data 1

A Mechanism Reversal of Transetherification: Ring-closing C–O/C–O Metathesis of Ethers with Primary Aliphatic Alcohols

Hongmei Liu,[1] Qing Huang,[1] Rong-zhen Liao,[1] Man Li,[1]* & Youwei Xie[1]*

[1]Hubei Key Laboratory of Bioinorganic Chemistry and Materia Medica; Key Laboratory of Material Chemistry for Energy Conversion and Storage, Ministry of Education; Hubei Key Laboratory of Materials Chemistry and Service Failure; School of Chemistry and Chemical .Engineering, Huazhong University of Science and Technology, 1037 Luoyu Road, Wuhan, 430074, China

*e-mail: manli_hx@hust.edu.cn, xieyw@hust.edu.cn

Table of Contents

1. Cartesian Coordinates of the Computed Structures……………………………………...2

5.1 Cartesian coordinates for all optimized structures

Re_2O_7

Re	0.00000000	0.00000000	1.88983500
O	0.00000000	1.59814700	2.47923800
O	-1.38403600	-0.79907400	2.47923800
O	1.38403600	-0.79907400	2.47923800
O	0.00000000	0.00000000	0.00000000
Re	0.00000000	0.00000000	-1.88983500
O	-1.38403600	-0.79907400	-2.47923800
O	1.38403600	-0.79907400	-2.47923800
O	0.00000000	1.59814700	-2.47923800

$HReO_4$

Re	0.03388800	-0.00228300	-0.00000200
O	0.54883900	0.85723500	-1.38215500
O	0.55027000	0.85419100	1.38350400
O	-1.83272900	-0.19532700	0.00057600
O	0.72380300	-1.56244600	-0.00199600
H	-2.46307200	0.54200200	0.00071000

$(HFIP)_2$

O	-1.73122000	0.31883500	1.72268300
H	-1.83607800	-0.37059200	2.39573100
C	-2.59106400	0.05252400	0.65120900
H	-3.65502700	0.04810500	0.94986400
C	-2.31273900	-1.33409100	0.03864400
C	-2.42880700	1.20666400	-0.34722300
F	-3.12168700	0.95658300	-1.46270700
F	-1.14294400	1.38851000	-0.67859300
F	-2.88275900	2.33827100	0.19131600
F	-1.16668900	-1.37893400	-0.64588400
F	-3.30518300	-1.70433200	-0.77117200
F	-2.23247400	-2.22449000	1.04425300
H	0.12033100	0.15777400	1.31007500
O	0.93903500	-0.36031300	1.22834400
C	1.55871200	-0.05289100	0.02402600
H	0.86416900	-0.00920800	-0.83229600
C	2.22829500	1.32959100	0.09361700
C	2.53734200	-1.18863200	-0.28770800
F	1.32041100	2.22194200	0.53273900
F	2.64729100	1.73283900	-1.11327900
F	3.26846000	1.35885100	0.93090000
F	1.85388600	-2.32122300	-0.50062600
F	3.39793000	-1.40437200	0.70877200
F	3.24083200	-0.91622800	-1.39871600

$[ReO_4(HFIP)_2]^{\ominus}$

Re	-1.97779700	-0.09920700	0.03606000
O	-0.95108600	-0.21064200	1.43526700
O	-2.63780700	1.49698300	-0.08030000
O	-1.01255500	-0.46006200	-1.38903500
O	-3.27286900	-1.24148200	0.16926200
O	1.61587000	-0.43789700	-1.54301200
H	0.62672100	-0.45299700	-1.62402800
C	1.91189500	-0.18890900	-0.21319700
H	1.12117400	-0.49839800	0.49378900
C	3.15745100	-0.99750700	0.15392600
C	2.09341900	1.31912400	0.02167700
F	3.54899200	-0.76768000	1.42292700
F	2.89982800	-2.30992500	0.04738900
F	4.20438100	-0.72691900	-0.64176200
F	2.30205700	1.61037700	1.31772000
F	0.99056100	1.97099800	-0.37115000
F	3.12949400	1.82542600	-0.67358900

$[ReO_4(HFIP)_2]^{\ominus}$

Re	-0.01210800	-1.49101400	-0.44189800
O	1.40146500	-1.60003400	-1.46542700
O	0.01878100	0.05274700	0.39200900
O	-1.44378700	-1.56701200	-1.41145600
O	-0.01707900	-2.78991200	0.69055400
O	-2.08425500	1.63383200	0.84242800
H	-1.23670200	1.13252400	0.78463100
C	-3.02844500	0.94161700	0.10109200
H	-2.60904400	0.35812000	-0.73778800
C	-3.97735500	1.96626800	-0.52473100
C	-3.75845300	-0.08515300	0.98279900
F	-4.61059100	-0.84434500	0.27479100
F	-4.45991000	0.49587000	1.97123100
F	-2.85982700	-0.90206700	1.55350000
F	-4.54666600	2.76690300	0.38728100
F	-4.96872800	1.36867100	-1.21344300
F	-3.30438900	2.74491600	-1.38477900
O	3.67175500	-0.23483000	-1.16761300
H	2.91473100	-0.83455000	-1.36864000
C	3.25196600	0.64664400	-0.18404000
H	2.16550900	0.84609100	-0.18660800
C	3.93421600	1.99302900	-0.43711400

C	3.55466200	0.07889000	1.21258400
F	3.06768600	0.85776500	2.19130100
F	4.87308600	-0.06850700	1.43006000
F	2.98985000	-1.13270000	1.33352600
F	5.27040500	1.89234500	-0.48564900
F	3.63037800	2.89110900	0.51862400
F	3.52304300	2.49868000	-1.60941900

1a

O	-2.88099400	0.52510900	0.39599400
C	-2.30071200	-0.59524100	1.02614900
H	-1.41990600	-0.30668700	1.63783600
C	-1.89801600	-1.69198200	0.03608800
H	-1.46438600	-2.53880100	0.59789100
H	-2.80836500	-2.06413000	-0.46457800
C	-0.90188600	-1.23133200	-1.04914500
H	-0.72275700	-2.07181300	-1.73876500
H	-1.37032600	-0.43294700	-1.64402000
C	0.42863200	-0.75838900	-0.50595000
C	1.45299900	-1.67285300	-0.23516000
C	0.67343500	0.60462600	-0.22202300
C	2.68436600	-1.27447100	0.29552800
H	1.27534100	-2.73026200	-0.45326600
C	1.90421000	1.01817600	0.30572700
C	2.90546800	0.07555900	0.56391300
H	3.46361000	-2.01423700	0.49255600
H	2.09359900	2.06931000	0.52144600
H	3.86110300	0.40912800	0.97620600
H	-2.15732700	0.98064700	-0.06086400
O	-0.35111200	1.47627300	-0.49338700
C	-0.17906700	2.85927600	-0.25261500
H	0.64414000	3.27651200	-0.85857100
H	-1.12036500	3.34476700	-0.54346500
H	0.01615600	3.06703100	0.81432600
H	-3.05023600	-0.99979200	1.72734300

TS1

Re	2.01687000	-0.68423200	0.06346500
O	2.11917600	-0.77987800	1.76229500
O	3.40647800	0.20163200	-0.38411300
O	2.27215800	-2.24025600	-0.58214500
O	0.84631800	0.40054000	-1.16330800
C	1.32231700	1.29821700	-2.21232000
H	2.37813600	1.52445900	-2.00102400

C	0.48339900	2.56310700	-2.28417500
H	0.77040300	3.07647900	-3.21857700
H	-0.58050500	2.29915300	-2.38946000
C	0.66508800	3.53171000	-1.09973100
H	1.71224700	3.87476200	-1.06922100
H	0.03703600	4.41706800	-1.28870200
C	0.30061900	2.93115200	0.23705900
C	1.27363800	2.58941500	1.18060200
C	-1.05004500	2.63232900	0.52736400
C	0.94149900	1.93608000	2.37448500
H	2.32149600	2.81468900	0.96261000
C	-1.39237800	1.97639000	1.71851000
C	-0.39178200	1.62033900	2.63043700
H	1.72392800	1.64729800	3.07697500
H	-2.42677400	1.71557400	1.93484600
H	-0.66628000	1.08628400	3.54275000
O	-0.19925400	-1.37476500	0.56064300
H	-0.45869900	0.29641000	-1.30399100
Re	-1.81652000	-1.20128000	-0.08073200
O	-2.95843400	-0.70963200	1.09254900
O	-2.35292700	-2.65294800	-0.79527500
O	-1.56379100	0.06814200	-1.34323000
O	-1.94997300	3.00012700	-0.42307700
C	-3.31960600	2.70644400	-0.23555800
H	-3.72910900	3.22670500	0.64869400
H	-3.84025200	3.06242100	-1.13420700
H	-3.49423400	1.62356100	-0.12284800
H	1.27189900	0.73374300	-3.15752800

Int1

Re	1.51664700	-0.04795100	-0.18637400
O	1.00589800	-1.42529700	-1.06289800
O	1.79415200	-0.44341600	1.45421700
O	2.99074100	0.51425900	-0.84974600
O	0.24058700	1.28154800	-0.35011400
C	-0.16948000	2.45557300	0.36947200
H	0.16933300	2.37182200	1.41766000
C	-1.67992100	2.60668600	0.27859700
H	-1.92847100	3.65081700	0.53651600
H	-1.97458100	2.45307600	-0.77040900
C	-2.47840300	1.65886400	1.19462200
H	-2.30905800	1.94891500	2.24491000
H	-3.54973800	1.81575600	0.98724400
C	-2.14466100	0.19312500	1.03748500

C	-1.45659600	-0.51140900	2.02787800
C	-2.49029100	-0.49061900	-0.15216300
C	-1.10023000	-1.85663300	1.86288700
H	-1.17407100	0.01063400	2.94628500
C	-2.13909200	-1.83406900	-0.32686400
C	-1.44238000	-2.51024700	0.68242000
H	-0.53967100	-2.37378200	2.64344100
H	-2.38128500	-2.35916400	-1.24997800
H	-1.15266400	-3.55138100	0.52429600
O	-3.15460800	0.25164900	-1.08169500
C	-3.48581200	-0.32948300	-2.32406400
H	-4.17104600	-1.18856800	-2.20644800
H	-3.99126600	0.45030200	-2.90986200
H	-2.58620700	-0.66483400	-2.87130600
H	0.34722300	3.31203900	-0.09401800

TS2

Re	-0.91245200	-1.92925600	-0.56021800
O	-0.23585200	-1.77225900	1.03622700
O	0.14280700	-2.90723500	-1.50039200
O	-2.49411400	-2.59860200	-0.48598000
O	-1.00693100	-0.30145100	-1.28295600
C	0.03393600	1.39587400	-0.43227100
H	-0.90552700	1.54732000	0.09129400
C	0.26946600	2.06971600	-1.73926200
H	0.97506100	1.46858300	-2.32640200
H	-0.69375500	2.10165600	-2.26897000
C	0.80705600	3.52430000	-1.61525700
H	0.92091500	3.92903400	-2.63066300
H	0.04745300	4.13350300	-1.09799300
C	2.11222100	3.58697600	-0.87455500
C	3.31406400	4.03224100	-1.43013600
C	2.13417800	3.10963200	0.44606000
C	4.49713600	4.01491000	-0.68332500
H	3.32083000	4.39694000	-2.46071900
C	3.30152100	3.06762700	1.20543400
C	4.48712200	3.53357500	0.62695400
H	5.42875500	4.36938500	-1.12949100
H	3.30841700	2.66671300	2.21792200
H	5.41098400	3.50397500	1.20872700
O	0.89887100	2.66493100	0.89206900
C	0.76763800	2.14678400	2.22591300
H	0.99936600	2.94632300	2.94463600
H	-0.27950500	1.83908500	2.33196000

H	1.43156600	1.28228200	2.36887700
H	0.72990100	0.65617900	-0.04800100
O	2.14103100	-0.62692600	1.13947700
H	1.31215100	-1.12673800	1.33152300
C	2.90948800	-1.38698900	0.25850200
H	2.30194900	-1.98924200	-0.44333800
C	3.77749500	-2.38771200	1.03827700
C	3.72252500	-0.41847900	-0.60535800
F	4.48084100	-1.06972600	-1.48971500
F	4.50948800	0.37680500	0.12362500
F	2.87278800	0.37341300	-1.29704400
F	4.65165600	-1.77495600	1.84403100
F	4.45826700	-3.18950100	0.21060400
F	2.97631800	-3.14938100	1.79606700
O	-3.16480700	1.24246400	-0.84832400
H	-2.48563500	0.63505700	-1.20844000
C	-3.97139600	0.53170400	0.03808000
H	-3.88985300	-0.56423300	-0.07149600
C	-5.43638400	0.88788900	-0.25060600
C	-3.55336600	0.84450300	1.48350300
F	-4.26255600	0.16071900	2.37994600
F	-3.63521800	2.14587100	1.77270900
F	-2.24825400	0.48970200	1.64185600
F	-5.66345700	2.19992900	-0.12516800
F	-6.26276100	0.23972800	0.58362300
F	-5.74193300	0.52802800	-1.50117500

Int2

C	2.11717700	0.02696700	-0.78358700
H	1.76929900	0.23816300	-1.80387600
C	2.10888200	1.23423600	0.12566000
H	2.41899100	0.93401400	1.14002900
H	2.87900500	1.93560400	-0.23169300
C	0.72483800	1.89529600	0.13562500
H	0.62094400	2.55007200	1.01425800
H	0.62333100	2.55337100	-0.74479600
C	-0.41770700	0.90215100	0.09693700
C	-1.75444100	1.32417800	0.21768400
C	-0.23907200	-0.45936600	-0.14487300
C	-2.81606200	0.42837400	0.09960500
H	-1.95244500	2.38286700	0.40427100
C	-1.26719200	-1.38387100	-0.29934200
C	-2.57771500	-0.92665800	-0.16640600
H	-3.84138000	0.78923500	0.20387700

H	-1.04977300	-2.42908400	-0.52718500
H	-3.40880800	-1.62525700	-0.28008900
O	1.12150000	-0.97718300	-0.27286500
C	1.59184000	-1.84799400	0.82552000
H	1.76953300	-1.23628100	1.71922200
H	0.80187700	-2.58374900	1.00177900
H	2.50518300	-2.33498600	0.46544600
H	3.06896700	-0.51638400	-0.81924900

TS3

Re	-0.72297200	-2.06818900	-0.67943700
O	-0.63439300	-0.91410300	0.62370700
O	0.27789900	-3.42609000	-0.29046100
O	-2.35199700	-2.54692900	-0.93184700
O	-0.07047500	-1.23980300	-2.09930200
C	1.93464100	3.10478700	-2.68567000
H	1.73668000	4.18658600	-2.76690200
C	3.34747700	2.82182200	-2.20510400
H	3.50305800	1.73413100	-2.14902800
H	4.06225700	3.21032800	-2.94726600
C	3.57520000	3.46005100	-0.83168800
H	4.48582600	3.05374300	-0.36785700
H	3.74027800	4.54632400	-0.95040400
C	2.39610400	3.25171400	0.09331400
C	2.49951100	3.44463100	1.47969000
C	1.13778300	2.88629600	-0.40008000
C	1.40303900	3.27488700	2.32542900
H	3.47319200	3.71511000	1.89720500
C	0.01721600	2.74535400	0.42018200
C	0.15476100	2.93028600	1.79442900
H	1.52276500	3.40803100	3.40275800
H	-0.94963800	2.48140100	-0.00525800
H	-0.71928000	2.79053500	2.43339900
O	0.95445300	2.57160400	-1.76021300
C	0.50871300	0.70986100	-2.00929700
H	0.09476700	0.70687100	-1.00762700
H	-0.14180500	0.92042700	-2.85289400
H	1.53295500	0.38249100	-2.16880300
H	1.70696800	2.63425900	-3.65196400
O	2.88611700	-2.68302500	0.20429500
H	2.00220300	-3.10073400	0.14653600
C	2.76644000	-1.36662300	0.60752100
H	1.82665600	-0.87091700	0.30654200
C	3.88430500	-0.58387600	-0.08575200

C	2.78834400	-1.23106100	2.14108400
F	2.47374400	0.02010500	2.51664600
F	3.98188400	-1.53439300	2.66349600
F	1.87733300	-2.06179400	2.66164300
F	5.07957000	-1.13855700	0.06027600
F	3.95252400	0.68670300	0.35774800
F	3.61727200	-0.51689700	-1.41449900
O	-2.41571600	0.96900900	1.31129500
H	-1.68843500	0.31514000	1.24262100
C	-3.50351500	0.48075800	0.59722500
H	-3.47672400	-0.61167200	0.43690000
C	-3.53141900	1.10616300	-0.80648500
C	-4.77725500	0.76876200	1.40380800
F	-5.86868100	0.33851100	0.75309600
F	-4.92648800	2.07544300	1.64717600
F	-4.71462900	0.12933600	2.57604500
F	-3.60007400	2.44114500	-0.76880800
F	-4.53189300	0.64833400	-1.55633900
F	-2.36989800	0.79472500	-1.44443900

2a

C	2.25734500	-0.78121000	-0.29172200
H	2.32801500	-0.66597400	-1.39193500
C	2.36919500	0.57975800	0.38354100
H	2.31329500	0.43643100	1.47611500
H	3.34914100	1.03033300	0.15884900
C	1.22140500	1.47363900	-0.09338800
H	1.15244800	2.38700000	0.51979500
H	1.42458700	1.81201800	-1.12636100
C	-0.09418800	0.72376500	-0.05455100
C	-1.32684300	1.39286900	-0.06451100
C	-0.10390700	-0.68457400	0.00619300
C	-2.53793200	0.70155200	-0.01426800
H	-1.32630100	2.48665900	-0.10609400
C	-1.31710700	-1.38744400	0.06660400
C	-2.52717300	-0.69742300	0.05578400
H	-3.48383900	1.24805400	-0.02311800
H	-1.27735400	-2.47735200	0.11980500
H	-3.46701000	-1.25379900	0.10073100
O	1.03573900	-1.43287200	0.03524300
H	3.05633200	-1.46598800	0.02817400

$MeOReO_3$

Re	0.25263200	0.00311200	0.00000000

O	0.51384400	-0.96195800	1.38746000
O	1.32115700	1.33686000	-0.00015700
O	0.51355100	-0.96207500	-1.38743300
O	-1.49314400	0.64106100	0.00012500
C	-2.75597500	-0.02037900	0.00001400
H	-2.85896500	-0.64763300	0.90074500
H	-3.53723300	0.75348800	-0.00035500
H	-2.85858900	-0.64810700	-0.90043200

TS4

Re	-0.49414300	-0.05780300	2.33362300
O	0.26696000	1.43673300	2.63670400
O	0.60639200	-1.21518700	2.95121200
O	-1.84362100	-0.19184200	3.36985700
O	-0.26546600	-1.07241900	0.53575600
C	0.71762600	-2.12312400	0.23429900
H	0.93541700	-2.62994200	1.18260100
C	1.96378100	-1.55129100	-0.40717500
H	2.58470600	-2.40776600	-0.71726400
H	1.68270700	-1.00802700	-1.32232300
C	2.79457000	-0.64448200	0.51646600
H	2.20394700	0.23354100	0.81347900
H	3.03363000	-1.20015700	1.43690500
C	4.06697600	-0.16692200	-0.14149100
C	4.20546100	1.13624100	-0.62681700
C	5.15182900	-1.06024800	-0.30339000
C	5.38098500	1.56360100	-1.25705500
H	3.37516800	1.83408100	-0.50281900
C	6.33378200	-0.64161200	-0.92857300
C	6.44115800	0.67132500	-1.40454900
H	5.46246000	2.58860000	-1.62572500
H	7.17326400	-1.32591100	-1.05109700
H	7.36660200	0.98880500	-1.89202200
O	-1.86438100	0.88859800	0.94645200
H	-0.87451100	-0.95849700	-0.34189800
O	4.95196900	-2.31878100	0.18390000
C	5.99512900	-3.26455600	0.11639800
H	6.89259500	-2.92622800	0.66570400
H	5.61673200	-4.18324300	0.58517000
H	6.28262100	-3.48816700	-0.92747900
H	0.19689000	-2.81897900	-0.43596100
C	-2.95185800	1.68790600	1.44250400
H	-2.56338600	2.51803200	2.05104100
H	-3.49649500	2.08305700	0.57469100

H	-3.60859800	1.05290400	2.05055400
O	-1.66268000	1.52845100	-1.40498800
H	-1.66505600	1.22408100	-0.04382100
C	-0.83352300	2.51695400	-1.88177100
H	-0.94920700	2.68713400	-2.96978100
C	0.63982300	2.13099000	-1.66929700
C	-1.20285200	3.84705600	-1.20282600
F	0.90299900	1.01072100	-2.36448800
F	1.49693000	3.07462800	-2.06544500
F	0.88734100	1.86239300	-0.37152800
F	-0.46405400	4.86347000	-1.66205000
F	-2.49030100	4.12800200	-1.43783400
F	-1.03733600	3.77678800	0.12930400
O	-1.44449100	-0.82590800	-1.59894200
H	-1.52020000	0.31011800	-1.68280700
C	-2.59983200	-1.49852000	-1.96728400
H	-3.10335500	-1.00424100	-2.81709500
C	-3.62131600	-1.50831800	-0.81389200
C	-2.22894500	-2.90846000	-2.45425600
F	-4.04973900	-0.25659600	-0.59114900
F	-4.68665500	-2.26536100	-1.08997700
F	-3.06703000	-1.95985000	0.32316700
F	-3.28600400	-3.53937300	-2.97543500
F	-1.28390900	-2.82879900	-3.39304600
F	-1.74884500	-3.66495900	-1.44808900

MeOH

O	0.74627100	0.12235100	0.00002000
H	1.13883400	-0.75955200	-0.00004300
C	-0.65516000	-0.01959500	0.00000900
H	-1.09198400	0.99153300	0.00077900
H	-1.04293000	-0.54585900	-0.89670200
H	-1.04312600	-0.54735900	0.89575000

TS2A

Re	-2.49562800	0.01454100	0.03951600
O	-1.52686600	1.34304000	-0.55053100
O	-3.36561500	0.48977100	1.44986400
O	-3.58031500	-0.53354400	-1.18261700
O	-1.32531800	-1.25391200	0.43904500
C	0.79397700	-0.45524000	0.06361900
H	0.31623600	-0.32722700	-0.90245800
C	1.53301800	-1.71460600	0.34516500
H	1.83212100	-1.76661600	1.40235400

H	0.79332100	-2.51161900	0.16889800
C	2.76047200	-1.89406700	-0.57138400
H	3.16392700	-2.90914600	-0.45032900
H	2.44098700	-1.79775100	-1.62453200
C	3.82355100	-0.87591200	-0.25033800
C	5.15680800	-1.20893600	0.00940100
C	3.46063100	0.47816600	-0.14948800
C	6.09722300	-0.22849900	0.34216900
H	5.45963500	-2.25745400	-0.05338800
C	4.37489300	1.47334000	0.19603300
C	5.70457200	1.10757100	0.43500300
H	7.13489500	-0.51034700	0.53276000
H	4.07541900	2.51781400	0.27207700
H	6.43145300	1.87968700	0.69685500
O	2.12409200	0.75268100	-0.43408500
C	1.62766700	2.10968800	-0.46045000
H	2.17439400	2.66574400	-1.23420800
H	0.55648900	2.04285400	-0.70240200
H	1.75829700	2.58031100	0.52582700
H	0.43021800	0.16984400	0.87302000

TS3A

Re	2.40370900	0.02149700	0.03096400
O	1.33168800	0.88656100	-1.03626400
O	3.92319400	-0.27888400	-0.72156200
O	2.61955400	0.92022700	1.48695100
O	1.56157900	-1.50170700	0.38793300
C	-3.09199500	-2.26699900	0.10785300
H	-2.96949400	-2.33792800	1.20295300
C	-4.54161400	-2.07224200	-0.28090100
H	-4.61099800	-1.99846200	-1.37879200
H	-5.10909500	-2.96457300	0.02608500
C	-5.08163800	-0.80704200	0.38183200
H	-6.08076700	-0.55822000	-0.00781100
H	-5.20716100	-0.98124000	1.46598100
C	-4.14251800	0.35820300	0.16196600
C	-4.58882600	1.68192300	0.29551600
C	-2.79890000	0.16717400	-0.19635200
C	-3.73797600	2.76567000	0.08422300
H	-5.63393500	1.85215500	0.56898400
C	-1.92839900	1.23593300	-0.43360500
C	-2.40943000	2.53777700	-0.28478300
H	-4.11198400	3.78534900	0.19890100
H	-0.88815900	1.08971900	-0.73022100

H	-1.72816200	3.37241100	-0.46285000
O	-2.28650300	-1.15017600	-0.36225600
C	-0.54501800	-1.36472100	0.00358000
H	-0.55300500	-0.82233100	0.94490900
H	-0.53724200	-2.45111700	0.03383400
H	-0.19730100	-0.86624300	-0.89504500
H	-2.64909900	-3.15402100	-0.36362900

TS4A

Re	1.64507500	-0.03891000	-0.03651600
O	1.81961200	-1.63493300	0.56791800
O	1.79918700	0.97700300	1.33739400
O	3.01060000	0.22990900	-1.02401600
O	0.28174100	1.33573200	-0.83705900
C	-0.14420700	2.63361500	-0.43950900
H	0.39370100	2.89360300	0.48839000
C	-1.65109700	2.71244900	-0.24106700
H	-1.91944400	3.78230700	-0.19206500
H	-2.15637000	2.30481500	-1.13161300
C	-2.18721600	2.02068300	1.02583600
H	-1.68878800	2.45628100	1.90721400
H	-3.25959000	2.26341000	1.11004300
C	-2.03438100	0.51770000	1.07444700
C	-1.14255300	-0.10453700	1.95366300
C	-2.82277700	-0.30334400	0.23578500
C	-1.01200100	-1.49858200	2.00754700
H	-0.51944100	0.51888000	2.59856800
C	-2.71785400	-1.69922500	0.29939600
C	-1.80548400	-2.29052200	1.18112800
H	-0.28382100	-1.95318500	2.68067900
H	-3.33549200	-2.33387200	-0.33614600
H	-1.71803600	-3.37930000	1.21260900
O	0.11403500	-0.77776500	-1.25079000
H	-0.24314900	0.33960400	-1.35305700
O	-3.66854400	0.35734100	-0.60664500
C	-4.52885100	-0.37781300	-1.44673300
H	-5.21634900	-1.02283200	-0.86984000
H	-5.11903400	0.35626100	-2.01193300
H	-3.96834900	-1.00974000	-2.16067200
H	0.17527600	3.34382300	-1.22263200
C	-0.10242000	-2.02438600	-1.88023600
H	0.24673200	-2.81892800	-1.20379700
H	-1.18000100	-2.15463500	-2.06055500
H	0.45156000	-2.08277000	-2.83246500

TS1-HFIP

Re	2.28784200	0.45709000	-1.12266800
O	3.43800100	-0.35903500	-0.15850100
O	3.06204400	1.94031100	-1.47474700
O	2.18976000	-0.34394800	-2.62341100
O	0.56785000	1.47994300	-0.74801200
C	0.36477000	2.89655100	-1.00004900
H	1.34685300	3.35340700	-1.18752100
C	-0.34597100	3.59002400	0.15285700
H	-0.57935200	4.61144100	-0.19573800
H	-1.31003200	3.10590500	0.36492900
C	0.47598700	3.68347000	1.45118600
H	1.40274600	4.24618600	1.25225800
H	-0.11071300	4.27053300	2.17640800
C	0.83274900	2.35220400	2.07121500
C	2.14466100	1.86843500	2.06678500
C	-0.17630900	1.54765200	2.64883700
C	2.47156900	0.61929100	2.60902200
H	2.92926400	2.48054600	1.61197500
C	0.14240100	0.29800000	3.20024900
C	1.46601200	-0.16035300	3.17655500
H	3.49782500	0.25385900	2.56046200
H	-0.63181600	-0.33071400	3.63848200
H	1.69542200	-1.14450100	3.58793800
O	0.88532500	-1.00724400	-0.20833400
H	-0.56212700	0.96926800	-0.36269200
Re	-0.06501000	-2.25773100	0.58713600
O	0.70333700	-2.93290200	1.95113300
O	-0.39897900	-3.51592600	-0.50972100
O	-1.59839200	-1.45174700	1.05985600
O	-1.43215700	2.06819700	2.61839400
C	-2.51438200	1.31959000	3.13596500
H	-2.40878000	1.15149900	4.22262700
H	-3.41825400	1.91246000	2.94598300
H	-2.61688000	0.34476000	2.63094300
H	-0.21903400	2.98141300	-1.93419300
C	-2.52943600	0.86273700	-1.12346300
H	-2.26246000	1.76360300	-1.69776600
C	-2.51496900	-0.31081200	-2.12125200
C	-3.90219400	1.13022200	-0.48874000
O	-1.61094700	0.65401200	-0.09171100
H	-1.70831400	-0.40363400	0.53248800
F	-3.01683400	-1.42678400	-1.58154400

F	-3.20682100	-0.01341000	-3.22050700
F	-4.22940600	0.16292700	0.37403800
F	-3.85922600	2.28838200	0.18607600
F	-4.85553200	1.21972200	-1.41832700
F	-1.24249400	-0.55898700	-2.47042500

TS1-2HFIP

Re	3.11789400	0.30566300	0.11191200
O	3.67134100	-1.29945600	0.31514300
O	4.17861900	1.20728200	1.10505200
O	3.47317700	0.81618800	-1.47504000
O	1.55276400	1.26447700	0.96441100
C	1.64228200	2.26786900	2.01633800
H	2.65381800	2.21843900	2.44205400
C	0.58820900	2.05035300	3.08770200
H	0.58963000	2.96094700	3.71228900
H	-0.41195900	1.99209500	2.63053000
C	0.83607600	0.82731900	3.98809400
H	1.86259500	0.87784800	4.38758600
H	0.14940700	0.89333200	4.84658300
C	0.62953100	-0.50624500	3.31123100
C	1.69626500	-1.31152900	2.90471900
C	-0.68574900	-0.96801800	3.07368400
C	1.48958800	-2.54328100	2.27226300
H	2.71848000	-0.96669300	3.08939400
C	-0.90525200	-2.20974800	2.46442000
C	0.18578000	-2.99179900	2.06556500
H	2.34135600	-3.13022800	1.92679400
H	-1.91768800	-2.56296000	2.27155100
H	0.00880200	-3.94757800	1.56879800
O	1.27474000	-0.68049900	-0.63681300
H	0.39167300	1.31710200	0.42300200
Re	0.39332900	-2.06026300	-1.30774200
O	1.13685300	-3.54376400	-0.92095600
O	0.36032600	-1.92329900	-3.00641000
O	-1.27551200	-2.03609900	-0.69255300
O	-1.68565300	-0.13250500	3.47454200
C	-2.99443500	-0.65006000	3.61682000
H	-3.00695600	-1.52551400	4.29057300
H	-3.60252100	0.15396000	4.05213400
H	-3.43116000	-0.93406800	2.64920800
H	1.52722500	3.25147700	1.54151100
C	-0.87103500	2.38513600	-1.00893700
H	-1.92373000	2.70671100	-1.02327900

C	-0.06918600	3.68164600	-0.81127300
C	-0.61160600	1.71686200	-2.37112200
O	-0.67313800	1.48885600	0.04149700
H	-1.60700600	0.64139800	0.22300900
F	1.25259800	3.47584100	-0.86740300
F	-0.39504700	4.57613700	-1.74650600
F	0.65877600	1.37690000	-2.54856300
F	-1.35991300	0.58517400	-2.42346400
F	-0.99812500	2.50289200	-3.37581300
F	-0.35799100	4.20165400	0.38934200
C	-3.63890100	0.16311100	-0.32398300
H	-3.49457200	0.52970700	-1.35396300
C	-4.52421700	-1.09437500	-0.43850100
C	-4.31434600	1.30040700	0.46375900
O	-2.41944800	-0.17170200	0.29218000
H	-1.91384700	-1.10876200	-0.15860800
F	-4.57738300	-1.76804000	0.71544100
F	-5.76668500	-0.75326900	-0.79184700
F	-4.82887500	0.89089800	1.62329300
F	-3.39971000	2.25267000	0.73005500
F	-5.28402200	1.85483600	-0.26407100
F	-4.02649600	-1.90613400	-1.37212800

TS2B

Re	2.58909900	-0.97354700	-0.12084400
O	2.69197200	0.40500300	0.95339600
O	1.76678000	-0.46058100	-1.55582200
O	4.14741700	-1.59637200	-0.48736400
O	1.59496300	-2.20401300	0.65362700
C	-0.63827500	-1.77565200	0.89176500
H	-0.44783800	-2.36260400	1.78888300
C	-0.98703000	-2.48374900	-0.37149300
H	-0.90060000	-1.79587800	-1.22262300
H	-0.22015700	-3.26406700	-0.48706200
C	-2.38688700	-3.13979900	-0.33196100
H	-2.50566700	-3.77959200	-1.21752000
H	-2.45195700	-3.79648400	0.55371200
C	-3.47127800	-2.09872500	-0.29806500
C	-4.51434400	-2.03538200	-1.22730600
C	-3.39700000	-1.08497200	0.66875700
C	-5.45576700	-1.00250900	-1.18505100
H	-4.58354900	-2.80844000	-1.99706700
C	-4.30510000	-0.02893300	0.71632600
C	-5.34658300	-0.00249500	-0.21699600

H	-6.26675100	-0.97285100	-1.91579700
H	-4.20902200	0.77339200	1.44526200
H	-6.06824400	0.81683100	-0.18662100
O	-2.33685000	-1.21757900	1.56812500
C	-2.16706900	-0.24792700	2.62235800
H	-3.08741300	-0.21346400	3.22117400
H	-1.33825800	-0.61318900	3.24033800
H	-1.91000100	0.73513200	2.20620200
H	-0.28556400	-0.74658400	0.88320400
O	0.24472900	1.38838600	1.10228600
H	1.19808300	1.14865000	1.24612800
C	0.16754200	2.06293700	-0.11600400
H	0.84080100	1.64232100	-0.88473900
C	0.55072400	3.54124200	0.06058100
C	-1.25258700	1.87616100	-0.65249900
F	-1.46401400	2.56775500	-1.77109800
F	-2.18399500	2.23993200	0.24846900
F	-1.46289200	0.57151300	-0.92581400
F	-0.33145800	4.20206300	0.82381400
F	0.62851600	4.16571200	-1.12206800
F	1.74757200	3.61446100	0.65262300

TS3B

Re	-0.35630400	-0.25465500	-0.77794800
O	0.90543500	-1.21675200	-1.47365800
O	-1.67572800	-0.08789300	-1.86988100
O	-0.90163500	-0.99556100	0.69639500
O	0.40263700	1.30992000	-0.44381000
C	4.96496800	2.31689200	0.17285500
H	4.58435100	2.52680700	1.18801200
C	6.47091300	2.16396000	0.16451600
H	6.80430800	1.95121400	-0.86467400
H	6.92144800	3.12256200	0.46538800
C	6.87337600	1.03281500	1.10835900
H	7.94427200	0.79980800	1.00441800
H	6.72700200	1.35357400	2.15579000
C	6.05196900	-0.20836100	0.83889600
C	6.49475800	-1.47468200	1.25089800
C	4.82876600	-0.14711200	0.15307200
C	5.75517700	-2.62815400	0.99472400
H	7.44831800	-1.54362600	1.78185300
C	4.07753200	-1.29180800	-0.13489600
C	4.54827200	-2.53283600	0.29632200
H	6.12294500	-3.59996600	1.33079600
H	3.13726000	-1.24600200	-0.68522000

H	3.95844800	-3.42538700	0.07746400
O	4.32625700	1.09959900	-0.30363100
C	2.50626500	1.25185000	-0.35170800
H	2.34807800	0.80417800	0.62519900
H	2.47252000	2.33424100	-0.44276600
H	2.40815300	0.64617000	-1.24610600
H	4.62177100	3.10382300	-0.51171100
O	-3.45824000	-0.81207300	1.65516700
H	-2.50777400	-0.94071300	1.46158100
C	-4.04620800	-0.22264100	0.54545900
H	-3.60208100	-0.52740100	-0.41914600
C	-3.89469200	1.30720300	0.60127900
C	-5.51000700	-0.67332900	0.49869600
F	-6.16675700	-0.08259600	-0.51472400
F	-6.16415300	-0.39440100	1.63070700
F	-5.56652500	-1.99761800	0.30398500
F	-4.53668000	1.85051400	1.64124900
F	-4.33460800	1.89882500	-0.51608600
F	-2.58489700	1.61407100	0.73372800

TS4B

Re	2.13644400	-0.94669900	-0.25788600
O	3.14774500	-1.02492000	1.12127400
O	3.10697100	-0.12228900	-1.40306800
O	2.00379400	-2.55111900	-0.81962000
O	0.49319100	-0.04346500	-1.23947900
C	0.49233400	0.65633200	-2.50732300
H	1.53023300	0.67625000	-2.86707400
C	-0.08227500	2.05569500	-2.36834800
H	-0.17658100	2.46358200	-3.38990800
H	-1.10218700	1.99560900	-1.96260600
C	0.76089000	3.02902000	-1.52323700
H	1.76171400	3.12756800	-1.97430100
H	0.28083800	4.02014200	-1.58380900
C	0.91731100	2.66069600	-0.06435400
C	2.16077800	2.31562500	0.47755500
C	-0.20903600	2.65564700	0.79110000
C	2.30737300	1.96611300	1.82800700
H	3.03582900	2.31427700	-0.17683700
C	-0.07252200	2.29801200	2.14102300
C	1.18451500	1.96194700	2.65384700
H	3.28779100	1.68565500	2.21572400
H	-0.94316300	2.28003500	2.79604400
H	1.27733700	1.68796500	3.70764300

O	0.54805200	-0.88149500	1.15434500
H	-0.50137800	0.01755300	-0.71748900
O	-1.38328100	3.01628100	0.21840700
C	-2.58640500	2.89765200	0.95354200
H	-2.59971300	3.58005400	1.82252700
H	-3.39584800	3.17600600	0.26650300
H	-2.74529200	1.86061900	1.28528400
H	-0.11007200	0.05450500	-3.20641300
C	0.62287400	-1.31665400	2.51656200
H	1.58038500	-0.99177700	2.94187900
H	-0.20967200	-0.86278500	3.07013400
H	0.54860000	-2.41401100	2.56157000
O	-1.47379300	-0.06962900	0.13732100
H	-0.44599800	-0.53917300	0.85201200
C	-2.43528700	-0.99803200	-0.19906400
H	-2.02597200	-1.94696800	-0.60486900
C	-3.32743800	-0.42563000	-1.31135300
C	-3.22256000	-1.39682900	1.05820900
F	-2.55594200	-0.13975900	-2.37974400
F	-4.25960800	-1.30110800	-1.70309800
F	-3.94033800	0.70407700	-0.94262800
F	-4.12854700	-2.34491800	0.80009200
F	-2.36057900	-1.88713100	1.97140500
F	-3.85331000	-0.35639900	1.61786400

TS1’

O	1.13452800	0.58762500	1.82668500
C	-0.38149700	1.64093300	0.44874800
H	0.55316100	2.06051200	0.08317400
C	-1.11496700	2.29907600	1.56306200
H	-1.97113300	1.67653600	1.85829600
H	-0.40866300	2.30806000	2.40633700
C	-1.56129700	3.73708900	1.22601100
H	-1.90154100	4.23430700	2.14526700
H	-0.69302300	4.30919700	0.85233800
C	-2.66982600	3.73040700	0.20830400
C	-3.88901000	4.39033600	0.39461300
C	-2.51168500	2.96826700	-0.95969300
C	-4.91046100	4.30442100	-0.55575000
H	-4.03616900	4.97884500	1.30407100
C	-3.52458600	2.84586200	-1.91065700
C	-4.72580400	3.53103000	-1.70344800
H	-5.85282400	4.83211600	-0.39425600
H	-3.39888500	2.22323500	-2.79487600

H	-5.52271200	3.44654000	-2.44574000
O	1.60765000	-0.31084300	-1.64914100
H	1.08037600	-0.37177500	1.64910200
Re	0.76759700	-1.78629100	-1.19181300
O	1.56166400	-3.16292100	-1.83281800
O	-0.84714100	-1.69242600	-1.80002500
O	0.67544900	-1.88158200	0.56113800
O	-1.27234100	2.34641900	-1.09460000
C	-0.96315300	1.59088500	-2.28629200
H	-1.10108200	2.24200000	-3.16024500
H	0.08805400	1.29301700	-2.19956300
H	-1.59074200	0.69183100	-2.34569800
H	-0.62917600	0.61499300	0.19640700
H	1.96729800	0.85043400	1.38782400
O	-1.69768400	-1.00567900	1.49736400
H	-0.85957000	-1.49369000	1.35012600
C	-2.73393100	-1.67762700	0.85521000
H	-2.40850400	-2.22394500	-0.04656900
C	-3.72148200	-0.61575500	0.36573900
C	-3.36905600	-2.71134600	1.79753100
F	-4.26985200	-3.46650100	1.15326500
F	-3.97491700	-2.13858700	2.84366400
F	-2.40626100	-3.51942000	2.26277100
F	-4.09094400	0.22538700	1.33928100
F	-4.81997900	-1.15651700	-0.16485700
F	-3.12493000	0.12715700	-0.59312200
O	2.98696300	1.26403100	-0.14066300
H	2.55083700	0.67090400	-0.81596800
C	4.35096100	1.00455100	-0.05892500
H	4.84584700	0.95723700	-1.04596900
C	4.59890700	-0.35659100	0.61815000
C	4.98509100	2.18214500	0.69277400
F	6.29940700	1.99846700	0.86317600
F	4.42389400	2.35295300	1.89623300
F	4.80964700	3.30749600	-0.00961100
F	4.07419700	-0.39425100	1.85015500
F	5.89848500	-0.64694300	0.69799600
F	4.00089200	-1.31898000	-0.10716100

TS1’-A

O	-0.50066400	2.81507100	-0.54206300
C	1.05335400	1.34838200	0.25982100
H	0.44130200	1.45861200	1.15143000
C	2.18366800	2.29321800	-0.03795600

H	2.30158800	2.37126200	-1.12796400
H	1.88376000	3.28364400	0.33090400
C	3.53959300	1.89624000	0.59610000
H	4.28894500	2.65373900	0.32628500
H	3.43652500	1.91040400	1.69470700
C	3.97882000	0.53193200	0.14379900
C	5.17765400	0.25327900	-0.51762800
C	3.08897100	-0.53016200	0.36411800
C	5.47808500	-1.04965200	-0.93132800
H	5.88357200	1.06640400	-0.70621200
C	3.35306300	-1.83322800	-0.05291400
C	4.56927100	-2.08472700	-0.69969000
H	6.42227100	-1.25509000	-1.44029800
H	2.64002600	-2.63987100	0.11414800
H	4.79826300	-3.10112100	-1.02768700
O	-1.75271200	0.64981700	1.29643200
H	-0.93186700	2.17229300	-1.15755500
Re	-2.29987700	-0.25580200	-0.09681100
O	-4.01999500	-0.29159700	-0.16756800
O	-1.64822200	-1.86013000	-0.01418300
O	-1.62991800	0.56942400	-1.49589200
O	1.92416000	-0.14865400	1.02246300
C	1.00101800	-1.15895100	1.50223400
H	1.55027200	-1.82143800	2.18499300
H	0.20644900	-0.61906200	2.02995600
H	0.55292700	-1.71493500	0.66772700
H	0.61732900	0.74736700	-0.53644600
H	-1.12271800	2.80347900	0.20396500

TS1’-B

O	0.33640100	0.44594600	2.44889500
C	-1.33717100	-0.03712300	0.93572200
H	-0.69412400	0.55783800	0.28957700
C	-2.37113600	0.65270800	1.76821000
H	-2.77948400	-0.03267900	2.52477000
H	-1.84168900	1.45459400	2.29955300
C	-3.51813200	1.27348800	0.93692300
H	-4.12986200	1.91144900	1.59043700
H	-3.08049700	1.92126100	0.15817100
C	-4.36546500	0.19548700	0.32072300
C	-5.74289500	0.06216200	0.51584800
C	-3.71325600	-0.78570800	-0.44267900
C	-6.44736900	-1.00877200	-0.04561900
H	-6.26866000	0.81092500	1.11434400

C	-4.38717600	-1.87556500	-0.99331300
C	-5.76939300	-1.97317300	-0.79417200
H	-7.52539800	-1.09267900	0.10809600
H	-3.85934000	-2.63627200	-1.56752400
H	-6.31279200	-2.81603300	-1.22731900
O	1.75875700	-0.76179900	-1.24964500
H	1.09329900	-0.17395700	2.31475800
Re	1.92301600	-1.93788200	0.05129100
O	3.10675000	-3.11444100	-0.35287100
O	0.37525100	-2.68862100	0.28635000
O	2.35145700	-1.11146000	1.52478500
O	-2.35132700	-0.57161800	-0.59749300
C	-1.60485600	-1.33107400	-1.57332800
H	-2.12484500	-1.25122500	-2.53800800
H	-0.61844100	-0.86352800	-1.63897900
H	-1.49516100	-2.37340500	-1.24824300
H	-1.01546500	-1.05638400	1.14742100
H	0.69545300	1.28610500	2.12706600
O	0.41253500	1.40526100	-1.20698200
H	1.08754200	0.67134300	-1.33607300
C	0.95033200	2.66846200	-1.06424900
H	1.13280200	3.18401700	-2.02893300
C	2.31443900	2.65242800	-0.34993000
C	-0.09776300	3.51465800	-0.32441500
F	0.27620800	4.78901300	-0.21126900
F	-0.33145500	3.03896000	0.92062400
F	-1.26368300	3.47475600	-0.98252500
F	2.23606600	2.11147000	0.88142900
F	2.81280700	3.88646400	-0.22584300
F	3.17349500	1.91775900	-1.06003300

TS1C

O	0.28030500	-0.63490200	3.06302000
C	-0.42855900	0.55819400	3.29057000
H	-1.25266000	0.68467300	2.56398900
C	0.50657300	1.77797600	3.27208800
H	-0.01613600	2.67168700	3.65801400
H	1.32511500	1.57151100	3.97953000
C	1.13043700	2.04423500	1.90093600
H	2.00469300	2.71251800	1.98856000
H	1.53927700	1.09251600	1.53287700
C	0.21311700	2.61283900	0.84067100
C	-1.06793000	3.09136300	1.08935400
C	0.67204000	2.69616700	-0.50135100

C	-1.91358400	3.65430000	0.09645900
H	-1.45764400	3.02659400	2.10547300
C	-0.20804800	3.13767800	-1.56161700
C	-1.48046100	3.72662600	-1.19870100
H	-2.90488500	4.01157400	0.37902200
H	0.24503500	3.45826100	-2.50484500
H	-2.10934700	4.13918500	-1.99046100
H	0.25515400	-0.84143500	2.11691100
O	1.87182600	2.20406300	-0.74581500
C	2.44798700	2.21074000	-2.06116100
H	2.56280000	3.24402100	-2.42405100
H	3.42221100	1.72549100	-1.95117600
H	1.82763500	1.61929700	-2.75133900
H	-0.89877500	0.48965300	4.28768300
H	-0.60662300	1.97389100	-1.83379000
Re	-0.09380200	-0.82073500	-1.44861000
O	-1.06810700	-2.21307300	-1.67015800
O	0.32168000	-0.64746700	0.21490900
O	1.36779600	-0.89863600	-2.37565300
O	-1.02705200	0.62687200	-1.94380100
O	-2.79556900	0.63755300	0.23418800
H	-2.43239600	0.82539600	-0.64952000
C	-3.55394500	-0.53238900	0.17131700
H	-3.19676800	-1.24636200	-0.59304500
C	-5.00596600	-0.19195100	-0.19606400
C	-3.40607900	-1.25369900	1.51683200
F	-4.22246400	-2.31153700	1.58797100
F	-3.67556700	-0.44259200	2.54620100
F	-2.14595900	-1.68693300	1.65107300
F	-5.61394000	0.52725900	0.75285900
F	-5.73003300	-1.29246700	-0.42332400
F	-4.99775000	0.53879500	-1.32639400
O	3.94518000	-0.59799400	-1.34020500
H	3.15897700	-0.85789400	-1.85797000
C	3.55177200	-0.49235400	-0.00833100
H	2.58463500	0.01652900	0.11629200
C	4.56981300	0.38899200	0.71989100
C	3.37912600	-1.87690700	0.64042400
F	2.80648800	-1.77861000	1.84365200
F	4.53572500	-2.53212400	0.77276000
F	2.57560800	-2.62456000	-0.14344600
F	5.80800700	-0.10036900	0.67615000
F	4.22028200	0.54707100	2.00656400
F	4.59278200	1.61338200	0.15746500

Int1C

O	-3.60775800	0.47072400	-0.34786300
C	-3.40938000	-0.70063600	0.41753400
H	-3.97237100	-0.67290100	1.37128000
C	-1.92030200	-0.79632900	0.72484200
H	-1.63067700	0.10979300	1.28238200
H	-1.72893400	-1.66151600	1.38066100
C	-1.07790900	-0.90048900	-0.56296300
H	-1.26343900	-1.86846900	-1.05404300
H	-1.42054900	-0.11212700	-1.24952100
C	0.39784500	-0.74480200	-0.31408400
C	1.27899200	-1.81563800	-0.24938500
C	0.94973500	0.54418300	-0.09758100
C	2.68524700	-1.70254200	0.01440800
H	0.87074000	-2.81763500	-0.41552800
C	2.39957800	0.74364800	0.17846500
C	3.23704900	-0.48181100	0.22205200
H	3.29434200	-2.60790500	0.04316700
H	2.81449500	1.44866200	-0.57188500
H	4.30378600	-0.35905800	0.42476500
H	-4.53483800	0.52595100	-0.61221100
O	0.13042800	1.55073200	-0.15699300
C	0.53592100	2.91742300	0.03375500
H	-0.37724500	3.51195600	-0.07645300
H	0.95683400	3.05397700	1.04193800
H	1.27193900	3.20840300	-0.73163000
H	-3.73579000	-1.61003200	-0.12835000
H	2.50970000	1.30120800	1.13201700

TS2C

O	0.53462200	2.40564600	1.18131900
C	-0.77423000	2.92823800	1.52934100
H	-1.22100300	2.20373700	2.22536800
C	-1.65747000	3.14300400	0.30636700
H	-1.90248600	2.16888000	-0.14226800
H	-2.60767300	3.58620800	0.64391700
C	-0.97730600	4.02388600	-0.76073400
H	-1.62403000	4.09934600	-1.64685600
H	-0.81285700	5.04161200	-0.37194300
C	0.33163000	3.37722800	-1.13022300
C	0.50505600	2.60362900	-2.22900500
C	1.35797200	3.35996200	-0.06424400
C	1.66517300	1.75181400	-2.43422900

H	-0.29706600	2.56478300	-2.96982400
C	2.63873800	2.57972900	-0.30373500
C	2.64461300	1.69986800	-1.51489800
H	1.69531100	1.11037200	-3.31623600
H	3.43789300	3.33307100	-0.43647400
H	3.50826200	1.04619000	-1.63412800
H	0.47738800	1.40651100	0.90505600
O	1.45436600	4.53371300	0.57569500
C	2.38914100	4.66655000	1.64967800
H	2.13986800	5.60737400	2.15548900
H	2.29270100	3.82355800	2.35044500
H	3.42356500	4.72142300	1.27506800
H	-0.58676100	3.87083900	2.06355700
H	2.92701400	1.99894800	0.58743000
Re	-0.27515100	-0.86395300	-0.82427000
O	-1.17339200	-2.22601600	-0.25070200
O	0.48149100	0.01349900	0.53419500
O	0.95479800	-1.39039200	-1.90302800
O	-1.35562500	0.20667000	-1.63652900
O	3.02365800	-0.30632000	1.31701100
H	2.04882400	-0.27434200	1.20169100
C	3.52464300	-1.22741300	0.40256000
H	3.09193000	-1.13038300	-0.61050100
C	3.20231000	-2.66617700	0.84278700
C	5.02633600	-0.96370300	0.26551000
F	5.59448400	-1.82595200	-0.58520400
F	5.66676200	-1.03446700	1.43160400
F	5.21806400	0.28005700	-0.22866000
F	3.79305200	-2.99551600	1.99358000
F	3.56049200	-3.55548700	-0.08823000
F	1.86915800	-2.76719000	1.02192000
O	-3.66055100	-2.21086700	0.84466100
H	-2.75478700	-2.40871000	0.53307900
C	-4.05697200	-1.01284100	0.27488700
H	-3.50331800	-0.74215600	-0.64193200
C	-3.81121700	0.13827800	1.26277300
C	-5.52937400	-1.13348700	-0.14174100
F	-5.96459500	0.00732300	-0.70602700
F	-6.32567800	-1.40957100	0.89545700
F	-5.65913000	-2.11163500	-1.04312200
F	-4.45476900	-0.01653900	2.41665600
F	-4.15273500	1.33436100	0.75025300
F	-2.48351400	0.20021500	1.54374700

Int2C

O	0.52987700	2.43066100	1.10456400
C	-0.79804400	2.92898200	1.47190800
H	-1.20974400	2.17009700	2.15006600
C	-1.68051300	3.14566900	0.25160100
H	-1.93255700	2.17255500	-0.19440900
H	-2.62655000	3.59607200	0.59162900
C	-0.98456200	4.01782200	-0.81253200
H	-1.61093800	4.07768100	-1.71422800
H	-0.83391300	5.04043100	-0.43065400
C	0.33687200	3.37454200	-1.13535100
C	0.57414400	2.64720300	-2.24745900
C	1.29432300	3.31153600	0.02048600
C	1.75065800	1.80276500	-2.41410000
H	-0.18271500	2.63540500	-3.03574300
C	2.62412700	2.59289200	-0.22460400
C	2.68762000	1.73647300	-1.45376800
H	1.83030200	1.18776100	-3.31213100
H	3.38449900	3.38629700	-0.34109700
H	3.56714000	1.10075900	-1.55657200
H	0.50086300	1.37713200	0.82913300
O	1.38913400	4.53747900	0.61633300
C	2.26329200	4.68339700	1.73389300
H	2.02094800	5.65251800	2.18851900
H	2.10557600	3.87885200	2.46983500
H	3.32131900	4.68992200	1.42516800
H	-0.62171200	3.86117400	2.02493400
H	2.92664400	2.01294300	0.66146300
Re	-0.26076000	-0.82790300	-0.83706600
O	-1.12645100	-2.19701800	-0.23816100
O	0.50041000	0.08331200	0.51264100
O	0.95963600	-1.34985200	-1.92402900
O	-1.36994800	0.20583100	-1.65041500
O	3.02480600	-0.34429000	1.36735900
H	2.05621100	-0.27918400	1.23451200
C	3.51959900	-1.23371500	0.41882200
H	3.10337400	-1.08195400	-0.59424000
C	3.16140300	-2.68301200	0.79057700
C	5.02803000	-0.99505500	0.31128100
F	5.58937000	-1.83920000	-0.56258100
F	5.64956200	-1.12293100	1.48272700
F	5.25077300	0.25994400	-0.13443300
F	3.73299600	-3.07862300	1.92940200
F	3.50382500	-3.53788900	-0.17706100

F	1.82295500	-2.75871400	0.95549400
O	-3.63217800	-2.23759700	0.83845700
H	-2.72018900	-2.41650600	0.53648800
C	-4.04505900	-1.04240500	0.27403000
H	-3.50807600	-0.76864100	-0.65184700
C	-3.79365100	0.11213500	1.25668400
C	-5.52298400	-1.17553100	-0.11907300
F	-5.97708100	-0.03623300	-0.67075900
F	-6.29870100	-1.46340500	0.93004800
F	-5.65785400	-2.15092500	-1.02231600
F	-4.42304100	-0.04212500	2.41772700
F	-4.14296900	1.30560700	0.74502100
F	-2.46175900	0.17882700	1.52348600

Int3C

O	0.70633900	-3.35575600	0.17658500
C	1.00911500	-3.55390400	1.58068400
H	0.58502000	-4.53690200	1.82801500
C	0.41266200	-2.43230000	2.41811900
H	-0.68600000	-2.48106900	2.36284500
H	0.68437200	-2.58600600	3.47433300
C	0.90920100	-1.06498800	1.92798700
H	0.31873200	-0.25526500	2.37997600
H	1.95353000	-0.91193900	2.25235200
C	0.87082300	-0.93325800	0.42539200
C	0.66934600	0.23490000	-0.22005300
C	1.26807000	-2.15277200	-0.39476300
C	0.79265400	0.37198400	-1.66877500
H	0.46611800	1.13399700	0.36247800
C	0.76620800	-2.08882100	-1.84000300
C	0.88016500	-0.71975500	-2.44922300
H	0.76645600	1.37744700	-2.09093900
H	1.26377400	-2.86156400	-2.44111700
H	0.92923500	-0.63869000	-3.53761800
H	-0.82944600	-3.30468900	0.05645800
O	2.68140200	-2.23092600	-0.30018700
C	3.37905500	-3.23023100	-1.04378000
H	4.37938700	-3.30140500	-0.59727800
H	2.86881600	-4.20349100	-0.97158600
H	3.48922600	-2.93583400	-2.09924300
H	2.10346500	-3.60499400	1.69307200
H	-0.30323100	-2.36224200	-1.84115400
Re	-2.72866000	-1.55006900	0.02056000
O	-4.39721600	-1.88992200	0.05830400

O	-1.84298200	-3.16208600	0.05690300
O	-2.34362500	-0.68365900	-1.41745600
O	-2.31412000	-0.58791900	1.37486200
O	-1.78233200	2.04062500	-1.52219800
H	-1.99515100	1.09829400	-1.65522500
C	-2.13380000	2.40681900	-0.22865000
H	-1.85543200	1.66377300	0.54018400
C	-3.65637800	2.57990000	-0.09393100
C	-1.35169100	3.68132300	0.10820000
F	-1.65251500	4.11834000	1.33825800
F	-1.58557700	4.66569700	-0.75862400
F	-0.03159100	3.41350100	0.08208500
F	-4.13386500	3.56095100	-0.85993900
F	-4.01421900	2.81580100	1.17307300
F	-4.25370700	1.43059300	-0.47958900
O	4.11036000	-0.47002400	1.11794900
H	3.62378000	-1.21524700	0.71150400
C	4.13988600	0.54167900	0.16563900
H	3.31338600	0.47337700	-0.56195500
C	5.42678000	0.44413800	-0.66576000
C	3.96252500	1.88439700	0.88037600
F	3.98272700	2.90110000	0.00685300
F	4.90735700	2.09947800	1.79765100
F	2.76991000	1.90086700	1.50180900
F	6.52989800	0.61620700	0.06340700
F	5.43789000	1.33365600	-1.66744100
F	5.48404100	-0.78837800	-1.21686600

TS3C

O	1.95677300	4.19377400	0.87842100
C	2.92663400	3.90994500	1.91596800
H	3.03174100	4.85603500	2.46248400
C	4.23864700	3.42112400	1.32724200
H	4.68004300	4.22262600	0.71115100
H	4.94270800	3.20867500	2.14691300
C	3.98706400	2.16812800	0.48212000
H	4.89466600	1.84395600	-0.04728900
H	3.70246900	1.32434800	1.13368000
C	2.87349400	2.40751300	-0.50145700
C	2.77165600	1.80620500	-1.71269500
C	1.74294500	3.26200200	-0.05529400
C	1.62115900	1.96225800	-2.59025500
H	3.57165800	1.12910000	-2.02356000
C	0.80834500	3.77224200	-1.12527300

C	0.66353300	2.86602700	-2.30792400
H	1.53134900	1.30798800	-3.45881300
H	-0.15890400	4.04772100	-0.68209400
H	-0.21109000	2.98859500	-2.94933900
H	0.72962100	1.16252700	0.36740700
O	0.81812300	2.07378400	0.85407200
C	-0.44252900	2.47546900	1.41272400
H	-0.67468700	1.80261400	2.24802300
H	-0.32679400	3.50313000	1.78315500
H	-1.24594000	2.41318800	0.66541100
H	2.48859000	3.15400500	2.58875500
H	1.25708500	4.72821800	-1.46076500
Re	-0.82550100	-0.41481100	-1.47460000
O	-1.71990900	-1.81746700	-1.00163400
O	0.51051400	-0.10084800	-0.32510300
O	-0.19557900	-0.64161000	-3.05352600
O	-1.88034900	0.95070500	-1.45412500
O	3.04093400	-0.82513300	-0.18768700
H	2.13962100	-0.50916700	-0.42112800
C	2.85864300	-1.88781000	0.68670300
H	1.95626500	-1.77713600	1.31531300
C	4.05122900	-1.91427100	1.64596500
C	2.66560600	-3.19810100	-0.09533000
F	2.34308000	-4.21230200	0.71720100
F	3.75621700	-3.54174400	-0.78599000
F	1.65628700	-3.03274800	-0.97028000
F	5.21857000	-1.97231300	1.00272300
F	3.98015700	-2.95110600	2.48966200
F	4.04815400	-0.78577700	2.38658900
O	-3.92096500	-1.88592100	0.62578000
H	-3.14024400	-2.06505000	0.06493700
C	-4.13130800	-0.51707000	0.64004900
H	-3.80808700	0.00244200	-0.27942600
C	-3.33443600	0.13412800	1.78254300
C	-5.64000600	-0.26681600	0.75785600
F	-5.89922500	1.04971100	0.86090000
F	-6.17280400	-0.87604300	1.81975700
F	-6.25582200	-0.72121200	-0.33856800
F	-3.74061400	-0.24760800	2.99220000
F	-3.38170900	1.47953000	1.73274400
F	-2.02872200	-0.21251100	1.66672600

Int4C

O	-0.98518200	-1.40043600	0.06656100

C	-2.32591800	-0.81636100	0.20076100
H	-2.98794400	-1.54974000	-0.27549300
C	-2.39363300	0.55995900	-0.43108000
H	-2.27108900	0.46957500	-1.52324500
H	-3.39487200	0.97949900	-0.25253400
C	-1.30311600	1.46352300	0.15488800
H	-1.25353100	2.42232000	-0.38253000
H	-1.53667000	1.70478100	1.20710100
C	0.03502500	0.76353500	0.08790500
C	1.25370600	1.42730300	0.05903100
C	0.07219700	-0.65153100	0.03542500
C	2.53058300	0.77974800	-0.01284600
H	1.23698700	2.52205900	0.09020700
C	1.35801200	-1.39501400	-0.06600700
C	2.59398100	-0.57520400	-0.07805800
H	3.43563600	1.38964200	-0.02311100
H	1.39657900	-2.13655400	0.75811800
H	3.55082100	-1.09957100	-0.14408700
H	-2.53677200	-0.79520300	1.28175300
H	1.31728700	-2.03907200	-0.96878100

TS4C

O	1.32245100	-3.20360300	1.26914100
C	2.35625500	-2.26755000	1.65719400
H	3.28354900	-2.85380300	1.66245200
C	2.06051900	-1.62557600	3.00123200
H	2.08379500	-2.40320900	3.78309200
H	2.85604400	-0.90028300	3.22595600
C	0.69193100	-0.93813000	2.96939900
H	0.37756400	-0.64067500	3.98139500
H	0.76229400	-0.00736200	2.38333400
C	-0.35017800	-1.84092300	2.35019500
C	-1.71734600	-1.64823200	2.52238000
C	0.05676700	-2.91925700	1.53120200
C	-2.69899600	-2.48229000	1.93605900
H	-2.04362400	-0.79197000	3.11702400
C	-0.91658500	-3.74655100	0.85730200
C	-2.30969700	-3.54515100	1.15724700
H	-3.75458400	-2.26298400	2.10124800
H	-0.80618700	-3.25173900	-0.33158400
H	-3.04604200	-4.20887100	0.69850900
H	2.42900800	-1.51752500	0.85700900
H	-0.56999200	-4.74760500	0.56895500
Re	0.05691100	-1.07131000	-1.72414600

O	0.17043500	-0.43434200	-0.10786100
O	1.65070700	-1.03866900	-2.39260600
O	-0.54121000	-2.73642200	-1.55095600
O	-1.00816000	-0.11928100	-2.66687900
O	3.41498400	1.05331200	-1.55059000
H	2.95773500	0.35587800	-2.05357700
C	2.81184700	1.15675000	-0.30546900
H	2.19314700	0.28962200	-0.04241500
C	1.84093900	2.34967600	-0.26288500
C	3.89490900	1.20751400	0.77548000
F	3.34232100	1.32539200	2.00336200
F	4.75891300	2.20340200	0.61114100
F	4.57727100	0.04740200	0.76535000
F	2.45570600	3.52291500	-0.40150000
F	1.14285900	2.37063700	0.88509000
F	0.95039900	2.22392300	-1.26902500
O	-1.48851700	1.09035500	1.31676500
H	-0.77370100	0.55633800	0.91804800
C	-2.23164100	1.61104800	0.26240300
H	-1.66311200	1.67816800	-0.68140100
C	-2.62746300	3.04743000	0.62354200
C	-3.43225600	0.69423400	-0.01887300
F	-4.19995000	1.14010500	-1.01335500
F	-4.19972800	0.52523600	1.06724300
F	-2.97543500	-0.53147100	-0.36977300
F	-3.33831700	3.09809100	1.75623200
F	-3.35722300	3.60797000	-0.35209700
F	-1.52278500	3.78256300	0.78937400

TS2D

O	-1.63662800	-0.68192000	-0.90170300
C	-2.12465000	-2.02913300	-0.89550900
H	-1.69708100	-2.54305700	-1.77173700
C	-3.64952800	-2.02121600	-0.95387000
H	-3.95918100	-1.57953900	-1.91548500
H	-4.00840900	-3.06343500	-0.93709900
C	-4.30036500	-1.21363700	0.19506300
H	-5.38642500	-1.14571300	0.03162300
H	-4.13538000	-1.72799500	1.15466500
C	-3.68967800	0.16286800	0.22463100
C	-4.24592500	1.25886200	-0.35156100
C	-2.27638800	0.22297300	0.63735400
C	-3.51420400	2.48941200	-0.60039200
H	-5.27974000	1.19256800	-0.70443100

C	-1.54449900	1.53681600	0.56298400
C	-2.22611800	2.60674400	-0.22455000
H	-4.01874200	3.29979700	-1.13108900
H	-1.47258900	1.89036300	1.61165900
H	-1.64864900	3.51096900	-0.43099100
H	-0.58701300	-0.68255400	-0.95847800
O	-1.97795500	-0.62534500	1.60978500
C	-0.62909000	-0.78306800	2.08580100
H	-0.70551100	-1.32992500	3.03217100
H	-0.05703600	-1.37294400	1.35744800
H	-0.12435300	0.17983800	2.24576300
H	-1.77039400	-2.55469400	0.00913600
H	-0.49173600	1.41490200	0.26323300
Re	2.19253500	0.01607200	-0.11168600
O	3.50251600	0.30291300	-1.18799200
O	0.82681000	-0.74973100	-0.95562500
O	2.68862900	-1.01331500	1.18001400
O	1.60973000	1.50768200	0.56055900

Int2D

O	1.87012100	-0.99092000	-1.03083800
C	2.09070800	-2.27737700	-0.41527300
H	1.79542100	-3.01633200	-1.17391000
C	1.27692100	-2.40526900	0.86493700
H	0.20378800	-2.40711100	0.62074300
H	1.49782700	-3.37368600	1.34210300
C	1.59416200	-1.24623300	1.82186300
H	0.88357000	-1.23269100	2.66082500
H	2.60537200	-1.40356200	2.24096900
C	1.59476600	0.09329000	1.13115800
C	1.15186500	1.23859400	1.68923000
C	2.29169800	0.14838100	-0.22022200
C	1.32321800	2.54188100	1.05249400
H	0.67117400	1.19771000	2.67087900
C	1.90784800	1.38583000	-1.04622300
C	1.72684800	2.63080200	-0.22558200
H	1.09676000	3.43905200	1.63304700
H	2.62932700	1.52447900	-1.86300200
H	1.83002500	3.59854600	-0.72258000
H	0.33952900	-0.86554200	-1.30605200
O	3.65813100	0.02959000	0.06013200
C	4.56966000	0.07177300	-1.02577400
H	5.51250600	-0.36124600	-0.66243300
H	4.21074000	-0.52028300	-1.88495500

H	4.76750000	1.10632100	-1.35722000
H	3.16840000	-2.38910200	-0.21574600
H	0.94348500	1.17548800	-1.54192600
Re	-1.80882700	-0.01207300	-0.12655700
O	-3.40796600	-0.30079700	-0.65625100
O	-0.67505000	-0.79551200	-1.35818300
O	-1.55123600	1.67731400	-0.03222200
O	-1.60144300	-0.70174700	1.42878300

TS3D

O	3.42827800	-0.74256900	1.01106200
C	4.42658100	-1.11750400	0.03408000
H	5.23641300	-1.56813700	0.62242900
C	4.88915300	0.07988500	-0.77879800
H	5.39065900	0.80151300	-0.11216200
H	5.63168900	-0.25578300	-1.51975300
C	3.68150000	0.73465400	-1.45923900
H	3.96748700	1.66109200	-1.97933200
H	3.27904100	0.04465700	-2.22278500
C	2.60291600	1.01474400	-0.44542700
C	1.74105300	2.06521300	-0.50487000
C	2.41741400	0.03063800	0.62725600
C	0.67339100	2.28790800	0.44961500
H	1.85476200	2.78169400	-1.32431800
C	1.50116100	0.36653100	1.77374800
C	0.55102200	1.49081300	1.52846800
H	-0.04993800	3.08255400	0.26394000
H	0.94301200	-0.52756300	2.09261200
H	-0.25860000	1.62775300	2.24624200
H	0.60455800	-0.80325900	-0.84340200
O	1.45955300	-1.23867500	-0.41375400
C	1.09088600	-2.45033200	0.24077200
H	0.92929700	-3.23273400	-0.51847200
H	1.92501400	-2.75394400	0.89180700
H	0.17510000	-2.32528800	0.83877700
H	3.98083000	-1.88840000	-0.61508800
H	2.16406500	0.61650700	2.62488800
Re	-2.02911800	-0.07615400	-0.13083100
O	-2.42506900	1.59882000	-0.00264000
O	-0.66509800	-0.29847200	-1.25146100
O	-1.53187700	-0.68254300	1.42000300
O	-3.39578900	-0.95711600	-0.69154700

TS4D

O	-2.61487800	-0.45642900	-1.39710300
C	-2.91453800	-1.76842300	-0.83913500
H	-3.63721200	-2.20496900	-1.54011200
C	-3.46417800	-1.64948700	0.57252800
H	-4.44142600	-1.13775600	0.53993300
H	-3.64032500	-2.66113300	0.96937300
C	-2.48602800	-0.87464100	1.46117500
H	-2.94099400	-0.63573900	2.43470300
H	-1.58888000	-1.48557400	1.65226800
C	-2.02793200	0.38543200	0.77125400
C	-1.46021100	1.45277000	1.44273400
C	-2.07242400	0.45494800	-0.64148300
C	-0.97027600	2.63821600	0.79960600
H	-1.38139300	1.38909600	2.53071400
C	-1.48084600	1.59920200	-1.37500400
C	-1.00581600	2.73459700	-0.55062400
H	-0.54349100	3.43206600	1.41355600
H	-0.54306100	1.18182800	-1.82392500
H	-0.60583500	3.60661200	-1.07188300
H	-1.96672900	-2.33110100	-0.86915100
H	-2.10136900	1.88705600	-2.23937900
Re	1.43093600	-0.26044800	-0.00512500
O	1.17780100	0.50656700	1.53643800
O	-0.03180300	-1.15169200	-0.37788900
O	1.57572600	0.97472400	-1.22425000
O	2.81365600	-1.29097900	0.03054600

TS1E

O	-2.63993300	0.51070900	-2.14166100
C	-3.74961800	-0.09671100	-1.51056600
H	-4.14789900	-0.92055000	-2.13388400
C	-4.83735100	0.95978800	-1.30794900
H	-4.88638300	1.54063000	-2.24432400
H	-5.81922500	0.47306000	-1.18639900
C	-4.61373600	1.91229700	-0.10485300
H	-5.22049200	2.82158600	-0.24544900
H	-4.97606300	1.42989200	0.81304000
C	-3.16646300	2.30078300	0.08893100
C	-2.53816900	3.23194600	-0.73020100
C	-2.37213400	1.66176800	1.07182600
C	-1.16022300	3.54825300	-0.65243400
H	-3.14591000	3.72926100	-1.49146000
C	-0.93776900	1.83701800	1.06891300
C	-0.36787600	2.88412300	0.24825700

H	-0.73553900	4.29579700	-1.32454300
H	-0.37069300	1.54078800	1.95554400
H	0.70991400	3.04348100	0.29485300
H	-1.81768000	0.17855600	-1.74720500
O	-2.98059500	0.81104600	1.88167700
C	-2.30194400	0.19526600	2.98255100
H	-1.82820200	0.95695600	3.62243500
H	-3.07945400	-0.32921700	3.55026500
H	-1.55809300	-0.53513100	2.62917700
H	-3.47075300	-0.55355800	-0.54011200
H	-0.70429400	0.85630100	0.31509300
Re	-0.29982700	-1.99177000	-0.09363200
O	1.31221300	-2.50320000	-0.40530900
O	-1.40838400	-2.91754400	-1.01593800
O	-0.66692900	-2.15449000	1.58202100
O	-0.45648900	-0.24105600	-0.54845800
O	1.85479000	1.14299800	-1.03898400
H	1.04336700	0.59782900	-1.05578700
C	2.86583000	0.46441200	-0.36527200
H	2.83815600	-0.63075600	-0.50494500
C	2.74825100	0.70337600	1.14957400
C	4.20458200	0.95005200	-0.93713700
F	5.23246000	0.30495500	-0.36706100
F	4.37898700	2.26388600	-0.74147900
F	4.24429900	0.71402300	-2.25038100
F	2.68732400	2.00917700	1.45382700
F	3.74432300	0.15496000	1.84138800
F	1.59023300	0.15143600	1.59698200

TS2E

O	-2.25564100	1.47220800	-1.03762400
C	-1.39691100	2.51905600	-0.54266500
H	-0.53530700	2.58454300	-1.22384000
C	-0.93176700	2.28502800	0.89238100
H	-0.26503700	1.41150000	0.92488600
H	-0.32807600	3.15361100	1.19963900
C	-2.09357100	2.05542200	1.88213500
H	-1.68566800	1.81220600	2.87387400
H	-2.70151500	2.96927300	1.98016700
C	-2.93899500	0.91212800	1.38340400
C	-2.83857800	-0.36540500	1.83871900
C	-3.70142000	1.16349300	0.15954300
C	-3.42894100	-1.50821000	1.16469700
H	-2.21361400	-0.55455500	2.71493600

C	-4.40729300	0.01678300	-0.52803700
C	-4.11041100	-1.34507600	0.01644200
H	-3.22056200	-2.50601200	1.55165200
H	-5.49136700	0.20496900	-0.40034200
H	-4.48993100	-2.20137700	-0.54509000
H	-1.74297900	0.57763500	-1.14195100
O	-4.24571600	2.37471500	0.10102000
C	-4.94727100	2.78164400	-1.07645300
H	-5.13571700	3.85575800	-0.96129300
H	-4.32674000	2.59778200	-1.96617600
H	-5.90924000	2.25317500	-1.17106300
H	-1.97889800	3.45009200	-0.62408400
H	-4.22639300	0.06237200	-1.61383500
Re	-0.08986000	-1.72210800	-0.16624600
O	1.39090400	-2.11727000	-0.98365100
O	-1.12317700	-0.73438300	-1.20218500
O	-0.92690700	-3.16098100	0.26849500
O	0.28075500	-0.81904600	1.26431200
O	3.58398300	-0.54053100	-1.13738000
H	2.87082500	-1.20929400	-1.21672400
C	3.25603200	0.31117900	-0.09653600
H	2.56789900	-0.12669100	0.64832900
C	2.53517000	1.55318700	-0.64400200
C	4.53914600	0.66091600	0.66765800
F	4.28271300	1.51324400	1.67716300
F	5.46034700	1.22451100	-0.12043400
F	5.06369600	-0.45032700	1.19474900
F	3.27775100	2.25576100	-1.49844700
F	2.13046500	2.38439100	0.33527600
F	1.41915000	1.15959400	-1.30477200

Int2E

O	0.76201500	2.32369200	0.52022700
C	0.56813800	2.35942800	1.95399300
H	1.27156200	3.12080800	2.31874600
C	0.82669700	0.98917600	2.56249300
H	1.88876200	0.72924700	2.43595800
H	0.63604100	1.03157300	3.64653600
C	-0.05954600	-0.07610800	1.90165200
H	0.28323100	-1.08414900	2.17431100
H	-1.09529300	0.02076900	2.27288800
C	-0.09879700	0.04705800	0.39869600
C	-0.23752900	-1.00294400	-0.43822500
C	-0.14132600	1.45332300	-0.18545000

C	-0.44052200	-0.84333400	-1.87578700
H	-0.24233200	-2.01257500	-0.02033900
C	0.28490900	1.49989600	-1.65581900
C	-0.24104200	0.34888300	-2.46442900
H	-0.73405700	-1.71749100	-2.46143000
H	0.02502800	2.47548400	-2.08786600
H	-0.35167800	0.47691700	-3.54369800
H	2.26337200	1.80605500	0.26033200
O	-1.47029800	1.91466300	0.02041600
C	-1.84938000	3.19407800	-0.48505500
H	-2.77302300	3.47087500	0.03992100
H	-1.06813700	3.94397600	-0.28486600
H	-2.06323800	3.14839800	-1.56461400
H	-0.45825600	2.70450300	2.15807600
H	1.38735800	1.44858800	-1.68755000
Re	3.45116300	-0.45451700	-0.13046400
O	5.14114000	-0.69839400	-0.16929300
O	3.15881700	1.35267000	0.15516300
O	2.77655700	-0.95327400	-1.62214200
O	2.77578000	-1.39399900	1.13233000
O	-3.33359000	0.43606900	1.22677300
H	-2.64342100	1.06180600	0.92512900
C	-3.66996300	-0.33134000	0.11890200
H	-2.86804600	-0.37049600	-0.63791200
C	-4.88181300	0.28091700	-0.59793700
C	-3.88759200	-1.77593600	0.57734200
F	-4.21217400	-2.56634900	-0.45776300
F	-4.84659100	-1.87875000	1.49913400
F	-2.74745200	-2.24942400	1.10995200
F	-5.97761100	0.30209700	0.16215700
F	-5.16753100	-0.37197000	-1.73327400
F	-4.58329200	1.55600400	-0.92785400

TS3E

O	-3.72136500	2.32912700	0.94523900
C	-4.48151900	3.03470100	-0.06354800
H	-4.78561800	3.97094300	0.42196700
C	-5.66480300	2.21204000	-0.54400400
H	-6.36346400	2.05383800	0.29465100
H	-6.20367200	2.77794300	-1.32000700
C	-5.16746300	0.86607900	-1.08383800
H	-6.00565500	0.20625900	-1.35319700
H	-4.58464700	1.04106000	-2.00653700
C	-4.27807900	0.19388300	-0.07061600

C	-4.17299400	-1.15061900	0.09458200
C	-3.39171000	1.05937300	0.72567500
C	-3.25986900	-1.77818000	1.03165700
H	-4.80370700	-1.79980300	-0.52050200
C	-2.67392800	0.46012700	1.90747900
C	-2.53999200	-1.02678200	1.88659300
H	-3.13848200	-2.86179900	1.01011900
H	-1.69168900	0.93876000	2.03824600
H	-1.83773700	-1.48007500	2.58761400
H	-1.71956600	0.39861400	-0.92234800
O	-2.07749300	1.30294300	-0.56862500
C	-0.97533800	2.14537000	-0.21424300
H	-0.44236400	2.43032800	-1.13191500
H	-1.38126200	3.04290700	0.27308200
H	-0.27232400	1.63092900	0.45533200
H	-3.79576200	3.26739400	-0.89448600
H	-3.26326300	0.76341900	2.79495000
Re	0.13914900	-1.67532100	-0.22755500
O	1.63318200	-1.93398600	-1.07106500
O	-1.03041600	-0.85849700	-1.27031700
O	-0.50153400	-3.18498300	0.29286700
O	0.43310000	-0.65667000	1.14601100
O	3.87680900	-0.40690200	-0.98794100
H	3.15856100	-1.05018900	-1.16403700
C	3.42170800	0.47753100	-0.02469900
H	2.69737500	0.04155800	0.68609400
C	2.68892200	1.65739300	-0.68441100
C	4.62152900	0.92585700	0.81730700
F	4.25387400	1.85353000	1.72135000
F	5.60078900	1.44522300	0.07174000
F	5.11753400	-0.12126400	1.48611300
F	3.48620900	2.42124700	-1.43214300
F	2.08910900	2.45304800	0.22547600
F	1.71248700	1.18022600	-1.48801900

TS4E

O	2.18339200	2.38512300	-1.27329600
C	0.91617800	3.07674800	-1.05139200
H	0.87820400	3.83396800	-1.84406500
C	0.85555300	3.67626400	0.34151500
H	1.61033700	4.47654700	0.42893200
H	-0.13411400	4.13782000	0.47540800
C	1.09242600	2.59396500	1.39994400
H	1.24174800	3.04164700	2.39459600

H	0.20857000	1.94006300	1.45331600
C	2.27747400	1.73416700	1.03320600
C	2.94606100	0.93349200	1.94154200
C	2.71535800	1.68397900	-0.31623900
C	4.06049800	0.09823500	1.61219300
H	2.59736700	0.93888100	2.97847900
C	3.79161500	0.76691500	-0.73613200
C	4.51396400	0.05496900	0.33243900
H	4.52546400	-0.49858100	2.39786500
H	3.22514200	-0.08136400	-1.24600800
H	5.35420600	-0.58102600	0.04566000
H	0.13000400	2.32615200	-1.20968000
H	4.41349400	1.18723100	-1.54160300
Re	0.83879700	-1.48341400	-0.19384300
O	1.38950900	-1.85655200	1.40060800
O	-0.52264700	-2.47401400	-0.63660400
O	0.33738500	0.18273400	-0.20640600
O	2.17658300	-1.63185900	-1.30625400
O	-2.99736600	-1.27418700	-0.89982000
H	-2.19437000	-1.83586400	-0.88985700
C	-2.80229200	-0.20791300	-0.04059300
H	-1.89058700	-0.29008300	0.56931500
C	-3.96105000	-0.14776300	0.96422500
C	-2.64427700	1.10893700	-0.82768500
F	-2.10535900	2.07775900	-0.04670400
F	-3.80624200	1.57858600	-1.29732900
F	-1.82420300	0.94187800	-1.87278000
F	-5.15655100	-0.13468000	0.37332800
F	-3.86615800	0.95175300	1.73950300
F	-3.90583100	-1.21766000	1.76863800

1r

O	-2.6292916596	0.1864063234	2.9559880874
C	-2.0095954131	1.1827532812	2.167590731
H	-2.7481038499	1.9168710901	1.7787972343
C	-1.3136858301	0.5026332067	0.9989134181
H	-0.594901083	-0.2309991221	1.4035957157
H	-2.0662676362	-0.0803592129	0.4400075406
C	-0.6009828294	1.4797068742	0.0635051608
H	-1.3301333687	2.2152607871	-0.3250552093
H	0.1406725391	2.0651319814	0.6387734912
H	-3.0706682386	0.6122962788	3.7007412756
O	1.4130029384	1.0316791503	-3.0808610357
C	2.1004613961	1.828642761	-4.0054487734

H	2.9244287727	2.4040396253	-3.5327266963
H	2.5335353501	1.1653803664	-4.7693154284
H	1.4313596328	2.5564281841	-4.5115182094
H	-1.2636796423	1.7637243425	2.751968944
C	0.0964347373	0.7878893967	-1.1084812626
H	-0.6345870277	0.2097267566	-1.6995593194
H	0.8368993197	0.0595785715	-0.7352415538
C	0.8056920306	1.7576757229	-2.0399566749
H	0.0804214591	2.4965386658	-2.4483751258
H	1.5649810528	2.344599949	-1.4762769795

2r

C	2.2348378877	-0.8319232871	-0.0924578693
H	2.328628379	-0.9246369276	-1.1973945857
C	2.3773156819	0.637392275	0.3106681732
H	2.4073553369	0.6974655612	1.4127907762
H	3.3337583925	1.0388195337	-0.065391851
C	1.1872465169	1.450162973	-0.21693283
H	1.2295110381	2.4907153611	0.1447475637
H	1.2402222495	1.4968658943	-1.3209021513
O	1.0020604768	-1.3778587969	0.3376059329
H	3.0262606268	-1.4495952256	0.3601199526
C	-0.1299145805	0.7791418889	0.196396323
H	-0.9967569113	1.2835606575	-0.2634823458
H	-0.2528769846	0.848531453	1.2914706572
C	-0.1172983374	-0.6990236439	-0.1995850297
H	-0.1202599473	-0.7860411784	-1.3090151537
H	-1.011087955	-1.2217617282	0.1756307979

HFIP-Int1

Re	-1.6113098252	-0.2316981263	-0.4873636271
O	-2.6653064026	1.0721686166	-0.1794621244
O	-2.0313070748	-1.5327182968	0.5309904922
O	-1.7802132852	-0.7207812924	-2.1131159717
O	0.1545077314	0.3465742509	-0.1759059216
C	1.4105492179	0.0897173691	-0.7348703317
H	1.3696560478	0.0656546678	-1.8374963402
C	2.3349936757	1.2554185607	-0.3423313466
C	1.9093815266	-1.2937868887	-0.279636558
F	2.4383100366	1.3797470222	0.9789332393
F	3.5550662878	1.0633436914	-0.8550467601
F	2.0884246373	-1.3555451956	1.0368314226
F	0.9780636586	-2.206532703	-0.6194014853
F	3.0486631157	-1.6105694641	-0.8926940625

F	1.8387562925	2.3913531383	-0.8405876648

Int1r

Re	-5.172933352	-0.0640752908	1.1855490307
O	-6.7733977217	0.29264208	1.6683560057
O	-4.5231222011	-1.2927671649	2.1844215327
O	-5.1543416505	-0.6128622799	-0.4342738961
O	-4.1588440748	1.4822763124	1.3408467177
C	-2.8395284486	1.8513496405	0.8987021663
H	-2.8862891897	2.0142921852	-0.1911552838
C	-1.7949315035	0.804816637	1.2575127919
H	-2.0358827703	-0.1396800281	0.7342099431
H	-0.8299970276	1.1404958699	0.8387085359
C	-1.6609971384	0.5494789758	2.7604586898
H	-1.3714299314	1.4908814966	3.2623837944
H	-2.6441305538	0.2687458179	3.1728272595
O	0.4218211818	-1.8404846879	4.7708799058
C	0.5961610067	-2.2060509425	6.1131715003
H	-0.3382019089	-2.6010444823	6.564012544
H	1.3622404136	-2.9948113513	6.1522168835
H	0.9372068923	-1.3539619934	6.7382407891
H	-2.6262332691	2.8144301327	1.3891171597
C	-0.6510900325	-0.5508640069	3.083136077
H	0.3493036831	-0.2823712525	2.7013688943
H	-0.9407405709	-1.4867997593	2.5756872424
C	-0.5380610629	-0.8329178416	4.5729825506
H	-1.527085133	-1.1442176056	4.9755359409
H	-0.2535633967	0.094300789	5.1192860046

Int2r

C	1.0592417982	2.754699864	3.5785083244
H	1.7421551149	3.4816269834	4.0482225573
C	0.0379380846	3.3924174116	2.663182804
H	-0.6556747819	2.6160514295	2.3006599282
H	-0.5542133177	4.0864525387	3.281775664
C	0.7021410583	4.1147168581	1.4867662077
H	-0.0642207838	4.5306194824	0.8170547807
H	1.2985656355	4.967668069	1.8556084823
O	1.9337675761	1.8312141338	2.7721255922
C	2.7856510482	0.9444999262	3.5715802105
H	3.2500516649	0.2360788074	2.8760863429
H	3.5408339273	1.5436539302	4.1013703191
H	2.1280273759	0.4163403625	4.2715251629
H	0.6113550751	2.1072516472	4.3425838282

C	2.6336670005	2.5009475198	1.6192780483
H	3.3312374027	3.2255498921	2.0707013497
H	3.1912804892	1.6915281034	1.1319191834
C	1.6007521329	3.140592261	0.7184002082
H	0.9983064146	2.3495524866	0.2423981144
H	2.1535186548	3.6501474131	-0.0876877383

TS1Cr

O	-2.7813184878	-0.3366980271	-2.4223452071
O	-2.066923346	-1.9113407835	1.3640465938
H	-1.9345859429	-0.7670723183	-2.1147765472
Re	-0.7483025483	-2.4130704792	0.343699975
O	-0.8897778057	-4.0895303092	0.0225439412
O	0.7717650231	-2.085736059	1.1234487983
O	-0.8373466921	-1.5169736837	-1.1685867301
O	-0.4853763202	1.0812566158	-0.145976514
H	-0.1129029173	0.4038163704	-0.7367435692
C	0.435324857	2.1165584773	0.0392803569
H	1.4627298553	1.7448416841	0.1693355511
C	0.0958168419	2.8200582116	1.3543509988
C	0.4296367512	3.0453414097	-1.1813928131
F	1.3578746051	3.9998434544	-1.0793129769
F	-0.7614666573	3.6391394704	-1.365608756
F	0.6892065701	2.3164150971	-2.2784008235
F	-1.1264854187	3.3873734418	1.3372356322
F	0.9865659328	3.7773259432	1.6231992995
F	0.1084720621	1.9333398864	2.3571952906
C	-3.4128957597	1.1643538068	2.044566064
H	-2.3232815714	1.2183774791	1.8883273672
C	-4.1123127853	2.3634521768	1.4230236577
H	-5.1908096789	2.2957730884	1.6507693907
H	-3.7338555274	3.2745033535	1.9125860155
C	-3.9103587596	2.4787850258	-0.0934393038
H	-4.4624323745	3.357934562	-0.4622187716
H	-2.8475492005	2.6603373075	-0.3151318369
O	-3.9347538437	-0.0242975811	1.4355161398
H	-3.6004271871	1.1229297402	3.1318589705
C	-3.5372751236	0.0366512617	-0.6240297478
H	-2.5067977427	0.1735328124	-0.3165187863
H	-3.9601739575	-0.9642902036	-0.6050959668
C	-4.3814611886	1.2461105631	-0.8849910777
H	-5.4344478165	1.0096308788	-0.6631297637
H	-4.3169768843	1.4609622482	-1.9634612141
H	-3.3302331595	-0.7799809156	1.6317830338

C	-3.5155458028	-1.2743715069	-3.2005294777
H	-3.6652655026	-2.2282105602	-2.6635237775
H	-4.4951218751	-0.8317630551	-3.4334947609
H	-2.9861822854	-1.4766981856	-4.1456818465
O	2.4262173057	-0.0269106464	1.0157008953
H	1.7597314126	-0.707035408	1.2637044176
C	3.3322068136	-0.6272100393	0.1507470278
H	3.2354865028	-1.7261333538	0.1310545985
C	4.75447258	-0.316674891	0.637716507
C	3.0730356923	-0.1476462027	-1.2866427556
F	3.8470311279	-0.7654758381	-2.1786691189
F	3.250036865	1.1760158534	-1.4144071753
F	1.7855278716	-0.4081417441	-1.610327651
F	4.9689737373	0.9994645757	0.7314622616
F	5.6740227335	-0.8321289147	-0.1936747567
F	4.9387750224	-0.858037089	1.8461989414

TS1r'

O	0.9358345579	0.6762261365	2.1746758257
O	0.8116548507	-1.1383106279	-1.292800967
H	0.4426676303	-0.1707013855	2.0662518481
Re	-0.1071959401	-2.2621014387	-0.2981680275
O	0.8196207653	-3.6743057367	-0.0139425247
O	-1.5871768789	-2.6737345925	-1.0710947329
O	-0.4704197952	-1.4564536986	1.2329659558
H	1.7702270687	0.5091310257	1.6965645483
O	-2.6470610124	0.1045304213	1.3302972297
H	-1.9214769091	-0.5535098608	1.4109366272
C	-3.5129774258	-0.2645371697	0.3070136828
H	-3.3926891606	-1.3113902234	-0.0203705365
C	-3.2364300658	0.5938041365	-0.9375510464
C	-4.9499305986	-0.1162316446	0.8248021243
F	-5.8458308328	-0.4291023333	-0.1228167165
F	-5.1993089196	1.1348721109	1.2345706241
F	-5.1376755625	-0.9389152863	1.8611422649
F	-3.3637583332	1.9074152749	-0.6854242877
F	-4.0312871799	0.2871511886	-1.961880721
F	-1.959819044	0.3891932032	-1.3344779081
O	2.4861376398	0.4704181018	-0.1846021835
H	1.9030826134	-0.1656891712	-0.6924965966
C	3.8290447879	0.1768684812	-0.3770407283
H	4.0401772798	-0.2857820657	-1.3571813069
C	4.2888539292	-0.8269269911	0.6930231323
C	4.583577192	1.5109021159	-0.3451865913

F	5.9043554777	1.3396655522	-0.4380958405
F	4.3204032547	2.1953966981	0.7757869788
F	4.1866805866	2.2672551752	-1.3845593586
F	4.1149279917	-0.3271742691	1.9280451445
F	5.5708712754	-1.1669497659	0.5492659611
F	3.5404183848	-1.9348261551	0.5944637609
C	-0.2990014593	4.2153572614	-1.0820596167
H	0.755770045	4.5218937717	-0.9334068421
C	-1.1911282779	4.7460140428	0.0253160097
H	-2.2297921485	4.4428125579	-0.1871790918
H	-1.1568684614	5.8471388983	-0.0081734577
C	-0.77713151	4.2419365716	1.4108376599
H	-1.4139673259	4.7139853834	2.1749304215
H	0.259557149	4.5571388925	1.6296280304
O	-0.3782516212	2.7853248139	-1.0884761034
C	0.3977204561	2.1558819548	-2.1125692734
H	0.2279277051	1.0755228379	-2.042598267
H	1.4718743063	2.3687086813	-1.9782567418
H	0.0588916329	2.5198627171	-3.0959485228
H	-0.6233216634	4.5805903855	-2.0724768943
C	0.036834509	1.9448091256	0.6979691965
H	1.0665896846	2.261615376	0.5585667897
H	-0.2739390108	1.0068865109	0.2523941334
C	-0.9033251485	2.719611919	1.5567013819
H	-1.9280665419	2.3755195704	1.3640560448
H	-0.6645309465	2.4229005221	2.5900945088

TS1r

Re	0.5602599197	1.8956627729	0.1243607781
O	0.3831895698	1.8842161491	1.8192848771
O	-0.7028822496	2.9127052705	-0.4111818046
O	1.9832215836	2.7197255907	-0.3218029603
O	-0.1720019269	0.3942057962	-1.0188512897
C	-1.5424343876	0.3301747474	-1.5083024428
H	-1.8121593281	1.3348952202	-1.8652535026
C	-2.4770291677	-0.1535968739	-0.4148876208
H	-2.1227742076	-1.1345161221	-0.0531268004
H	-2.4129834639	0.5444737122	0.4390851443
C	-3.9285639951	-0.2576581738	-0.8892339714
H	-4.2621689013	0.7293797291	-1.2581751834
H	-3.9831624235	-0.94311305	-1.7548986395
O	1.9876998235	0.1061059092	0.480532591
H	0.3020924248	-0.7496867195	-1.1085810305
Re	2.2089367422	-1.5840138808	0.0483418244

O	2.0836606974	-2.6149993661	1.4015858466
O	3.6990767056	-1.8543069973	-0.736287122
O	0.8201959732	-1.8069589149	-1.0742220471
O	-7.1032776747	-1.2968145375	0.8244970134
C	-8.467444022	-1.4256044697	0.52454751
H	-8.6485587898	-2.1528030668	-0.2944857729
H	-8.9795208592	-1.7851013355	1.4293551018
H	-8.9223614423	-0.4587364155	0.2236554992
H	-1.5246203639	-0.3603836625	-2.3665182892
C	-4.874707884	-0.7425977294	0.2097780537
H	-4.8313611981	-0.0622173758	1.0773445373
H	-4.557394492	-1.7338610773	0.5762293978
C	-6.3212554149	-0.8417687682	-0.2496688753
H	-6.4009830222	-1.5338048322	-1.1177677917
H	-6.6781962254	0.1500614714	-0.6065960305

TS2r

Re	0.1760005217	-1.8169643444	-0.2270230885
O	0.7857809574	-1.3358885653	1.3262184015
O	1.4462486416	-2.576665107	-1.0953746944
O	-1.1675391557	-2.8734063805	-0.0519535239
O	-0.3688308988	-0.3472480531	-1.1017064169
O	2.6890211942	0.5195000839	1.0365824897
H	2.1041188406	-0.1725017247	1.4142212258
C	3.6408569428	-0.0935859166	0.2205992883
H	3.3578262227	-1.1175576166	-0.0822640442
C	4.9762673243	-0.2090094088	0.9717727076
C	3.7367272372	0.7153569786	-1.0792019256
F	4.7058405655	0.2640409362	-1.8764639288
F	3.941362317	2.0152498029	-0.8491970993
F	2.5666938964	0.6066510536	-1.7476085995
F	5.4992514341	0.9904809612	1.2487341274
F	5.8729283596	-0.9061989145	0.2629237089
F	4.7683768131	-0.8521237076	2.1282018462
O	-2.9918308062	0.3172855597	-1.0086719309
H	-2.1124643503	-0.0582974665	-1.2174126733
C	-3.6027085511	-0.4326238384	-0.0071642825
H	-3.2145347023	-1.4633544606	0.0708152899
C	-5.0959998934	-0.5479140947	-0.3461443883
C	-3.3507871499	0.2247537874	1.3603495294
F	-3.9868233729	-0.3879225516	2.3554309373
F	-3.6905763782	1.5217420733	1.3690984524
F	-2.0197498354	0.1713280896	1.6335909481
F	-5.6886966999	0.6522749973	-0.3793494851

F	-5.7364222797	-1.3048551856	0.5556617142
F	-5.2381759983	-1.1191825827	-1.5445333187
C	-1.0783260126	4.0308477105	0.580669117
H	-1.9102529193	3.4066995293	0.9619011092
C	-1.2940187252	4.4102071554	-0.8758847793
H	-0.4508751316	5.0469460462	-1.1949119461
H	-2.2040315037	5.0294472848	-0.9348440331
C	-1.4260769219	3.2057068707	-1.816328972
H	-1.5714266954	3.5696305276	-2.8455302528
H	-2.3230882617	2.619077201	-1.556399348
O	0.1520810878	3.303064168	0.6774495115
C	0.5079579504	2.9062198097	1.9982803479
H	1.4879838521	2.415449093	1.9365868035
H	-0.235737652	2.2010635375	2.4127906094
H	0.5745541644	3.7917761989	2.6529903433
H	-1.0063452344	4.9290253234	1.2204829231
C	-0.0489207369	1.5391359613	-0.506396341
H	-0.9048917061	1.3678323606	0.1344680594
H	0.9336118751	1.2651613677	-0.1334452878
C	-0.203986809	2.2746132374	-1.7931704008
H	0.7327122228	2.8173672444	-1.9974545476
H	-0.2877530389	1.5084949685	-2.5805361823

TS3r

Re	-0.2090959054	-1.6260098984	-0.2544316138
O	0.561053286	-1.0868311315	1.1862103018
O	0.9655888213	-2.4334451616	-1.2380553466
O	-1.5287067233	-2.685540968	0.1090335028
O	-0.8439437198	-0.188606275	-1.080756556
C	1.5571987033	3.4966389258	0.3061056775
H	2.1924423085	3.2612748654	-0.568117761
C	1.8930271113	4.8614310907	0.8876701408
H	1.3128814188	5.0038502635	1.8150146868
H	2.9591756307	4.8693614735	1.1659274662
C	1.5649420197	5.9754075293	-0.1134655312
H	1.7389184793	6.9653509315	0.33583496
H	2.2407992502	5.8986142229	-0.9845632741
O	0.169539512	3.4576216812	-0.1083491345
C	-0.3633334296	1.6617365228	-0.6062907466
H	-0.077990621	1.321813685	0.3853620724
H	-1.3896517653	1.9687031036	-0.7786168905
H	0.3537521639	1.6402648298	-1.4193344935
H	1.6786315532	2.6892184008	1.0386701818
O	3.6399041657	-1.8124307978	-1.3147706018

H	2.7284115782	-2.1369782614	-1.4569274014
C	3.6974085457	-1.2276649672	-0.0608609917
H	2.9215187796	-1.5815482391	0.6397314353
C	3.4840995545	0.291383148	-0.1715451239
C	5.0459043803	-1.5819483614	0.5806691019
F	5.1980769024	-0.9427319017	1.7546094184
F	6.0766427623	-1.2566767771	-0.2032448801
F	5.0962285049	-2.8963151245	0.8164556766
F	4.4755898668	0.9267764213	-0.7980413585
F	3.3139036871	0.8718346002	1.028720317
F	2.3523796975	0.5290238735	-0.8844263328
O	-3.9044310051	-1.7504879486	1.1022335944
H	-3.1183120088	-2.2823126185	0.8709442929
C	-3.9541065359	-0.6630933252	0.2451076284
H	-3.2655820048	-0.7395455543	-0.6134188551
C	-3.5332334327	0.6077102395	0.9997978652
C	-5.3646177844	-0.562103945	-0.3529319031
F	-5.4648209913	0.5023625592	-1.1710727527
F	-6.3063153142	-0.4502385111	0.5865923039
F	-5.6189699923	-1.6585070683	-1.0748404214
F	-4.3542621043	0.9190174326	1.9987365658
F	-3.461719725	1.6790698428	0.1754762337
F	-2.2996010171	0.4343207541	1.5188574305
C	-0.1646369154	4.4424263791	-1.1076596904
H	0.4255334548	4.2347353413	-2.0213125462
H	-1.2292686587	4.2907206533	-1.3355455081
C	0.1103965107	5.8450804851	-0.5811858949
H	-0.5753834313	6.0459233141	0.2591747869
H	-0.1251775633	6.5719852659	-1.3754070313

TS4r

Re	-1.5650765504	-0.4371974919	-2.0340942854
O	-2.2798924413	-1.9531899197	-1.7253128519
O	-0.4732894211	-0.7611524945	-3.3130844287
O	-2.7502594362	0.5756006345	-2.7266903268
O	0.0928136663	0.4049583722	-1.0948895716
C	1.4694262763	0.4778171168	-1.5899227791
H	1.4487436998	0.1545756941	-2.6373904441
C	2.3876073204	-0.3802896255	-0.7416661625
H	2.289067929	-0.0768849596	0.311279869
H	2.0553287343	-1.4297423509	-0.8024711336
C	3.8483879558	-0.2546313404	-1.1795089347
H	3.9448034259	-0.5300926106	-2.2459235649
H	4.1580797246	0.8043351703	-1.1063777065

O	-2.2446290223	0.228409891	-0.090958825
H	0.0926945709	0.8641494533	-0.1240214555
O	7.022096071	-1.8176131713	0.0869792031
C	8.3945623281	-1.7824328432	-0.1960511859
H	8.821726884	-0.7649419159	-0.0727658476
H	8.9024513802	-2.4605056661	0.5059958155
H	8.6175419061	-2.1171759178	-1.2309840052
H	1.7497643171	1.5399687426	-1.5496834098
C	-3.6541551972	0.4014600506	0.1298094894
H	-4.1868608983	-0.5403135383	-0.0717776835
H	-3.7950454344	0.6943818922	1.1789770641
H	-4.0184020673	1.1901219137	-0.5412856051
O	-1.2663627709	-0.2096718401	2.1026217249
H	-1.8162345833	-0.0758197639	0.8390239272
C	-1.0445440278	-1.4685691167	2.617028967
H	-0.7399760682	-1.4454051189	3.6811545553
C	0.1041083695	-2.173004612	1.873206074
C	-2.3634186803	-2.2592677442	2.5711211752
F	1.240200492	-1.4832039414	2.0753335745
F	0.3017485062	-3.4249400742	2.2870120504
F	-0.1112034416	-2.2012286867	0.5413684411
F	-2.2392936318	-3.4708828675	3.1221825791
F	-3.3092145823	-1.5905775027	3.24257764
F	-2.7934689351	-2.4130115673	1.3071235426
O	0.3201016212	1.2888357708	1.1813061275
H	-0.3497222714	0.562102881	1.7805713641
C	0.249738041	2.6183303231	1.5569485688
H	0.1463930083	2.7297682975	2.6512434144
C	-0.9897039754	3.2910518305	0.9377984762
C	1.565407422	3.3210755434	1.1818522846
F	-2.0971379453	2.7384433017	1.4577915187
F	-1.0263443653	4.6037385454	1.1862235352
F	-1.0290595203	3.1115377044	-0.391282841
F	1.6263364933	4.5505363324	1.702123096
F	2.6031363037	2.617151633	1.6444865031
F	1.7075695812	3.4281323901	-0.1532975741
C	4.7860558937	-1.1201464683	-0.3369896916
H	4.4962734301	-2.1820795252	-0.4142300198
H	4.694143766	-0.8510207539	0.7291511477
C	6.2455527753	-0.9897742082	-0.7424946194
H	6.3730303302	-1.2723214626	-1.8113393453
H	6.5729800442	0.0705536149	-0.6582064301

TS1F

Re	1.875553054	-1.0316095006	1.0005060446
O	1.3486348754	-1.3672098121	2.5831251552
O	3.3378153506	-0.1685102685	1.2229368898
O	2.3903988409	-2.4834289616	0.274465443
O	1.231113082	0.685457343	-0.0122188067
O	-0.0914723897	-1.4575930131	0.2268891931
H	-0.0705043	1.0120044179	-0.2411526782
Re	-1.4353282448	-2.0408153061	-0.7885231057
O	-2.5438876172	-2.9653475089	0.1165260522
O	-0.8748061261	-2.9769474656	-2.0959835752
O	-2.2051657679	-0.5649675706	-1.3927120842
C	-1.6762887141	2.1024126438	0.6146096478
H	-0.9659069102	2.8772944277	0.9329869781
C	-2.9126578466	2.8130333546	0.0411464196
C	-1.952202657	1.202867384	1.8313650893
O	-1.0907692989	1.3490018526	-0.4292054719
H	-1.7072029709	0.5459211263	-0.9017344068
F	-3.7924622044	1.9228601874	-0.4276729897
F	-3.5023521184	3.5536197074	0.9793379172
F	-2.7439279597	0.1732143652	1.5057058427
F	-0.7843945621	0.7039644744	2.2658875513
F	-2.5145469954	1.8871391564	2.8208559705
F	-2.5377433424	3.6072576068	-0.9613375216
C	2.118980758	1.5940191906	-0.6145389101
H	3.1438326089	1.4453316507	-0.2406843792
C	2.1309494569	1.3030978077	-2.1217360628
C	1.7479981695	3.0420026297	-0.2558990707
F	0.8892992083	1.3063091161	-2.6260005995
F	2.8687524756	2.1904376314	-2.7877175563
F	0.6474635475	3.4701116228	-0.8988764279
F	1.4898160004	3.1208196042	1.0609248033
F	2.7493621299	3.8664509505	-0.5462268156
F	2.6454504681	0.0792211561	-2.3139205357

TS1G

Re	0.5476985711	1.8372007892	0.0422273281
O	0.1040665872	2.7025978654	-1.352578697
O	2.25435592	1.9581104661	0.0573800925
O	0.0764847458	2.7254270565	1.4136237554
O	0.898143281	-0.2490481419	0.0690421586
O	-1.4288434875	0.9129084018	0.0300956734
H	0.0098401917	-1.1074096939	0.0811097494
Re	-2.3389723734	-0.6118448194	0.0369791998
O	-3.2759802069	-0.813186796	-1.3702034263

O	-3.3395285228	-0.7651345272	1.4059972208
O	-0.9858892497	-1.7755287457	0.0876173313
C	2.1801573417	-0.8454904169	-0.0025970533
H	2.9537099205	-0.0640189609	0.0145895391
C	2.3909011296	-1.71571728	1.243327198
C	2.3030704269	-1.5749662879	-1.3468328931
F	1.4866259621	-2.6968444588	1.3199467083
F	3.6105584428	-2.2530014638	1.239442642
F	1.394743174	-2.5482976633	-1.4651110919
F	2.0921048807	-0.6894411324	-2.3316515545
F	3.5189210169	-2.0982212217	-1.4939178623
F	2.2621302482	-0.9451749692	2.3309039818

1a-O radical

O	-1.66578300	-0.61336900	1.54324000
C	-2.68796800	-0.16307200	0.76611200
H	-3.25743100	-0.98508900	0.26934600
C	-2.26624900	0.90503600	-0.25128600
H	-3.16193800	1.33746600	-0.73045200
H	-1.74315900	1.71196100	0.28389400
C	-1.33227200	0.31586800	-1.32737100
H	-1.89403000	-0.41197900	-1.93623200
H	-1.01826300	1.13096000	-1.99975100
C	-0.11410200	-0.36199100	-0.74988100
C	0.01119200	-1.75576400	-0.72193500
C	0.93008400	0.41329400	-0.18855900
C	1.13144100	-2.38362400	-0.16649600
H	-0.79419000	-2.36117900	-1.14710100
C	2.05490700	-0.20495900	0.36936200
C	2.14880400	-1.60281200	0.37777900
H	1.20217600	-3.47346700	-0.15636900
H	2.86035600	0.38753600	0.80285100
H	3.03033600	-2.07538200	0.81881000
O	0.74997500	1.76134200	-0.24274500
C	1.72286900	2.61584500	0.31419200
H	2.70220600	2.50663800	-0.18696200
H	1.36059700	3.64232200	0.16598000
H	1.85624100	2.43531700	1.39639500
H	-3.40866700	0.30418800	1.48412200

Int1-C radical

C	-2.87860000	-0.56125000	1.14183300
H	-2.14522000	-1.32541700	1.41449100
C	-2.54472500	0.47970000	0.13068300

H	-3.47085400	0.89562600	-0.30659600
H	-2.04167100	1.34276400	0.61647900
C	-1.62301000	-0.00832200	-1.01271200
H	-2.12474700	-0.82437700	-1.55719000
H	-1.48450000	0.82410500	-1.72110300
C	-0.27059800	-0.48568200	-0.53933700
C	0.05084300	-1.84390900	-0.46498600
C	0.70631400	0.45205300	-0.12681300
C	1.29674800	-2.28453700	0.00011900
H	-0.69833000	-2.57487000	-0.78249200
C	1.95706400	0.02334500	0.33768700
C	2.24559000	-1.34587900	0.40034000
H	1.51937100	-3.35303300	0.04652000
H	2.71190400	0.74361200	0.65303000
H	3.22360100	-1.66997000	0.76557200
O	0.33892400	1.76138100	-0.22053700
C	1.24981800	2.76867200	0.15481600
H	2.16938000	2.74235900	-0.45839400
H	0.74105300	3.72854800	-0.00871000
H	1.53362000	2.69187300	1.22062200
H	-3.78167400	-0.47741100	1.75228800

ReO_4 radical

Re	0.02306800	-0.00000600	-0.00051200
O	0.95789900	-1.39589800	0.30244800
O	-0.62247700	-0.01338500	-1.61514100
O	-1.50696000	0.00711000	1.03709200
O	0.95527300	1.40222900	0.28040600

ReO_3 radical

Re	0.00000000	-0.00005000	-0.09566100
O	-1.44093200	-0.83316200	0.29897800
O	1.44228400	-0.83082100	0.29897800
O	-0.00135100	1.66445300	0.29886700

B

C	2.16707200	0.07655700	-1.03726600
H	2.14208900	0.85498400	-1.82546600
C	2.27379200	0.72302000	0.33767500
H	2.31310200	-0.07368800	1.09951000
H	3.20989100	1.29980800	0.40715700
C	1.05124800	1.61401000	0.57197900
H	0.99652500	1.93651800	1.62442700
H	1.14647300	2.53771600	-0.02855900

C	-0.22172300	0.88876800	0.18808700
C	-1.47680800	1.29382900	0.66602600
C	-0.16696000	-0.23713100	-0.65688000
C	-2.64631000	0.61143400	0.32805000
H	-1.52679300	2.16399800	1.32804000
C	-1.33587000	-0.93457000	-0.99539000
C	-2.56949200	-0.51124900	-0.50612500
H	-3.61057500	0.94753500	0.71625300
H	-1.24412300	-1.80899100	-1.64273000
H	-3.47530800	-1.06043800	-0.77552600
O	1.00324400	-0.73798800	-1.14939900
C	0.73231600	-2.38196000	1.79737100
H	-0.07367400	-1.69827700	2.06767900
H	1.07272500	-2.41419300	0.76199700
H	1.16336200	-3.04763200	2.54696700
H	3.01676700	-0.58968900	-1.24571700
